# Mapping molluscan endocrinology: a systematic and critical appraisal

**DOI:** 10.1002/brv.70112

**Published:** 2025-12-16

**Authors:** Konstantinos Panagiotidis, Thomas H. Miller, Olwenn V. Martin, Alice Baynes

**Affiliations:** ^1^ Environmental Sciences, Department of Life Sciences Brunel University of London Kingston Ln London Uxbridge UB8 3PH UK; ^2^ Department of Arts and Science, Faculty of Arts & Humanities University College London Gower St London WC1E 6BT UK

**Keywords:** cholesterol, steroid, hormone, thyroid, retinoid, ecdysteroid, phytosterol, mollusc, sterol, steroidogenesis, endocrinology

## Abstract

Historically, a vertebrate‐centric paradigm has framed our interpretation of molluscan endocrinology, with considerable research focusing on vertebrate‐type steroid hormones (e.g. oestrogens, testosterone). However, contradictory evidence on the occurrence of vertebrate‐type steroid hormones in molluscan tissues, and a lack of the specific steroidogenesis enzymes involved in producing these steroids has fuelled an ongoing debate about the ability of molluscs to biosynthesise vertebrate‐type steroids *de novo*. Consequently, the exploration of other hormonal pathways that may exist in the phylum remains a significant knowledge gap. This study systematically identified, combined and evaluated evidence from 147 eligible studies (published between 2012 and 2021) on the occurrence of hormones, hormone receptors and hormone‐metabolising enzymes in Mollusca according to the 2015 PRISMA‐P systematic review guidelines and the 2020 COSTER guidelines. The data collected are holistically summarised and visualised in a fully searchable, interactive and openly accessible online database using Tableau Public 2023.1 software. A critical appraisal assessment (Risk‐of‐Bias tool) accompanied by tailor‐made guidelines as well as a narrative synthesis using comparative endocrinology is presented. Strikingly, 95% of studies measuring hormones in molluscs did not investigate the hormones' ability to bind to their respective receptors. Moreover, many studies either used methods now considered unreliable (e.g. lack specificity) to identify relevant biomolecules (i.e. hormones, receptors, enzymes) or did not employ robust internal validation steps, with 83% of all studies not independently repeating their experiments. This highlights an urgent need for greater experimental rigour in the field. Most studies were also found to be heavily skewed towards vertebrate‐type sex steroidogenesis, with 66% measuring 17β‐oestradiol in mollusc tissues, despite unconvincing evidence that molluscs can biosynthesise vertebrate‐type steroids. By contrast, the retinoic acid signalling pathway, known to be more evolutionarily conserved (and a target of environmental pollution), has received far less attention. However, a limited number of studies are now looking beyond vertebrate‐type sex steroids, notably those looking at thyroid hormones, phytosterols (plant sterols) and ecdysteroids (insect steroids) in molluscs. These studies should act as a catalyst to spark interest in further exploration of understudied or unexplored hormonal pathways in Mollusca to elucidate fully the endocrinology of this important phylum.

## I. INTRODUCTION

Molluscs are known for their incredible diversity. The number of known mollusc species estimated at 81,000–92,000 living species (MolluscaBase eds., 2025) is second only to insects. With a rich fossil record (60,000–100,000 fossil species; MolluscaBase eds., 2025) dating back to the Cambrian (500 million years ago), molluscs have adapted and radiated into almost every environment from deep ocean trenches and hydrothermal vents, open seas and intertidal shores, freshwater rivers and lakes, to terrestrial locations including deserts and mountains. Molluscs display diversity at many levels. Feeding strategies range from giant clams with symbiotic photosynthetic zooxanthellae (Klumpp, Bayne & Hawkins, 1992), to herbivores and detritivores (e.g. typical garden slugs and snails) and carnivores (e.g. squid). Reproductive strategies include gonochorism, hermaphroditism (both sequential and simultaneous) and parthenogenesis, with a range of parental care levels (broadcast spawners and prolific egg layers, to brooders). Molluscs also exhibit a variety of body plans, structures and levels of behavioural complexity. To humans, molluscs can represent food (e.g. oysters), agricultural pests (terrestrial slugs), parasite vectors (e.g. schistosomiasis), or have ornamental uses (pearls and shells). However, given their ubiquitous and diverse nature, molluscs are vital components of major ecosystems now threatened by climate change, habitat destruction, and pollution (Abreu *et al*., 2019; Böhm *et al*., 2021; Cuttelod, Seddon & Neubert, 2011; Thomas *et al*., 2021).

A clear example of how pollution can disrupt mollusc populations and lead to regional extinctions is the case of the anti‐fouling chemical, tributyltin (TBT). In the 1970s two seemingly unrelated disruptions to mollusc populations occurred, namely reports from American and European harbours of normally gonochoristic female marine gastropods developing male sexual structures (penis, vas deferens, prostate) known as ‘imposex’ (Blaber, 1970; Smith, 1971), and the collapse of the oyster fishery in Arcachon bay, France (Ruiz *et al*., 1996). Both were later linked to widespread use of TBT antifoulant on boat hulls (reviewed in Santillo, Johnston & Langston, 2001). The imposex condition, caused by TBT exposure, is known to have driven population declines of marine snails globally (Fernandez, 2019). The link between TBT pollution and imposex has been credited as a clear warning of the risk posed by endocrine‐disrupting chemicals on wild species (or humans) (Fernandez, 2019). Testing chemicals for possible endocrine‐disrupting activity is now being implemented in a number of countries and regions (e.g. EU, USA), with a growing number of internationally recognised testing methods and protocols available (e.g. OECD, 2018). However, somewhat paradoxically, TBT's mechanism of action leading to imposex in molluscs was misunderstood for decades and has only recently become better resolved (Castro *et al*., 2007; Giulianelli *et al*., 2020; Lesoway & Henry, 2021; Nishikawa *et al*., 2004; Zhou *et al*., 2021). Indeed, although molluscs could be viewed as an early indicator of the issues of endocrine‐disrupting chemicals, molluscs are yet to be properly integrated into endocrine‐disrupting chemical testing guidelines due to a lack of detailed understanding of their endocrinology. Given their economic and environmental value, this paucity of knowledge on mollusc endocrinology is in stark contrast to the wealth of information we have on vertebrate hormone systems (vital for medical and pharmaceutical interventions), as well as our understanding of insect hormones (used to develop insecticides) and plant hormones (used to support agricultural innovations).

Historically, the identification of vertebrate‐type sex steroids such as androgens (e.g. testosterone) or oestrogens (e.g. 17β‐oestradiol) in the tissues of molluscs led to the assumption that these animals have the ability to biosynthesise or metabolise such steroids *de novo* (Lafont, 1991; Lafont & Mathieu, 2007; Lehoux & Sandor, 1970). Moreover, the presence of nuclear receptors (NRs) homologs, such as the oestrogen receptor, identified from sequence data (Ip *et al*., 2016; Lü *et al*., 2016), along with the detection of certain vertebrate‐type steroidogenic enzymes in molluscan tissues using non‐specific techniques (Prisco *et al*., 2017; Rosati *et al*., 2019b) further supported the hypothesis of endogenous vertebrate‐type sex steroid synthesis in this phylum. Contradictory findings, from researchers exposing freshwater gastropods (*Biomphalaria glabrata*, *Lymnaea stagnalis*) to potent vertebrate androgens [testosterone, 5α‐dihydrotestosterone (DHT) or 17α‐methyltestosterone (MT)], showed no effects on growth, reproductive development or reproductive output (eggs per individual) (Giusti *et al*., 2014; Kaur *et al*., 2016). These findings were supported by molecular investigations which demonstrated that *B. glabrata* and the marine limpet, *Lottia gigantea*, do not have a nuclear androgen receptor, indeed, the whole 3C group of NRs (including the glucocorticoid receptor, mineralocorticoid receptor and progesterone receptor) were absent in these species (Giusti *et al*., 2014; Kaur, 2015). Additional genomic and evolutionary searches revealed that molluscs (and other invertebrates) do not contain the cholesterol side‐cleavage enzyme (encoded by *CYP11A1*) that is essential for the induction of vertebrate steroidogenesis (Adema *et al*., 2017; Markov *et al*., 2017). These conflicting observations led to an ongoing debate regarding the ability of molluscs to biosynthesise vertebrate‐type steroids *de novo* and what their role in molluscan endocrinology could be (Scott, 2012, 2013). However, our current understanding of molluscan endocrinology is characterised by significant lack of knowledge beyond potential similarities with vertebrate steroidogenesis.

To date, the most thorough discussion on molluscan endocrinology is a series of critical reviews by Scott (2012, 2013, 2018) and Fodor *et al*. (2020). While these reviews provide valuable information on the occurrence of vertebrate‐type sex steroids in molluscs, the exploration of other hormonal pathways that may exist within the phylum remains a significant knowledge gap. Therefore, the aim of this systematic evidence map is to provide a comprehensive assessment of our current understanding of hormone biosynthesis in molluscs. This is achieved through a critical evaluation of the wider literature, bringing together evidence for different hormones, hormone receptors and metabolic pathways present in molluscs. This new evidence base raises important questions and highlights critical knowledge gaps which should guide future research efforts.

## II. METHODS

The systematic evidence map protocol was drafted according to PRISMA‐P (Preferred Reporting Items for Systematic review and Meta‐Analysis Protocols) 2015 guidelines (Shamseer *et al*., 2015) together with the COSTER (recommendations for the Conduct Of Systematic reviews in Toxicology and Environmental health Research) checklist (Whaley *et al*., 2020). The second version of the protocol was developed after receiving feedback during open peer review, and is published on the open repository Zenodo (Panagiotidis, 2022). Additional iterations were made to improve the clarity of the protocol resulting in a third and final version (see online Supporting Information, Appendix S1 and S2).

### (1) PO statements and eligibility criteria

In this review, each research question is defined by a separate PO (Population, Outcome) statement as well as PO‐specific inclusion and exclusion criteria; these are summarised in Table S1.1 of Appendix S1. The Mollusca AND Hormones PO aimed to identify the presence of hormones found in molluscan tissues; the Mollusca AND Receptors PO aimed to identify the presence of hormone receptors in molluscan tissues; and the Mollusca AND Enzymes PO aimed to identify the presence of hormone‐metabolising enzymes in molluscan tissues.

#### (a) Defining ‘population’

The population was defined as Mollusca, including all seven molluscan living classes (Gastropoda, Bivalvia, Polyplacophora, Cephalopoda, Scaphopoda, Aplacophora, Monoplacophora). The seven molluscan classes were included as search terms in addition to several other mollusc‐specific terms including ‘oysters’, ‘mussels’, ‘squids’ and ‘chitons’.

#### (b) Defining outcome – ‘hormones’, ‘hormone receptors’ and ‘hormone‐metabolising enzymes’

Three sets of keyword strings relevant to our research questions and PO statements were devised to capture the published literature. Full details of the database searches and keyword strings used are provided in Appendix S3. Searches were conducted in *PubMed*, *Web of Science* and *Scopus*.

The keyword string for Mollusca AND Hormones included 24 steroid hormone names involved in vertebrate steroidogenesis (Table S3.1 in Appendix S3). These were extracted from Häggström & Richfield (2014) and Fodor *et al*. (2020). Additionally, four identified ecdysteroids involved in arthropod steroidogenesis were extracted from Niwa & Niwa (2014) and were included as search terms. To avoid missing important literature, generic terms such as ‘sterols’, ‘hormones’ as well as synonyms for each included steroid were identified and included. Data on retinoids were captured *via* the Mollusca AND Receptors keyword string (see below). Data on hormones involved in neurohormonal signalling were outside the scope of this review.

The Mollusca AND Receptors search string (Table S3.2 in Appendix S3) included general hormone receptor terms, such as ‘hormone receptors’, ‘nuclear receptors’ and ‘retinoid receptors’. The aim of this search was to collect data on the occurrence of receptors known to interact with hormones and retinoids, as well as receptors known to be indirectly involved in hormone signalling in molluscs. The Mollusca AND Receptors PO was amended from the originally proposed ‘Mollusca AND Nuclear receptors’ PO (Panagiotidis, 2022) which was intended to address the occurrence of NRs in molluscan tissues. However, our pilot searches captured a considerable number of receptors outside the NR superfamily, and therefore the PO statement was updated accordingly.

Lastly, the Mollusca AND Enzymes search string aimed to capture information on the enzymes involved in steroidogenesis and retinoid signalling in molluscs, referred to herein as ‘hormone‐metabolising enzymes’ (Table S3.3 in Appendix S3). A list of genes encoding enzymes involved in vertebrate steroidogenesis was identified and extracted from Wikipedia (Häggström & Richfield, 2014). In addition, the keyword strings included names of 10 genes known to be involved in insect steroidogenesis (Niwa & Niwa, 2014) and names of key transport proteins and enzymes involved in retinoid signalling. The Mollusca AND Enzymes PO represents an amended version of the initially proposed ‘Mollusca AND Steroidogenesis‐related genes’ (Panagiotidis, 2022), intended to address the occurrence of steroidogenesis‐related genes in molluscs. The data captured from the systematic searches revealed a range of hormone‐metabolising enzymes outside the scope of vertebrate steroidogenesis, requiring the PO statement to be updated accordingly.

### (2) Search strategy

Eligible studies included in the systematic map were any peer‐reviewed publications written in English, that met the eligibility criteria presented in Table S1.1 (Appendix S1). Grey literature was not included. Data from review studies were excluded, although relevant review studies were used as information sources for discussion of our results. Searches of reference lists from eligible studies were carried out to capture any additional articles not uncovered by the main search.

The date range for inclusion was 1st January 2012 to 10th September 2021 (the date the search was conducted). The 2012 cut‐off date was driven by two factors. (*i*) The first molluscan draft whole‐genomes were published in 2012 (Takeuchi *et al*., 2012; Wang *et al*., 2012), providing high‐quality and detailed molecular data not previously available; (*ii*) Traditional immunological‐based assays [e.g. radioimmunoassay (RIA), enzyme‐linked immunosorbent assay (ELISA)] employed to measure hormones in molluscan tissues were widely considered to be sensitive and reliable (Lavado, Janer & Porte, 2006; Liu, Li & Kong, 2008; Warrier, Tirumalai & Subramoniam, 2001). However, the reliability and specificity of applying antibodies for vertebrates to molluscs came into question in the early 2010s with the emergence of studies that compared immunoassay techniques with analytical chemical methods (Gust *et al*., 2010; Krasowski *et al*., 2014), showing that analytical chemistry [e.g. gas chromatography–mass spectrometry (GC–MS), liquid chromatography‐mass spectrometry (LC–MS)] exhibited higher precision in detecting low concentrations of steroids and other metabolites than traditional immunoassay methods (Gust *et al*., 2010).

Date limitations were not applied during the literature search, as this resulted in significant inaccuracies during citation export in trial searches (e.g. the number of exported citations did not match the number of retrieved papers).

### (3) Data management and screening

The studies captured by the systematic searches and across all databases, were merged and then exported to the reference management software *Zotero* to facilitate the process of duplicate removal. Following duplicate removal, the remaining studies were imported to the online tool *Rayyan* (Ouzzani *et al*., 2016) for title and abstract screening. In an initial step, the eligibility criteria from the draft version of the protocol (Panagiotidis, 2021) were applied to 20% (*N* = 1377) of the merged list of eligible studies, by two coders (K.P., A.B.) working independently. This pilot screening of retrieved studies allowed us to identify potential limitations with the eligibility criteria of the draft protocol. Disagreements after pilot screening were resolved between the two coders and eligibility criteria were updated accordingly in the second version of the protocol (Panagiotidis, 2022). Following amendment of eligibility criteria, the remaining 80% of studies were screened by a single evaluator (K.P.) at title and abstract level. Eligible studies were then screened at full‐text level, by the same evaluator, and reasons for exclusion were recorded. Studies found eligible for inclusion at full‐text screening were included in the data‐extraction inventory. During full‐text screening, final amendments were made to the eligibility criteria. For example, the presence of thyroid hormones, thyroid receptors or enzymes that metabolise thyroid hormones (or those involved in thyroid hormone signalling) were not part of the initial inclusion criteria. However, identification of those biomolecules during full‐text screening provided an opportunity to include discussion on the occurrence of the thyroid hormone pathway in molluscs. Thus, the occurrence of thyroid biomolecules became part of the inclusion criteria, across all PO statements (Table S1.1 in Appendix S1). Full details on amendments made to the eligibility criteria are described in Appendix S2.

### (4) Data extraction

Data on hormones, hormone receptors and hormone‐metabolising enzymes were extracted from eligible studies and included in the data‐extraction inventory entered into the Excel data‐extraction template (Appendix S4). The data‐extraction template was designed to record information for the three PO statements and was piloted with nine eligible studies. The results of the pilot activities are summarised in the draft version of the protocol (Panagiotidis, 2021). Upon completion of the full‐text screening and subsequent data extraction, the data‐extraction template was amended slightly for purposes of clarity and data interpretation. The data‐extraction template and inventory are provided in Appendix S4, with changes made to the data‐extraction template summarised in Appendix S2.

### (5) Risk‐of‐bias assessment

Limitations in the analysis or experimental design of individual studies can result in incorrect assumptions about the origin and synthesis of hormones in molluscan tissues. Critical appraisal tools can be used to assess the internal validity of studies through selection bias, detection bias, performance bias, etc. (Martin *et al*., 2021). Although attempts have been made to evaluate bias in studies that investigate sex steroid biosynthesis in molluscs (Scott, 2013), to our knowledge, there is no Risk‐of‐Bias (RoB) tool adapted for endocrinological investigations in these animals. For the purposes of this systematic evidence map, a tailor‐made RoB tool (Appendix S5) was developed to assess critically potential flaws or errors in the design, conduct or reporting of eligible studies. The tool consists of a series of criteria that aimed to evaluate the collected data in respect to each PO statement. Eligible studies were assessed on both internal validity (e.g. verification of mechanism of action of hormones) and study design criteria (e.g. within‐ or between‐study repetition) and these are summarised in the accompanying RoB guidelines (Appendix S5), together with detailed information on how studies are coded (Appendix S6). Internal validity criteria were specific to each PO, whereas study design criteria were applicable to all PO statements. The assessment criteria were created using information from peer‐reviewed literature and the ARRIVE guidelines 2.0 (Percie du Sert *et al*., 2020a). After the second version of the protocol was published (Panagiotidis, 2022), modifications were made to the RoB tool and guidelines. These changes were introduced to refine the reliability and objectivity of the RoB assessment and are addressed in detail in Appendix S2.

The outcomes reported in the included studies varied considerably, so a single approach for RoB was difficult to implement. Therefore, two assessment categories were used. The Assessment A category evaluated both internal validity and study design criteria, whereas Assessment B evaluated study design criteria only. Studies were allocated to the appropriate assessment category during data extraction (see Appendix S5).

Assessment A studies focused on the investigation of activity, function and/or mechanism of action (MOA) of hormones, hormone receptors, and/or hormone‐metabolising enzymes in molluscs. Consequently, the study objectives should adhere to a methodology considered appropriate for an internal validity assessment. Assessment B studies were any studies that did not fit within the scope of Assessment A study criteria (e.g. toxicological assessments). If the study did not determine (or attempt to comment on) the activity, function and/or MOA of an outcome of interest it was assessed solely on study design criteria (i.e. Assessment B), but was included in the systematic map to broaden the range of information captured.

The RoB tool (Appendix S5, Questionnaire tab) consists of a series of questions that require the evaluator to allocate a score of Definitely low risk of bias (++), Probably low risk of bias (+), Probably high risk of bias (−) and Definitely high risk of bias (− −). Each study then received a summary score: Level 1 = studies with lower risk of bias; Level 2 = studies with moderate risk of bias; Level 3 = studies with higher risk of bias (see Appendix S5, RoB summary score tab).

### (6) Data analysis and narrative synthesis

The primary aim of this systematic evidence map was to provide a narrative synthesis of the available data, based on the three PO statements, highlighting key findings for different molluscan classes. The RoB assessment is also a primary outcome of this review, identifying multiple levels of reliability for the collected evidence. This synthesis also allows identification of knowledge gaps within our current understanding of molluscan endocrinology.

#### (a) Mollusca AND hormones

Hormones were classified according to their chemical structure as steroids, retinoids, biogenic amines or protein hormones, and into subgroups (e.g. phytosterols, sterols, fungal sterols, zymosterols, secosterols). The structure of hormones was determined from online databases (Kim *et al*., 2023; Sud *et al*., 2007) or molecule‐specific information found in the respective publications.

#### (b) Mollusca AND receptors

Data for both receptor genes and proteins were collected from eligible studies. The identified receptors are reported by their nomenclature gene and enzyme name, verified through the nomenclature databases Genenames.org, Flybase.org and WormBase.org (Davis *et al*., 2022; Gramates *et al*., 2022; Seal *et al*., 2023). Receptors whose names could not be identified are reported by the names used in the respective publication. Receptor genes and proteins were categorised by type (cell surface receptor, G‐protein‐coupled receptor, intracellular receptor, ligand‐gated ion channel, NR, unknown), phylogenetic origin (vertebrate or non‐vertebrate) and mollusc class (Bivalvia, Cephalopoda, Gastropoda, Polyplachophora). The classification of receptors by type was achieved through manual searches in the online database *UniProt* (UniProt Consortium, 2023). For clarity, receptors primarily known from vertebrates (e.g. oestrogen receptors) are described herein as ‘vertebrate‐type’ receptors, whereas receptors primarily known from invertebrates (e.g. ecdysone receptor) are defined as ‘non‐vertebrate‐type’ receptors.

The receptors included in the Mollusca AND Receptors inventory were identified by a range of molecular assays. To assess the reliability of the collated evidence, these publications were classified into three primary categories based on the methodologies employed: (*i*) DNA/RNA detection and localisation techniques; (*ii*) DNA/RNA detection and localisation techniques and other *in vitro* assays; (*iii*) protein and other *in vitro* assays.

#### (c) Mollusca AND enzymes

Information about genes, enzymes and proteins associated with the synthesis of cholesterol, steroids, thyroid hormones, and retinoids was included in the Mollusca AND Enzymes inventory. Hormone‐metabolising enzymes were classified according to their metabolic or signalling pathways (steroid biosynthesis, cholesterol biosynthesis, retinoic acid signalling, etc.). The classification of hormone‐metabolising enzymes according to their respective pathways was achieved through manual searches in the online database *UniProt* (UniProt Consortium, 2023). Data from a range of molecules indirectly involved in the main metabolic or signalling pathways of interest were also collected. These include members of the cytochrome P450 superfamily of enzymes known to be involved in xenobiotic metabolism as well as the synthesis of cholesterol, steroids, and other lipids.

Based on the method employed, publications were clustered into three main categories: (*i*) DNA/RNA detection and localisation techniques; (*ii*) DNA/RNA detection and localisation techniques and protein assays; (*iii*) protein assays. These were further sub‐classified according to the specific molecular or protein techniques used. Studies examining receptor occurrence *in vitro* employed a range of reporter gene assays, transfection and transactivation assays as well as immunostaining diagnostics. As information on method validation can provide important insights into the reliability of the evidence/data reported, details on method validation, such as the implementation and names of reference genes or proteins were also extracted.

### (7) Data visualisation and analysis

Data collected in the data extraction inventory were visually summarised using the Tableau Public 2023.1 software. The data were systematically categorised according to several criteria: the tissue where each hormone, receptor, or enzyme was identified; the methods and validation steps taken for their identification; the species in which they were identified; information on potential activity of the hormone, receptor or enzyme; and the observed effect of the hormone, receptor or enzyme in response to pharmaceutical interventions. The online database is an interactive, publicly available and fully searchable resource that can be accessed online *via* the links provided in Appendix S7.

### (8) Meta‐biases

As the variation in approach among studies excluded a formal meta‐analysis, statistical methods for detecting meta‐biases in the evidence report were not possible to implement. However, all changes made to the final protocol (Panagiotidis, 2022) are explicitly stated and described in Appendix S2. Of note is the inclusion of the thyroid signalling pathway in our discussion, although this was not present in the search strings (see Table S3.1 in Appendix S3). Specifically, key words for biomolecules involved in the thyroid hormone signalling pathway were not initially part of our search strategy, but the identification of these biomolecules in papers identified by our systematic searches created an opportunity to discuss their occurrence in molluscs. We note that the absence of relevant terms in our search strategy is likely to have led to omission of important information regarding the occurrence of the thyroid hormone signalling pathway in molluscs, and that this pathway could be the focus of future studies.

## III. RESULTS

### (1) Overall summary of inventory

**Fig. 1:**
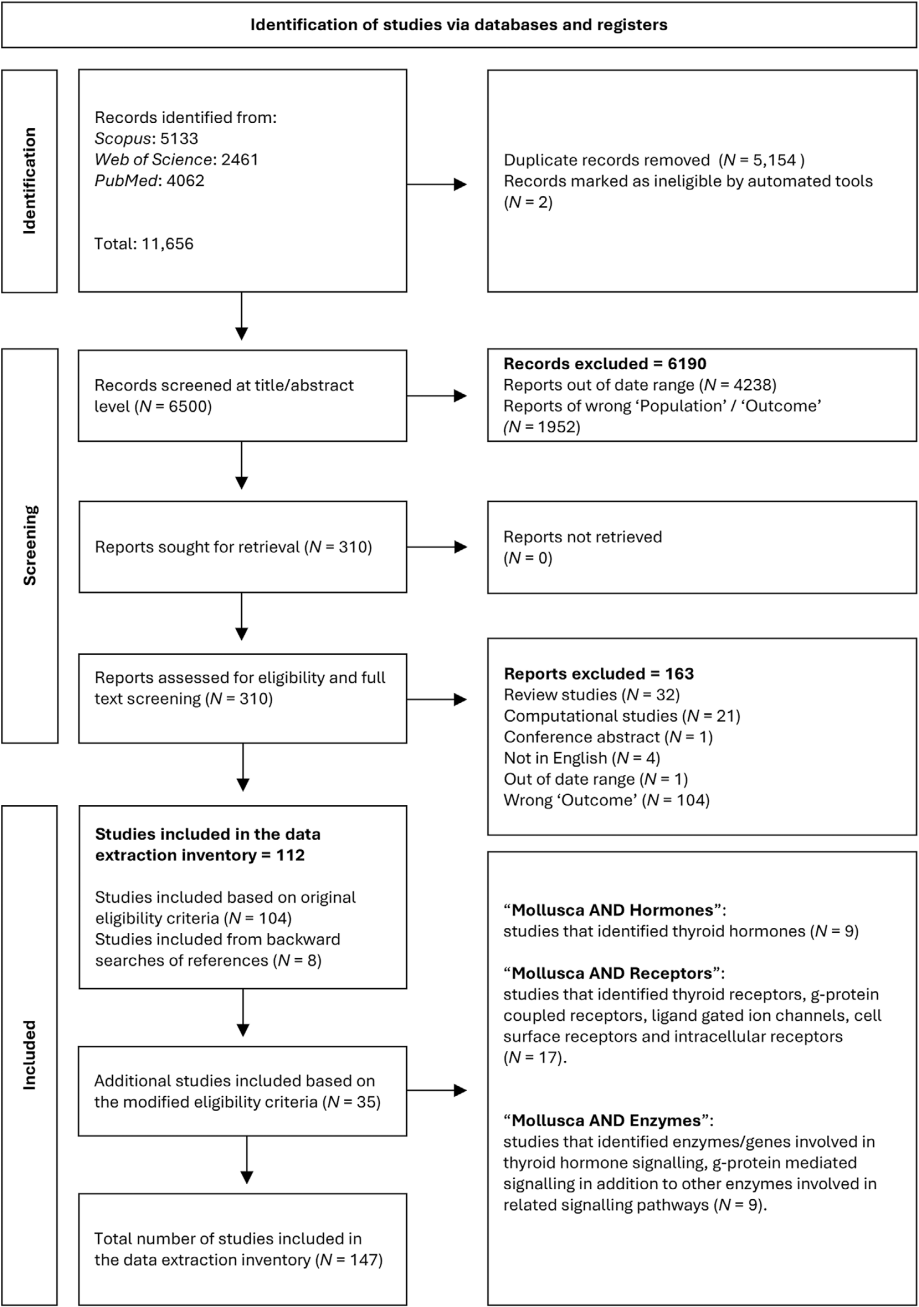
Study selection flow diagram, documenting the number of studies identified, removed, screened and included in our data extraction inventory (as per PRISMA 2015 guidelines; Shamseer *et al*., 2015).

The systematic literature searches identified 11,656 records from three databases (Fig. 1). Following duplicate removal, 6,500 records were screened at the title and abstract level for relevance. During this process and based on the initial eligibility criteria of the draft protocol (Panagiotidis, 2021), an additional 6,190 records were excluded. The remaining 310 records were screened at full‐text level to identify eligibility for inclusion in the data extraction inventory. During full‐text screening, 112 studies were found eligible for inclusion in the inventory based on the initial eligibility criteria (draft protocol). Additionally, 35 studies were included in the data‐extraction inventory based on the final modified eligibility criteria (Table S1.1 in Appendix S1). Thus, a total of 163 studies were excluded during full‐text screening, and 147 studies were included in the data‐extraction inventory.

The number of eligible studies retrieved by year of publication according to the PO statements can be found in Fig. S7.1 of Appendix S7.

### (2) Risk‐of‐bias of included studies

**Fig. 2:**
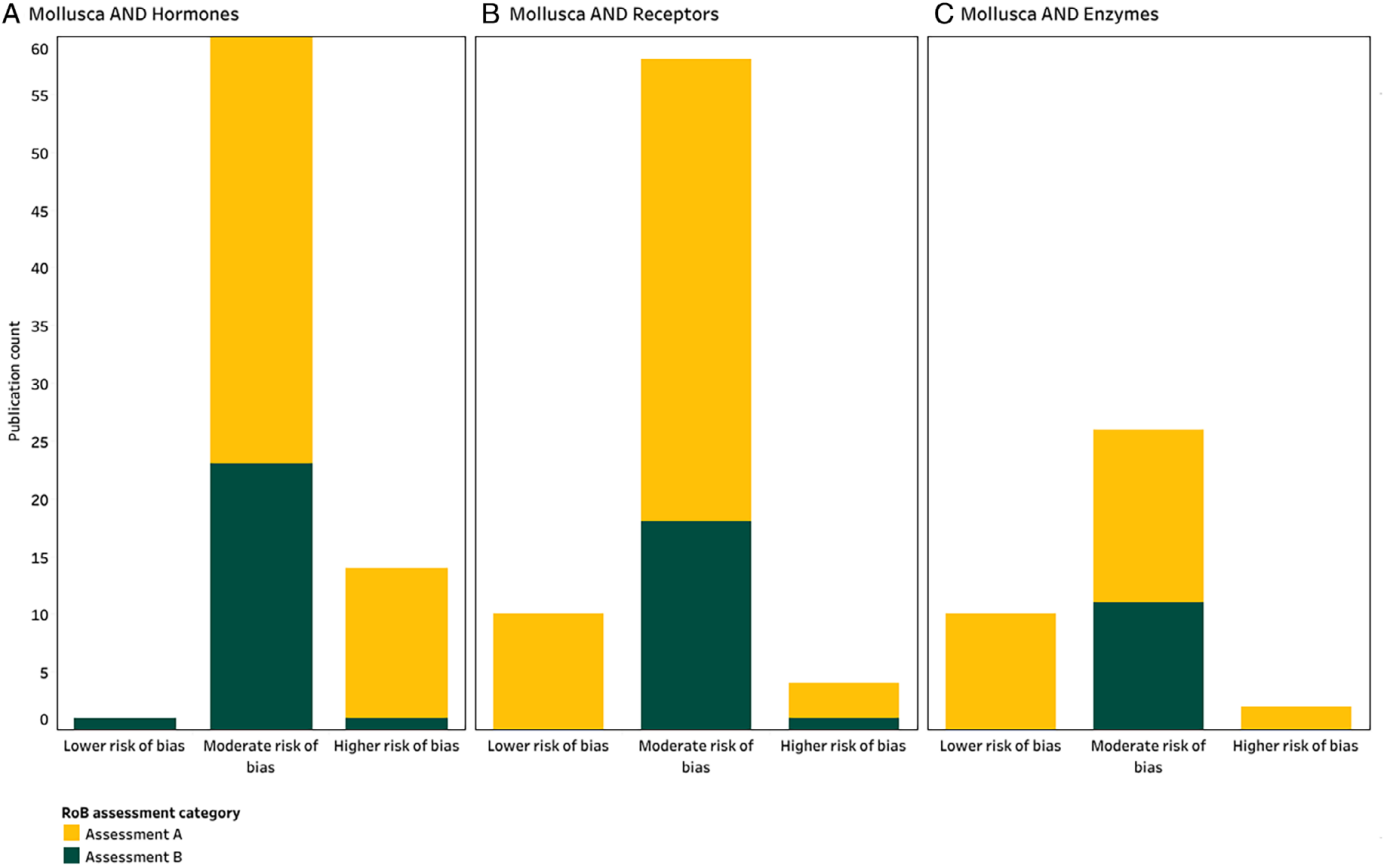
Results of the Risk‐of‐Bias (RoB) appraisal for included studies according to assessment category, for the (A) Mollusca AND Hormones, (B) Mollusca AND Receptors, and (C) Mollusca AND Enzymes data inventories. RoB assessments were carried out using the RoB tool and guidelines (see Appendix S5 and S6). The graph can be seen in interactive view *via* the links in Appendix S7. References used to create this figure are listed in Appendix S7, Sections 4.1–4.3, respectively.

Figure 2 details the number of extracted studies allocated to the different RoB categories for each PO statement. Independent of assessment category, most studies were rated as moderate RoB (Fig. 2). In the overwhelming majority of these studies, the moderate rating was due to issues related to study design. For example, 83% of studies in all three data inventories lacked independent repetition of experiments.

Many studies also had issues related to internal validity. For example, a significant factor that contributed to a rating of moderate and higher RoB in the Mollusca AND Hormones inventory (Fig. 2A), was the absence of ligand binding assays. Among the studies that focused on steroids, endogenous retinoids, protein hormones, or biogenic amines, 95% did not investigate the ability of these hormones to bind to their respective hormone receptors.

Moreover, 45% of studies that examined NRs from the Mollusca AND Receptors inventory were considered to have moderate or higher RoB due to lack of a sequence similarity analysis for the receptor's DNA binding domain (DBD) or ligand binding domain (LBD). DBD and LBD are characteristic regions of NR proteins, which recognise and bind to specific DNA fragments or receptor ligands, respectively (Vogeler *et al*., 2014). A sequence similarity analysis of DBD and LBD is of particular importance as it provides insights regarding NR functional ability, increasing the reliability of the reported outcomes. In studies from the Mollusca AND Enzymes inventory, phylogenetic analyses were not included in 48% of studies aiming to assess the function of hormone‐metabolising enzymes in molluscs. Full details of the RoB assessment criteria and scoring for studies included in the data extraction inventories are provided in Appendix S5.

One limitation of the RoB assessment is the potential oversimplification introduced by grouping the data into lower, medium and higher overall RoB scores. For example, during peer review, a considerable methodological flaw was identified in the study of Chong Sánchez *et al*. (2019). The authors measured sex hormones in the snail *Lobatus gigas* using high‐performance liquid chromatography (HPLC) with ultraviolet (UV) absorption. Amongst other reasons, this approach can be inaccurate due to the inability of UV detectors to differentiate compounds with overlapping spectra (reviewed by Scott, 2021). While this limitation was categorised as high RoB, the overall score of this study was rated as moderate RoB because of its lower scores in other aspects. As quantification errors, such as potentially present in Chong Sánchez *et al*. (2019), cannot be corrected for statistically, the overall moderate RoB score given may not adequately reflect the validity of a study. Moreover, it is likely that some confounding factors inevitably remained unaddressed, depending on the specific experimental design adopted by each study. While efforts were taken to ensure our RoB assessment reliably scored each study (through the use of two separate assessments based on the objectives of each study), developing a critical appraisal tool to account for every possible methodological limitation proved challenging. Therefore, it is recommended that the overall RoB scores for each publication should be considered in combination with the individual scores for each criterion listed in Appendix S6.

### (3) Mollusca AND hormones

#### (a) Inventory characteristics

**Fig. 3:**
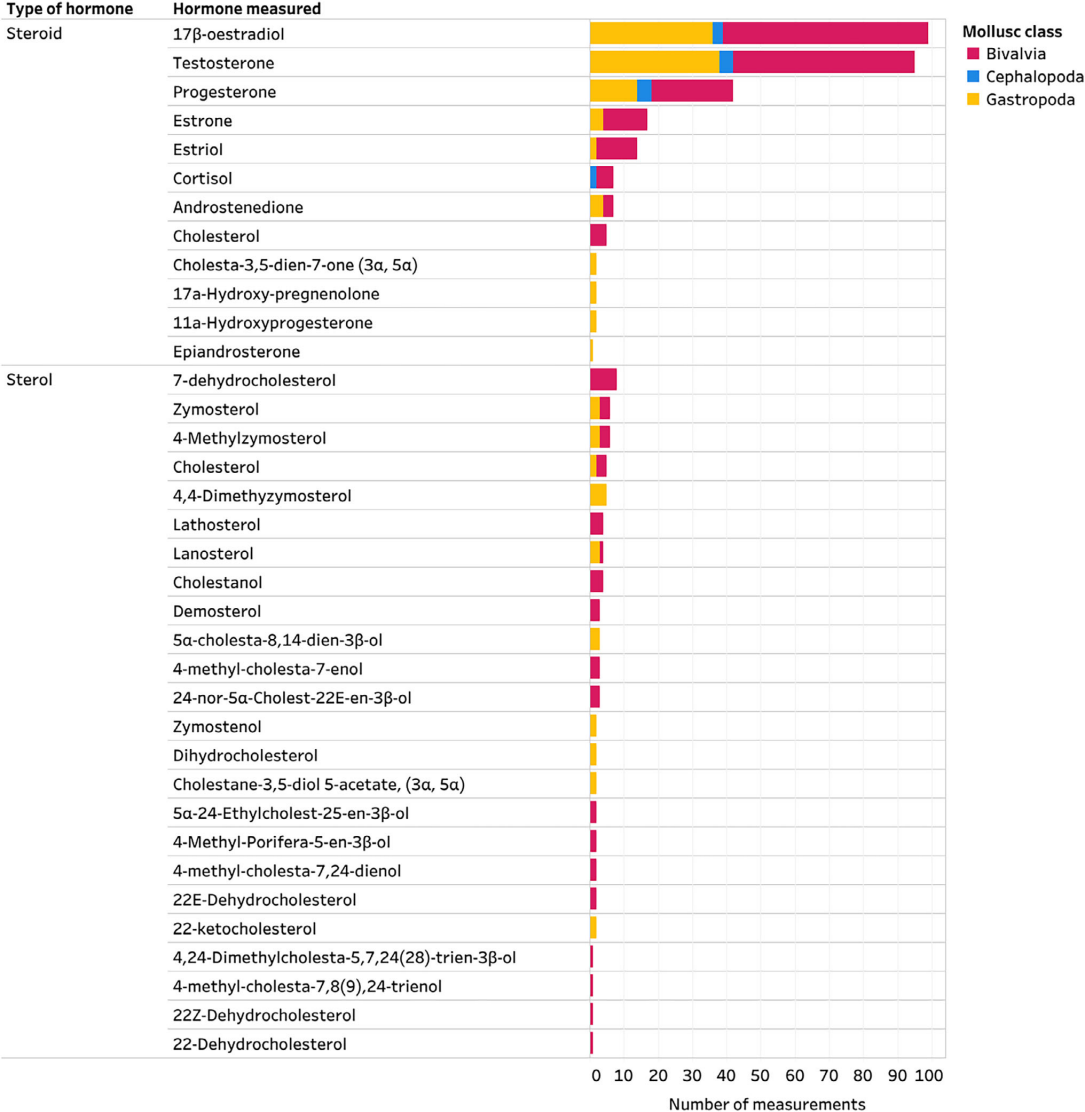
Steroids and sterols identified in Mollusca. Data on hormones is clustered according to hormone type and name. Number of measurements on the *x*‐axis indicates the total number of measurements of each hormone across species, sexes, life stages, tissues and methodological approaches. Therefore, multiple measurements can come from one publication, for example, two studies (Binder *et al*., 2019; Zhang *et al*., 2021) identified cortisol in Mollusca, with a total of 7 measurements (2 in cephalopods and 5 in bivalves). The Mollusca AND Hormones inventory, including details on all hormones identified, their reported activity, methodological details of included studies and the list of references can be seen in interactive view *via* the links in Appendix S7. References of studies included in this figure are listed in Appendix S7 (Section 3.1).

**Fig. 4:**
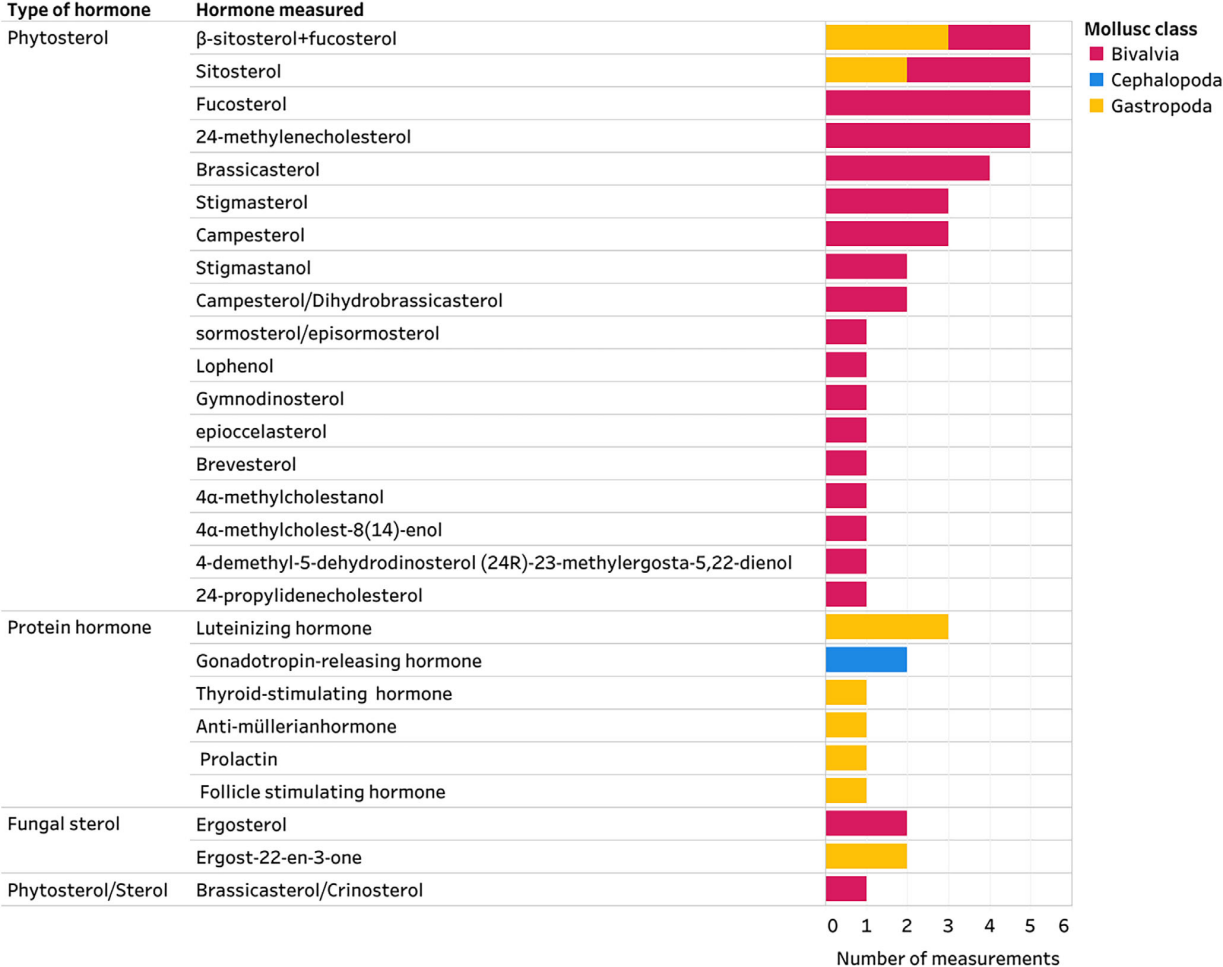
Phytosterols and protein hormones identified in Mollusca. Number of measurements on the *x*‐axis indicates the total number of measurements of each hormone across species, sexes, life stages, tissues, and methodological approaches. Therefore, multiple measurements can come from one publication, for example, two studies (Abd El‐Atti *et al*., 2020; Nuurai *et al*., 2020) identified luteinizing hormone in Mollusca, with a total of three measurements. The entire Mollusca AND Hormones inventory, including details on all hormones identified, their reported activity, methodological details of included studies and the list of references can be seen in interactive view *via* the links in Appendix S7. References included in this figure are listed in Appendix S7 (Section 3.2).

The Mollusca AND Hormones inventory was dominated by research on Bivalvia, Cephalopoda, and Gastropoda (Fig. 3). The steroid with the highest number of measurements was 17β‐oestradiol (66% of all studies), followed by testosterone and progesterone (Fig. 3). Eighty percent of studies in the Mollusca AND Hormones inventory measured at least one steroid. Sterols (e.g. 7‐dehydrocholesterol) were the second most frequently documented group, reported in 8% of included studies, while biogenic amines (e.g. thyroxine) and retinoids [e.g. 13‐cis‐retinoic acid (13‐cis‐RA)] were reported in 14% of studies. Less commonly recorded hormones, such as phytosterols (e.g. sitosterol), protein hormones (e.g. thyroid‐stimulating hormone) and fungal sterols (e.g. ergosterol), were found in 8%, 4% and 3% of studies, respectively (Fig. 4). Studies often report hormone levels for different sexes within a species or examine different chemical forms of the same hormone (e.g. esterified *versus* unesterified forms of testosterone) (see Appendix S4). These are combined as a total sum in the figures presented herein.

#### (b) Methodology characteristics for the Mollusca AND hormones inventory

**Fig. 5:**
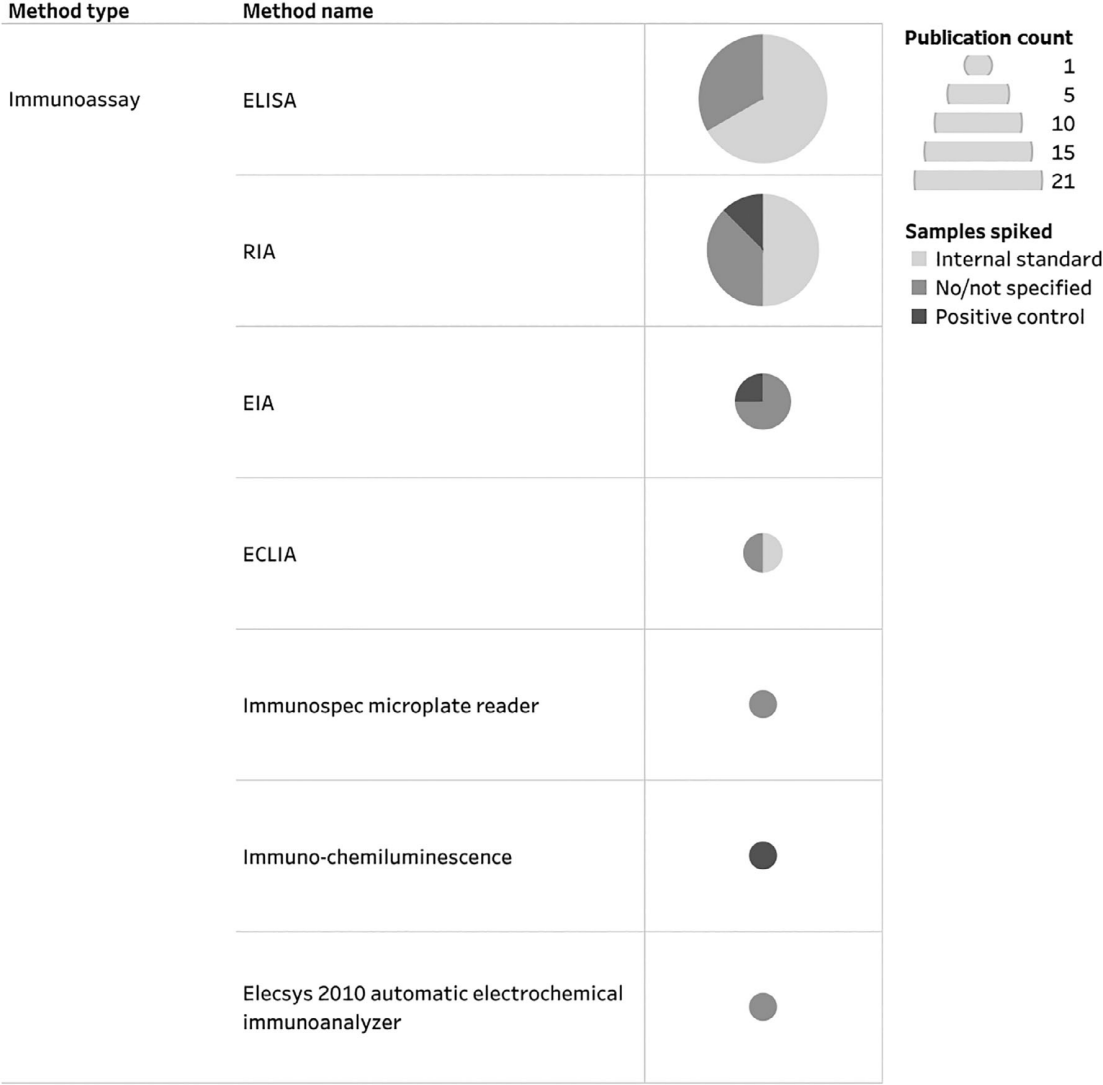
Methodological characteristics of studies that employed immunoassays to examine the presence of hormones in molluscs. Studies were classified based on the method employed and the inclusion of positive quality controls or internal standards in their assays. Abbreviations: ECLIA, electrochemiluminescence immunoassay; EIA, enzyme immunoassay; ELISA, enzyme‐linked immunosorbent assay; RIA, radioimmunoassay. The entire Mollusca AND Hormones inventory, including details on all hormones identified, their reported activity, methodological details of included studies and the list of references can be seen in interactive view *via* the links in Appendix S7. References included in this figure are listed in Appendix S7 (Section 3.3).

For studies included in the Mollusca AND hormones inventory, 62% used at least one type of immunoassay to measure hormones in molluscs (Fig. 5), but only 59% of these implemented the use of a positive quality control or internal standard. The most common immunoassay method used was ELISA (46% of immunoassay‐based studies), followed by RIA (35%) and enzyme immunoassay (EIA) (9%). In comparison, chemical analyses were adopted by 41% of studies in this inventory, out of which 83% included positive controls or internal standards as part of their method development. Within the studies employing chemical analysis to measure hormones in molluscs, 40% used HPLC, 20% used GC–MS and 27% used LC–MS.

### (4) Mollusca AND receptors

#### (a) Inventory characteristics

**Fig. 6:**
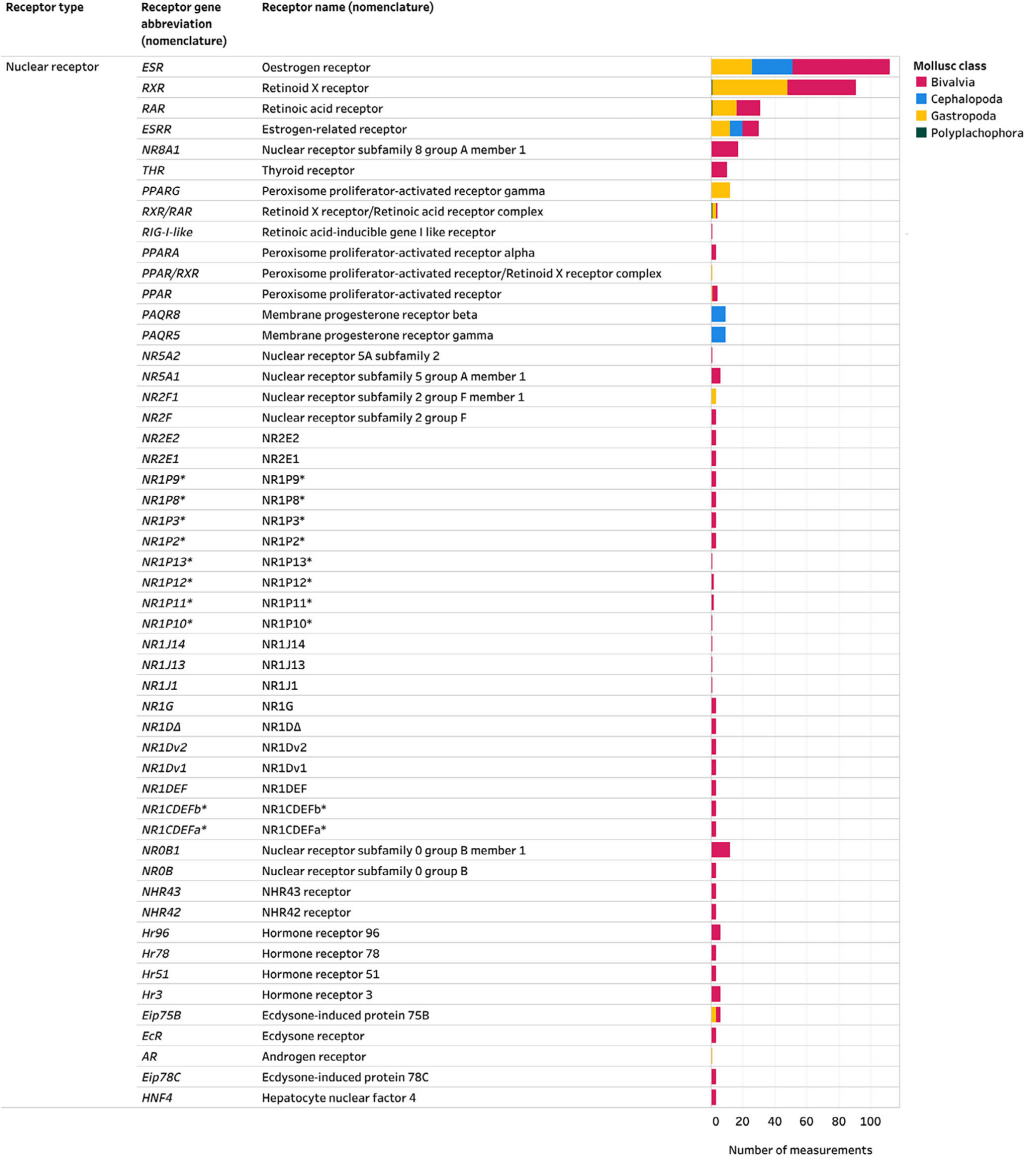
Nuclear receptors identified in molluscs. Number of measurements on the *x*‐axis indicates the total number of measurements across species, tissues, sexes, life stages, and methodological approaches. Therefore, multiple measurements can come from one publication. * indicates that an accepted nomenclature abbreviation or name for the reported gene could not be identified, and gene abbreviations are taken from the respective publications. The entire Mollusca AND Receptors inventory, including details on all receptors identified, their reported activity, methodological details of included studies and the list of references can be seen in interactive view *via* the links in Appendix S7. References included in this figure are listed in Appendix S7 (Section 3.4).

The receptor with the highest number of measurements in the Mollusca AND Receptors inventory (Fig. 6), was *ESR* (oestrogen receptor), followed by *RXR* (retinoid X receptor) and *RAR* (retinoic acid receptor). *ESR* was reported in Bivalvia, Cephalopoda and Gastropoda, while *RXR* and *RAR* were recorded in Bivalvia, Gastropoda and Polyplacophora (Fig. 6). Polyplacophora was the least studied molluscan class in the Mollusca AND Receptors inventory and was included in only one study (André *et al*., 2019).

Non‐vertebrate type receptors have been relatively understudied and we have a limited understanding of their roles in molluscs. Vertebrate‐type receptors were identified in 96% of included studies compared to only 13% of studies reporting non‐vertebrate‐type receptors. Some non‐vertebrate receptors seem to occur exclusively in molluscs, suggesting that distinct receptor signalling mechanisms may be present in this phylum. For example, the NR *NR8A1* found in tissues of the bivalve *Crassostrea gigas* (Huang *et al*., 2015a) seems to belong to a novel NR subfamily *NR8*. Although phylogenetic analyses have shown that *NR8* originated in eumetazoans (likely in a sister clade to sponges), this NR subfamily seems to have disappeared from both vertebrates and ecdysozoans (Huang *et al*., 2015a; Simion *et al*., 2017). Other non‐vertebrate receptors in molluscs reported in this inventory include *Eip78C* (ecdysone‐induced protein 78C) and *EcR* (ecdysone receptor) were identified in tissues of the bivalve *Crassostrea gigas* (Vogeler *et al*., 2016). Ecdysone is traditionally considered an arthropod hormone, and its presence in bivalve molluscs suggests a possible endocrinological pathway that needs further study.

Zhang *et al*. (2020) identified the presence of gonadotropin‐releasing hormone receptor (*GnRHR*) in the scallop *Patinopecten yessoensis*. Whole‐genome and transcriptome sequencing data have demonstrated the presence of GnRH‐type receptors and peptides that act as ligands for these receptors in molluscs (reviewed in Roch, Busby & Sherwood, 2011). However phylogenetic analyses of the evolutionary origins of GnRH signalling in molluscs revealed that they are paralogues of the corazonin (CRZ) signalling system, originating from a gene duplication in the common ancestor of bilaterians (Roch, Tello & Sherwood, 2014; Zandawala, Tian & Elphick, 2018). A duplication of the GnRH signalling system specifically in arthropods resulted in the adipokinetic hormone (AKH) and AKH/CRZ‐related peptide signalling systems (Hauser & Grimmelikhuijzen, 2014). Additional phylogenetic analyses in molluscs demonstrated that the *Octopus vulgaris GnRHR* is more closely related to arthropod *CRZ* receptors than the vertebrate *GnRHR* (Roch *et al*., 2014). Since studies have shown that CRZ and ‘AKH‐like’ signalling exists in molluscs (Li *et al*., 2016; Dubos, Bernay & Favrel, 2017; Fodor *et al*., 2024b), but true AKH signalling is specific only to arthropods, a change in nomenclature of these peptides and receptors was proposed. Thus, Zandawala *et al*. (2018) recommended that ‘GnRH‐like’ peptides and receptors identified in molluscs should be termed CRZ peptides or receptors, while peptides and receptors previously termed ‘AKH’ should now be classified as members of the GnRH superfamily. To ensure this is reflected in our Mollusca AND Receptors inventory, a note was added to the interactive figure for the molluscan ‘*GnRHR*’ nomenclature term (Appendix S7).

#### (b) Methodology characteristics for Mollusca AND receptors inventory

**Fig. 7:**
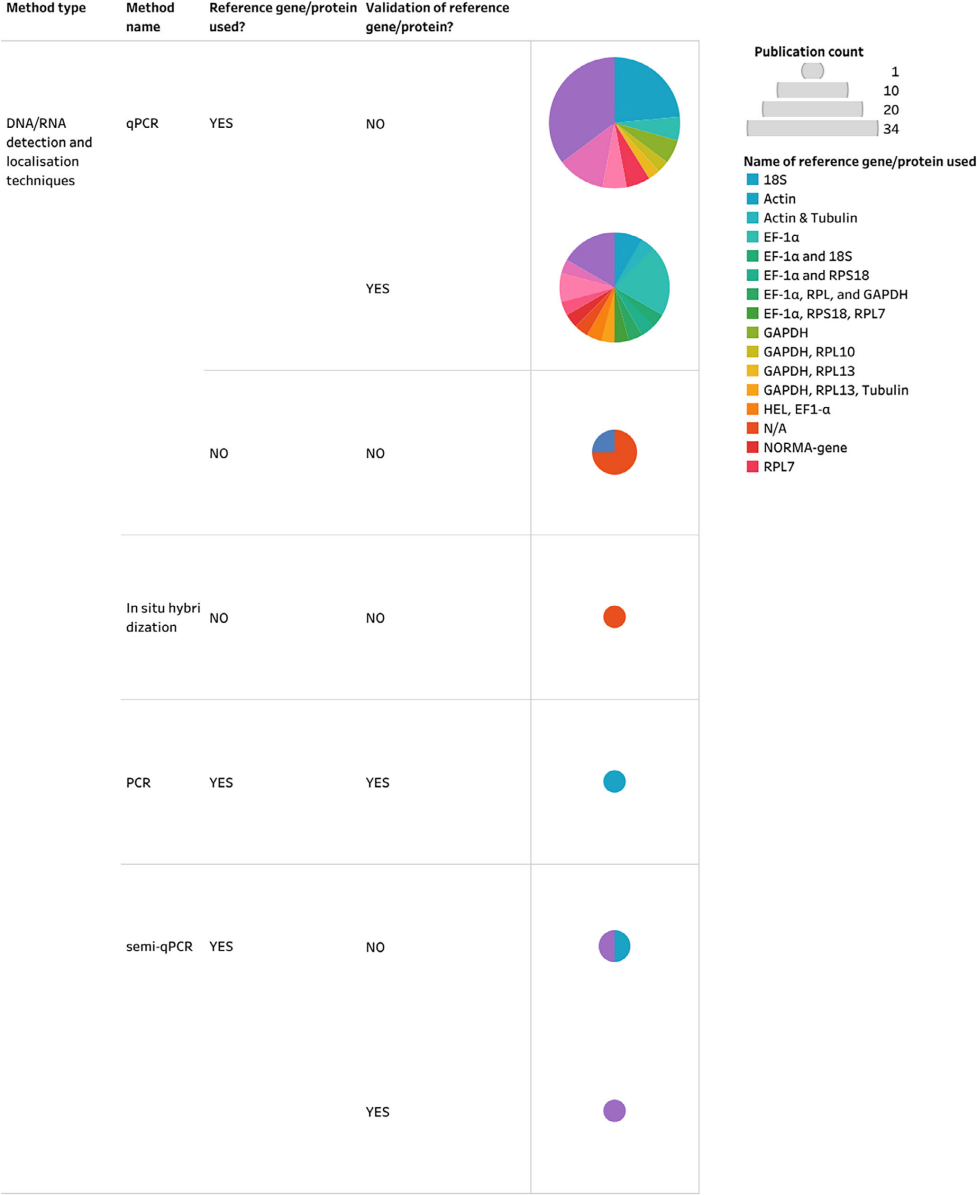
Methodological characteristics of studies that employed a DNA/RNA detection and localisation technique to measure receptor genes and proteins in molluscs. Studies are classified based on the type and name of method employed, the implementation of reference genes or proteins, as well as the experimental validation of those genes or proteins. Abbreviations: PCR, polymerase chain reaction; qPCR, quantitative PCR; semi‐qPCR, semi‐quantitative PCR. The entire Mollusca AND Receptors inventory, including details on all receptors identified, their reported activity, methodological details of included studies and the list of references can be seen in interactive view *via* the links in Appendix S7. References included in this figure are listed in Appendix S7 (Section 3.5).

The majority (85%, *N* = 61) of studies included in the Mollusca AND Receptor inventory utilised an DNA/RNA detection and localisation technique (Fig. 7). Among these studies, 78% used reference genes in their analyses. However, only 40% of studies that implemented reference genes validated their expression stability across experimental samples. Validation of expression stability in messenger RNA (mRNA) quantification assays is important as it ensures the suitability of reference genes (or proteins in protein quantification assays) for data normalisation and comparative expression (Bustin *et al*., 2009; Cowan *et al*., 2017). By contrast, studies that employed protein and other *in vitro* assays accounted for only 16% of the Mollusca AND Receptors inventory.

### (5) Mollusca AND enzymes

#### (a) Inventory characteristics

**Fig. 8:**
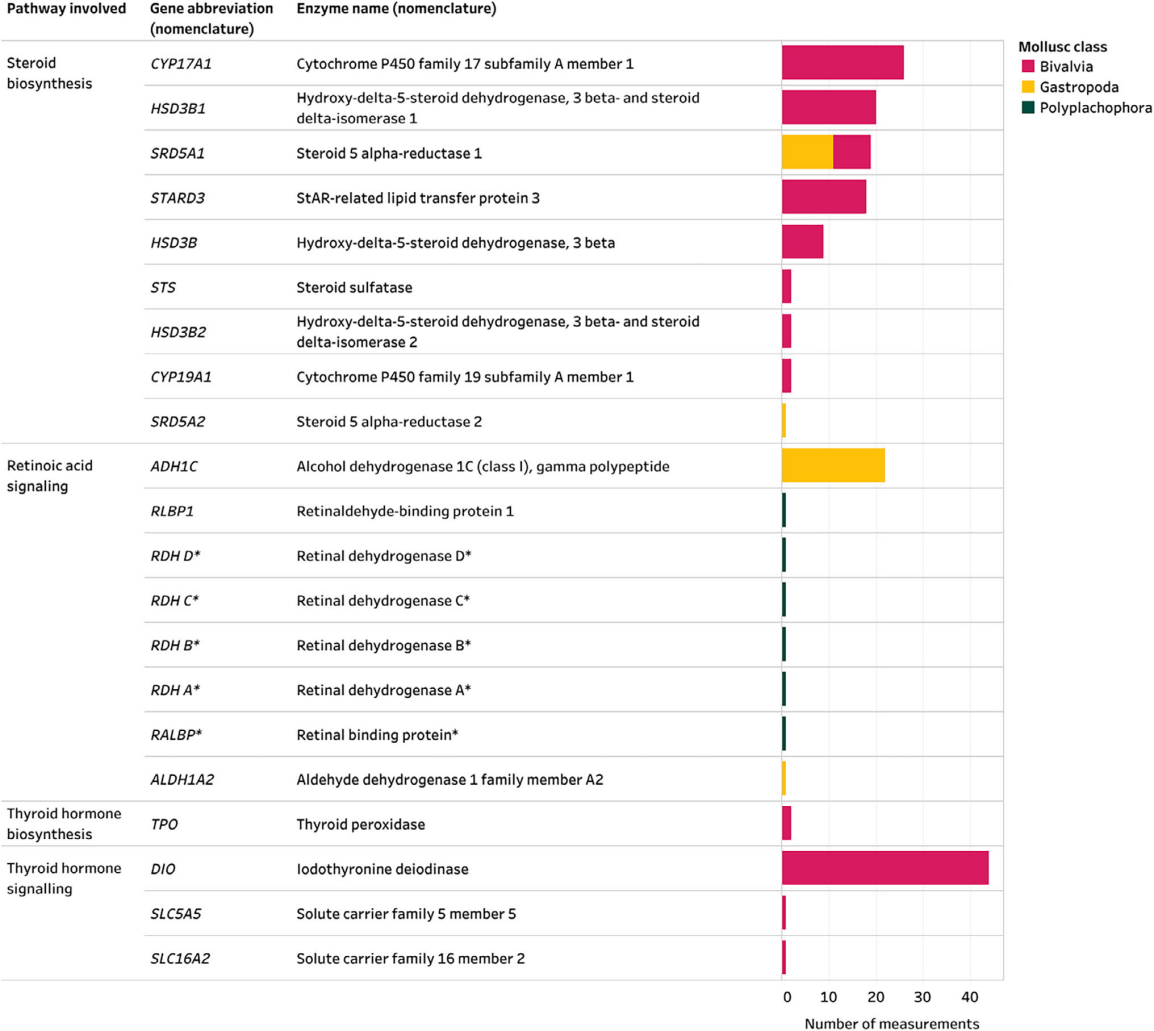
Enzymes involved in steroid biosynthesis, retinoic acid signalling, thyroid hormone signalling and thyroid hormone biosynthesis identified in Mollusca. Number of measurements on the *x*‐axis indicates the total number of measurements across species, tissues, life stages, and methodological approaches. Therefore, multiple measurements can come from one publication, for example three studies (Huang *et al*., 2015b; Jiang *et al*., 2019; Song *et al*., 2016) identified iodothyronine deiodinase in Mollusca. * indicates that a specific nomenclature abbreviation or name for the reported gene could not be identified, and the reported gene abbreviations are taken from the respective publications. The entire Mollusca AND Enzymes inventory, including details on all enzymes identified, their reported activity, methodological details of included studies and the list of references can be seen in interactive view *via* the links in Appendix S7. References included in the figure are listed in Appendix S7 (Section 3.6).

Some of the genes, enzymes or proteins identified in the Mollusca AND Enzymes inventory are involved in more than one metabolic or signalling pathway, such as *HSD17B8* (which encodes the enzyme 17β‐hydroxysteroid dehydrogenase type 8) which is known to participate in cholesterol biosynthesis as well as the metabolism of steroids and other lipids. Genes, enzymes and proteins involved in the vertebrate‐type steroid biosynthesis pathway were found in 41% of included studies (Fig. 8). *CYP17A1* which encodes the enzyme 17α‐hydroxylase, 17,20‐lyase, was the most studied biomolecule, found in 18% (*N* = 7) of studies. In humans, 17α‐hydroxylase/17,20 lyase adds a hydroxyl group to the 17‐carbon position of progesterone or pregnenolone and can further convert these 17‐OH products (*via* lyase activity) to androstenedione or dehydroepiandrosterone (DHEA), respectively. Therefore, in vertebrates, *CYP17A1* facilitates the production of steroid precursors of cortisol, oestrogen and testosterone. Based on the data collected, the steroidogenic genes *CYP17A1*, *HSD3B1*, *STARD3*, *HSD3B*, and *HSD3B2* have only been reported in bivalves. However, sequences of some of these genes (e.g. *STARD3*) in other mollusc classes have been deposited in databases (e.g. NCBI) not considered in this review, perhaps suggesting a reporting bias towards bivalves in the extracted publications. Steroid sulfatase (encoded by *STS*) and cytochrome P450 family 19 subfamily A member 1 (encoded by *CYP19A1*) were also reported in bivalves, however these proteins were identified by a western blot and an enzyme‐linked immunosorbent assay (ELISA) respectively, and the studies did not confirm that *STS* and *CYP19A1* genes are present in bivalve genomes. The steroidogenic genes *SRD5A1* and *SRD5A2* which encode the enzymes 5α‐reductase type 1 (5αR1) and 5α‐reductase type 2 (5αR2) respectively, were identified in gastropods and/or bivalves (Fig. 8). In this inventory Bivalvia were the most studied molluscan class (66% of studies), followed by Gastropoda (24%), while Cephalopoda and Polyplachophora were used in 8% and 5% of included studies, respectively.

In addition to several genes involved in retinoic acid and thyroid hormone signalling (discussed in Sections III.6.*c* and III.6.*e*), of note is the identification of molluscan homologues for the protein retinochrome in Polyplacophora (Vöcking, Leclère & Hausen, 2021). Retinochrome, which was first identified in cephalopods, plays an important role in the visual system of some marine molluscs and is known to interact with the vitamin A metabolite, retinal (Vöcking *et al*., 2021). To the best of our knowledge, retinochrome has not been identified outside molluscs. Although the role of retinochrome in retinoic acid signalling is currently not well understood, its identification in several molluscan classes hints at distinct functions of certain non‐vertebrate‐type proteins in these animals that are known to interact with retinoids.

#### (b) Methodology characteristics for Mollusca AND enzymes inventory

As outlined for receptors in Section III.4.*b*, the identification of mRNA transcripts corresponding to genes involved in metabolic and signalling pathways serves as robust evidence for the existence of their encoded proteins within an organism. However, hormone‐metabolising enzymes were identified in eligible studies by DNA/RNA detection and localisation techniques, as well as protein assays.

**Fig. 9:**
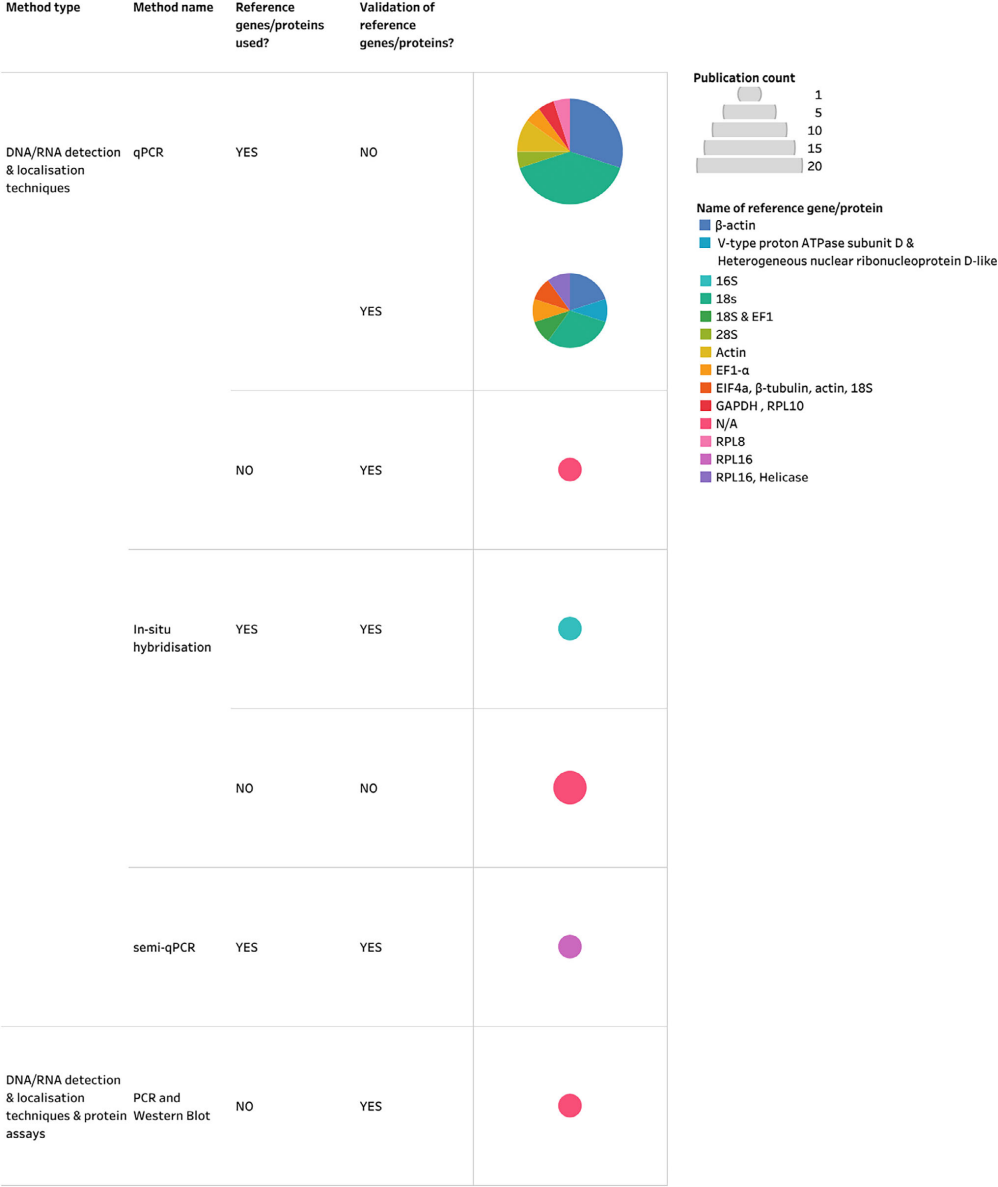
Methodological characteristics of studies that measured hormone‐metabolising enzymes using a DNA/RNA detection and localisation technique. Studies were classified based on the type and name of method employed, the implementation of reference genes or proteins, as well as the experimental validation of those genes or proteins. Abbreviations: qPCR, quantitative PCR; semi‐qPCR, semi‐quantitative PCR. The entire Mollusca AND Enzymes inventory, including details on all enzymes identified, their reported activity, methodological details of included studies and the list of references can be seen in interactive view *via* the links in Appendix S7. References included in this figure are listed in Appendix S7 (Section 3.7).

Of all studies included in this inventory, 89% (*N* = 34) employed a DNA/RNA detection and localisation technique to investigate the presence of hormone‐metabolising enzymes in molluscs (Fig. 9). Among these studies, 91% utilised at least one reference gene in their analyses to measure relative gene expression of relevant transcripts. However, only 35% of these studies attempted to validate the expression stability of the reference genes across experimental samples. Consequently, two thirds (65%) of studies from this inventory that utilised reference genes or proteins in their mRNA assays might have reported inaccurate results. Protein quantification assays were employed in 13% of studies included in the Mollusca AND Enzymes inventory, but none of these studies utilised reference proteins in their analyses which raises further concerns regarding the accuracy of the reported outcomes.

### (6) Comparative endocrinology across molluscs, vertebrates, and other invertebrates

Given the ongoing debate on the occurrence, and possible role, of vertebrate‐type steroids in molluscs (Fodor *et al*., 2020; Scott, 2012, 2013, 2018), a comprehensive comparison between vertebrate steroidogenesis and the evidence supporting the biosynthesis of these molecules in molluscs is required. Using the findings from our three data inventories, we present schematics of the vertebrate cholesterol synthesis pathway and steroidogenesis pathway outlining the evidence for the necessary genes and proteins in molluscs and highlighting gaps in our knowledge. A comparative assessment of the retinoic acid signalling pathway between molluscs and vertebrates is also conducted, while the presence of understudied hormonal pathways in molluscs (e.g. ecdysone biosynthesis, thyroid hormone synthesis) is also discussed.

#### (a) Evidence for cholesterol biosynthesis in molluscs

**Fig. 10:**
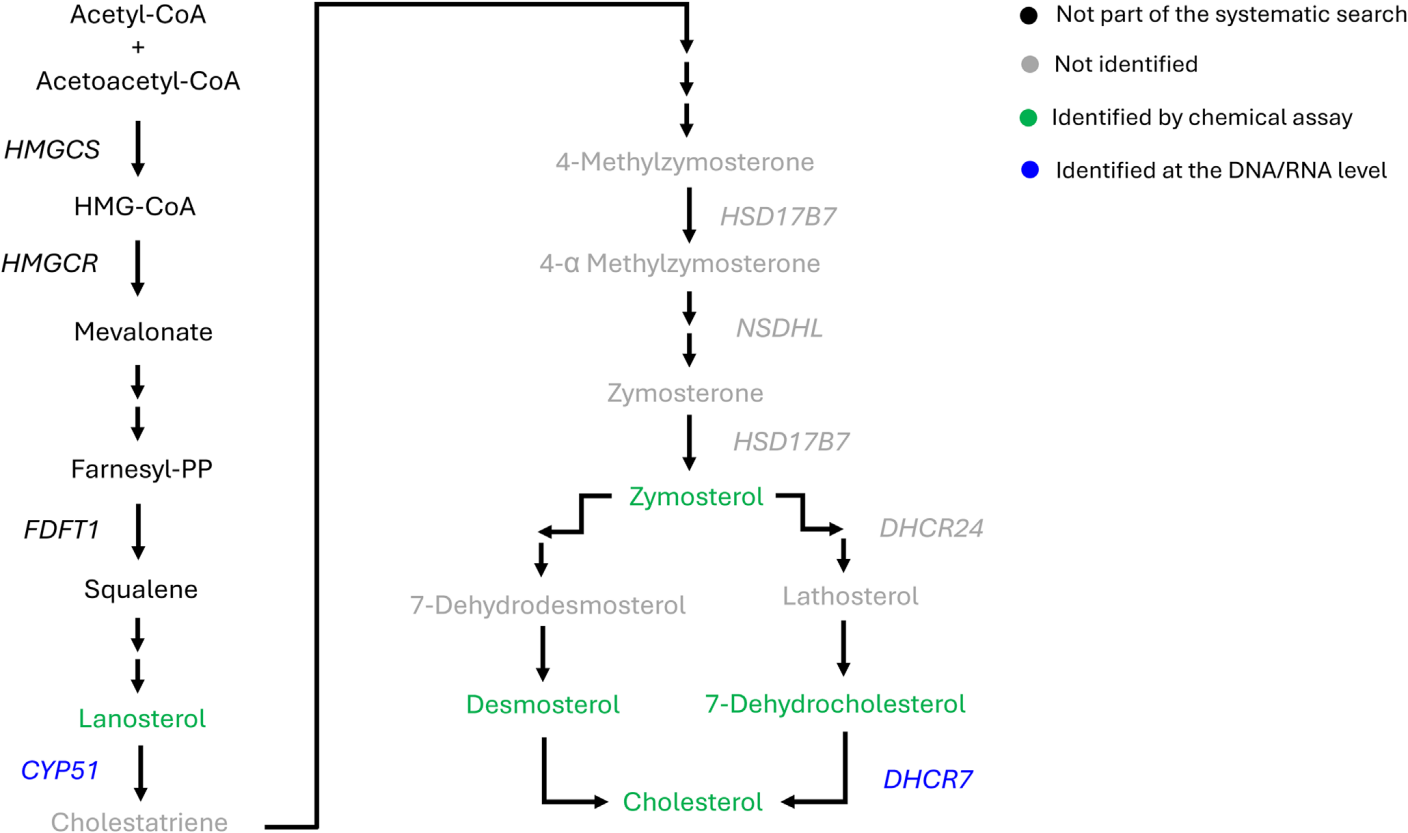
Schematic representation of the cholesterol biosynthesis pathway in vertebrates (adapted from Saloniemi *et al*., 2012) which includes the conversion of sterols (lanosterol, 4‐methylzymosterone, 4‐α methylzymosterone, zymosterone, zymosterol, 7‐dehydrodesmosterol, lathosterol, desmosterol, 7‐dehydrocholesterol, cholesterol) and sterol‐related molecules (acetyl‐CoA, acetoacetyl‐CoA, β‐Hydroxy β‐methylglutaryl‐CoA (HMG‐CoA), mevalonate, farnesyl‐PP, squalene, cholestratriene) by related enzymes (*HMGCS*, 3‐hydroxy‐3‐methylglutaryl‐CoA synthase; *FDFT1*, farnesyl‐diphosphate farnesyltransferase 1; *CYP51*, cytochrome P450 family 51; *HSD17B7*, hydroxysteroid 17‐beta dehydrogenase 7; *NSDHL*, NAD(P)‐dependent steroid dehydrogenase‐like; *DHCR24*, 24‐dehydrocholesterol reductase; *DHCR7*, 7‐dehydrocholesterol reductase). Biomolecules included in our three data inventories are highlighted by colour according to the method used for their identification: black (biomolecules not part of the search strategy); grey (biomolecules that were part of the search strategy but were not identified); green (biomolecules identified in molluscs by a chemical assay); blue (biomolecules identified in molluscs at the DNA/RNA level). References included in this figure are listed in Appendix S7 (Section 3.8).

Cholesterol is a commonly occurring steroid and serves as a precursor for the synthesis of seven classes of steroids in vertebrates (oestrogens, androgens, progestins, glucocorticoids, mineralocorticoids, vitamin D steroids and bile acids) (Norman & Litwack, 1997b). The cholesterol biosynthesis pathway is outlined in Fig. 10 (Saloniemi *et al*., 2012). Only a few of the biomolecules known to be involved in cholesterol biosynthesis were identified for molluscs from our systematic searches (Fig. 10). Among these, the sterol enzyme 14‐demethylase is encoded by the *CYP51* gene and metabolises lanosterol to cholestatriene. Lanosterol was identified in the ovaries, testis and gills of several gastropod species (Kawashima, Ohnishi & Ogawa, 2013; Takishita *et al*., 2017) while *CYP51* was identified in the gill epithelial bacteriocytes of the bivalve *Bathymodiolus platifrons* (Takishita *et al*., 2017). However, neither the activity of *CYP51* nor the identification of the metabolite cholestatriene was recorded in any of our data inventories (Fig. 10). Additionally, several other biomolecules of the cholesterol biosynthesis pathway, including the steroidogenic gene *HSD17B7* which is essential for the conversion of lanosterol to zymosterol, were not located by our systematic searches (Fig. 10). Although the identification of 7‐dehydrocholesterol and its metabolising gene *DHCR7* have been identified in the gills of *B. platifrons* (Takishita *et al*., 2017), functional studies for *DHCR7* have not been reported in any study included the Mollusca AND Enzymes inventory. Currently, the availability of recent evidence in support of a cholesterol biosynthesis pathway in Mollusca is sparse. Some older studies (Voogt, 1967, 1967), and more recently reviewed by Scott (2012), suggested that cholesterol biosynthesis in molluscs may depend on whether the animals are herbivores or carnivores. However, further research and robust species‐specific evidence is required to support this hypothesis.

#### (b) Parallels of vertebrate steroidogenesis identified in mollusc tissues

**Fig. 11:**
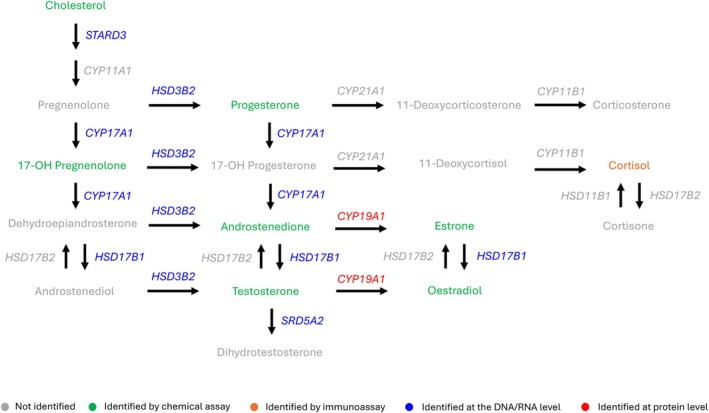
Schematic representation of the steroid biosynthesis pathway in vertebrates (adapted from Chakraborty *et al*., 2021) which includes the conversion of steroids (cholesterol, pregnenolone, 17‐OH pregnenolone, dehydroepiandrosterone, androstenediol, progesterone, 17‐OH progesterone, androstenedione, testosterone, dihydrotestosterone, 11‐deoxycorticosterone, 11‐deoxycortisol, estrone, oestradiol (17β‐oestradiol), corticosterone, cortisol, cortisone) by their respective enzymes (*STARD3*, StAR‐related lipid transfer domain containing 3; *CYP11A1*, cytochrome P450 family 11 subfamily A member 1; *CYP17A1*, cytochrome P450 family 17 subfamily A member 1; *HSD17B1*, hydroxysteroid 17‐beta dehydrogenase 1; *HSD3B2*, hydroxy‐delta‐5‐steroid dehydrogenase, 3 beta‐ and steroid delta‐isomerase 2; *CYP17A1*, cytochrome P450 family 17 subfamily A member 1; *SRD5A2*, steroid 5 alpha‐reductase 2; *CYP21A1*, cytochrome P450 family 21 subfamily A member 1; *CYP19A1*, cytochrome P450 family 19 subfamily A member 1; *HSD17B2*, hydroxysteroid 17‐beta dehydrogenase 2; *CYP11B1*, cytochrome P450 family 11 subfamily B member 1; *HSD11B1*, hydroxysteroid 11‐beta dehydrogenase 1). Biomolecules included in the three data inventories are highlighted by colour according to the method used for their identification: grey (biomolecules that were part of the search but were not identified); green (biomolecules identified in molluscs by a chemical assay); orange (biomolecules identified in molluscs by an immunoassay); blue (biomolecules identified in molluscs at the DNA/RNA level); red (biomolecules identified in molluscs at the protein level). The level of robustness is considered higher for biomolecules identified using chemical assays (green) or at the DNA/RNA level (blue). References included in this figure are listed in Appendix S7 (Section 3.9).

**Fig. 12:**
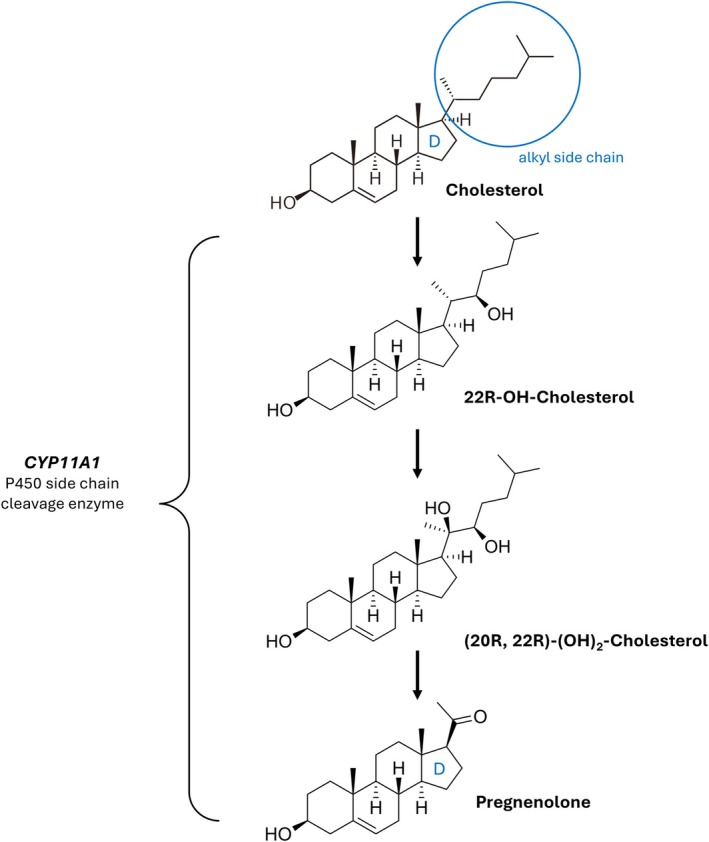
Metabolic activity of *CYP11A1* (P450 side chain cleavage enzyme) during vertebrate steroidogenesis, which includes the 22R‐hydroxylation and 20R‐hydroxylation of cholesterol and its eventual conversion to pregnenolone (*via* the removal of the alkyl side chain from cholesterol's D carbon ring). Figure adapted from Norman & Litwack (1997b).

The vertebrate steroidogenesis pathway begins with the metabolism of cholesterol to pregnenolone by the P450 cholesterol side chain cleavage enzyme (encoded by *CYP11A1*) (Figs 11 and 12). Pregnenolone can be converted into a range of steroids, including testosterone, 17β‐oestradiol, cortisol and progesterone by a series of metabolic reactions (Fig. 11).

##### (i) Cortisol pathway

In vertebrate steroidogenesis, pregnenolone is converted to progesterone which in turn is catalysed to corticosterone or cortisol *via* a series of metabolic reactions, mediated by the enzymes 21‐hydroxylase and 11β‐hydroxylase, which are encoded by *CYP21A1* and *CYP11B1*, respectively (Fig. 11). Although cortisol has been reported to be present in molluscan tissues *via* immunoassays (Binder *et al*., 2019; Zhang *et al*., 2021), *CYP21A1* and *CYP11B1* were not reported in any of the studies included in the Mollusca AND Enzymes inventory (Fig. 11). The absence of molluscan *CYP11B1* is also supported by phylogenetic analyses that highlight the lack of *CYP11* enzymes in molluscan genomes (Nelson, Goldstone & Stegeman, 2013). Therefore, the only evidence in the inventory for *de novo* synthesis of cortisol or corticosterone in molluscs relies on the detection of cortisol in tissues *via* immunoassays, which have known limitations in terms of accuracy and specificity (Gust *et al*., 2010), implying this pathway is not conserved within invertebrates. Interestingly, a recent study demonstrated the inability of the common octopus (*Octopus vulgaris*) to produce cortisol or corticosterone in response to stress (Maskrey *et al*., 2025), supporting the suggestion that molluscs have different endocrine systems compared to vertebrates.

##### (ii) Sex steroid pathway

In vertebrates, androgens are synthesised *via* two main metabolic pathways, namely Δ^4^ and Δ^5^, both of which are derived from pregnenolone (Norman & Litwack, 1997b). Both pathways involve the 17α‐hydroxylation of pregnenolone or progesterone via 17α‐hydroxylase/17,20 lyase (*CYP17A1*) and the conversion of either dehydroepiandrosterone (DHEA) or androstenedione by 17β‐hydroxysteroid dehydrogenase (*HSD17B1*) to androstenediol or testosterone, respectively. Androstenediol can be converted to testosterone by 3β‐steroid dehydrogenase (*HSD3B2*), which is in turn metabolised to the more potent androgen, dihydrotestosterone, by 5α‐reductase (*SRD5A2*). Estrone and oestradiol (i.e. 17β‐estradiol), are products of androstenedione and testosterone, respectively, catalysed by the enzyme aromatase, encoded by the *CYP19A1* gene (Fig. 11). According to our inventory, a range of steroids (e.g. progesterone, testosterone, 17β‐estradiol) in these pathways have been quantified in molluscan tissues *via* analytical methods (Fig. 11), and homologs of many (but not all) of the key steroidogenic enzyme genes have been identified in molluscs (e.g. *CYP17A1*, *HSD3B2*, *HSD17B1*, *SRD5A2*). According to our systematic searches, *CYP19A1* has not been identified in molluscs by a DNA/RNA detection or localisation technique (Fig. 11) or in the genomes of molluscs (reviewed by Fodor *et al*., 2020; Scott, 2012). Aromatase (*CYP19A1*) has been reported in molluscan tissues using vertebrate antibodies (Prisco *et al*., 2017; Rosati *et al*., 2019a). However, evidence based on vertebrate antibodies in molluscs needs to be considered with caution, as this methodology can be highly inaccurate for detecting proteins in invertebrates, as extensively reviewed by Fodor *et al*. (2022). This emphasises the necessity for changing how we identify enzymes in molluscs. If we are to ensure reliability in the reported outcomes, future investigations should prioritise the utilisation of molluscan antibodies in protein quantification assays. These analyses should be further accompanied by genomic investigations of the mRNA transcripts that encode those proteins.

#### (c) Evidence on retinoic acid signalling in molluscs

**Fig. 13:**
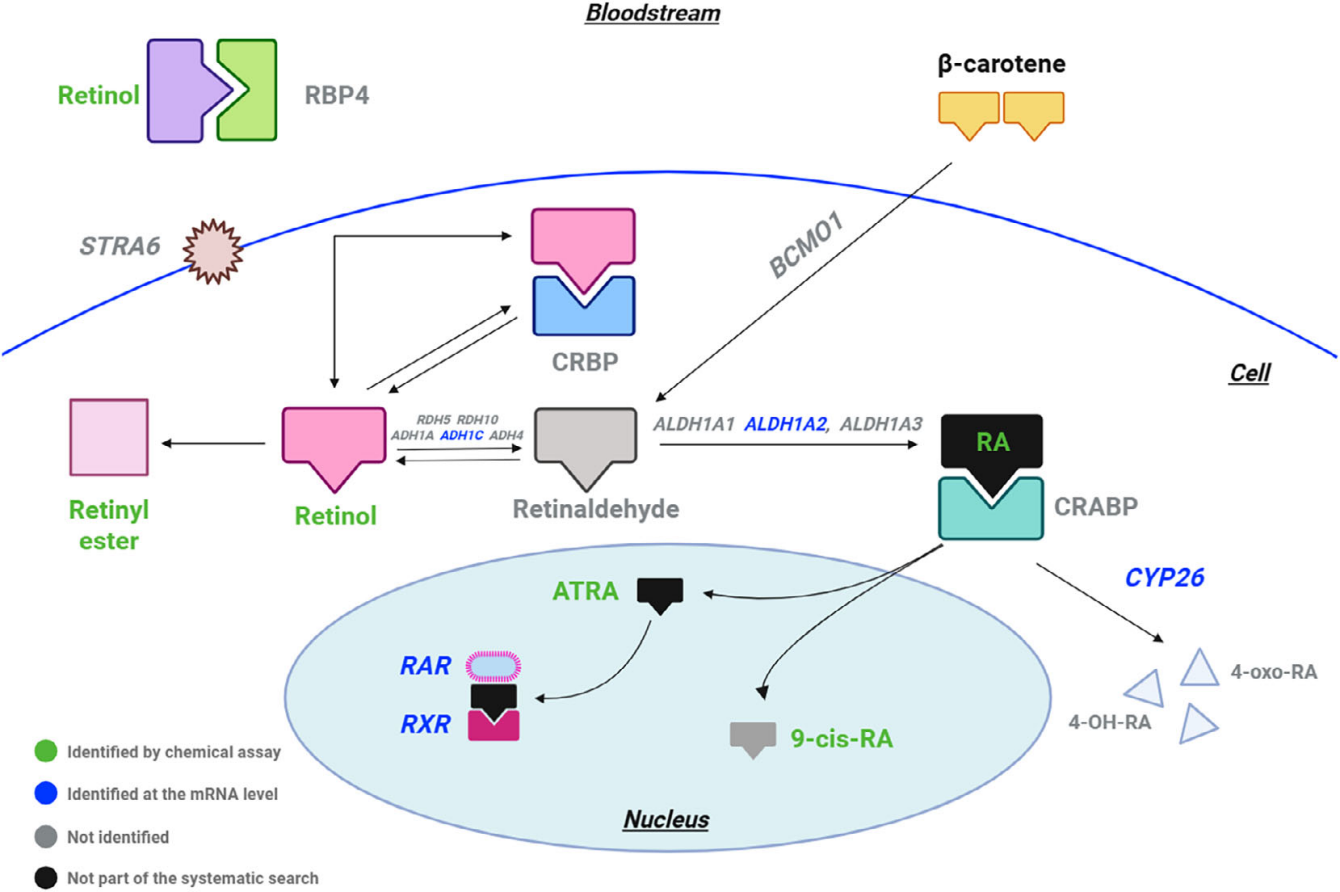
The retinoic acid signalling pathway in vertebrates, including the enzymes and nuclear receptors involved in retinoid metabolism and retinoid transfer. Biomolecules included in the systematic searches are highlighted by text colour according to the method used for their identification: black (biomolecules not part of the search); grey (biomolecules that were part of the search but not identified); green (biomolecules identified by a chemical assay); blue (biomolecules identified at the DNA/RNA level). Abbreviations: CRABP, cellular retinoic acid‐binding protein; CRBP, cellular retinol‐binding protein; ATRA, all‐trans retinoic acid; 9‐cis‐RA, 9‐cis retinoic acid; 4‐oxo‐RA, 4‐oxo‐retinoic acid; RA, retinoic acid; RPB4, retinol‐binding protein 4. Figure created with BioRender.com. References included in this figure are listed in Appendix S7 (Section 3.10).

Retinoic acid (RA) is a metabolic derivative of retinol (vitamin A). Isomers of RA, such as 9‐cis‐RA, all‐trans‐RA and 13‐cis‐RA, exhibit distinct biological activities and play important roles in different processes (Ghyselinck & Duester, 2019). In vertebrates, RA synthesis begins with the conversion of retinol to retinaldehyde by two types of enzymes: alcohol dehydrogenases (*ADH1A*, *ADH1C*, and *ADH4*) or retinol dehydrogenase (*RDH5* and *RDH10*) (Kumar *et al*., 2012). Retinaldehyde is then metabolised to RA with the help of the enzymes retinaldehyde dehydrogenase 1, 2 and 3 (RALDH1, RALDH2 and RALDH3), encoded by the *ALDH1A1*, *ALDH1A2*, and *ALDH1A3* genes respectively (Fig. 13). Interestingly, some vertebrates, like zebrafish (*Danio rerio*), are known to have lost their *ALDH1A1* ortholog and thus utilise only *ALDH1A2* and *ALDH1A3* during RA synthesis (Cañestro *et al*., 2009). Eventually, the metabolism of retinaldehyde to RA leads to the degradation of RA by cytochrome P450 family 26 enzymes (CYP26A1, CYP26B1, CYP26C1), encoded by the *CYP26* genes (*CYP26A1*, *CYP26B1*, *CYP26C1*), which convert it to inactive metabolites (Ghyselinck & Duester, 2019). Once metabolised, RA isomers act as ligands for nuclear retinoic acid receptors (RARs). The main RA that binds to RARs is all‐trans‐RA (ATRA), although other isomers with lesser affinity, such as 9‐cis‐RA can also bind to RARs (Kumar *et al*., 2012). Once bound to a ligand, RARs are known to form heterodimers with retinoid X receptors (RXRs), which in contrast to RARs, can only bind to 9‐cis‐RA. Once formed, the RAR/RXR complex can modulate gene transcription by binding to specific DNA sequences known as RA response elements (RARE) (Ghyselinck & Duester, 2019).

Many of the biomolecules involved in the RA signalling pathway have also been reported to be present in molluscs (Fig. 13). Our systematic searches identified retinol, RA and RA isomers in various molluscan species and tissues (Fig. 13). Consequently, the presence of *ADH1C* and *ALDH1A2* orthologs in molluscan genomes (Coelho *et al*., 2012; Rothwell *et al*., 2014) provides evidence for the potential conservation of retinoid acid signalling in Mollusca. Indeed, exposure of gastropod *Nucella lapillus* females to retinol was shown to downregulate *ADH1C* expression levels in the gonads (Coelho *et al*., 2012). However, whether *ADH1C* and *ALDH1A2* can metabolise their respective retinoids in molluscs remains to be elucidated. Nonetheless, there is evidence that some retinoid receptors found in molluscs exhibit comparable functions to those found in vertebrates. In particular, RXR in molluscs was shown to bind to the RA isomer 9‐cis‐RA *in vitro* (Gutierrez‐Mazariegos *et al*., 2014). Conversely, *in vitro* exposure of molluscs to retinoids and retinoic acid isomers including retinal, retinol, ATRA, 9‐cis‐RA and 13‐cis‐RA, demonstrated an inability of molluscan RARs to bind to, and thus be activated by, their respective ligands. However, it was shown that RAR can form a heterodimer complex with RXR in molluscs (Gutierrez‐Mazariegos *et al*., 2014; Urushitani *et al*., 2013). The evidence suggests that some similarities exist between the vertebrate and molluscan RA signalling pathway. However, there are also differences in the regulatory mechanisms of retinoid receptors between these two groups, implying that the RA signalling pathway of molluscs and vertebrates has followed separate evolutionary paths (Gutierrez‐Mazariegos *et al*., 2014).

#### (d) The occurrence of phytosterols and edcysteroids in molluscs

Interestingly, our systematic searches identified the presence of a wide range of phytosterols (plant sterols) in molluscan tissues. Until recently, phytosterols were thought to occur exclusively in plants and that phytosterols were only able to enter an animal's body through its diet (Özyurt *et al*., 2013). However, a recent study has demonstrated that some marine annelids can synthesise sitosterol (a plant sterol) as well as cholesterol *de novo* (Michellod *et al*., 2023). Plant‐feeding insects were previously shown to metabolise phytosterols and convert them into cholesterol (Ikekawa, Morisaki & Fujimoto, 1993). This conversion involves the breakdown of alkyl groups that are attached to the 24th carbon atom of the phytosterol molecule (Ikekawa *et al*., 1993). Surprisingly, bivalve molluscs were found to possess similar metabolic abilities. The northern bay scallop *Argopecten irradians irradians* was shown to metabolise the radiolabelled phytosterols 24‐methylenecholesterol, 24‐propylidenecholesterol, epioccelasterol, brassicasterol, 4α‐methylcholestanol and 4α‐methylcholest‐8(14)‐enol to cholesterol (Giner *et al*., 2016). An interesting observation was the ability of this species to produce Δ^5,7^ sterols, such as provitamin D, from Δ^5^ (phyto)sterols (Giner *et al*., 2016). The induction of a double bond at the Δ^7^ position of the sterol molecule is the reverse mechanism from the one that exists in vertebrate sterol biosynthesis. This ability seems to be conserved in nematodes and insects, where the induction of a double bond at the Δ^7^ position of a sterol molecule induces the biosynthesis of the insect steroid ecdysone (Chitwood, Lusby & Salt, 1987; Huang, Warren & Gilbert, 2008). Notably, a recent phylogenetic analysis suggests that molluscs do not contain the 24‐C sterol methyltransferase (*SMT*) gene in their genomes, which is vital for the synthesis of phytosterols (Brunoir *et al*., 2023). Together, this evidence suggests that molluscs may take up Δ^5^ (phyto)sterols from the environment and convert them to Δ^5,7^ sterols for subsequent steroid biosynthesis. These observations imply that insect steroid hormones, like ecdysone, may be produced in molluscs endogenously.

Although the identification of insect steroids in molluscs was part of our search strategy, 7‐dehydrocholesterol was the only ecdysteroid intermediate reported to exist in molluscs in the Mollusca AND Hormones inventory (Hurtado *et al*., 2012; Takishita *et al*., 2017). Nevertheless, a growing body of evidence has identified many types of receptors in molluscs that were previously thought to exist exclusively in insects (Pes *et al*., 2021; Raingeard *et al*., 2013; Stange & Oehlmann, 2012; Vogeler *et al*., 2016). Among these, gene transcripts of the ecdysone receptor (*EcR*), which binds to and is activated by ecdysone in insects, were found to be expressed in the embryo and whole body of the bivalve *Crassostrea gigas* (Vogeler *et al*., 2016). The relative expression of *EcR* transcripts was found to vary across developmental stages and was particularly up‐regulated 15 days post‐fertilisation (Vogeler *et al*., 2016). A recent study has identified the expression of both membrane and nuclear *EcR* homologs in the genome and neural transcriptome of *Lymnaea stagnalis* (Fodor *et al*., 2024a) whereas another study measured ecdysone concentrations and the relative expression of *EcR* transcripts in tissues and larval developmental stages of the oyster *Pinctada fucata martensii* (Xiong *et al*., 2022). *EcR* was found to be most highly expressed at the gastrula stage of *P. f. martensii* larvae and in the mantle tissue of adults. Most importantly, a shell‐notching experiment in the same study revealed increasing ecdysone serum production from 2 to 8 h post‐shell damage which coincided with increasing relative expression levels of *EcR* (Xiong *et al*., 2022). Such findings suggest that molluscs, or at least bivalves, may produce ecdysone endogenously, although care must be taken with these initial studies as ecdysone was measured *via* an insect 20‐hydroxyecdysone ELISA Kit (Xiong *et al*., 2022). Another hypothesis is that molluscs absorb ecdysone from their diet rather than synthesising it endogenously (Garcia, Griffond & Lafont, 1995), thus leading to its interaction with a functional *EcR* in these animals. Notably, exogenous ecdysone has been demonstrated to interact with an endogenous G protein–coupled receptor–type (GPCR‐type) receptors in mammals, *via in vitro* and *in silico* approaches (Lafont *et al*., 2022). However, it remains unclear whether exogenous ecdysone can activate molluscan *EcR*. Moreover, as was highlighted for *RAR* and *ESR* above, molluscan NRs do not always bind to, or are activated by, their expected ligands. Elucidating possible ecdysteroid biosynthesis pathways, and ecdysone's potential binding affinity to EcR will be important to understand fully any role ecdysteroids might play in molluscan endocrinology.

#### (e) The occurrence of thyroid hormones in molluscs

Over the past decade, the discovery of thyroid biomolecules in molluscs has generated considerable interest, suggesting the potential existence of a thyroid hormone signalling pathway in these animals. Notably, the two main thyroid hormones thyroxine (T4) and triiodothyronine (T3) have been chemically observed in the haemolymph tissues of the gastropod *Achatina fulica* (Lustrino *et al*., 2017), as well as in embryos and other tissues of the bivalves *Rutidapes philippinarum* and *Crassostrea gigas* according to our systematic searches (Huang *et al*., 2015c, 2019; Jiang *et al*., 2019; Song *et al*., 2016). Consequently, mRNA transcripts of the thyroid receptor (*THR*) were measured at several larval development stages of another bivalve, *Mytilus unguiculatus* (Li *et al*., 2020). Interestingly, an RNA interference experiment, which caused the knockdown of *THR* gene expression, revealed significant downregulation of its relative gene expression levels, which in turn led to significant inhibition of larval metamorphosis and a decline in larval viability (Li *et al*., 2020). Such findings indicate the potential involvement of *THR* in the early development of bivalves, however, the activity of *THR* in molluscs does not seem to be a result of binding to T4 or T3 (Huang *et al*., 2015c; Morthorst *et al*., 2023). Nonetheless, gene transcripts that encode multiple thyroid hormone‐metabolising enzymes were also recorded in the Mollusca AND Enzymes inventory. These include transcripts of the enzyme thyroid peroxidase (*TPO*) (Jiang *et al*., 2019; Song *et al*., 2016) which is involved in thyroid hormone biosynthesis in vertebrates, and isoforms of the enzyme deiodinase (*Deio*) known to activate and deactivate thyroid hormones. In fact, the relative mRNA expression of the latter was found to be up‐regulated following exposure of *Crassostrea gigas* larvae to exogenous T4 (Huang *et al*., 2015c).

## IV. DISCUSSION

**Fig. 14:**
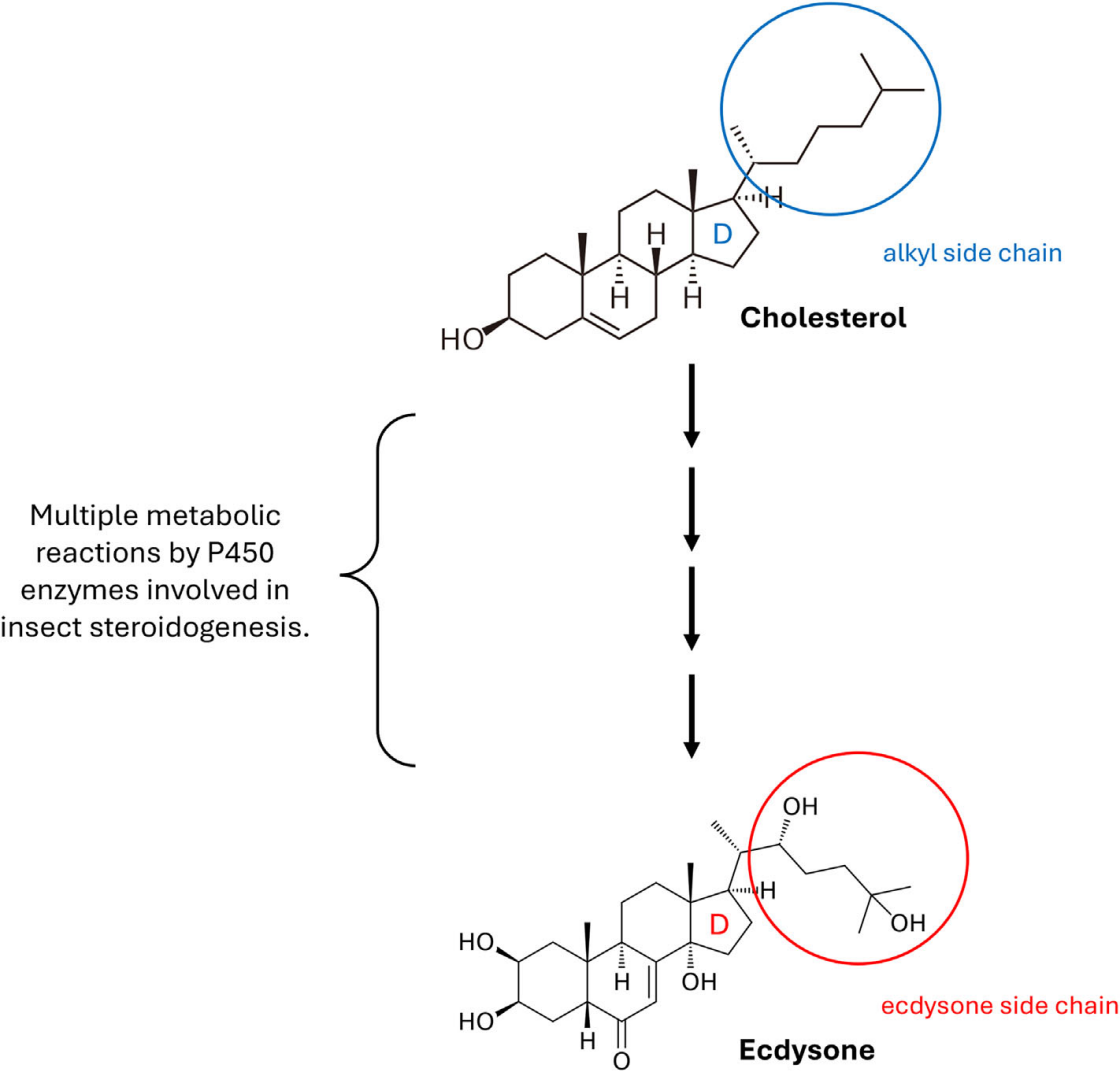
The conversion of cholesterol to ecdysone in insect steroidogenesis after a series of metabolic steps catalysed by P450 enzymes specific to this pathway. The cholesterol molecule contains an alkyl side chain at its D carbon ring which is converted to an ecdysone‐specific side chain attached to the same carbon ring.

Gene expression assays such as quantitative PCR (qPCR) and *in situ* hybridisation can accurately determine the presence of molecules (such as receptors or hormone‐metabolising enzymes) at the genomic (DNA) and transcription (RNA) level (Kirby *et al*., 2007; Nygaard & Hovig, 2009). On the other hand, protein expression assays, like western blots or immunohistochemistry techniques, are only able to detect molecular components at the protein level (Shebl *et al*., 2010). Consequently, the presence of a protein revealed by such assays in an organism does not necessarily mean that it is encoded by a gene in the organism's genome. Indeed, the concept of exogenous protein uptake in molluscs has been known for decades (Bottke, Sinha & Keil, 1982; Bottke & Sinha, 1979). To confirm the endogenous nature of a receptor or hormone‐metabolising enzyme in any organism it is necessary to examine the respective gene at the genome (DNA) and expression (RNA) level (Nygaard & Hovig, 2009; Shebl *et al*., 2010). Fundamental to the question of vertebrate‐type steroidogenesis in molluscs is the *CYP11A1* enzyme, which plays a crucial role during vertebrate steroidogenesis as it presents the very first step of *de novo* steroid biosynthesis from cholesterol (Figs 11 and 12). Positive identification of a molluscan *CYP11A1* was absent from the Mollusca AND Enzymes inventory, as well as from the genomes of molluscs studied through previous transcriptomic and whole‐genome analysis studies (Adema *et al*., 2017; Fodor, Koene & Pirger, 2021). This lack of *CYP11A1* implies that molluscs are unable to metabolise cholesterol to produce pregnenolone, and therefore any of the other subsequent vertebrate‐type steroids *de novo* (Fig. 12). If molluscs are endogenously producing steroids from cholesterol, it is likely their structure(s) will differ from those of vertebrates, as is the case in arthropods. Arthropods use cholesterol to produce ecdysone through a series of metabolic reactions catalysed by a distinct set of P450 enzymes involved in insect steroidogenesis (Petryk *et al*., 2003). Ecdysone's structure varies from that of any vertebrate‐type steroid, such as pregnenolone, as it contains a unique side chain on its D carbon ring (Fig. 14).

If molluscs cannot synthesise vertebrate‐type steroids the considerable body of literature that has measured these steroids, using robust and sensitive analytical chemistry methods, in mollusc tissues needs to be addressed. Why are oestrogens or testosterone detected in molluscan tissues if they are not being produced by these animals? Researchers have proposed that molluscs can take up and accumulate steroids in their tissues from exogenous sources/the environment (Fodor *et al*., 2022; Schwarz *et al*., 2017). For example, a study conducted by Schwarz *et al*. (2017) demonstrated that species within the genus *Mytilus* possess the ability to absorb radiolabelled testosterone from water, esterify it and store it in their tissues. Similarly, Fodor *et al*. (2022) confirmed that the freshwater gastropod *Lymnaea stagnalis* can absorb and esterify a range of steroids from water, including testosterone, 17β‐oestradiol and progesterone.

**Fig. 15:**
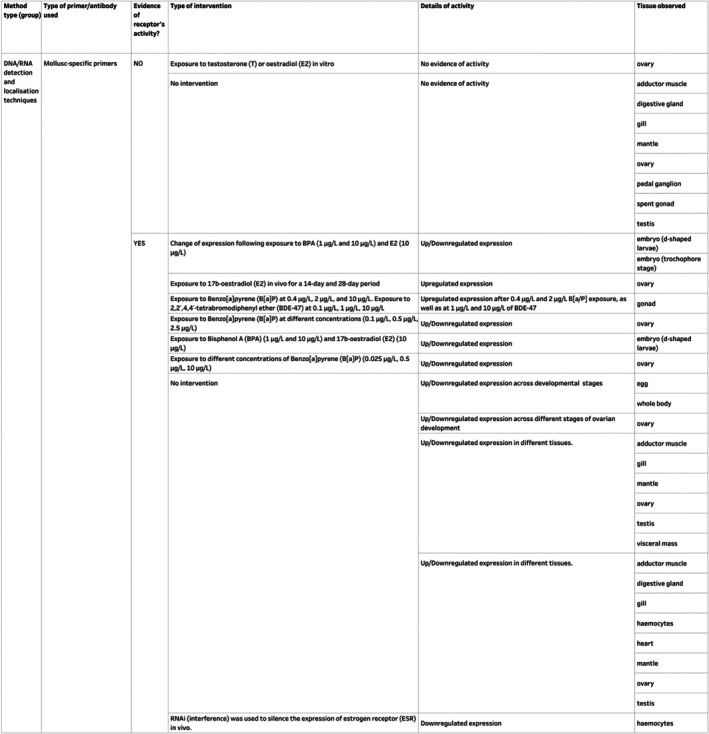
Oestrogen receptor (*ESR*) activity in bivalves as observed from studies included in the Mollusca AND Receptors inventory. Full details on ESR activity, including details on their reported activity, methodological details of included studies and the list of references can be seen in interactive view *via* the links in Appendix S7. References included in this figure are listed in Appendix S7 (Section 3.11).

Additional lines of evidence also need to be considered, beyond the process of steroid synthesis, to consider steroid signalling pathways. The identification of nuclear oestrogen receptor (ESR) in molluscs has been taken as supporting evidence for the biological relevance of oestrogens in molluscs (Lü *et al*., 2016). However, although the *ESR* gene in molluscs was initially thought to be homologous to the vertebrate nuclear *ESR*, a recent study suggested that molluscan *ESR* is in fact orthologous to the common ancestor of vertebrate *ESR* and oxosteroid receptors (androgen receptor, glucocorticoid receptor, mineralocorticoid receptor and progesterone receptor) (Hochberg *et al*., 2020). Moreover, the ligand binding pocket of mollusc ESRs does not bind to oestrogens (or other steroids), and oestrogens do not activate molluscan ESRs (Bridgham *et al*., 2014). This does not mean that mollusc ESRs are redundant. Instead, mollusc ESRs may be constitutively active and regulate gene transcription without a ligand (i.e. steroids). For example, in bivalves, *ESR* expression has been shown to change during key developmental stages (Vogeler *et al*., 2016) (Fig. 15). Additional lines of evidence have indicated the presence of non‐genomic oestrogen signalling in bivalves, suggesting that exogenous oestrogens may be modulating ESRs through independent receptor pathways (reviewed in Balbi, Ciacci & Canesi, 2019). Such alternative pathways may be associated with membrane or cytosolic adaptor proteins and may take place either on the plasma membrane or in the cytosol, rather than the cell nucleus (Balbi *et al*., 2019). However, in the absence of evidence for a functional and specific membrane oestrogen receptor homolog in molluscs, capable of mediating non‐genomic oestrogen signalling (as it does in vertebrates), as well as membrane androgen receptor homologues to respond to testosterone, these suggestions cannot be confirmed. Notably, a recent study by Fodor *et al*. (2024a) demonstrated the unresponsiveness of the membrane‐bound G protein‐coupled oestrogen receptor 1 (*GPER1*) when exposed to oestrogens, thus suggesting an absence of this homologue in *Lymnaea stagnalis*. Additionally, the authors demonstrated the inability of a membrane progesterone receptor (*mPR*) homologue to respond to progesterone in the same model organism (Fodor *et al*., 2024a). Together, it was hypothesised that both genomic and non‐genomic sex steroid signalling may not be conserved in molluscs. In line with these observations, it has been recommended that genes previously termed as molluscan nuclear *ESRs* should be classified as *NR3D* (Fodor *et al*., 2024a; Markov & Laudet, 2011).

Despite poor evidence for vertebrate‐type steroid biosynthesis in molluscs, some vertebrate‐type steroidogenic enzymes were reported to exist in molluscan genomes. Most notably, the genes *CYP17A1*, *HSD17B1*, *SRD5A1* and *SRD5A2*, whose enzymes in vertebrates metabolise progesterone, and rostenedione and testosterone respectively, were all measured in molluscan tissues at the DNA or RNA level. However, the identification of steroidogenic enzymes in molluscan genomes does not confirm the existence of identical enzyme substrates or products to those found in vertebrates. For example, in plants, the *DET2* gene encodes an enzyme that has a significant sequence similarity with mammalian 5αR1/5αR2 (encoded by *SRD5A1*/*SRD5A2* in vertebrates) (Li & Chory, 1999). In vertebrates, 5αRs convert testosterone to dihydrotestosterone. *DET2* has been shown to be a functional ortholog of vertebrate 5αR, catalysing testosterone to dihydrotestosterone under experimental conditions (Li & Chory, 1999; Rosati *et al*., 2003). Although *DET2* has a similar catalytic role as *SRD5A2*, in plants it acts on a different set of substrates, called brassinosteroids (plant steroids), metabolising campesterol to campestenol (Li & Chory, 1999). The presence of enzymes like Det2/5αR in broad groups of organisms (e.g. plants, vertebrates, invertebrates) suggests they have a long evolutionary history (Markov *et al*., 2017) and substrates may differ as organisms evolve.

For example, Markov *et al*. (2017) suggested that newly emerged metabolites can act as substrates for already existing enzymes, and this could lead to either an extension or extinction of metabolic pathways. An interesting example is the late emergence of modern oestrogens (e.g. 17β‐estradiol) in basal vertebrates which in turn outcompeted an already existing group of metabolites called paraestrols (Markov *et al*., 2017). Taken together, the evidence supporting endogenous synthesis of vertebrate‐type steroids in molluscs is unconvincing. Future steroid research in molluscs should be directed towards understanding possible novel substrates, products, and pathways, rather than continuing the vertebrate‐centric approach of the last few decades.

On the other hand, the evident similarities that exist between molluscan and vertebrate retinoic acid signalling pathways indicate the activity of retinoids and a role in molluscan biology. The retinoid system is known as a key regulator of various biological processes in other organisms, from embryonic development and body patterning to reproductive and immune function (OECD, 2021). By contrast, the retinoid system in molluscs (beyond the impacts of TBT on marine gastropods) remains largely understudied. In addition, the evidence for the existence of understudied hormonal signalling pathways in molluscs, including potential biosynthesis of ecdysteroids and thyroid hormones, seems promising. However, the lack of information regarding the activity of retinoid, thyroid and ecdysteroid biomolecules calls for further molecular investigations on various components within those pathways, with potential to unveil insights into the regulatory mechanisms and evolutionary history of molluscan endocrine systems.

## V. FUTURE RESEARCH NEEDS AND PERSPECTIVES

This review provides a comprehensive and systematic overview of molluscan endocrinology. It combines reports of hormones, receptors and enzymes identified in Mollusca with a RoB assessment to consider the robustness of the data presented. Evidently, the historical focus of research on molluscan endocrinology, attempting to discover parallels with the vertebrate steroid hormone signalling pathway, still persists in our three inventories. We also note the frequent use of approaches or methodologies which are not specific enough or reliable in molluscan tissues. Moreover, there is a large disparity between the number of papers investigating vertebrate‐type steroids compared to retinoids, which is surprising given that retinoids have been known to be involved in key developmental processes in molluscs since the early 2000s (Nishikawa *et al*., 2004). There is evidence indicating endogenous production of other hormones, including insect steroids, retinoids and thyroid hormones, in molluscs. This provides opportunities for novel investigations that could unlock a better understanding of molluscan endocrinology.

Given the evidence presented here, we strongly recommend that the research community begins to explore these less‐investigated endocrine pathways in molluscs rather than continuing to focus on a vertebrate‐centric approach (Goździk *et al*., 2023).

In summary, our recommendations are:

(1) Development and use of non‐targeted approaches (e.g. ‘omics) to avoid a vertebrate‐orientated‐bias in molluscan endocrine research. Combinations of metabolomics, proteomics, lipidomics and genomics are needed to support detailed endocrine pathway analysis.
(2) Collaboration between experts in the evo‐devo, analytical chemistry and experimental biology fields should be encouraged.
(3) Further investigations of exogenous hormone uptake in molluscs are needed, e.g. studying the mechanisms through which molluscs take up and store hormones from their environment.
(4) When investigating if a hormone is involved in an animal's endocrinology, a holistic approach should be taken, i.e. by investigating the interactions of hormones confirmed to be present in molluscs (using robust methodologies), with their respective receptors, and with hormone‐metabolising enzymes known to be expressed in molluscan genomes. Other components of relevant metabolic pathways should also be examined.
(5) Expand retinoid signalling research in molluscs by examining the presence and function of understudied retinoic acid signalling biomolecules. These investigations should include a comparative assessment of potential similarities and differences between the molluscan and vertebrate retinoic acid signalling pathway.
(6) Further explore the function of thyroid signalling in molluscs by investigating the role of thyroid hormones T3 and T4, as well as the function of *THR* during molluscan development.
(7) Explore the presence of ecdysteroid signalling in molluscs by investigating the occurrence and function of related biomolecules in molluscs. Investigations should also focus on the uptake and conversion of Δ^5^ (phyto)sterols to Δ^5,7^ sterols in molluscs and their potential involvement in ecdysteroid biosynthesis.
(8) For all such investigations, robust analytical methods must be implemented, such as investigations performed at the molecular level to confirm the presence of respective biomolecules at the genome (DNA) and expression (RNA) level using appropriately validated approaches. Protein‐level investigations should implement mollusc‐specific antibodies to ensure reliability of findings.

## VI. CONCLUSIONS

(1) This study provides a fully searchable database that comprises the systematic categorisation and critical appraisal of 9 years of evidence on molluscan endocrinology. This resource is available for the scientific community to use and aims to assist in directing future research efforts towards the exploration of understudied hormonal pathways in molluscs using robust methodological design.
(2) At present, the occurrence of cholesterol biosynthesis in molluscs remains poorly understood and lacks supporting evidence. Although some key enzymes such as *CYP51* have been identified, evidence for their metabolic activity in molluscs remains sparse. Consequently, the absence of key steroidogenic genes from molluscan genomes, including *CYP11A1* and *CYP21A1*, suggests that molluscs are unable to synthesise vertebrate‐type steroids *de novo*.
(3) Existing evidence on retinol metabolism in molluscan tissues suggests partial similarity between the vertebrate and molluscan retinoic acid signalling pathway. However, more research is needed in molluscs to elucidate the role of understudied biomolecules that are involved in this pathway.
(4) The occurrence of thyroid hormones and the reported activity of the thyroid receptor in molluscs, highlights potential involvement of these biomolecules in developing bivalves, but existing evidence on the role of thyroid signalling in these animals remains sparse.
(5) The presence of a wide range of phytosterols and the production of Δ^5,7^ sterols in molluscs indicates potential endogenous synthesis of insect steroid hormones (e.g. ecdysteroids). Further research should be accompanied by evidence for the presence and activity of their respective nuclear receptors.
(6) Future investigations should implement robust experimental designs and analytical methodologies to study molluscan endocrine systems and should focus on the investigation of understudied or novel hormonal pathways in these animals.

## AUTHOR CONTRIBUTIONS

K.P.: Writing – Original Draft, Review & Editing. K.P. is the guarantor of the systematic evidence map and was also responsible for drafting the protocol, the search strategy, the data extraction template, the RoB tool, RoB guidelines and the manuscript. K.P. is responsible for the data extraction of all eligible studies, their assessment for RoB, and the visualisation of data. A.B.: Writing – Review & Editing, Methodology, Supervision, Conceptualization, Resources, Project Administration. A.B. provided expertise and specialist knowledge in the subject area, contributed to the development of the search strategy and participated in initial screening. A.B. provided feedback on the conduct of all pilot activities, including that of data extraction and RoB assessment, as well as the manuscript. O.V.M: Writing – Review & Editing, Methodology. O.V.M. contributed knowledge and good practice of systematic evidence map methods as a systematic review expert. T.H.M.: Writing – Review & Editing, Supervision.

## Supporting information


**Appendix S1.** Protocol for a systematic evidence map on hormone biosynthesis in Mollusca.

**Appendix S2.** Updates made to the final protocol for the systematic evidence map of hormone biosynthesis in Mollusca.

**Appendix S3.** Pilot search for a systematic evidence map of hormone biosynthesis in Mollusca.

**Appendix S4.** Data extraction template.

**Appendix S5.** Risk‐of Bias tool and data.

**Appendix S6.** Risk‐of‐Bias tool guidelines.

**Appendix S7.** Supplementary information.

## Data Availability

The data that supports the findings of this study are available in the supplementary material of this article. Data supporting this study, including links to the interactive online database, are included within the supporting information. Data on the draft protocol are available in Panagiotidis (2022).
