# Supplementary material for: Mapping molluscan endocrinology: a systematic and critical appraisal
Source: Biol Rev Camb Philos Soc. 2025 Dec 16;101(2):970–1002. doi: 10.1002/brv.70112 (PMC12965858; doi:10.1002/brv.70112)
Supplement: Supplementary file 1 — Appendix S1. Protocol for a systematic evidence map on hormone biosynthesis in Mollusca. [file BRV-101-970-s003.docx]

Appendix S1. Protocol for a systematic evidence map on hormone biosynthesis in Mollusca

**Mapping Molluscan Endocrinology: A Systematic and Critical Appraisal**

**Authors:** Konstantinos Panagiotidis^1*^, Thomas H. Miller^1^ , Olwenn V. Martin^2^, Alice Baynes^1^ **Affiliation addresses:**

^1^*Environmental Sciences, Departure of Life Sciences, Brunel University London, Kingston Ln, London, Uxbridge UB8 3PH, UK*
^2^*Department of Arts and Science, Faculty of Arts & Humanities, University College London, Gower St, London WC1E 6BT, UK*

**Correspondence:** Konstantinos Panagiotidis ([constantinospan@outlook.com](mailto:constantinospan@outlook.com))

In accordance with the PRISMA-P 2015 guidelines, the first version of the systematic review protocol was registered with the open-access repository Zenodo on 12 July 2021. Version 2 of the protocol was uploaded on 8th September 2022 (doi: 10.5281/zenodo.7061510) and was last updated alongside the publishing of the manuscript.

Authors:
Konstantinos Panagiotidis, Department of Life Sciences, Brunel University London, [Konstantinos.panagiotidis@brunel.ac.uk](mailto:Konstantinos.panagiotidis@brunel.ac.uk)

Dr Olwenn V Martin, Department of Life Sciences, Brunel University London, [olwenn.martin@brunel.ac.uk](mailto:olwenn.martin@brunel.ac.uk)

Dr Alice Baynes, Department of Life Sciences, Brunel University London, [Alice.Baynes@brunel.ac.uk](mailto:Alice.Baynes@brunel.ac.uk)

Author contributions

K.P. is the guarantor of the systematic evidence map and was also responsible for drafting the protocol, the search strategy, the data extraction template, the Risk-of-Bias tool, Risk-of-Bias guidelines and the manuscript. K.P. is responsible for the data extraction of all eligible studies, their assessment for Risk-of-Bias, and the visualisation of data. A.B. provided expertise and specialist knowledge in the subject area and contributed to the development of the search strategy. A.B. provided feedback on the conduct of all pilot activities, including that of data extraction and Risk-of-Bias assessment, as well as the manuscript. O.V.M contributed her knowledge and good practice of systematic evidence map methods as a systematic review expert.

Funding
This systematic evidence map will be incorporated into Konstantinos Panagiotidis’ doctoral thesis and is funded by The London NERC DTP in collaboration with the UK Research and Innovation (UKRI).

1. **INTRODUCTION**
   1. **Molluscs as biomarkers of pollution**

Molluscs represent one of the most diverse phyla of the animal kingdom with seven molluscan classes and more than 130,000 species (Oehlmann *et al*., 2007). Molluscan species represent a significant part of biodiversity and are remarkably influential for ecosystem functioning (Oehlmann *et al*., 2007). Aside from their ecological importance, molluscs also play a key role in ecotoxicological research; they possess a range of characteristics that make them attractive biomarkers for the study of pollution in both aquatic and terrestrial environments. The occurrence of imposex in gastropod snails, following exposure to the organotin tributyltin (TBT), has drawn major scientific attention over the past three decades and represents one of the most widely studied cases of endocrine disruption in aquatic invertebrates (Blaber, 1970; Gibbs *et al*., 1987). To date, the underlying mechanisms of TBT-induced imposex in molluscs have yet to be fully elucidated. This is due to the lack of a fundamental understanding of molluscan endocrinology which has also led to failure in regulating the distribution of TBT in the past. Although TBT is well regulated now, the biggest challenge for ecotoxicologists is to prevent these detrimental chemicals entering the environment rather than stopping them once they have impacted wildlife populations. Hence, if an adequate understanding of molluscan endocrinology or appropriate regulatory testing is not achieved, it is expected that mollusc populations might be at risk of similar consequences in the future.

- 1. **Molluscan *vs* vertebrate steroidogenesis**

Several vertebrate-type steroids and steroidogenesis-related genes have been identified in molluscan tissues. The presence of androgens (i.e. testosterone) in the tissues of some gastropod molluscs led to the assumption that these species have the ability to biosynthesise or metabolise vertebrate steroids *de novo* (Lafont, 1991; Lafont & Mathieu, 2007; Lehoux & Sandor, 1970). Recently, however, concerns have arisen on whether molluscs use vertebrate-type steroids in their reproduction and development. Kaur *et al*. (2016) demonstrated that exposure of *Biomphalaria glabrata* (Mollusca: Gastropoda) to potent vertebrate androgens [i.e. 5α-dihydrotestosterone (DHT) and 17α-methyltestosterone (MT)] did not affect the growth nor the development of its reproductive organs. Such evidence makes it unlikely that vertebrate steroid androgens are involved in the reproductive development of these freshwater gastropods*.* Additionally, genomic searches have shown that the genetic material of molluscs (and other invertebrates) does not contain the cholesterol side-cleavage enzymes (CYP11A) essential for vertebrate sex hormone steroidogenesis (Markov *et al*., 2017). The absence of CYP11A enzymes in the molluscan genome reflects the inability of these animals to process cholesterol and thus biosynthesise vertebrate-type steroids *de novo* (Adema *et al*., 2017). Therefore, it is fair to assume that the structure of molluscan sex hormones might differ from those of vertebrates, although their identification has yet to be achieved.

- 1. **Insect *vs* molluscan steroidogenesis**

In insects and nematodes, steroid hormones are considered necessary for controlling development, metamorphosis, and diapause. Similar to vertebrates, insects use cholesterol as a precursor for their steroid biosynthesis. Insects can also use plant sterols as precursors for steroidogenesis although these sterols, including cholesterol, must be obtained from their diet (Niwa & Niwa, 2014). Apart from vertebrate-type steroids, arthropod steroids (i.e. ecdysteroids) were also identified in molluscs (Whitehead, 1977) in early experiments. However, studies have failed to provide evidence of ecdysteroid biosynthetic pathways in molluscs as no orthologous genes to those involved in arthropod steroidogenesis have been identified in their tissues (Adema *et al*., 2017; Lafont & Mathieu, 2007). Unlike molluscs, a great number of molecules responsible for the regulation of steroidogenesis in Ecdysozoa have been successfully identified and characterised during the past 20 years. A careful investigation of how enzymes that metabolise ecdysteroids are conserved within arthropods could provide insights into the diversity amongst steroids and between different phyla (Niwa & Niwa, 2014).

Although the synthesis of ecdysteroids is based on cholesterol, insects use different steroidogenic enzymes than vertebrates. Amongst them, the functions of the enzymes Neverland (*Nvd*), Phantom (*CYP306A1*), Disembodied (*CYP302A1*), Shadow (*CYP215A1*) and Shade (*CYP314A1*), have been validated both *in vivo* and *in vitro* (Niwa & Niwa, 2014). The first step of steroidogenesis in insects is the conversion of cholesterol to 7-dehydrocholesterol (7-dC) which is modulated by Nvd. On the other hand, the conversion of 5β-ketodiol to 20-hydroxyecdysone (20E) is catalysed by a sequence of cytochrome P450 monooxygenases namely Phantom (*CYP306A1*), Disembodied (*CYP302A1*), Shadow (*CYP215A1*) and Shade (*CYP314A1*) (Niwa *et al*., 2005, 2004; Petryk *et al*., 2003; Warren *et al*., 2002, 2004).

- 1. **The use of sterols in molluscan steroidogenesis**

Sterols are a subgroup of steroids that can be found on the cell membranes of animals, plants and microorganisms and that possess the ability to bind on and condense the lipid bilayer (Urich & Urich, 1994). Cholesterol is the most important type of sterol in animal tissues and functions as a precursor to steroid hormones and fat-soluble vitamins. However, the potential endogenous function of sterols in molluscs remains unclear. Aromatised sterols (i.e. ‘paraestrols’) have been identified in cnidarians and sponges (Markov *et al*., 2017). Paraestrols are metabolites of cholesterol that have not been subjected to side-chain cleavage, and are thought to be the original ligands for the ‘ancestral’ steroid oestrogen receptor (ESR) in both molluscs and vertebrates. It has been speculated that paraestrols were probably not as efficient in binding to ESRs as other compounds (i.e. steroids) that resulted from the evolution of side-chain cleavage in vertebrates (Markov *et al*., 2017). It was also demonstrated that sterols (including testosterone and aldosterone) are substrates of the steroidogenic enzyme 5-alpha-reducatse (5αR) which is present in both molluscan and plant genomes. Thus, further examination of sterol derivatives might shed light on the elucidation of molluscan endocrinology and the biosynthesis of hormones in molluscs (Fodor *et al*., 2020).

- 1. **The role of nuclear receptors in molluscan endocrinology**

Evaluation of hormonal functions in molluscs not only relies on the presence of steroids in their tissues, but also on the occurrence of specific nuclear or membrane-bound receptors in their genomes (Ni *et al*., 2013). Apart from vertebrate-type and invertebrate-type steroids, several nuclear hormone receptors have also been identified or suggested to exist in molluscs. Nuclear receptors (NRs) belong to a family of ligand-modulated transcription factors specific to metazoans that are known to translate signalling messages of molecules into transcriptional responses (Miglioli *et al*., 2021). Most signalling pathways, therefore, involve the binding of a signalling molecule (e.g. a ligand) to NRs which in turn trigger events inside the cell. Upon binding, the NR goes through a conformational change known as receptor activation. Amongst others, typical ligands for NRs can include a range of steroid hormones such as progesterone and testosterone, as well as retinoids which are derivatives of Vitamin A (Norman & Litwack, 1997*a*).

In vertebrates, NRs are key players in several embryological and physiological processes including reproduction, metabolism, and development (Miglioli *et al*., 2021). It is also thought that NR signalling plays an important role in the regulation of several developmental processes in marine invertebrates. Due to their ligand-dependent activity, NRs are susceptible to endocrine disruption. Consequently, a certain set of vertebrate NR orthologs including the retinoic acid receptor (RAR), the retinoid X receptor (RXR) as well as oestrogen receptors (ER, ERR) have now started to be characterised in invertebrates with the aim of developing a greater understanding of their underlying mechanisms (Miglioli *et al*., 2021). To date, we have developed a good understanding of how NRs are able to regulate transcription though ligand binding, however our knowledge around NR action has been heavily focused on vertebrate models. Hence, questions still remain about the evolutionary origin of NRs and particularly, around the evolutionary elaboration of certain ligand–receptor pairs (Gutierrez-Mazariegos *et al*., 2014). Lecroisey *et al*. (2012) demonstrated that certain ligand–receptor pairs might have undergone modifications in their ligand-binding processes during animal microevolution which is further evidence that NRs might have not remained unaffected over time. Additionally, in contrast to what was initially thought, orthologs of ESRs that have been previously identified in molluscs were shown to be insensitive to oestradiol and thus incapable of binding oestrogens (Iguchi *et al*., 2007; Thornton & Need, 2003; Tran *et al*., 2016). Similarly, the presence of RARs in the genomes of Lophotrochozoa (e.g. molluscs, annelids) does not necessarily mean that RARs possess a functional ability (Gutierrez-Mazariegos *et al*., 2014). It has been shown that RARs identified in molluscs were unable to bind and thus activated by their respective retinoic acid ligands, 9-cis-RA and all-trans-RA (André *et al*., 2019). Hence, this is a further indication that certain NRs found in molluscs have possibly lost their functional ability during evolution (Gutierrez-Mazariegos *et al*., 2014).

By contrast, orthologs of the RXR identified in molluscan tissues were previously shown to be involved in endocrine-disruption processes and are thought to play an important role in molluscan sexual development. In vertebrates, RXR is a key player in cellular endocrine processes with its unique ability to act as a heterodimer or homodimer with other NRs (including RAR and the peroxisome proliferator-activated receptor, PPAR) (Fonseca *et al*., 2020). Recent findings suggest that TBT-induced imposex in molluscs is a result of the unusual modification of the RXR signalling pathway caused by TBT exposure (Horiguchi, 2017). The binding of TBT to RXR was shown to initiate the activation of PPARγ, which functions as a heterodimerisation partner of RXR, which in turn induces imposex in female molluscs (Giulianelli *et al*., 2020). These data suggests that retinoids as well as their interaction with the RXR/ PPARγ complex play a significant role in the development of male reproductive organs in aquatic molluscs (Giulianelli *et al*., 2020). Although it is still unclear to what extent the adverse effects of endocrine-disrupting chemicals are influenced by NRs in invertebrates, it seems likely that NRs have an active role in their embryonic and post-embryonic development (Bodofsky *et al*., 2017; Handberg-Thorsager *et al*., 2018; Vogeler *et al*., 2016).

- 1. **Steroidogenesis-related genes identified in molluscs**

Recent advances in genome sequencing technology have been used in molluscan models for the discovery of genes and key enzymes that could be involved in molluscan steroidogenesis. Consequently, the identification of vertebrate-type steroid hormones in molluscs needs to be accompanied by fundamental evidence of their biosynthetic pathways that confirms their involvement in endogenous steroidogenesis. For example, Thitiphuree *et al*. (2019) recently confirmed the expression of several steroidogenic genes in molluscs that are known to encode steroid-metabolising enzymes. Amongst these, Thitiphuree *et al*. (2019) revealed an ever-present expression of genes for the steroid-metabolising enzymes *star*, *cyp17a*, *hsd17b*, and *hsd3b* in the peripheral and gonadal tissues of the scallop *Mizuhopecten yessoensis*. Additionally, the study demonstrated that *hsd3b* and *hsd17b* showed a synchronous pattern related to gonad maturity levels, suggesting their possible involvement in scallop steroidogenesis (Thitiphuree *et al*., 2019).

Moreover, despite previous indications of the absence of vertebrate-type androgens in molluscan reproductive development, homologues of the steroidogenic gene for 5αR have been identified in the genome of the freshwater gastropod *Biomphalaria glabrata* (Adema *et al*., 2017). In vertebrates 5αR is known to convert testosterone (T) to DHT, as well as being involved in conversion of other steroids such as cortisol to 5α-corticosterone or progesterone into 5α-dihydroprogesterone (Baynes *et al*., 2019). In *B. glabrata* snails, homologues of 5αR were found to be expressed during embryonic development although the role of this enzyme in gastropods remains unknown. Previous observations of 5αR transcripts in the mantle tissue of *B. glabrata* (Adema *et al*., 2017) raised questions regarding a potential link of this enzyme with molluscan shell formation. Interestingly, mammalian 5αR has been shown to exhibit significant sequence similarities with de-etiolated-2 (DET2), the prime enzyme used for the synthesis of plant steroids (i.e. brassinosteroids) (Li & Chory, 1999). In plants, DET2 catalyses the conversion of campesterol to campestanol. Surprisingly, under experimental conditions it was also shown that DET2 is able to convert T to DHT which suggests that DET2 is a functional ortholog of 5αR in plants (Li *et al*., 1997).

1. **SCIENTIFIC RATIONALE AND METHODOLOGICAL APPROACH**

The continuing debate around the presence of vertebrate-type steroids in molluscs as well as their possible involvement in molluscan reproduction and development, reflects the gap of knowledge that exists within molluscan endocrinology. Although previous attempts to address the occurrence of vertebrate-type steroids as well as the expression of steroidogenesis-related genes in molluscs (Cuvillier-Hot & Lenoir, 2020; Horiguchi & Ohta, 2020; Scott, 2012) are informative, doubts remain as to whether these steroids possess a functional role. A systematic collection of evidence on the presence of different hormones, hormone receptors and hormone-metabolising enzymes identified in Mollusca remains needed. This systematic evidence map aims to: (1) provide a comprehensive assessment of our current understanding of hormone biosynthesis in molluscs through an appropriate evaluation of the evidence that exists in the wider literature and (2) highlight gaps in our knowledge for future research. In the hope of developing a more thorough understanding of molluscan endocrinology this review will thus encompass three research questions:

- What evidence is there for different hormones in molluscan tissues?
- What evidence is there for different hormone receptors in molluscan tissues?
- What evidence is there for different hormone-metabolising enzymes in molluscan tissues?

The protocol was drafted according to the PRISMA-P (Preferred Reporting Items for Systematic review and Meta-Analysis Protocols) 2015 checklist (Shamseer *et al*., 2015) as well as the “Recommendations for the conduct of systematic reviews in toxicology and environmental health research (COSTER)” (Whaley *et al*., 2020). A draft version of the protocol was published on *Zenodo* on 14th July 2021 (Panagiotidis, 2021; doi: 10.5281/zenodo.4693859) and reviewers were invited to send their comments until 17th September 2021. No responses were received *via* the form provided, however, feedback was collected *via* email communication (Panagiotidis, 2022). Upon receiving feedback from reviewers, the protocol was updated extensively, and the second version was uploaded on Zenodo on 8^th^ September 2022 (Panagiotidis, 2022) (doi: 10.5281/zenodo.7061510). The final amendments made to the protocol are documented in Appendix S2.

**2.1. Eligibility criteria**

Each research question is defined by a separate PO (Population, Outcome) statement as well as PO-specific inclusion and exclusion criteria. The inclusion and exclusion criteria presented in the draft protocol (Panagiotidis, 2021) were revised upon pilot screening activities and are documented in Panagiotidis (2022). The eligibility criteria were further updated alongside the preparation of the systematic map review article and presented in Table S1.1. The changes made to the eligibility criteria are discussed in detail in Appendix S2.

**Table S1.1.** Population, Outcome (PO) statements and inclusion and exclusion criteria.

| **PO Statements** |  | **Inclusion criteria** | **Exclusion criteria** |
| --- | --- | --- | --- |
| **1. The presence of hormones in molluscan tissues: Mollusca AND Hormones** | | | |
| Population | Mollusca (bivalves, gastropods, cephalopods, polyplacophores, scaphopods, Aplacophora, Monoplacophora) of any life stage (embryos, juveniles, adults) and sex (female, male, hermaphrodite) |  |  |
| Outcome | Presence of hormones (vertebrate-type and invertebrate-type) in molluscan tissues which include:  *Primary outcomes*:  – Mollusc class, species, sex  – steroid/sterol measured  – their reported concentrations or range of reported concentrations  – tissue observed.  *Secondary outcomes:*  – concentration variability between seasons if reported (i.e. seasonality)  – change of steroid concentrations due to an intervention (e.g. chemical exposure)  – method specifications (limit of detection (LOD), method name, extraction procedure, if positive and/or negative controls were used, method validation, etc.)  – repetition of experiments. | Objectively measured vertebrate-type steroids and invertebrate-type steroids (e.g. ecdysteroids).  Objectively measured retinoids involved in the retinoic acid pathway (retinoic acid, retinal, retinol).  Objectively measured hormones involved in thyroid signalling pathway.  Studies that examined the biological significance of objectively measured sterol concentrations in molluscs and consequently highlight important information about molluscan biology (e.g. how sterols are used in or by the mollusc).  Objectively measured hormones from control (i.e. non-exposed) animals, from ecotoxicological studies that used a chemical intervention (e.g. exposing molluscs to a steroid or a set of steroids). | Data from studies published before 2012.  Data from review studies.  Neurohormones (e.g. dopamine, serotonin, etc.) or anabolic hormones (e.g. insulin).  Hormones measured in environmental-monitoring studies examining bioaccumulation of contaminants in molluscs (e.g. synthetic steroids including hormones derived from pharmaceutical products such as growth promoters or any other hormones that are considered polluting substances instead of an endogenous synthesis).  Hormones derived from studies that measured sterol/lipid composition in molluscs but do not provide important information about their biological significance in molluscs (e.g. studies investigating the dietary significance of sterols). |
| **2. The presence of hormone receptors in molluscan tissues: Mollusca AND Receptors** | | | |
| Population | Mollusca (bivalves, gastropods, cephalopods, polyplacophores, scaphopods, Aplacophora, Monoplacophora) of any life stage (embryos, juveniles, adults) and gender (female, male, hermaphrodite) |  |  |
| Outcome | Presence of receptors (by examining the expression of receptor genes or proteins) in molluscs which include:  *Primary outcomes:*  – mollusc class, species, gender  – receptor identified  – tissue in which is expressed  – relative expression compared to housekeeping gene(s) (if applicable)  – evidence of receptor’s activity.  *Secondary outcomes:*  – changes in expression due to an intervention (e.g. between seasons, chemical exposure, etc.)  – method specifications (method type, method name, validation of housekeeping genes)  – data on conservation analyses amongst receptors (e.g. phylogenetic trees and/or sequence conservation analyses showcasing homologies amongst ligand- or DNA- binding domains, between different species/class/phyla, etc.)  – repetition of experiments. | Objectively measured hormone  receptors, including retinoid receptors and receptors known to be directly or indirectly involved in hormone signalling pathways, including thyroid hormone signalling. | Data from studies published before 2012.  Data from review studies.  Neurohormone receptors.  Data from silico/computational studies which did not conduct any molecular work.  Data from transcriptomic studies or genome-wide identification studies. Data extraction from such studies is difficult to achieve with accuracy and efficiency. Data from such studies are used only as a point of reference for the discussion of results. |
| **3. The presence of hormone-metabolising enzymes in molluscan tissues: Mollusca AND Enzymes** | | | |
| Population | Mollusca (bivalves, gastropods, cephalopods, polyplacophores, scaphopods, Aplacophora, Monoplacophora) of any life stage (embryos, juveniles, adults) and gender (female, male, hermaphrodite) |  |  |
| Outcome | Studies that measured the expression of hormone-metabolising proteins or genes (vertebrate origin and/or invertebrate origin) in molluscs, which include:  *Primary outcomes:*  – mollusc class, species, gender  – hormone-metabolising enzyme/gene identified  – tissue in which is expressed  – relative expression reported  – evidence of steroidogenesis-related gene activity.  *Secondary outcomes:*  – changes in expression due to an intervention (e.g. between seasons, chemical exposure, etc.)  – method specifications (method type, method name, validation of housekeeping genes)  – data on conservation analyses amongst hormone-metabolising enzymes/ genes (e.g. phylogenetic trees and/or sequence conservation analyses showcasing homologies amongst gene(s) of interest and between different species/class/phyla, etc.)  – repetition of experiments. | Objectively measured hormone-metabolising enzymes or genes. These include key enzymes involved in vertebrate and invertebrate steroidogenesis (e.g. insect steroidogenesis), thyroid hormone signalling, as well as transport proteins and enzymes involved in the production and breakdown of retinoids (e.g. enzymes in the metabolism of Vitamin A/retinol including RBP, CRBP, SDR, CYP26).  For chemical intervention studies, data on enzyme or gene expression obtained only from control (non-exposed) animals. | Data from studies published before 2012.  Data from review studies.  Observations on enzyme or gene expression after chemical interventions (e.g. gene expression in exposed molluscs).  Studies that examined neurological processes related to retinoids.  Studies that investigated vitellogenin expression.  Enzymes involved in metabolising neurohormones.  *In silico*/computational studies that did not conduct molecular work.  Transcriptomic studies or genome-wide identification studies.  Data extraction from such studies is difficult to achieve with accuracy and efficiency. Thus, data from such studies are used only as a point of reference for the discussion of results.  Enzymes of the CYP450 superfamily involved in xenobiotic metabolism and detoxification processes (according to the study aims/objectives). Usually members of the CYP1, CYP2 and CYP3 families, these enzymes include: CYP1A2, CYP2A6, CYP2C9, CYP2C19, CYP2D6, CYP2E1 and CYP3A4. Note: due to the involvement of many CYP450 enzymes in the metabolism of both xenobiotic and endogenous compounds, excluded studies were assessed according to their relevance to the objectives of this protocol. |

CRBP: cellular retinol binding protein; RBP: retinol binding protein; SDR: short-chain dehydrogenase/reductases (Kin *et al*., 2012).

**2.1.1. *Study design***

Eligible studies included in the systematic evidence map were any peer-reviewed publications that objectively measured hormones, receptors, and/or hormone-metabolising enzymes in any molluscan species. Data from review studies were not extracted, and thus are not incorporated in the evidence report. However, review studies identified as relevant to the PO statements were used as supporting material for the discussion of the extracted data. Specific characteristics of eligible studies are listed in Table S1.1.

**2.1.2. *Timing***

For inclusion, studies must be published between 2012 and 10th September 2021. The start date cut-off was chosen according to the following observations:

- Molluscan genome sequencing became more prominent during the past decade (Takeuchi, 2017), with advances in this technology revealing consistent expression of genes associated with hormone metabolism in molluscs, including hormone receptor transcripts. Whole-genome sequencing in any organism, and particularly in molluscs, makes gene identification analysis more comprehensive. A fully annotated (or at least semi-annotated) genome comprises important information about the functional elements along with the entire genome sequence, thus providing potential functions for newly obtained DNA sequences (Abril & Castellano, 2019). DNA sequencing technology has increased the quality and completeness of whole genome analyses, which can in turn be used to gain insights into various biological processes (Abril & Castellano, 2019).
- During the last decade, chemical analysis [e.g. Gas Chromatography–Mass Spectrometry (GC-MS), Liquid Chromatography–Mass Spectrometry (LC-MS)] has exhibited higher precision in detecting low concentrations of steroids and other metabolites than was possible using traditional immunoassay methods (Gust *et al*., 2010; Krasowski *et al*., 2014). Immunoassays used to be common practice in molluscan endocrinology research and were generally considered reliable. The date range cut-off at 2012 thus was applied to eliminate bias or outdated information from the data extraction inventory which could affect the quality of the evidence report.

**2.1.3. *Language***

Only articles written in English were included.

**2.1.4. *Setting***

No restrictions were applied according to the type of setting of each study.

**2.2. Information sources**

The searches for peer-reviewed papers and articles were conducted in the bibliographic databases *PubMed*, *Web of Science* and *Scopus*.

Data on the identification of hormones, hormone receptors and hormone-metabolising enzymes in Mollusca were considered eligible for inclusion only if presented in peer-reviewed literature conducted in an academic or research environment. Grey literature was not searched to avoid including inaccurate or misleading information. Manual searches of the citations and bibliography of eligible studies were carried out in cases where highly important data might have been absent or not explicitly addressed in the study of interest. Date limitations on retrieved papers were not applied during the literature search, as this led to significant inaccuracies during citation export in trial searches (e.g. the number of exported citations did not match the number of retrieved papers during the original search when a date limit was applied). Therefore, all retrieved papers were exported from each database individually, duplicates removed (using the reference management software *Zotero*), and then assessed for inclusion (using the screening tool *Rayyan*), based on the eligibility criteria described above.

**2.3. Search strategy**

**2.3.1. *Defining ‘Population’***

To devise a specific search strategy inclusive of all relevant literature, we piloted three sets of keyword strings relevant to our research questions and PO statements. The population was defined as Mollusca, including all seven molluscan living classes (Gastropoda, Bivalvia, Polyplacophora, Cephalopoda, Scaphopoda, Aplacophora, Monoplacophora). The two extinct classes of Mollusca (Rostroconchia and Helcionelloida) were excluded from the search strategy. Additionally, several mollusc-specific terms including “oysters”, “mussels”, “squids” and “chitons” were also defined as key objectives and included.

**2.3.2. *Defining ‘hormones’, ‘hormone receptors’ and ‘hormone-metabolising enzymes’***

The presence of both ‘vertebrate-type’ and ‘non-vertebrate’ steroid hormones has been reported in molluscs, although their origin and synthesis has yet to be elucidated. This systematic map combined all available evidence from 1st January 2012 to 10th September 2021 (the day all papers were extracted) on the identification of different hormones, hormone receptors and hormone-metabolising enzymes reported in molluscan tissues. Data on 24 steroid hormones known to be involved in vertebrate steroidogenesis were extracted from Häggström & Richfield (2014) and Fodor *et al*. (2020). Additionally, four identified ecdysteroids (insect steroids) involved in arthropod steroidogenesis were extracted from Niwa & Niwa (2014) and were included as part of the “Mollusca AND Hormones” search string. To avoid missing important literature, generic terms such as “sterols”, “hormones” as well as synonyms for each included steroid were identified and included in the search. Data on retinoids was captured *via* the “Mollusca AND Receptors” keyword string while data on hormones involved in neurohormonal signalling was outside the scope of this review.

In the draft protocol (Panagiotidis, 2021), the search string for “Mollusca AND Receptors” comprised a set of general receptor terms connected using the Boolean operator ‘AND’ to an additional 147 receptor names found to be expressed in molluscs. These were initially extracted from the papers of Vogeler *et al*. (2014) and Kaur *et al*. (2015). However, upon revision of the search strategy, we noticed that the additional “AND” was narrowing results of searches on the specific receptors: Mollusca AND “general terms for receptors” AND “other specific terms”. To make the search more inclusive, the string was updated as part of the second version of the protocol (Panagiotidis, 2022). In the second version, general terms for hormone receptors were added and specific receptor names were removed. Consequently, the “Mollusca AND Receptors” PO aimed to collect data on the occurrence of hormone receptors, including retinoid receptors and those known to be directly or indirectly involved in other hormone signalling pathways, such as the thyroid hormone signalling pathway. Data on receptors involved in neurohormonal signalling were outside the scope of this review.

Finally, the keyword string for“Mollusca AND Enzymes” initially aimed to capture information on the enzymes involved in vertebrate steroidogenesis, insect steroidogenesis, and retinoid signalling (Panagiotidis, 2022). However, an interest in thyroid signalling enzymes was developed after the publication of the second protocol. These were included in the eligibility criteria as part of the systematic map manuscript. A list of genes encoding enzymes involved in vertebrate steroidogenesis were identified and extracted from *Wikipedia*. The ‘Mollusca AND Enzymes” string comprised 44 terms identified as the encoding genes for every steroidogenic enzyme previously found in vertebrates. In addition, 10 genes known to be involved in insect steroidogenesis were identified from Niwa & Niwa (2014) and included in the search. The search also included names of key transport proteins and enzymes involved in retinoid signalling. To avoid missing important literature, our search strings included gene synonyms and topical vocabulary for steroidogenesis-related processes. The amended search strategy and the updated search strings can be found in Appendix S3.

**2.4. Data management**

Initially, the systematic evidence map and literature review process were intended to be managed with the support of the online tool *CADIMA* (Kohl *et al*., 2018). However, trial screening activities found *CADIMA* to be over-complicated and time-consuming in managing and screening literature. Thus, the online tool *Rayyan* (Ouzzani *et al*., 2016) was chosen as the most appropriate tool.

**2.4.1. *Relevance screening***

Duplicate records from all exported citations were removed with the help of the reference management software *Zotero*. After duplicate removal, the remaining records were imported to *Rayyan* for screening. The list of inclusion & exclusion criteria was applied to 20% (pilot screening) of the merged citation list in duplicate (i.e. by K.P. and A.B. working independently) at two different stages. The first stage involved the pilot screening of title and abstract of obtained studies to identify relevance to the research questions. This led us to update the eligibility criteria in the second version of the protocol (Panagiotidis, 2022). The online tool *Rayyan* facilitated the process of consistency checking by identifying conflicting decisions between the two coders. Disagreements during pilot screening were resolved through discussion by the two coders, thus allowing amendment of the eligibility criteria and the continuation of the screening process by one of the two evaluators (K.P.). The results of pilot screening activities are summarised in the supplementary material of the second protocol (Panagiotidis, 2022). The second stage of the screening process involved full-text screening of the selected studies. The eligible studies were then included in the data extraction inventory. Reasons for exclusion were recorded during full-text screening.

**2.4.2. *Data extraction***

The hormones, hormone receptors and hormone-metabolising enzymes present in molluscs differed in terms of chemical structure, concentrations or expression levels, tissues in which they were expressed, methodological approaches, and more. The preliminary form of the data extraction template aimed to collect essential information for use in an evidence synthesis, specific to each research question. Pilot screening revealed an even greater number of factors relevant to the scope of this review, and particularly helpful for the Risk-of-Bias assessment of individual studies. To interpret this diversity, the preliminary data-extraction template was updated extensively for each PO statement in the second revision of the protocol (Panagiotidis, 2022). To improve clarity, the data-extraction template has been slightly amended, and its final version can be viewed in Appendix S4.

Piloting of the data-extraction template was performed independently by two evaluators (K.P. and A.B.) using a handful of eligible studies. Discrepancies were resolved by discussion between these evaluators, where individual piloting activities of the data-extraction template were compared, and conflicts were addressed. Following amendments, further piloting of the data-extraction template was carried out by a single evaluator (K.P.) using nine eligible studies (three studies per outcome). The piloting outcomes of the data-extraction template can be seen in the supplementary material of the second protocol (Panagiotidis, 2022). Following piloting, data extraction of all eligible studies was conducted by a single evaluator (K.P.).

**2.4.3. *Risk-of-Bias assessment***

Inadequacies in the analysis or experimental design of individual studies can lead to incorrect assumptions about the origin and synthesis of sex hormones in molluscan tissues. Critical appraisal tools can be used to assess the internal validity of studies through selection bias, detection bias (reliability of outcome measurements), performance bias, etc. (Martin *et al*., 2021). Many Risk-of-Bias (RoB) tools have been created for chemical studies involving molluscs, but none has assessed the internal validity of studies claiming endogenous synthesis of hormones in those organisms. For the purposes of this systematic evidence map, a tailor-made RoB tool was developed to assess the quality of evidence on the presence of hormones, hormone receptors and hormone-metabolising enzymes in molluscan tissues. The tool consists of a series of criteria that aimed to evaluate the available evidence for each of the three PO statements. Included studies were assessed on both internal validity and study design criteria. Some of the criteria incorporated in the RoB tool were obtained from the ARRIVE guidelines 2.0 (Percie du Sert *et al*., 2020*a*,*b*), which were specifically created to ensure transparency and thorough reporting in studies describing animal research.

The preliminary RoB tool was amended upon receiving feedback from reviewers, and further amended after uploading the second version of the protocol on Zenodo (Panagiotidis, 2022). The amended tool follows a more thorough approach of assessing individual studies and is meant to be used in accordance with the updated RoB guidelines. The guidelines consist of detailed information on how a study was coded in different case scenarios. The scoring method was altered from “Fulfilled”, “Partially fulfilled”, “Not fulfilled” and “Not determined” in the draft protocol (Panagiotidis, 2021), to “Definitely low risk”, “Probably low risk”, “Probably high risk” and “Definitely high risk” in the second protocol (Panagiotidis, 2022). Individual studies were assessed for RoB based on internal validity and study design criteria. Internal validity criteria were created individually for each PO statement based on peer-reviewed literature. However, reported outcomes across studies varied significantly and a one-fits-all approach was difficult to implement. Therefore, included studies were assessed for RoB either fully (based on internal validity and study design criteria) or partly (solely based on study design criteria). Specifically, two types of tailor-made RoB assessments were designed for this purpose:

- **RoB Assessment A** (internal validity and study design criteria):

Eligible studies must provide clear information that ensures an appropriate fit to the research question. Thus, the aims of studies to be assessed with RoB Assessment A must have focused on the investigation of activity, function and/or mechanism of action (MOA) of hormones/ receptors/steroidogenesis-related genes in molluscs. Consequently, the study objectives should adhere to a methodology considered appropriate for an internal validity assessment. Studies that did not employ appropriate methodology but still attempted to draw conclusions on the activity/function/MOA of the outcomes of interest were marked eligible for RoB Assessment A. Studies that used multiple methods to examine a single outcome were assessed for the same outcome independently, based on the methodology implemented. For example, if a study used both a GC-MS and a radioimmunoassay (RIA) approach to examine the presence of steroids in a mollusc, the study was assessed for RoB twice in respect to each technique.

- **RoB Assessment B** (study design criteria only):

RoB Assessment B was used for studies that did not fit within the scope of RoB Assessment A, for example, ecotoxicological assessments investigating the downstream effects of pharmaceuticals by looking at changes in hormone concentrations or gene expression levels (of receptors or enzymes). If the study did not aim to determine (or attempt to comment on) the activity/function/MOA of an outcome of interest, it was assessed solely on study design criteria.

Studies that provided ill-defined detail about the reported outcomes were marked as ‘Unclear’, which is equivalent to a ‘Probably high risk’ score. In cases where a single study addressed more than one outcome (e.g. hormones and hormone receptors), it was assessed independently with respect to the PO statement. Every study assessed for RoB received a final summary score: namely Level 1, Level 2 or Level 3. The Level 1 score represents studies of lower RoB, Level 2 score represents studies with moderate RoB, whereas a Level 3 score represents studies with higher RoB (see RoB summary score tab in Appendix S5 for full information on the scoring systems).

RoB Assessment A studies rated as Level 1 must have received a score of “Definitely low risk” or “Probably low risk” for all internal validity criteria, and a score of “Definitely low risk” or “Probably low risk” for the majority of study design criteria. RoB Assessment A rated as Level 2 were those that met neither Level 1 nor Level 3 scoring criteria (see RoB summary score tab in Appendix S5). Studies rated as Level 3 must have received “Definitely high risk” or “Probably high risk” scores for all internal validity criteria and for most of the study design criteria. Eligible studies for RoB Assessment B were assigned a Level 1 score if all their study design criteria were rated as either “Definitely low risk of bias” or “Probably low risk of bias”. Studies that met neither Level 1 nor Level 3 criteria were assigned a Level 2 score, whereas studies rated with a Level 3 received a “Definitely high risk” or “Probably high risk” for all study design criteria (see Appendix S5). The idea for the level system used was obtained from the OHAT Approach for Systematic Review and Evidence Integration (National Institute of Environmental Health Sciences, 2015), and was adjusted to meet our outcome-specific criteria.

Initial piloting of the RoB tool was performed independently by two evaluators as described in Section 2.4.2. Following initial amendments, the RoB tool was piloted using the nine eligible studies that were used in the data-extraction template. Both the RoB tool and RoB guidelines were additionally amended after publishing the second protocol (Panagiotidis, 2022). These changes are described in detail in the Appendix S2.

1. **OUTCOMES TO BE ANALYSED**

The primary outcome of this systematic evidence map was the extensive synthesis of an evidence report for collected data, in respect to each PO statement and for the overall data. A thorough synthesis of collected evidence was carried out for each PO, highlighting key findings for different molluscan classes. The RoB assessment served as a primary outcome for this review. The assessment showcases multiple levels of reliability for the collected evidence, based on our extensive list of tailor-made RoB guidelines and case scenarios for how a study was coded. The secondary outcome of this systematic map was to identify the knowledge gaps that exist within our current understanding of molluscan endocrinology. These were highlighted as part of a comparative evidence report which thoroughly evaluated the results obtained from the systematic data mapping.

1. **DATA ANALYSIS**

In addition to an in-depth evidence report, the data collated for each outcome were visually summarised using the data visualisation software *Tableau*. Visual comparisons in the form of graphs and summary tables aimed to highlight relationships amongst the outcomes of interest (e.g. hormones, hormone receptors, hormone-metabolising enzymes) with the other criteria included in the data-extraction template (e.g. tissue observed, changes in expression due to an intervention, evidence of activity, etc.). As discussed in the draft protocol (Panagiotidis, 2021), it was expected that experimental designs across studies and PO would vary, as did the species examined in each study. In the field of environmental sciences, good quantitative data are often rare and methodological details are usually insufficiently reported (Haddaway & Verhoeven, 2015). Thus, obtaining a quantitative answer to our research questions using meta-analysis was not feasible.

1. **META-BIASES**

In the absence of a meta-analysis, statistical methods for detecting meta-biases in the evidence report were not possible to implement. To avoid reporting bias, reviewers were welcomed to access both versions of the protocol on the open-access repository Zenodo and compare the reporting outcomes with those of the final manuscript. Any changes made in the reported outcomes following the submission of this protocol are explicitly stated in the final manuscript.

1. **STRENGTH OF EVIDENCE**

The COSTER (Whaley *et al*., 2020) and PRISMA-P (Shamseer *et al*., 2015) recommendations for use of a strength-of-evidence assessment are primarily directed towards systematic reviews in the fields of clinical medicine or environmental health. Hence, the GRADE approach (Guyatt *et al*., 2011) or an interpretation of such an approach, was difficult to implement in the context of the present work. Instead, the collected data were arranged systematically according to factors including mollusc class, sex, life stage and species name.

1. **REPORTING**

The systematic evidence map adhered to the PRISMA-P (Preferred Reporting Items for Systematic review and Meta-Analysis Protocols) 2015 checklist (Shamseer *et al*., 2015) in consideration with the COSTER guidelines (Whaley *et al*., 2020) and is incorporated into the doctoral thesis of K.P. It is also included in the present article.

1. **REFERENCES**

Abril, J. F., & Castellano, S. (2019). *Genome Annotation* (S. Ranganathan, M. Gribskov, K. Nakai, & C. B. T.-E. of B. and C. B. Schönbach (eds.); pp. 195–209). Academic Press. https://doi.org/https://doi.org/10.1016/B978-0-12-809633-8.20226-4

Adema, C. M., Hillier, L. D. W., Jones, C. S., Loker, E. S., Knight, M., Minx, P., Oliveira, G., Raghavan, N., Shedlock, A., Do Amaral, L. R., Arican-Goktas, H. D., Assis, J. G., Baba, E. H., Baron, O. L., Bayne, C. J., Bickham-Wright, U., Biggar, K. K., Blouin, M., Bonning, B. C., … Wilson, R. K. (2017). Whole genome analysis of a schistosomiasis-transmitting freshwater snail. *Nature Communications*, *8*(May). https://doi.org/10.1038/ncomms15451

André, A., Ruivo, R., Fonseca, E., Froufe, E., Castro, L. F. C., & Santos, M. M. (2019). The retinoic acid receptor (RAR) in molluscs: Function, evolution and endocrine disruption insights. *Aquatic Toxicology*, *208*(January), 80–89. https://doi.org/10.1016/j.aquatox.2019.01.002

Baynes, A., Montagut Pino, G., Duong, G. H., Lockyer, A. E., McDougall, C., Jobling, S., & Routledge, E. J. (2019). Early embryonic exposure of freshwater gastropods to pharmaceutical 5-alpha-reductase inhibitors results in a surprising open-coiled “banana-shaped” shell. *Scientific Reports*, *9*(1), 1–12. https://doi.org/10.1038/s41598-019-52850-x

Blaber, S. J. M. (1970). The occurrence of a penis-like outgrowth behind the right tentacle in spent females of Nucella lapillus (L.). *Journal of Molluscan Studies*, *39*(2–3), 231–233. https://doi.org/10.1093/oxfordjournals.mollus.a065097

Bodofsky, S., Koitz, F., Wightman, B., & Baldwin, W. (2017). Nuclear Receptors in Animal Development. *Nuclear Receptor Research*, *4*, 33. https://doi.org/10.11131/2017/101305

Cuvillier-Hot, V. & Lenoir, A. (2020). Invertebrates facing environmental contamination by endocrine disruptors: Novel evidences and recent insights. *Molecular and Cellular Endocrinology* **504**, 110712.

Fodor, I., Urbán, P., Scott, A. P., & Pirger, Z. (2020). A critical evaluation of some of the recent so-called ‘evidence’ for the involvement of vertebrate-type sex steroids in the reproduction of mollusks. *Molecular and Cellular Endocrinology*, *516*(May). https://doi.org/10.1016/j.mce.2020.110949

Fonseca, E., Ruivo, R., Borges, D., Franco, J. N., Santos, M. M., & Castro, L. F. C. (2020). Of retinoids and organotins: The evolution of the retinoid x receptor in metazoa. *Biomolecules*, *10*(4), 1–16. https://doi.org/10.3390/biom10040594

Gibbs, P. E., Bryan, G. W., Pascoe, P. L., & Burt, G. R. (1987). The use of the dog-whelk, Nucella lapillus, as an indicator of tributyltin (TBT) contamination. *J. Mar. Biol. Ass. U.K*, *67*, 507–523. https://doi.org/10.1017/S0025315400027260

Giulianelli, S., Primost, M. A., Lanari, C., & Bigatti, G. (2020). *RXR Expression in Marine Gastropods with Different Sensitivity to Imposex Development*. *Scientific Reports* 10, 1–8. https://doi.org/10.1038/s41598-020-66402-1

Gust, M., Vulliet, E., Giroud, B., Garnier, F., Couturier, S., Garric, J., & Buronfosse, T. (2010). Development, validation and comparison of LC-MS/MS and RIA methods for quantification of vertebrates-like sex-steroids in prosobranch molluscs. *Journal of Chromatography B: Analytical Technologies in the Biomedical and Life Sciences*, *878*(19), 1487–1492. https://doi.org/10.1016/j.jchromb.2010.03.046

Gutierrez-Mazariegos, J., Nadendla, E. K., Lima, D., Pierzchalski, K., Jones, J. W., Kane, M., Nishikawa, J. I., Hiromori, Y., Nakanishi, T., Santos, M. M., Castro, L. F. C., Bourguet, W., Schubert, M., & Laudet, V. (2014). A mollusk retinoic acid receptor (RAR) ortholog sheds light on the evolution of ligand binding. *Endocrinology*, *155*(11), 4275–4286. https://doi.org/10.1210/en.2014-1181

Guyatt, G., Oxman, A. D., Akl, E. A., Kunz, R., Vist, G., Brozek, J., Norris, S., Falck-Ytter, Y., Glasziou, P., Debeer, H., Jaeschke, R., Rind, D., Meerpohl, J., Dahm, P., & Schünemann, H. J. (2011). GRADE guidelines: 1. Introduction - GRADE evidence profiles and summary of findings tables. *Journal of Clinical Epidemiology*, *64*(4), 383–394. https://doi.org/10.1016/j.jclinepi.2010.04.026

Haddaway, N. R., & Verhoeven, J. T. A. (2015). Poor methodological detail precludes experimental repeatability and hampers synthesis in ecology. *Ecology and Evolution*, *5*(19), 4451–4454. https://doi.org/10.1002/ece3.1722

Häggström, M., & Richfield, D. (2014). Diagram of the pathways of human steroidogenesis. *WikiJournal of Medicine*, *1*(1), 1. https://doi.org/10.15347/wjm/2014.005

Handberg-Thorsager, M., Gutierrez-Mazariegos, J., Arold, S. T., Kumar Nadendla, E., Bertucci, P. Y., Germain, P., Tomançak, P., Pierzchalski, K., Jones, J. W., Albalat, R., Kane, M. A., Bourguet, W., Laudet, V., Arendt, D., & Schubert, M. (2018). The ancestral retinoic acid receptor was a low-affinity sensor triggering neuronal differentiation. *Science Advances,* 4(10), eaao1261. https://doi.org/10.1126/sciadv.aao1261.

Horiguchi, T. (2017). Mode of Action of Organotins to Induce the Development of Imposex in Gastropods, Focusing on Steroid and the Retinoid X Receptor Activation Hypotheses. In *Biological Effects by Organotins* (eds. Horiguchi, T.)(pp. 199–219). Springer Tokyo. https://doi.org/10.1007/978-4-431-56451-5_9

Horiguchi, T. & Ohta, Y. (2020). A critical review of sex steroid hormones and the induction mechanisms of imposex in gastropod mollusks. In *Advances in Invertebrate (Neuro)Endocrinology* (1st ed., pp. 30). Apple Academic Press.

Iguchi, T., Katsu, Y., Horiguchi, T., Watanabe, H., Blumberg, B., & Ohta, Y. (2007). Endocrine disrupting organotin compounds are potent inducers of imposex in gastropods and adipogenesis in vertebrates. *Molecular & Cellular Toxicology*, *3*(1), 1–10.

Kaur, S., Baynes, A., Lockyer, A. E., & Routledge, E. J. (2016). Steroid androgen exposure during development has no effect on reproductive physiology of *Biomphalaria glabrata*. *PLoS ONE*, 11, e0159852. https://doi.org/10.1371/journal.pone.0159852

Kaur, S., Jobling, S., Jones, C. S., Noble, L. R., Routledge, E. J., & Lockyer, A. E. (2015b). The nuclear receptors of Biomphalaria glabrata and Lottia gigantea:Implications for developing new model organisms. *PLoS ONE*, *10*(4), e0121259. https://doi.org/10.1371/journal.pone.0121259

Kin, R., Kam, T., Deng, Y., Chen, Y., & Zhao, H. (2012). Retinoic acid synthesis and functions in early embryonic development. *Cell & Bioscience* 2, 11. https://doi.org/10.1186/2045-3701-2-11

Kohl, C., Mcintosh, E. J., Unger, S., Haddaway, N. R., Kecke, S., Schiemann, J., & Wilhelm, R. (2018). Correction to: Online tools supporting the conduct and reporting of systematic reviews and systematic maps: A case study on CADIMA and review of existing tools (Environmental Evidence (2018) 7 (8) DOI: 10.1186/s13750-018-0115-5). *Environmental Evidence*, *7*(1), 13750. https://doi.org/10.1186/s13750-018-0124-4

Krasowski, M. D., Drees, D., Morris, C. S., Maakestad, J., Blau, J. L., & Ekins, S. (2014). Cross-reactivity of steroid hormone immunoassays: Clinical significance and two-dimensional molecular similarity prediction. *BMC Clinical Pathology*, *14*(1), 1–13. https://doi.org/10.1186/1472-6890-14-33

Lafont, R. (1991). Reverse endocrinology, or “hormones” seeking functions. In *Insect Biochemistry* (Vol. 21, Issue 7, pp. 697–721). Pergamon. https://doi.org/10.1016/0020-1790(91)90112-R

Lafont, R. & Mathieu, M. (2007). Steroids in aquatic invertebrates. In *Ecotoxicology* (Vol. 16, Issue 1, pp. 109–130). https://doi.org/10.1007/s10646-006-0113-1

Lecroisey, C., Laudet, V. & Schubert, M. (2012). The cephalochordate amphioxus: a key to reveal the secrets of nuclear receptor evolution. *Briefings in Functional Genomics*, 11(2), 156–166.

Lehoux, J. G., & Sandor, T. (1970). The occurrence of steroids and steroid metabolizing enzyme systems in invertebrates. A review. *Steroids*, *16*(C), 141–171. https://doi.org/10.1016/S0039-128X(70)80102-7

Li, J., Biswas, M. G., Chao, A., Russell, D. W., & Chory, J. (1997). Conservation of function between mammalian and plant steroid 5-reductases. *Proceedings of the National Academy of Sciences of the United States of America*, 94(8), pp.3554-3559.

Li, J., & Chory, J. (1999). Brassinosteroid actions in plants. Journal of Experimental Botany, 50(332), 275–282. https://doi.org/10.1093/jxb/50.332.275

Markov, G. V, Gutierrez-Mazariegos, J., Pitrat, D., Billas, I. M. L., Bonneton, F., Moras, D., Hasserodt, J., Lecointre, G., & Laudet, V. (2017). Origin of an ancient hormone/receptor couple revealed by resurrection of an ancestral estrogen. Science Advances, 3(3), 1–14. https://doi.org/10.1126/sciadv.1601778

Martin, O., Scholze, M., Ermler, S., McPhie, J., Bopp, S. K., Kienzler, A., Parissis, N., & Kortenkamp, A. (2021). Ten years of research on synergisms and antagonisms in chemical mixtures: A systematic review and quantitative reappraisal of mixture studies. *Environment International*, *146*(June 2020), 106206. https://doi.org/10.1016/j.envint.2020.106206

Miglioli, A., Canesi, L., Gomes, I. D. L., Schubert, M., & Dumollard, R. (2021). Nuclear Receptors and Development of Marine Invertebrates. *Genes*, *12*(1), 83. https://doi.org/10.3390/genes12010083

National Institute of Environmental Health Sciences (2015). *Handbook for Conducting a Literature-Based Health Assessment Using OHAT Approach for Systematic Review and Evidence Integration*. 1–94. http://ntp.niehs.nih.gov/ntp/ohat/pubs/handbookjan2015_508.pdf

Ni, J., Zeng, Z., & Ke, C. (2013). *Sex steroid levels and expression patterns of estrogen receptor gene in the oyster Crassostrea angulata during reproductive cycle*. Aquaculture. https://doi.org/10.1016/j.aquaculture.2012.11.023

Niwa, R., Sakudoh, T., Namiki, T., Saida, K., Fujimoto, Y., & Kataoka, H. (2005). The ecdysteroidogenic P450 Cyp302a1/disembodied from the silkworm, Bombyx mori, is transcriptionally regulated by prothoracicotropic hormone. *Insect Molecular Biology*, *14*(5), 563–571. https://doi.org/10.1111/j.1365-2583.2005.00587.x

Niwa, Ryusuke, Matsuda, T., Yoshiyama, T., Namiki, T., Mita, K., Fujimoto, Y., & Kataoka, H. (2004). CYP306A1, a cytochrome P450 enzyme, is essential for ecdysteroid biosynthesis in the prothoracic glands of Bombyx and Drosophila. *Journal of Biological Chemistry*, *279*(34), 35942–35949. https://doi.org/10.1074/jbc.M404514200

Niwa, Ryusuke, & Niwa, Y. S. (2014). Enzymes for ecdysteroid biosynthesis: Their biological functions in insects and beyond. *Bioscience, Biotechnology and Biochemistry*, *78*(8), 1283–1292. https://doi.org/10.1080/09168451.2014.942250

*Norman, A. W. & Litwack, G. (1997*a*). General Considerations of Hormones. In A. W. Norman & G. Litwack (Eds.), In *Hormones* (2nd ed., pp. 1-46). Academic Press.

Oehlmann, J., Di Benedetto, P., Tillmann, M., Duft, M., Oetken, M., & Schulte-Oehlmann, U. (2007). Endocrine disruption in prosobranch molluscs: Evidence and ecological relevance. In *Ecotoxicology* (Vol. 16, Issue 1, pp. 29–43). https://doi.org/10.1007/s10646-006-0109-x

Ouzzani, M., Hammady, H., Fedorowicz, Z., & Elmagarmid, A. (2016). Rayyan-a web and mobile app for systematic reviews. *Systematic Reviews*, *5*(1), 1–10. https://doi.org/10.1186/s13643-016-0384-4

Panagiotidis, K. (2021). *Protocol for a systematic evidence map of sex hormone biosynthesis in Mollusca*. https://doi.org/10.5281/ZENODO.4693859

Panagiotidis, K. (2022). *Protocol for a systematic evidence map of sex hormone biosynthesis in Mollusca*. https://doi.org/10.5281/ZENODO.7061510

Percie Du Sert, N., Ahluwaliaid, A., Alamid, S., Aveyid, M. T., Baker, M., Browneid, W. J., Clarkid, A., Cuthillid, I. C., Dirnaglid, U., Emerson, M., Garnerid, P., Holgate, S. T., Howellsid, D. W., Hurst, V., Karpid, N. A., et al. (2020*a*). Reporting animal research: Explanation and elaboration for the ARRIVE guidelines 2.0. PLoS Biology 18, e300041.

Percie Du Sert, N., Hurst, V., Ahluwalia, A., Alam, S., Avey, M. T., Baker, M., Browne, W. J., Clark, A., Cuthill, I. C., Dirnagl, U., Emerson, M., Garner, P., Holgate, S. T., Howells, D. W., Karp, et al. (2020*b*). The arrive guidelines 2.0: Updated guidelines for reporting animal research. *PLoS Biology* **18**, 1–12.

Petryk, A., Warren, J. T., Marqués, G., Jarcho, M. P., Gilbert, L. I., Kahler, J., Parvy, J. P., Li, Y., Dauphin-Villemant, C., & O’Connor, M. B. (2003). Shade is the Drosophila P450 enzyme that mediates the hydroxylation of ecdysone to the steroid insect molting hormone 20-hydroxyecdysone. *Proceedings of the National Academy of Sciences of the United States of America*, *100*(SUPPL. 2), 13773–13778. https://doi.org/10.1073/pnas.2336088100

Scott, A. P. (2012). Do mollusks use vertebrate sex steroids as reproductive hormones? Part I: Critical appraisal of the evidence for the presence, biosynthesis and uptake of steroids. *Steroids* **77**, 1450–1468. <https://doi.org/10.1016/j.steroids.2012.08.009>

Shamseer, L., Moher, D., Clarke, M., Ghersi, D., Liberati, A., Petticrew, M., Shekelle, P., Stewart, L. A., Altman, D. G., Booth, A., Chan, A. W., Chang, S., Clifford, T., Dickersin, K., Egger, M., Gøtzsche, P. C., Grimshaw, J. M., Groves, T., Helfand, M., … Whitlock, E. (2015). Preferred reporting items for systematic review and meta-analysis protocols (PRISMA-P) 2015: Elaboration and explanation. In *BMJ (Online)* (Vol. 349). BMJ Publishing Group. https://doi.org/10.1136/bmj.g7647

Takeuchi, T. (2017). Molluscan Genomics: Implications for Biology and Aquaculture. *Current Molecular Biology Reports*, *3*(4), 297–305. https://doi.org/10.1007/s40610-017-0077-3

Thitiphuree, T., Nagasawa, K., & Osada, M. (2019). Molecular identification of steroidogenesis-related genes in scallops and their potential roles in gametogenesis. *Journal of Steroid Biochemistry and Molecular Biology* 186, 22–33. https://doi.org/10.1016/j.jsbmb.2018.09.004

Thornton, J.W. & Need, E. D. C. (2003). Resurrecting the Ancestral Steroid Receptor: Ancient Origin of Estrogen Signaling. *Journal of Chemical Information and Modeling*, *53*(9), 1689–1699.

Tran, T. K. A., MacFarlane, G. R., Kong, R. Y. C., O'Connor, W. A., & Yu, R. M. K. (2016). Potential mechanisms underlying estrogen-induced expression of the molluscan estrogen receptor (ER) gene. *Aquatic Toxicology*, *179*, 82–94. https://doi.org/10.1016/j.aquatox.2016.08.015

Urich, K., & Urich, K. (1994). Sterols and Steroids. In *Comparative Animal Biochemistry* (pp. 624–656). Springer Berlin Heidelberg. https://doi.org/10.1007/978-3-662-06303-3_16

Vogeler, S., Bean, T. P., Lyons, B. P., & Galloway, T. S. (2016). Dynamics of nuclear receptor gene expression during Pacific oyster development. *BMC Development Biology* 16, 33. https://doi.org/10.1186/s12861-016-0129-6

Vogeler, S., Galloway, T. S., Lyons, B. P., & Bean, T. P. (2014). The nuclear receptor gene family in the Pacific oyster, Crassostrea gigas, contains a novel subfamily group. *BMC Genomics* 15, 369. https://doi.org/10.1186/1471-2164-15-369

Warren, J. T., Petryk, A., Marqués, G., Jarcho, M., Parvy, J. P., Dauphin-Villemant, C., O’Connor, M. B., & Gilbert, L. I. (2002). Molecular and biochemical characterization of two P450 enzymes in the ecdysteroidogenic pathway of Drosophila melanogaster. *Proceedings of the National Academy of Sciences of the United States of America*, *99*(17), 11043–11048. https://doi.org/10.1073/pnas.162375799

Warren, J. T., Petryk, A., Marqués, G., Parvy, J. P., Shinoda, T., Itoyama, K., Kobayashi, J., Jarcho, M., Li, Y., O’Connor, M. B., Dauphin-Villemant, C., & Gilbert, L. I. (2004). Phantom encodes the 25-hydroxylase of Drosophila melanogaster and Bombyx mori: A P450 enzyme critical in ecdysone biosynthesis. *Insect Biochemistry and Molecular Biology*, *34*(9), 991–1010. https://doi.org/10.1016/j.ibmb.2004.06.009

Whaley, P., Aiassa, E., Beausoleil, C., Beronius, A., Bilotta, G., Boobis, A., de Vries, R., Hanberg, A., Hoffmann, S., Hunt, N., Kwiatkowski, C. F., Lam, J., Lipworth, S., Martin, O., Randall, N., *et al*. (2020). Recommendations for the conduct of systematic reviews in toxicology and environmental health research (COSTER). *Environment International*, *143*. https://doi.org/10.1016/j.envint.2020.105926

Whitehead, D. L. (1977). Steroids enhance shell regeneration in an aquatic gastropod (biomphalaria glabrata). *Comparative Biochemistry and Physiology. Part C, Comparative*, *58*(2), 137–141. https://doi.org/10.1016/0306-4492(77)90094-6
