# Supplementary material for: Mapping molluscan endocrinology: a systematic and critical appraisal
Source: Biol Rev Camb Philos Soc. 2025 Dec 16;101(2):970–1002. doi: 10.1002/brv.70112 (PMC12965858; doi:10.1002/brv.70112)
Supplement: Supplementary file 2 — Appendix S2. Updates made to the final protocol for the systematic evidence map of hormone biosynthesis in Mollusca. [file BRV-101-970-s002.docx]

Appendix S2. Updates made to the final protocol for the systematic evidence map of hormone biosynthesis in Mollusca

**Mapping Molluscan Endocrinology: A Systematic and Critical Appraisal**

**Authors:** Konstantinos Panagiotidis^1*^, Thomas H. Miller^1^ , Olwenn V. Martin^2^, Alice Baynes^1^ **Affiliation addresses:**

^1^*Environmental Sciences, Departure of Life Sciences, Brunel University London, Kingston Ln, London, Uxbridge UB8 3PH, UK*^2^*Department of Arts and Science, Faculty of Arts & Humanities, University College London, Gower St, London WC1E 6BT, UK*

**Correspondence:** Konstantinos Panagiotidis ([constantinospan@outlook.com](mailto:constantinospan@outlook.com))

1. **TITLE**

To reflect the diverse information collected by our systematic searches (spanning hormones from a range of signalling pathways), the title of the systematic map protocol was changed from “Protocol for a systematic evidence map of sex hormone biosynthesis in Mollusca” to “Protocol for a systematic evidence map of hormone biosynthesis in Mollusca”.

1. **PO STATEMENTS**

In the second version of the draft protocol, the Population, Outcome (PO) statement Mollusca AND Nuclear receptors aimed to answer the question “Which nuclear receptor transcripts have been found to be present in molluscan tissues?”. However, keyword strings for this PO statement spanned multiple types of receptors, outside the nuclear receptor superfamily. For this reason, the PO statement was amended to Mollusca AND Receptors which aimed to answer the more comprehensive question, “What evidence is there for different hormone receptors in molluscan tissues?”.

Similarly the Mollusca AND Steroidogenesis-related genes PO initially aimed to answer the question “Which steroidogenesis-related transcripts have been found to be present in molluscan tissues?”. However, our search strategy comprised key words spanning vertebrate steroidogenesis, insect steroidogenesis and retinoid metabolism. Consequently, a range of biomolecules involved in hormone signalling pathways outside vertebrate steroidogenesis were captured in the search. Therefore, the Mollusca AND Steroidogensis-related genes PO was amended to the more comprehensive term Mollusca AND Enzymes which answered the question “What evidence is there for different hormone metabolising enzymes in molluscan tissues?”.

1. **ELIGIBILITY CRITERIA**

PO statement Mollusca AND Hormones

**Inclusion criteria:**The systematic searches identified a range of thyroid hormones that were not initially part of the eligibility criteria. Thus, the eligibility criteria for Mollusca AND Hormones PO were updated to include the presence of thyroid hormones in molluscs.

PO statement Mollusca AND Receptors

**Inclusion criteria:**

To make the systematic map more comprehensive, data were collected on all types of receptors found in molluscs. Thus, the term ‘Nuclear receptors’ was replaced by the term ‘Receptors’.

**Exclusion criteria:**The term ‘neurohormone receptors’ was added to the exclusion criteria as neurohormones, and thus receptors involved in neurohormonal signalling, are outside the scope of this review and were excluded from the initial systematic searches.

PO statement Mollusca AND Enzymes

**Inclusion criteria:**The term “plant steroidogenesis” was deleted as they do not represent invertebrate organisms.

1. **DATA-EXTRACTION TEMPLATE**

Upon submission of the second version of the protocol (Panagiotidis, 2022) additional changes were made to the data-extraction template (see Appendix S4) to facilitate comprehensive data visualisation, evidence synthesis and the Risk of Bias (RoB) tool. The amendments made were as follows:

**PO Statement: Mollusca AND Hormones**

Criteria included:

- “Pathway Involved”
- “Receptor(s) that interacts”
- “Notes”

Criteria removed:

- “Method validation”
- “SOP”

Criteria amended:

- “Sex hormone/sterol measured” was separated into “Hormone name”, “Hormone abbreviation” and “Hormone measured”.
- “Change of concentration due to an intervention” was separated into “Intervention used?”, “Type of intervention” and “Effect of intervention.”

**PO Statement: “Mollusca AND Receptors”**

Criteria included:

- “Vertebrate/invertebrate-type receptor”
- “Receptor type”
- “Method type (group)”. “Group” was included to distinguish other method type entries and to facilitate data visualisation.
- “Method type”

Criteria amended:

- “NR name” was separated into “Receptor name (reported on paper)”, “Receptor gene abbreviation (reported on paper)”, “Receptor gene abbreviation (nomenclature) and “Receptor name (nomenclature)”.
- “Evidence of NR activity” was changed to “Evidence of receptor’s activity”
- “Change of expression due to intervention” was split into “Type of intervention” and “Details of activity after intervention”.

**PO statement: “Mollusca AND Enzymes”**

Criteria included:

- “Pathway involved”
- “Main hormones the enzyme interacts with”
- “Retinoids the enzyme interacts with”
- “Other known activity”

Criteria amended:

- “Gene name” was separated into “Gene abbreviation (reported on paper)”, “Gene abbreviation (nomenclature) and “Enzyme name (nomenclature)”
- “Change of expression due to an intervention” was split into “Type of intervention” and “Change reported due to intervention”.
- “Evidence of activity/function of the steroidogenesis-related gene” was split into “Details on the evidence of activity/function of the hormone-metabolising enzyme (e.g. up- or downregulated expression, conversion to a steroid)?” and “Evidence of activity (Y/N)”.

1. **RISK-OF-BIAS TOOL AND RISK-OF-BIAS GUIDELINES:**

Upon revision of the RoB tool and its accompanied guidelines, further amendments were needed to ensure accuracy and un-biased appraisal of the collected evidence (see Appendix S5, Questionnaire tab). Changes to the RoB guidelines (and respective RoB tool criteria) were made as follows:

**Assessment A: internal validity criteria**

**PO Mollusca AND Hormones**

**Criteria amended:**

1. Identification of hormones in molluscs:

- **“***Did the analysis include: (1) samples spiked with internal standard/positive control, (2) LOD, (3) method validation, (4) recovery or reproducibility?”* was replaced by “*Did the analysis include details on: (1) accuracy (use of positive quality contols (QC) or samples spiked with internal standard); (2) linearity (limit of detection); (3) precision (repeatability = intra assay/inter assay OR reproducibility = inter-laboratory assay)?”*

The scoring criteria were updated accordingly and now include a set of generally accepted method-validation standards by which chemical techniques were assessed. Similarly, the amended scoring criteria now specify how inadequacies in the extraction and purification techniques were assessed.

2. Verification of MOA (mechanism of action) of hormones:

Since the amended PO “Mollusca AND Receptors” covers a range of receptors outside the nuclear receptor superfamily, it was necessary to state that not all receptors bind to hormones. Therefore, “Note 3” in the Risk of Bias guidelines was added to state that only certain types of hormones known to bind to receptors will be assessed for investigations into their MOA.

**PO Mollusca AND Receptors**

**Criteria amended:**

1. Verification of expression of receptors in molluscs:

- *“Which method was used to determine expression/presence of the NR in molluscan tissues?”* was replaced by *“Did the method examine temporal and/or spatial distribution of the receptor’s DNA or RNA?”*

The scoring criteria in the Risk of Bias guidelines were updated accordingly, specifying the difference between molecular techniques that measured protein levels compared to DNA/RNA expression levels.

2. Comparison of nuclear receptor affiliation (sequence similarity analysis) using the conserved DNA binding domain (DBD) and ligand binding domain (LBD) with other invertebrate and vertebrate species:

Since the “Mollusca AND Receptors” PO spans a range of receptors outside the nuclear receptor superfamily, a statement was included to state that only nuclear receptors, will be assessed for this criterion.

**PO Mollusca AND Enzymes**

**Criteria amended:**

1. Verification of expression of hormone-metabolising enzymes in molluscs:

- “*Which method was used to determine expression/presence of the steroidogenesis-related gene in molluscan tissues?*” was replaced by “*Did the method examine temporal and/or spatial distribution of the gene’s DNA or RNA, that encodes the hormone metabolising enzyme? Was there evidence of activity for the hormone-metabolising enzyme in molluscs (e.g. up - or down regulated expression of gene transcripts etc.)?*

Similarly to the changes made for “Mollusca AND Receptors”, the scoring criteria in the Risk of Bias guidelines were updated accordingly, specifying the difference between molecular techniques that measured protein levels compared to DNA/RNA expression levels.

**Assessment B: Study design criteria**

**Criteria amended:**

2. Within-study or between-study study repetition:

- “*Did the study attempt to repeat findings “within-study” or “between-study?*” was replaced by “*Was the number of biological replicates (n) clearly defined?”, “Did the study combine their biological measurements during analysis?” and “Did the study perform an independent verification experiment?”*

In addition, the scoring criteria for this question were changed from *“Probably low risk of bias”* and *“Probably high risk of bias”* to *“Definitely low risk of bias”, “Probably low risk of bias”, “Probably high risk of bias”* and *“Definitely high risk of bias*”. Thus, within-study or between-study repetition was considered any independent verification experiment. Additional points for lower-RoB scores were obtained based on information about biological measurements.

3. Statistics

- “*How many of the criteria mentioned in the RoB guidelines were used in the reporting?” and “If more than one statistical analysis was used, were the results consistent across analyses (if applicable)?”* were replaced by *“Did the study include at least two of the statistical characteristics included in ARRIVA guidelines 2.0?”*

The scoring criteria changed from *“Definitely low risk of bias”, “Probably low risk of bias”, “Probably high risk of bias”* and *“Definitely high risk of bias*” to either *“Probably low risk of bias”* or *“Probably high risk of bias”.*
