# Supplementary material for: Mapping molluscan endocrinology: a systematic and critical appraisal
Source: Biol Rev Camb Philos Soc. 2025 Dec 16;101(2):970–1002. doi: 10.1002/brv.70112 (PMC12965858; doi:10.1002/brv.70112)
Supplement: Supplementary file 3 — Appendix S3. Pilot search for a systematic evidence map of hormone biosynthesis in Mollusca. [file BRV-101-970-s001.docx]

Appendix S3: Pilot Search for a systematic evidence map of hormone biosynthesis in Mollusca

**Mapping Molluscan Endocrinology: A Systematic and Critical Appraisal**

**Authors:** Konstantinos Panagiotidis^1*^, Thomas H. Miller^1^ , Olwenn V. Martin^2^, Alice Baynes^1^ **Affiliation addresses:**

^1^*Environmental Sciences, Departure of Life Sciences, Brunel University London, Kingston Ln, London, Uxbridge UB8 3PH, UK*^2^*Department of Arts and Science, Faculty of Arts & Humanities, University College London, Gower St, London WC1E 6BT, UK*

**Correspondence:** Konstantinos Panagiotidis ([constantinospan@outlook.com](mailto:constantinospan@outlook.com))

**Table S3.1.** Search strings used for the Mollusca AND Hormones PO search across different databases (*PubMed*, *Web of Science*, *Scopus*). Number of hits represents the number of retrieved papers on the day the search was performed (20/09/21).

| **Population AND Outcome** | **Keyword strings** | **Database** | **Hits** |
| --- | --- | --- | --- |
| Mollusca AND Hormones | (Mollusc OR molluscs OR mollusca OR Mollusk OR Mollusks OR Bivalves OR Bivalve OR Bivalvia OR Gastropod OR Gastropods OR Gastropoda OR cephalopod OR Cephalopoda OR cephalopods OR polyplacophor OR polyplacophora OR polyplacophores OR scaphopod OR scaphopods OR scaphopoda OR aplacophor OR aplacophors OR aplacophora OR monoplacophor OR monoplacophors OR monoplacophora OR Oyster OR Oysters OR mussel OR mussels OR squid OR squids OR octopus OR octopuses OR chiton OR chitons OR Snail OR Snails) AND (hormone OR hormones OR steroid OR steroids OR steroidogenesis OR sterol OR sterols OR hormonogenesis OR “juvenile hormone” OR “juvenile hormones” OR “methyl farnesoate” OR estrone OR "estradiol-17β" OR "17-beta-estradiol" OR estradiol OR estriol OR estetrol OR "2-Hydroxyestrone" OR "2-ohe" OR "estra-1,3,5(10)-trien-2,3-diol-17-one" OR "16α-Hydroxyestrone" OR "16α-loh-e1" OR hydroxyestrone OR "estra-1,3,5(10)-triene-3,16α-diol-17-one" OR "2-Hydroxyestradiol" OR “2-lohe2” OR “estra-1,3,5(10)-triene-2,3,17β-triol” OR testosterone OR dehydroepiandrosterone OR dhea OR androstenedione OR androstenediol OR androsterone OR dihydrotestosterone OR dht OR androstanediol OR androstanedione OR progesterone OR pregnenolone OR allopregnanedione OR allopregnanolone OR "17α-Hydroxypregnenolone" OR "17-alpha-Hydroxypregnenolone" OR "17α-Hydroxyprogesterone" OR "17-alpha-Hydroxyprogesterone" OR hydroxyprogesterone OR ecdysone OR “alpha-Ecdysone” OR ecdysterone OR “20-Hydroxyecdysone” OR turkesterone OR “2-deoxyecdysone” OR “2-Deoxy-alpha-ecdysone”) | *PubMed* | 3,594 |
| Mollusca AND Hormones | (Mollusc* OR molluscs OR Mollusk* OR Bivalves OR Bivalv* OR Gastropod* OR Gastropods OR cephalopod* OR polyplacophor* OR scaphopod* OR aplacophor* OR monoplacophor* OR Oyster* OR mussel* OR squid* OR octopus* OR chiton* OR Snail* OR Snails) AND (hormone* OR steroid* OR steroidogenesis OR sterol*OR hormonogenesis OR “juvenile hormone*” OR “methyl farnesoate” OR estrone OR "estradiol-17β" OR "17-beta-estradiol" OR estradiol OR estriol OR estetrol OR "2-Hydroxyestrone" OR "2-ohe" OR "estra-1,3,5(10)-trien-2,3-diol-17-one" OR "16α-Hydroxyestrone" OR "16α-loh-e1" OR hydroxyestrone OR "estra-1,3,5(10)-triene-3,16α-diol-17-one" OR "2-Hydroxyestradiol" OR “2-lohe2” OR “estra-1,3,5(10)-triene-2,3,17β-triol” OR testosterone OR dehydroepiandrosterone OR dhea OR androstenedione OR androstenediol OR androsterone OR dihydrotestosterone OR dht OR androstanediol OR androstanedione OR progesterone OR pregnenolone OR allopregnanedione OR allopregnanolone OR "17α-Hydroxypregnenolone" OR "17-alpha-Hydroxypregnenolone" OR "17α-Hydroxyprogesterone" OR "17-alpha-Hydroxyprogesterone" OR hydroxyprogesterone OR ecdysone OR “alpha-Ecdysone” OR ecdysterone OR “20-Hydroxyecdysone” OR turkesterone OR “2-deoxyecdysone” OR “2-Deoxy-alpha-ecdysone”) | *Web of Science* | 1973 |
| Mollusca AND Hormones | (Mollusc* OR molluscs OR Mollusk* OR Bivalves OR Bivalv* OR Gastropod* OR Gastropods OR cephalopod* OR polyplacophor* OR scaphopod* OR aplacophor* OR monoplacophor* OR Oyster* OR mussel* OR squid* OR octopus* OR chiton* OR Snail* OR Snails) AND (hormone*) | *Scopus* (search 1) | 1957 |
| Mollusca AND Hormones | (Mollusc* OR molluscs OR Mollusk* OR Bivalves OR Bivalv* OR Gastropod* OR Gastropods OR cephalopod* OR polyplacophor* OR scaphopod* OR aplacophor* OR monoplacophor* OR Oyster* OR mussel* OR squid* OR octopus* OR chiton* OR Snail* OR Snails) AND (steroid*) | *Scopus* (search 2) | 1015 |
| Mollusca AND Hormones | (Mollusc* OR molluscs OR Mollusk* OR Bivalves OR Bivalv* OR Gastropod* OR Gastropods OR cephalopod* OR polyplacophor* OR scaphopod* OR aplacophor* OR monoplacophor* OR Oyster* OR mussel* OR squid* OR octopus* OR chiton* OR Snail* OR Snails) AND (steroidogenesis OR sterol* OR hormonogenesis OR {juvenile hormone*} OR {methyl farnesoate} OR estrone OR {estradiol-17β} OR {17-beta-estradiol} OR estradiol OR estriol OR estetrol OR {2-Hydroxyestrone} OR {2-ohe} OR {estra-1,3,5(10)-trien-2,3-diol-17-one} OR {16α-Hydroxyestrone} OR {16α-loh-e1} OR hydroxyestrone OR {estra-1,3,5(10)-triene-3,16α-diol-17-one} OR {2-Hydroxyestradiol} OR {2-lohe2} OR {estra-1,3,5(10)-triene-2,3,17β-triol} OR testosterone OR dehydroepiandrosterone OR dhea OR androstenedione OR androstenediol OR androsterone OR dihydrotestosterone OR dht OR androstanediol OR androstanedione OR progesterone OR pregnenolone OR allopregnanedione OR allopregnanolone OR {17α-Hydroxypregnenolone} OR {17-alpha-Hydroxypregnenolone} OR {17α-Hydroxyprogesterone} OR {17-alpha-Hydroxyprogesterone} OR hydroxyprogesterone OR ecdysone OR {alpha-Ecdysone} OR ecdysterone OR {20-Hydroxyecdysone} OR turkesterone OR {2-deoxyecdysone} OR {2-Deoxy-alpha-ecdysone}) | *Scopus* (search 3) | 1382 |

**Table S3.2.** Search strings used for Mollusca AND Receptors PO search across different databases (*PubMed*, *Web of Science*, *Scopus*). Number of hits represents the number of retrieved papers on the day the search was performed (20/09/21).

| **Population AND Outcome** | **Keyword strings** | **Database** | **Hits** |
| --- | --- | --- | --- |
| Mollusca AND Receptors | (Mollusc OR molluscs OR mollusca OR Mollusk OR Mollusks OR Bivalves OR Bivalve OR Bivalvia OR Gastropod OR Gastropods OR Gastropoda OR cephalopod OR Cephalopoda OR cephalopods OR polyplacophor OR polyplacophora OR polyplacophores OR scaphopod OR scaphopods OR scaphopoda OR aplacophor OR aplacophors OR aplacophora OR monoplacophor OR monoplacophors OR monoplacophora OR Oyster OR Oysters OR mussel OR mussels OR squid OR squids OR octopus OR octopuses OR chiton OR chitons OR Snail OR Snails) AND (“Nuclear receptor” OR “nuclear receptors” OR “nuclear hormone receptor” OR “nuclear hormone receptors” OR “hormone receptor” OR “hormone receptors” OR “steroid receptor” OR “steroid receptors” OR “retinoid” OR “retinoids” OR “retinoic”) | *PubMed* | 335 |
| Mollusca AND Receptors | (Mollusc* OR molluscs OR Mollusk* OR Bivalves OR Bivalv* OR Gastropod* OR Gastropods OR cephalopod* OR polyplacophor* OR scaphopod* OR aplacophor* OR monoplacophor* OR Oyster* OR mussel* OR squid* OR octopus* OR chiton* OR Snail* OR Snails) AND ("Nuclear receptor*" OR “nuclear hormone receptor*” OR “hormone receptor*” OR “retinoid*” OR “retinoic” OR “steroid receptor*”) | *Web of Science* | 394 |
| Mollusca AND Receptors | (Mollusc* OR molluscs OR Mollusk* OR Bivalves OR Bivalv* OR Gastropod* OR Gastropods OR cephalopod* OR polyplacophor* OR scaphopod* OR aplacophor* OR monoplacophor* OR Oyster* OR mussel* OR squid* OR octopus* OR chiton* OR Snail* OR Snails) AND ("Nuclear receptor*" OR “nuclear hormone receptor*” OR “hormone receptor*” OR retinoid* OR retinoic OR “steroid receptor*”) | *Scopus* | 654 |

**Table S3.2 updates:**

- Truncation (*) was not identified by *PubMed*, and thus additional keywords were included to cover both singular and plural forms of each string.
- Removed “receptor” term, specific nuclear receptor terms as well as the “**AND**” Boolean operator which limited the search using specific nuclear receptor names. Added terms: “nuclear hormone receptor”, “hormone receptor” and “steroid receptor”.

**Table S3.3.** Search strings used for Mollusca AND Enzymes PO search across different databases (*PubMed*, *Web of Science*, *Scopus*). Number of hits represent the number of retrieved papers on the day the search was performed (20/09/21).

| **Population AND Outcome** | **Keyword strings** | **Database** | **Hits** |
| --- | --- | --- | --- |
| Mollusca AND Enzymes | (Mollusc OR molluscs OR mollusca OR Mollusk OR Mollusks OR Bivalves OR Bivalve OR Bivalvia OR Gastropod OR Gastropods OR Gastropoda OR cephalopod OR Cephalopoda OR cephalopods OR polyplacophor OR polyplacophora OR polyplacophors OR scaphopod OR scaphopods OR scaphopoda OR aplacophor OR aplacophors OR aplacophora OR monoplacophor OR monoplacophors OR monoplacophora OR Oyster OR Oysters OR mussel OR mussels OR squid OR squids OR octopus OR octopuses OR chiton OR chitons OR Snail OR Snails) AND (“steroidogenesis-related gene” OR “steroidogenesis-related genes” OR “steroidogenic gene” OR “steroidogenic genes” OR cyp11a OR cyp11a1 OR P450scc OR cyp17a1 OR P450C17 OR cyp11b1 OR P450C11 OR cyp11b2 OR cyp21a2 OR akr1c4 OR hsd3b1 OR hsd3b2 OR hsd11b1 OR hsd11b2 OR hsd17b1 OR hsd17b2 OR hsd17b3 OR hsd17b4 OR hsd17b5 OR hsd17b6 OR hsd17b7 OR hsd17b8 OR hsd17b9 OR hsd17b10 OR hsd17b11 OR hsd17b12 OR hsd17b13 OR hsd17b14 OR akr1c1 OR srd5a1 OR "steroid 5 alpha-reductase 1" OR srd5a2 OR "steroid 5 alpha-reductase 2" OR srd5a3 OR "steroid 5 alpha-reductase 3" OR sult1a1 OR sult1e1 OR sult2a1 OR sult2b1 OR sts OR “steroid sulfatase” OR cyp19 OR DET2 OR CG40050 OR "Dmel\CG40050" OR "CG40050-PA" OR CG12068 OR "Dmel\CG12068" OR "CG12068-PA" OR CG8457 OR Cyp6T3 OR CG10594 OR CYP307A1 OR CG41624 OR CYP307A2 OR CG6578 OR CYP306A1 OR CG12028 OR CYP302A1 OR CG14728 OR CYP315A1 OR CG13478 OR CYP314A1 OR CG12390 OR CRABP OR "Cellular retinoic acid binding protein" OR CRBP OR "cellular retinol binding protein" OR RBP OR "Retinol binding protein" OR SRA6 OR SRA-6 OR "Serpentine receptor class alpha-6" OR TTR OR Transthyretin OR TBPA) | *PubMed* | 133 |
| Mollusca AND Enzymes | (Mollusc* OR molluscs OR Mollusk* OR Bivalves OR Bivalv* OR Gastropod* OR Gastropods OR cephalopod* OR polyplacophor* OR scaphopod* OR aplacophor* OR monoplacophor* OR Oyster* OR mussel* OR squid* OR octopus* OR chiton* OR Snail* OR Snails) AND (“steroidogenesis-related gene*” OR “steroidogenic gene*” OR cyp11a OR cyp11a1 OR P450scc OR cyp17a1 OR P450C17 OR cyp11b1 OR P450C11 OR cyp11b2 OR cyp21a2 OR akr1c4 OR hsd3b1 OR hsd3b2 OR hsd11b1 OR hsd11b2 OR hsd17b1 OR hsd17b2 OR hsd17b3 OR hsd17b4 OR hsd17b5 OR hsd17b6 OR hsd17b7 OR hsd17b8 OR hsd17b9 OR hsd17b10 OR hsd17b11 OR hsd17b12 OR hsd17b13 OR hsd17b14 OR akr1c1 OR srd5a1 OR "steroid 5 alpha-reductase 1" OR srd5a2 OR "steroid 5 alpha-reductase 2" OR srd5a3 OR "steroid 5 alpha-reductase 3" OR sult1a1 OR sult1e1 OR sult2a1 OR sult2b1 OR sts OR “steroid sulfatase” OR cyp19 OR DET2 OR CG40050 OR "Dmel\CG40050" OR "CG40050-PA" OR CG12068 OR "Dmel\CG12068" OR "CG12068-PA" OR CG8457 OR Cyp6T3 OR CG10594 OR CYP307A1 OR CG41624 OR CYP307A2 OR CG6578 OR CYP306A1 OR CG12028 OR CYP302A1 OR CG14728 OR CYP315A1 OR CG13478 OR CYP314A1 OR CG12390 OR CRABP OR "Cellular retinoic acid binding protein" OR CRBP OR "cellular retinol binding protein" OR RBP OR "Retinol binding protein" OR SRA6 OR SRA-6 OR "Serpentine receptor class alpha-6" OR TTR OR Transthyretin OR TBPA) | *Web of Science* | 94 |
| Mollusca AND Enzymes | (Mollusc* OR molluscs OR Mollusk* OR Bivalves OR Bivalv* OR Gastropod* OR Gastropods OR cephalopod* OR polyplacophor* OR scaphopod* OR aplacophor* OR monoplacophor* OR Oyster* OR mussel* OR squid* OR octopus* OR chiton* OR Snail* OR Snails) AND ({steroidogenesis-related gene*} OR {steroidogenic gene*} OR cyp11a OR cyp11a1 OR P450scc OR cyp17a1 OR P450C17 OR cyp11b1 OR P450C11 OR cyp11b2 OR cyp21a2 OR akr1c4 OR hsd3b1 OR hsd3b2 OR hsd11b1 OR hsd11b2 OR hsd17b1 OR hsd17b2 OR hsd17b3 OR hsd17b4 OR hsd17b5 OR hsd17b6 OR hsd17b7 OR hsd17b8 OR hsd17b9 OR hsd17b10 OR hsd17b11 OR hsd17b12 OR hsd17b13 OR hsd17b14 OR akr1c1 OR srd5a1 OR "steroid 5 alpha-reductase 1" OR srd5a2 OR "steroid 5 alpha-reductase 2" OR srd5a3 OR "steroid 5 alpha-reductase 3" OR sult1a1 OR sult1e1 OR sult2a1 OR sult2b1 OR sts OR “steroid sulfatase” OR cyp19 OR DET2 OR CG40050 OR "Dmel\CG40050" OR "CG40050-PA" OR CG12068 OR "Dmel\CG12068" OR "CG12068-PA" OR CG8457 OR Cyp6T3 OR CG10594 OR CYP307A1 OR CG41624 OR CYP307A2 OR CG6578 OR CYP306A1 OR CG12028 OR CYP302A1 OR CG14728 OR CYP315A1 OR CG13478 OR CYP314A1 OR CG12390 OR CRABP OR "Cellular retinoic acid binding protein" OR CRBP OR "cellular retinol binding protein" OR RBP OR "Retinol binding protein" OR SRA6 OR SRA-6 OR "Serpentine receptor class alpha-6" OR TTR OR Transthyretin OR TBPA) | *Scopus* | 125 |
