# Supplementary material for: Mapping molluscan endocrinology: a systematic and critical appraisal
Source: Biol Rev Camb Philos Soc. 2025 Dec 16;101(2):970–1002. doi: 10.1002/brv.70112 (PMC12965858; doi:10.1002/brv.70112)
Supplement: Supplementary file 6 — Appendix S6. Risk‐of‐Bias tool guidelines. [file BRV-101-970-s007.docx]

Appendix S6. Risk-of-Bias tool guidelines for a systematic evidence map of hormone biosynthesis in Mollusca

**Mapping Molluscan Endocrinology: A Systematic and Critical Appraisal**

**Authors:** Konstantinos Panagiotidis^1*^, Thomas H. Miller^1^ , Olwenn V. Martin^2^, Alice Baynes^1^ **Affiliation addresses:**

^1^*Environmental Sciences, Departure of Life Sciences, Brunel University London, Kingston Ln, London, Uxbridge UB8 3PH, UK*^2^*Department of Arts and Science, Faculty of Arts & Humanities, University College London, Gower St, London WC1E 6BT, UK*

**Correspondence:** Konstantinos Panagiotidis ([constantinospan@outlook.com](mailto:constantinospan@outlook.com))

**BACKGROUND INFORMATION**

Individual studies were assessed for Risk-of-Bias (RoB) based on internal validity and study design criteria. Internal validity criteria were created individually for each PO statement based on peer-reviewed literature. However, with variation in reported outcomes across studies, a one-fits-all approach was difficult to implement.

Therefore, included studies were assessed for RoB either fully (based on internal validity and study design criteria) or partly (based on study design criteria alone).

RoB Assessment A (internal validity and study design criteria):

Eligible studies must provide clear information that ensures an appropriate fit to the research question. Thus, the aims of studies to be assessed with RoB Assessment A must focus on the investigation of activity, function and/or mechanism of action (MOA) of hormones/ receptors/hormone-metabolising genes in molluscs. Consequently, the study objectives should adhere to a methodology appropriate for an internal validity assessment. Studies that did not employ appropriate methodology but still attempted to draw conclusions on the activity/function/MOA of the outcomes of interest were also marked eligible for a RoB Assessment A.

Some studies used different methods to examine a single outcome. Such studies were assessed independently, for the same outcome, based on the methodology implemented. For example, if a study used both a Gas Chromatography-Mass Spectrometry (GC-MS) and a radioimmunoassay (RIA) approach to examine the presence of steroids in *Biomphalaria glabrata*, the study was assessed twice, i.e. once for each technique.

RoB Assessment B (study design criteria only):

Studies that did not fit within the scope of RoB Assessment A were assessed as ROB Assessment B based on study design criteria only. For example, ecotoxicological assessments investigating the downstream effects of pharmaceuticals by looking at changes in hormone concentrations or gene expression levels were assessed using RoB Assessment B. If the study did not aim to determine (or attempt to comment on) the activity/function/MOA of an outcome of interest, it was assessed solely on study design criteria.

Studies addressing more than one research outcome:

Where a study addressed more than one outcome (e.g. the presence of hormones and of hormone receptors), it was assessed independently for each PO statement.

**INTERNAL VALIDITY CRITERIA: ASSESSMENT A ONLY**

See Appendix S5 ‘Questionnaire’ tab. Numbering below follows questionnaire numbering.

**Mollusca AND hormones**

1. **Identification of hormones in molluscs**

The quantification of hormones which exist in low concentrations in molluscs can be achieved using a range of techniques including analytical chemistry methods [e.g. GC-MS, Liquid Chromatography-Mass Spectrometry (LC-MS)] or immunoassays [e.g. enzyme-linked immunosorbent assay (ELISA), RIA]. The use of RIA for the measurement of steroid concentrations is considered rapid, inexpensive, and generally exhibits satisfactory precision (Gust *et al*., 2010). However, GC-MS and LC-MS are considered the gold standard for identifying and quantifying steroid hormones (Gust *et al*., 2010). Specifically, the LC-MS method has been shown to exhibit satisfactory precision in identifying low concentrations of steroids (Xu *et al*., 2007), and enables the detection of various compounds or metabolites in a single assay (Gust *et al*., 2010). By contrast, RIA-based methods have been shown to exhibit limited accuracy and specificity in identifying steroids as a result of cross-reactivity or interaction with the organic matrix (in which steroids are present) (Gust *et al*., 2010). Thus, data from studies that implemented immunoassays for the detection of hormones in molluscan tissues should be treated with caution. Due to the low limits of detection necessary to confirm the presence of hormones in molluscan tissues, a sensitive analytical procedure that includes extraction and purification steps should be developed, with adequate levels of recovery and reproducibility (Gust *et al*., 2010). To ensure the accuracy, precision and reproducibility of an analytical procedure, it is essential to employ optimal performance conditions for all steps of the protocol. A set of method characteristics that should be included in validation experiments for several analytical procedures [e.g. ELISA, LC-MS, GC-MS, High-Performance Liquid Chromatography (HPLC)] were identified through the literature (Minic & Zivkovic, 2021; Kim *et al*., 2013; James *et al*., 2019; Singh, 2013) and included in the RoB tool. Methods employed for measuring hormones/sterols in molluscan tissues will thus be assessed on (1) the type of analysis used (chemical or other), (2) the extent of information presented for sample extraction and purification, (3) the extent of information presented on method validation (accuracy, linearity, and precision).

- What type of analysis was used to measure hormone concentrations?
- Is the information about extraction and purification adequate?
- Did the analysis include details on:

(1) Accuracy (use of positive quality controls (QC) or samples spiked with Internal standard)

(2) Linearity (limit of detection),

(3) Precision (repeatability = intra-assay/inter-assay OR reproducibility = inter-laboratory assay)?

| Probably low risk of bias | Measurements of hormones using chemical analysis (e.g. LC-MS, GC-MS, HPLC, etc.) with adequate information about extraction **and** purification steps* which includes details on **at least two** of accuracy, linearity, and precision. |
| --- | --- |
|  | Measurements of hormones using an immunoassay (e.g. RIA, EIA, ELISA) with adequate information about extraction **and** purification steps* which includes details **on all of** accuracy, linearity and precision. |
| Probably high risk of bias | Measurements of hormones using chemical analysis (e.g. LC-MS, GC-MS, HPLC, etc.) without adequate information about extraction **and/or** purification steps*. |
|  | Measurements of hormones using chemical analysis (e.g. LC-MS, GC-MS, HPLC, etc.) which includes details on **only one** or **none** of accuracy, linearity and precision. |
|  | Measurements of hormones using an immunoassay (e.g. RIA, EIA, ELISA) without adequate information about extraction **and/or** purification steps*. |
|  | Measurements of hormones using an immunoassay (e.g. RIA, EIA, ELISA) which includes details **on two or fewer** of accuracy, linearity and precision. |
|  | Measurements of hormones using any other method (not immunoassay or chemical analysis). |
|  | Unclear information. |

*Inadequate information about extraction and purification procedures includes: (1) no detail on sample preparation/extraction; (2) no detail on sample purification. This also applies to studies that have followed and cited methodology by an external research group, unless modifications made during sample preparation, extraction and purification were explicitly stated.

**2. Verification of mechanism of action (MOA) of hormones**

Many hormones are able to bind to their respective receptors (e.g. steroid hormones binding to steroid receptors), which are in turn essential mediators of hormone action. Hormone receptors are activated by the binding of a ligand to influence the expression of target genes (Beato & Klug, 2000), by turning them on or off. Types of hormones known to bind to hormone receptors include steroid hormones, endogenous retinoids, protein hormones and biogenic amines. Thus, to verify whether those types of hormone are endogenously synthesised in molluscs (and thereby have a functional role), evidence should be provided for their interaction with their respective receptors. Verification of the ability (or inability) of a hormone to bind to its receptors enables more comprehensive and reliable assumptions about their activity, helping to eliminate selection and confirmation bias.

**Note 1:** changes in the messenger RNA (mRNA) expression of a receptor gene following exposure of a test animal to steroid concentrations **does not** count as evidence of hormone binding ability (e.g. changes in mRNA expression of the *ER* gene following exposure of *Mytillus* spp. to oestrogens; Fodor *et al*., 2020).

**Note 2**: certain genes that are homologous to membrane-associated steroid receptors have been shown to act as chaperone proteins for those receptors, but do not actually bind the steroid hormones themselves [e.g. progesterone receptor membrane component 1 (PGRMC1) acts as a chaperone protein for membrane progesterone receptor (mPR) but does not bind progesterone] (Fodor *et al*., 2020).

**Note 3**: since not all hormones bind to receptors, publications must be assessed for this criterion according to the type of hormone examined. Hormones known to bind to hormone receptors include steroid hormones, endogenous retinoids, protein hormones and biogenic amines. Certain exemptions apply for some hormones known to be part of these groups, and these are addressed individually in the data-extraction template. Studies that examined hormones that do not belong to these distinct groups are not assessed for this criterion.

- Was a ligand binding assay used to examine the mechanism of action (MOA) of hormones (e.g. potential interaction with a tissue-specific receptor binding site)?

| Probably low risk of bias | Verification of a hormone binding to a hormone receptor (e.g. E2 binding to ER) in molluscs using a ligand binding assay. |
| --- | --- |
|  | Verification of a hormone being incapable of binding to a hormone receptor in molluscs using a ligand binding assay. |
| Probably high risk of bias | No ligand binding assay used to verify the binding ability of a hormone (for eligible studies) |
|  | Unclear information. |

**Mollusca AND Receptors**

**6. Verification of expression of receptors in molluscs**

To confirm reliably the presence of receptor genes (and others hormone-metabolising enzymes) in molluscs, their identification in a tissue should be verified using a molecular technique that examines gene expression at the DNA or RNA level. Several receptors and hormone-metabolising enzymes have been identified in molluscs using protein assays (e.g. western blotting, antibody assays, etc.) and were reported to be present in molluscan tissues without confirmation of gene expression. Molecular techniques that measure gene expression at the mRNA level (e.g. reverse transcription quantitative polymerase chain reaction (RT-qPCR)) are considered the gold standard due to their ability to confirm the presence (and thus activity) of a gene in an organism’s genome.

- Did the method examine temporal and/or spatial distribution of the receptor’s DNA or RNA?
- Was there evidence of receptor activity in molluscs (binding to a ligand, up- or downregulated expression, etc.)?

| Definitely low risk of bias | Receptors found to be expressed in molluscan tissues using any molecular technique that examined **temporal and/or spatial distribution of RNA** (e.g. quantitative RT-PCR, qualitative PCR, *in-situ* hybridisation, Northern blotting, nuclease protection assays, etc.) **with direct evidence of the gene’s activity** (e.g. evidence of receptor binding to a ligand, up- or downregulation of receptors, etc.) in molluscan tissues. |
| --- | --- |
| Probably low risk of bias | Receptors found to be expressed in molluscan tissues using any molecular technique that examined **temporal and/or spatial distribution of DNA or RNA** (e.g. quantitative RT-PCR, qualitative PCR, *in-situ* hybridisation, Northern blotting, nuclease protection assays, etc.), **without evidence of activity** in molluscan tissues. |
| Probably high risk of bias | Identification or characterisation of a receptor by any molecular technique that measured protein levels (e.g. western blotting) instead of DNA or RNA expression in molluscan tissues. |
|  | Unclear information. |
|  | Identification or characterisation of a receptor by any other technique that did not examine temporal and/or spatial distribution of DNA or RNA. |
| Definitely high risk of bias | No approach used to confirm the expression or characterisation of receptor gene(s) in molluscan tissues. |
|  | Indications or assumptions about the presence of receptor gene(s) in molluscan tissues solely based on findings that support or indicate the conversion of hormones to other forms of hormones (aromatase converting testosterone to oestradiol in vertebrates; Fodor *et al*., 2020). |

1. **Comparison of nuclear receptor affiliation (sequence similarity analysis) using the conserved DNA binding domain (DBD) and ligand binding domain (LBD) with other invertebrate and vertebrate species**

The DNA binding domain (DBD) is a highly conserved domain containing: (1) a five-amino-acid sequence (P-box) that is responsible for determining DNA binding specificity; and (2) the D-box which controls the dimerization of the receptor (Vogeler *et al*., 2014). The ligand binding domain (LBD) is also highly conserved in structure and fairly conserved in sequence. The LBD is known to bind to hormonal ligands to cause a conformational change to the receptor which in turn induces or inhibits gene expression (Vogeler *et al*., 2014). Thus, the structure of a nuclear receptor can be determined based upon its conserved ligands, DBD and LBD, by conducting a conserved domain search and comparing its structure with previously identified nuclear receptors. This is an important step for determining the function and evolutionary history of a nuclear receptor identified in molluscs.

- Did the study conduct a nuclear receptor affiliation analysis (based on DBD and LBD)?

**Note**: due to a lack of information on DBDs and LBDs from receptors outside the nuclear receptor superfamily, studies that identified such types of receptors were not assessed for this criterion.

| Probably low risk of bias | Nuclear receptor affiliation analysis based on **both** **DBD and LBD** using appropriate methodology (e.g. conserved domain search, PFAM analysis, etc.) (Vogeler *et al*., 2014). |
| --- | --- |
| Probably high risk of bias | Nuclear receptor affiliation analysis based on **either** **the DBD or LBD** using appropriate methodology (e.g. conserved domain search, PFAM analysis, etc.). |
|  | Lack of a DBD and LBD affiliation analysis. |

1. **Phylogenetic analysis – estimation of homology among receptors**

Phylogenetics can be used to investigate the evolutionary history and relationships amongst receptors identified in different species. Similar to ligand binding affiliation, the level of homology between receptors and among different species can provide an indication of their functional preservation and phylogenetic relationships. Where there is significant similarity between two receptors, this provides strong evidence that they are related over evolutionary time. Depending on the quality of the obtained data, conclusions can also be drawn about the expression patterns and sequence similarities between receptors found in different species.

- Was a phylogenetic analysis (e.g. phylogenetic tree) conducted?
- What type of amino acid sequences were used for this analysis?
- Was homology compared with a range of species/order/class/phyla?

| Definitely low risk of bias | A phylogenetic tree was constructed by an appropriate method (e.g. neighbour joining method, MUSCLE, etc.) using **both DBD and LBD amino acid sequences**, which compares homology of the receptor transcripts with other species, order, class or phyla **(≥2**, e.g. compares homology of transcript with another species and animal class or more). |
| --- | --- |
| Probably low risk of bias | A phylogenetic tree was constructed by an appropriate method (e.g. neighbour joining method, MUSCLE) using the **entire amino acid sequence** which compares homology of the receptor transcripts with other species, order, class and phyla **(≥2**, e.g. compares homology of transcript with another species and animal class or more). |
| Probably high risk of bias | A phylogenetic tree was constructed by an appropriate method (e.g. neighbour joining method, MUSCLE) using **either DBD or LBD**, **or** **any other amino acid sequence,** which compares homology of the nuclear receptor transcripts with other species, order, class and phyla **(≥2**, e.g. compares homology of transcript with another species and animal class or more). |
|  | Unclear information. |
| Definitely high risk of bias | A phylogenetic tree was constructed by an appropriate method (e.g. neighbour joining method, MUSCLE) using **any amino acid sequence** which compares homology of the receptor transcripts with other species, order, class and phyla **(<2**, i.e. compares homology of transcript with another species only). |
|  | No phylogenetic analysis conducted. |

**Mollusca AND Enzymes**

**12. Verification of expression of hormone-metabolising enzymes in molluscs:**

- Did the method examine temporal and/or spatial distribution of the gene’s DNA or RNA, that encodes the hormone-metabolising enzyme?
- Was there evidence of activity for the hormone-metabolising enzyme in molluscs (e.g. up- or downregulated expression of gene transcripts etc.)?

| Definitely low risk of bias | Genes that encode hormone-metabolising enzymes or homologues of such genes found to be expressed in molluscan tissues using any molecular technique that examined **temporal and/or spatial distribution of DNA or RNA** (e.g. quantitative RT-PCR, qualitative PCR, *in-situ* hybridisation, Northern blotting, nuclease protection assays, etc.) with **direct evidence of the molecule’s activity** (e.g. up- or downregulation of the gene, evidence of gene/enzyme converting a molecule to its more potent form). Example: adding radiolabelled testosterone to molluscan tissues to investigate whether it can be converted to oestradiol (indicative of aromatase activity) or dihydrotestosterone (DHT) [indicative of 5-alpha-reductase (5αR) activity]. |
| --- | --- |
| Probably low risk of bias | Genes encoding hormone-metabolising enzymes or homologues of such genes found to be expressed in molluscan tissues using any molecular technique that examined **temporal and/or spatial distribution of DNA or RNA** (e.g. quantitative RT-PCR, qualitative PCR, *in-situ* hybridisation, Northern blotting, nuclease protection assays, etc.), **without evidence of the molecule’s activity** (e.g. *CYP17* homologues can cause either side-chain cleavage or 17-hydroxylation; Fodor *et al*., 2020). |
| Probably high risk of bias | Identification or characterisation of hormone-metabolising enzymes in molluscan tissues by any molecular technique that measured protein levels instead of mRNA expression (e.g. western blotting). |
|  | Unclear information. |
| Definitely high risk of bias | No approach taken to confirm the presence of the hormone-metabolising enzyme in molluscan tissues. |
|  | Indications or assumptions about the presence of the hormone-metabolising enzyme in molluscan tissues solely based on findings supporting or indicating the conversion of steroids to other forms of steroids (aromatase converting testosterone to oestradiol in vertebrates; Fodor *et al*., 2020). |

**13. Phylogenetic analysis – estimation of homology among hormone-metabolising enzymes**

Phylogenetics can also be used to verify homologous sequences between hormone-metabolising enzymes identified in molluscs with already-known protein sequences of enzymes from other species. The level of homology can reveal similarities in structure, function, or developmental mechanisms between hormone-metabolising enzymes. Depending on the quality of the obtained data, conclusions can be drawn about expression patterns and sequence similarities.

- Was a phylogenetic analysis (e.g. phylogenetic tree) conducted?
- Was homology compared with a range of species/order/class/phyla?

| Probably low risk of bias | A phylogenetic tree was constructed by an appropriate method (e.g. neighbour joining method, MUSCLE) using **any amino acid sequences** to compare homology of the hormone-metabolising enzyme with other species, order, class and phyla **(≥2**, e.g. compares homology of transcript with another species and animal class or more). |
| --- | --- |
| Probably high risk of bias | A phylogenetic tree was constructed by an appropriate method (e.g. neighbour joining method, MUSCLE) using **any amino acid sequences** which compares homology of the hormone-metabolising enzyme with other species, order, class and phyla **(<2**, e.g. compares homology of transcript with another species only). |
|  | No phylogenetic analysis conducted. |
|  | Unclear information |

**Study design criteria: Assessments A and B**

A series of criteria based on the ARRIVE guidelines 2.0 (Percie Du Sert *et al*., 2020*a*) was created to assess the quality of study design of eligible studies. Not all criteria presented in the ARRIVE guidelines were relevant or applicable, and only the three most appropriate are included in this framework. Each one was used for Assessments A and B and for studies from all three inventories (Molluscs AND Hormones; Molluscs AND Receptors; Molluscs AND Enzymes; see Appendix S6, ‘Questionnaire’ tab).

**3, 9, 14. Strategy used to minimise potential confounders**

Accounting for systematic differences between animals within different groups is a key principle during the planning of an experiment and during data analysis. The sensitivity of an experiment can be increased through the identification and management of confounding variables in the study design and analysis. Reporting any approaches taken to minimise potential confounders between treatment groups helps to eliminate bias and can be used to assess the internal validity of the study (Percie Du Sert *et al*., 2020*a*).

- Was there a description of any methods used to minimise confounding factors?
- If no measures were used to minimise confounders, was this explicitly stated and clearly explained?

| Probably low risk of bias | A description is given of the approaches taken to minimise potential confounding factors, which include (but are not limited to): randomising, time or day of the experiment, litter, animal characteristics (e.g. sex, mass, age), location of the animal/experimental unit (e.g. exposure to light or other disturbances). |
| --- | --- |
|  | Explicit statement and justification of why confounding factors were not considered/addressed. |
| Probably high risk of bias | No consideration of confounding factors, and no explanation of why this was not done. |
|  | Unclear information. |

**4, 10, 15. Within-study or between-study study repetition**

Within-study or between-study repetition occurs when a study attempts to confirm its findings independently, as part of a separate experiment (and thus a separate analysis). Independent replication must be stated explicitly by the authors (Percie Du Sert *et al*., 2020*a*), and the results must be consistent across analyses. Within-study or between-study repetition must not be confused with the number of biological replicates within one experiment (e.g. the number of experimental units), which are combined for analysis and are thus part of a single experiment (e.g. Scott, 2013). Repeating scientific findings independently helps to verify the accuracy and reliability of results. Thus, within-study or between-study repetition is an important practice that can be applied to identify and minimise performance bias (Percie Du Sert *et al*., 2020*a*).

- Was the number of biological replicates (n) clearly defined?
- Did the study combine their biological measurements during analysis?
- Did the study perform an independent verification experiment?

| Definitely low risk of bias | Studies that **combined** their biological replicates during analysis (as part of a single experiment), clearly defined the number of biological replicates (*n*) and performed at least one independent verification experiment. |
| --- | --- |
| Probably low risk of bias | Studies that did not combine their biological replicates during analysis (as part of a single experiment), but clearly defined the number of their biological replicates (*n*) and performed at least one independent verification experiment. |
|  | Studies that combined their biological replicates during analysis (as part of a single experiment), clearly defined the number of those replicates (*n*) but did not perform an independent verification experiment. |
| Probably high risk of bias | Studies that combined their biological replicates for analysis (as part of a single experiment) but did not clearly define the number of those replicates (*n*) and did not perform an independent verification experiment. |
|  | Studies that did not combine their biological replicates for analysis (as part of a single experiment), did not clearly define the number of those replicates (*n*) but did perform an independent verification experiment. |
| Definitely high risk of bias | No biological replicates performed and lack of independent verification. |
|  | Unclear/absence of information on both number of biological replicates (*n*) and independent verification of the experiment. |

**5, 11, 16. Statistics**

A detailed description of the analytical methods used is essential to ensure appropriate evaluation of the suitability of the methods selected and the validity of the results. The description of the statistical analysis should contain adequate information so that re-analysis of the raw data using the same methodology would produce the same results (Percie Du Sert *et al*., 2020*a*). Five key characteristics for inclusion were obtained from the ARRIVE guidelines 2.0.

- 1. The outcome measures
  2. The independent variables of interest
  3. The software used – including the software version and code used (if available)
  4. The exact statistical methods/analyses used, how they were performed, and the references for the methods used in the study
  5. The nuisance variables that were taken into consideration in each of the statistical tests
- Was a statistical analysis used?
- Did the study include **at least two** of the statistical characteristics included in ARRIVA guidelines 2.0?

| Probably low risk of bias | Clear description of the approach taken to analyse the data that must include **at least two** of the key characteristics. |
| --- | --- |
| Probably high risk of bias | Lack of information regarding the approach taken to analyse the data, which includes **less than two** of the key characteristics. |
|  | Unclear information. |
|  | No statistical analyses used. |

**REFERENCES**

Beato, M., & Klug, J. (2000). Steroid hormone receptors: An update. *Human Reproduction Update*, *6*(3), 225–236. https://doi.org/10.1093/humupd/6.3.225

Fodor, I., Urbán, P., Scott, A. P., & Pirger, Z. (2020). A critical evaluation of some of the recent so-called ‘evidence’ for the involvement of vertebrate-type sex steroids in the reproduction of mollusks. *Molecular and Cellular Endocrinology*, *516*(May). https://doi.org/10.1016/j.mce.2020.110949

Gust, M., Vulliet, E., Giroud, B., Garnier, F., Couturier, S., Garric, J., & Buronfosse, T. (2010). Development, validation and comparison of LC-MS/MS and RIA methods for quantification of vertebrates-like sex-steroids in prosobranch molluscs. *Journal of Chromatography B: Analytical Technologies in the Biomedical and Life Sciences*, *878*(19), 1487–1492. https://doi.org/10.1016/j.jchromb.2010.03.046

James, T., Collins, S., Amlôt, R., & Marczylo, T. (2019). Optimisation and validation of a GC–MS/MS method for the analysis of methyl salicylate in hair and skin samples for use in human-volunteer decontamination studies. *Journal of Chromatography B: Analytical Technologies in the Biomedical and Life Sciences*, *1109*(December 2018), 84–89. https://doi.org/10.1016/j.jchromb.2019.01.030

Kim, J. S., Kim, Y., Han, S. H., Jeon, J. Y., Hwang, M., Im, Y. J., Kim, J. H., Lee, S. Y., Chae, S. W., & Kim, M. G. (2013). Development and validation of an LC-MS/MS method for determination of compound K in human plasma and clinical application. *Journal of Ginseng Research*, *37*(1), 135–141. https://doi.org/10.5142/jgr.2013.37.135

Minic, R. & Zivkovic, I. (2020). Optimization, validation and standardization of ELISA. In: Norovirus. IntechOpen. https://doi.org/10.5772/intechopen.91972

Percie Du Sert, N., Ahluwaliaid, A., Alamid, S., Aveyid, M. T., Baker, M., Browneid, W. J., Clarkid, A., Cuthillid, I. C., Dirnaglid, U., Emerson, M., Garnerid, P., Holgate, S. T., Howellsid, D. W., Hurst, V., Karpid, N. A., *et al*., (2020*a*). *Reporting animal research: Explanation and elaboration for the ARRIVE guidelines 2.0*. https://doi.org/10.1371/journal.pbio.3000411

Scott, A. P. (2013). Do mollusks use vertebrate sex steroids as reproductive hormones? II. Critical review of the evidence that steroids have biological effects. *Steroids*, *78*(2), 268–281. https://doi.org/10.1016/j.steroids.2012.11.006

Singh, R. (2013). Hplc Method Development and Validation: an Overview. *Indian Journal of Pharmaceutical Education and Research*, *4*(1), p26.

Vogeler, S., Galloway, T. S., Lyons, B. P., & Bean, T. P. (2014). *The nuclear receptor gene family in the Pacific oyster, Crassostrea gigas, contains a novel subfamily group*. https://doi.org/10.1186/1471-2164-15-369

Xu, X., Keefer, L. K., Ziegler, R. G., & Veenstra, T. D. (2007). A liquid chromatography - Mass spectrometry method for the quantitative analysis of urinary endogenous estrogen metabolites. *Nature Protocols*, *2*(6), 1350–1355. https://doi.org/10.1038/nprot.2007.176
