# Supplementary material for: Mapping molluscan endocrinology: a systematic and critical appraisal
Source: Biol Rev Camb Philos Soc. 2025 Dec 16;101(2):970–1002. doi: 10.1002/brv.70112 (PMC12965858; doi:10.1002/brv.70112)
Supplement: Supplementary file 7 — Appendix S7. Supplementary information. [file BRV-101-970-s005.docx]

Appendix S7. Supplementary information

**Mapping Molluscan Endocrinology: A Systematic and Critical Appraisal**

**Authors:** Konstantinos Panagiotidis^1*^, Thomas H. Miller^1^ , Olwenn V. Martin^2^, Alice Baynes^1^ **Affiliation addresses:**

^1^*Environmental Sciences, Departure of Life Sciences, Brunel University London, Kingston Ln, London, Uxbridge UB8 3PH, UK*^2^*Department of Arts and Science, Faculty of Arts & Humanities, University College London, Gower St, London WC1E 6BT, UK*

**Correspondence:** Konstantinos Panagiotidis ([constantinospan@outlook.com](mailto:constantinospan@outlook.com))

## 1. Characteristics of the data extraction inventories


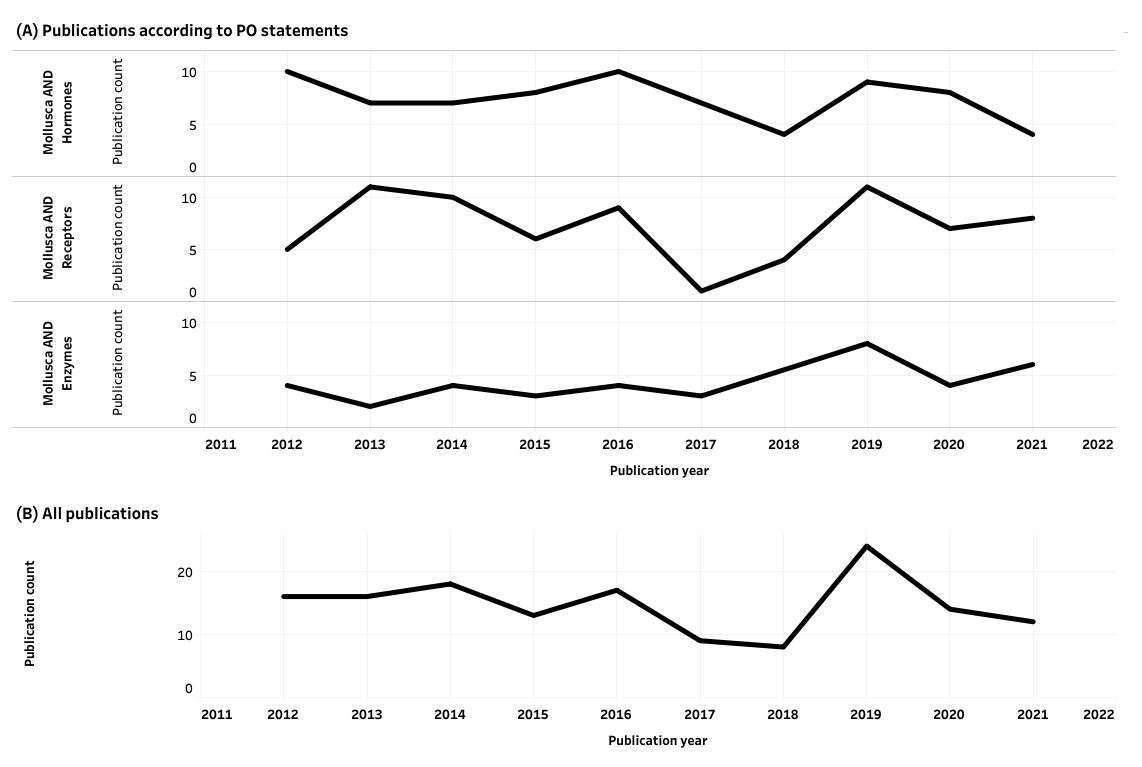


**Fig. S7.1.** (A) Number of eligible studies retrieved by year of publication according to Population, Outcome (PO) statement and (B) the total number of eligible studies retrieved in the combined data extraction inventory. Studies were retrieved on 20th September 2021. The full figure can be seen in interactive view *via* the link in Section 2 of this document.

The PO Mollusca AND Hormones was the PO statement that had the highest number of records with 74 studies, Mollusca AND Receptors had 72 and Mollusca AND Enzymes had 38 studies. Out of the 147 studies retrieved in total, 29 studies (19.7%) addressed two PO statements, whereas 4 studies (2.7%) addressed all three PO statements.

# 2. Links to data inventories and interactive figures

1. [Study design characteristics – Interactive view](https://public.tableau.com/views/Studydesigncharacteristics_/Studydesigncharacteristics?:language=en-US&:sid=&:redirect=auth&:display_count=n&:origin=viz_share_link)
2. [Mollusca AND Hormones inventory – Interactive view](https://public.tableau.com/shared/6RD8RR6YX?:display_count=n&:origin=viz_share_link)
3. [Mollusca AND Receptors inventory – Interactive view](https://public.tableau.com/views/MolluscaANDReceptors_Inventory_17616020828290/ReceptorsinMollusca?:language=en-US&:sid=&:redirect=auth&:display_count=n&:origin=viz_share_link)
4. [Mollusca AND Enzymes inventory – Interactive view](https://public.tableau.com/views/MolluscaANDEnzymes_Inventory/Hormone-metabolisingenzymes?:language=en-US&:sid=&:redirect=auth&:display_count=n&:origin=viz_share_link)
5. [Risk of Bias Assessment – Interactive view](https://public.tableau.com/views/RoBInventory_/RoBsummary?:language=en-US&:sid=&:redirect=auth&:display_count=n&:origin=viz_share_link)

# 3. List of references included in the figures

**3.1 Figure 3**

1. Abd El-Atti, M. S., El-Sayed, A. S. A., & Said, R. M. (2020). Usage of pharmaceutical contraceptive drug for controlling Eobania vermiculata snails by baits technique. *Heliyon* **6**, e05630. <https://doi.org/10.1016/j.heliyon.2020.e05630>
2. Abdel-Hamid, H., & Mekawey, A. A. I. (2014). Biological and hematological responses of Biomphalaria alexandrina to mycobiosynthsis silver nanoparticles. *Journal of the Egyptian Society of Parasitology* **44**, 627–637. <https://doi.org/10.12816/0007866>
3. Abidli, S., Santos, M. M. H., Lahbib, Y., Castro, L. F. C., Reis-Henriques, M. A., & Trigui El Menif, N. (2012). Tributyltin (TBT) effects on Hexaplex trunculus and Bolinus brandaris (Gastropoda: Muricidae): Imposex induction and sex hormone levels insights. *Ecological Indicators* **13**, 13–21. <https://doi.org/10.1016/j.ecolind.2011.05.001>
4. Abu El Einin, H. M., Ali, R. E., Gad El-Karim, R. M., Youssef, A. A., Abdel-Hamid, H., & Habib, M. R. (2019). Biomphalaria alexandrina: A model organism for assessing the endocrine disrupting effect of 17β-estradiol. *Environmental Science and Pollution Research* **26**, 23328–23336. <https://doi.org/10.1007/s11356-019-05586-0>
5. Afsar, N., Siddiqui, G., Rasheed, M., Ahmed, V. U., & Khan, A. (2012). GC-MS analysis of fatty acids (FAs) of prosobranch gastropod species thais carinifera from pakistan coast (North Arabian Sea). *Journal of the Chemical Society of Pakistan* **34**, 565–569.
6. Akcha, F., Barranger, A., Bachère, E., Berthelin, C. H., Piquemal, D., Alonso, P., Sallan, R. R., Dimastrogiovanni, G., Porte, C., Menard, D., Szczybelski, A., Benabdelmouna, A., Auffret, M., Rouxel, J., & Burgeot, T. (2016). Effects of an environmentally relevant concentration of diuron on oyster genitors during gametogenesis: Responses of early molecular and cellular markers and physiological impacts. *Environmental Science and Pollution Research* **23**, 8008–8020. <https://doi.org/10.1007/s11356-015-5969-2>
7. Avila-Poveda, O. H., Montes-Pérez, R. C., Benitez-Villalobos, F., & Rosas, C. (2013). Development and validation of a solid-phase radioimmunoassay for measuring progesterone and testosterone in octopus gonad extracts. *Malacologia* **56**, 121–134. <https://doi.org/10.4002/040.056.0209>
8. Avila-Poveda, O. H., Montes-Pérez, R. C., Koueta, N., Benítez-Villalobos, F., Ramírez-Pérez, J. S., Jimenez-Gutierrez, L. R., & Rosas, C. (2015). Seasonal changes of progesterone and testosterone concentrations throughout gonad maturation stages of the Mexican octopus, Octopus maya (Octopodidae: Octopus). *Molluscan Research* **35**, 161–172. <https://doi.org/10.1080/13235818.2015.1045055>
9. Binder, A. R. D., Pfaffl, M. W., Hiltwein, F., JuergenGeis, J., & Beggel, S. (2019). Does environmental stress affect cortisol biodistribution in freshwatermussels? *Conservation Physiology* **7**, 1–10. <https://doi.org/10.1093/conphys/coz101>
10. Cappello, T., Fernandes, D., Maisano, M., Casano, A., Bonastre, M., Bebianno, M. J., Mauceri, A., Fasulo, S., & Porte, C. (2017). Sex steroids and metabolic responses in mussels Mytilus galloprovincialis exposed to drospirenone. *Ecotoxicology and Environmental Safety* **143**, 166–172. <https://doi.org/10.1016/j.ecoenv.2017.05.031>
11. Cheour, M. K., Elgharsalli, R., Benmessaoud, R., & Aloui-Bejaoui, N. (2014). Variation of steroid concentrations during the reproductive cycle of the snail Osilinus articulatus in the Kerkennah Islands (Gulf of Gabes, Tunisia). *Cahiers de Biologie Marine* **55**, 191-199 NP – 9.
12. Chong Sánchez, F., Enríquez Díaz, M. & Aldana Aranda, D. (2020). Sex hormones in *Strombus pugilis* (Mollusca: Gastropoda) in different gonadal stages. *Bulletin of Marine Science* 96, 679–694.
13. Chong Sánchez, F., Enriquez Díaz, M., Murillo Rodríguez, E., & Aldana Aranda, D. (2019). First use of a non-invasive technique for determination of sex hormones in the queen conch Lobatus gigas, Mollusca Gastropoda. *Aquaculture International* **27**, 437–448. <https://doi.org/10.1007/s10499-018-0336-1>
14. Cubero-Leon, E., Puinean, A. M., Labadie, P., Ciocan, C., Itoh, N., Kishida, M., Osada, M., Minier, C., Hill, E. M., & Rotchell, J. M. (2012). Two CYP3A-like genes in the marine mussel Mytilus edulis: mRNA expression modulation following short-term exposure to endocrine disruptors. Marine Environmental Research 74, 32–39. [https://doi.org/10.1016/j.marenvres.2011.11.012](https://doi.org/10.1016/j.marenvres.2011.11.012?utm_source=chatgpt.com)
15. Dagorn, F., Couzinet-Mossion, A., Kendel, M., Beninger, P. G., Rabesaotra, V., Barnathan, G., & Wielgosz-Collin, G. (2016). Exploitable lipids and fatty acids in the invasive oyster Crassostrea gigas on the French Atlantic coast. *Marine Drugs* **14**, 1–12. <https://doi.org/10.3390/md14060104>
16. Dimastrogiovanni, G., Fernandes, D., Bonastre, M., & Porte, C. (2015). Progesterone is actively metabolized to 5α-pregnane-3,20-dione and 3β-hydroxy-5α-pregnan-20-one by the marine mussel Mytilus galloprovincialis. *Aquatic Toxicology* **165**, 93–100. https://doi.org/10.1016/j.aquatox.2015.05.018
17. Dokmak, H. A. A. S., El-Emam, M. A., Mossalem, H. S., El-Tayeb, T. A., & Khalil, M. T. (2021). Impact of carbamide perhydrate on the snail bulinus truncatus, the intermediate host of schistosoma haematobium. *Egyptian Journal of Aquatic Biology and Fisheries* **25**, 85–99. <https://doi.org/10.21608/ejabf.2021.172770>
18. Fiorini, R., Ventrella, V., Trombetti, F., Fabbri, M., Pagliarani, A., & Nesci, S. (2019). Lipid-protein interactions in mitochondrial membranes from bivalve mollusks: Molecular strategies in different species. *Comparative Biochemistry and Physiology Part B: Biochemistry & Molecular Biology* **227**, 12–20. <https://doi.org/10.1016/j.cbpb.2018.08.010>
19. Giusti, A., Ducrot, V., Joaquim-Justo, C., & Lagadic, L. (2013). Testosterone levels and fecundity in the hermaphroditic aquatic snail Lymnaea stagnalis exposed to testosterone and endocrine disruptors. *Environmental Toxicology and Chemistry* **32**, 1740–1745. <https://doi.org/10.1002/etc.2234>
20. Goto, Y., Kajiwara, M., Yanagisawa, Y., Hirose, H., Yoshimi, T., Umemura, M., Nakano, H., Takahashi, S., Shida, Y., Iguchi, T., Takahashi, Y., & Miura, T. (2012). Detection of vertebrate-type steroid hormones and their converting activities in the neogastropod Thais clavigera (Küster, 1858). *Journal of Molluscan Studies* **78**, 197–204. <https://doi.org/10.1093/mollus/eys001>
21. Gust, M., Gagné, F., Berlioz-Barbier, A., Besse, J. P., Buronfosse, T., Tournier, M., Tutundjian, R., Garric, J., & Cren-Olivé, C. (2014). Caged mudsnail Potamopyrgus antipodarum (Gray) as an integrated field biomonitoring tool: Exposure assessment and reprotoxic effects of water column contamination. *Water Research* **54**, 222–236. <https://doi.org/10.1016/j.watres.2014.01.057>
22. Habib, M. R., Ghoname, S. I., Ali, R. E., El-Karim, R. M. G., Youssef, A. A., Croll, R. P., & Miller, M. W. (2020). Biochemical and apoptotic changes in the nervous and ovotestis tissues of Biomphalaria alexandrina following infection with Schistosoma mansoni. *Experimental Parasitology* **213**, 107887. <https://doi.org/10.1016/j.exppara.2020.107887>
23. Halem, Z. M., Ross, D. J., & Cox, R. L. (2014). Evidence for intraspecific endocrine disruption of Geukensia demissa (Atlantic ribbed mussel) in an urban watershed. *Comparative Biochemistry and Physiology Part A : Molecular and Integrative Physiology* **175**, 1–6. <https://doi.org/10.1016/j.cbpa.2014.04.016>
24. Hallmann, A., Smolarz, K., Konieczna, L., Zabrzańska, S., Belka, M., & Baczek, T. (2016). LC-MS measurment of free steroids in mussels (Mytilus trossulus) from the southern Baltic Sea. *Journal of Pharmaceutical and Biomedical Analysis* **117**, 311–315. <https://doi.org/10.1016/j.jpba.2015.09.013>
25. Hallmann, A., Konieczna, L., Swiezak, J., Milczarek, R., & Smolarz, K. (2019). Aromatisation of steroids in the bivalve *Mytilus trossulus*. *PeerJ*, 7, e6953. <https://doi.org/10.7717/peerj.6953>
26. Hurtado, M. A., Racotta, I. S., Arcos, F., Morales-Bojórquez, E., Moal, J., Soudant, P., & Palacios, E. (2012). Seasonal variations of biochemical, pigment, fatty acid, and sterol compositions in female Crassostrea corteziensis oysters in relation to the reproductive cycle. *Comparative Biochemistry and Physiology Part B: Biochemistry and Molecular Biology* **163**, 172–183. <https://doi.org/10.1016/j.cbpb.2012.05.011>
27. Ibrahim, A. M., & Ghoname, S. I. (2018). Molluscicidal impacts of Anagallis arvensis aqueous extract on biological, hormonal, histological and molecular aspects of Biomphalaria alexandrina snails. *Experimental Parasitology* **192**, 36–41. <https://doi.org/10.1016/j.exppara.2018.07.014>
28. Ibrahim, A. M., & Sayed, D. A. (2019). Toxicological impact of oxyfluorfen 24% herbicide on the reproductive system, antioxidant enzymes, and endocrine disruption of Biomphalaria alexandrina (Ehrenberg, 1831) snails. *Environmental Science and Pollution Research* **26**, 7960–7968. <https://doi.org/10.1007/s11356-019-04251-w>
29. Kandil, M. A., Eweis, E. A., Mobarak Soha, A., & Nada Abbas, M. T. (2020). Effects of chitosan and emamectin benzoate on the reproductive system of Eobania vermiculata (Muller) land snails. *Egyptian Journal of Biological Pest Control* ***30***, 1–8. <https://doi.org/10.1186/s41938-020-00224-1>
30. Kawashima, H., Ohnishi, M., & Ogawa, S. (2013). Distribution of unusual cholesterol precursors, 4-Methyl- and 4, 4-dimethylsterols with Δ8 unsaturation, in gonads of marine archaeogastropods. *Journal of Oleo Science* **62**, 465–470. <https://doi.org/10.5650/jos.62.465>
31. Knigge, T., Dahboul, F., Alain, D., & Monsinjon, T. (2015). The gametogenic cycle and oestradiol levels in the zebra mussel Dreissena polymorpha: A 1-year study. *Journal of Molluscan Studies* **81**, 58–65. <https://doi.org/10.1093/mollus/eyu056>
32. Lazzara, R., Blázquez, M., Porte, C., & Barata, C. (2012). Low environmental levels of fluoxetine induce spawning and changes in endogenous estradiol levels in the zebra mussel Dreissena polymorpha. *Aquatic Toxicology* **106–107**, 123–130. <https://doi.org/10.1016/j.aquatox.2011.11.003>
33. Lecomte, V., Noury, P., Tutundjian, R., Buronfosse, T., Garric, J., & Gust, M. (2013). Organic solvents impair life-traits and biomarkers in the New Zealand mudsnail Potamopyrgus antipodarum (Gray) at concentrations below OECD recommendations. *Aquatic Toxicology* **140–141**, 196–203. <https://doi.org/10.1016/j.aquatox.2013.06.006>
34. Liu, J., Zhang, Z., Zhang, L., Liu, X., Yang, D., & Ma, X. (2014a). Variations of estradiol-17β and testosterone levels correlated with gametogenesis in the gonad of Zhikong scallop (Chlamys farreri) during annual reproductive cycle. *Canadian Journal of Zoology* **92**, 195–204. <https://doi.org/10.1139/cjz-2013-0202>
35. Liu, P., Miao, J., Song, Y., Pan, L., & Yin, P. (2017). Effects of 2,2’,4,4’-tetrabromodipheny ether (BDE-47) on gonadogenesis of the manila clam Ruditapes philippinarum. *Aquatic Toxicology* **193**, 178–186. <https://doi.org/10.1016/j.aquatox.2017.10.022>
36. Lü, Z. M., Liu, W., Liu, L. Q., Wang, T. M., Shi, H. L., Ping, H. L., Chi, C. F., Yang, J. W., & Wu, C. W. (2016). Cloning, Characterization, and Expression Profile of Estrogen Receptor in Common Chinese Cuttlefish, Sepiella japonica. Journal of Experimental Zoology Part A: *Ecological Genetics and Physiology* **325**, 181–193. <https://doi.org/10.1002/jez.2011>
37. Martínez-Pita, I., Sánchez-Lazo, C., & Herrera, M. (2016). A non-lethal method for establishing sexual maturation in mussels (Mytilus galloprovincialis (Lamarck, 1819)) during broodstock conditioning in hatcheries. *Aquaculture International* **24**, 1247–1254. <https://doi.org/10.1007/s10499-016-9981-4>
38. Martínez-Pita, I., Sánchez-Lazo, C., Ruíz-Jarabo, I., Herrera, M., & Mancera, J. M. (2012). Biochemical composition, lipid classes, fatty acids and sexual hormones in the mussel Mytilus galloprovincialis from cultivated populations in south Spain. *Aquaculture* **358–359**, 274–283. <https://doi.org/10.1016/j.aquaculture.2012.06.003>
39. Mezghani-Chaari, S., Machreki-Ajimi, M., Hamza-Chaffai, A., & Minier, C. (2017). High estradiol exposure disrupts the reproductive cycle of the clam Ruditapes decussatus in a sex-specific way. *Environmental Science and Pollution Research* **24**, 26670–26680. <https://doi.org/10.1007/s11356-017-0146-4>
40. Ni, J., Zeng, Z., & Ke, C. (2013). Sex steroid levels and expression patterns of estrogen receptor gene in the oyster Crassostrea angulata during reproductive cycle. Aquaculture 376–379, 105–116. [https://doi.org/10.1016/j.aquaculture.2012.11.023](https://doi.org/10.1016/j.aquaculture.2012.11.023?utm_source=chatgpt.com)
41. Nikonova, L. L., Nekhoroshev, M. V., & Ryabushko, V. I. (2017). Total testosterone and estradiol in the gonads and gametes of the mussel Mytilus galloprovincialis Lam. *Journal of Evolutionary Biochemistry and Physiology* **53**, 519–522. https://doi.org/10.1134/S0022093017060114
42. Nuurai, P., Wanichanon, C., & Wanichanon, R. (2020). Effect of gonadotropin releasing hormone on the expression of luteinizing hormone and estrogen in the nerve ganglia and ovary of a tropical abalone, Haliotis asinina Linnaeus. *Acta Histochemica* **122**, 151454. <https://doi.org/10.1016/j.acthis.2019.151454>
43. Ocharoen, Y., Boonphakdee, C., Boonphakdee, T., Shinn, A. P., & Moonmangmee, S. (2018). High levels of the endocrine disruptors bisphenol-A and 17β-estradiol detected in populations of green mussel, Perna viridis, cultured in the Gulf of Thailand. *Aquaculture*, **497**, 348–356. <https://doi.org/10.1016/j.aquaculture.2018.07.057>
44. Omran, N. E. S. E. S. (2012). Testosterone, gonadotropins and androgen receptor during spermatogenesis of Biomphalaria alexandrina snails (Pulmonata: Basommatophora). *Reproductive Biology* **12**, 301–308. <https://doi.org/10.1016/j.repbio.2012.10.002>
45. Omran, N. E., & Salama, W. M. (2016). The endocrine disruptor effect of the herbicides atrazine and glyphosate on Biomphalaria alexandrina snails. *Toxicology and Industrial Health*, 32, 656–665. <https://doi.org/10.1177/0748233713506959>
46. Rizk, M. Z., Metwally, N. S., Hamed, M. A., & Mohamed, A. M. (2012). Correlation between steroid sex hormones, egg laying capacity and cercarial shedding in Biomphalaria alexandrina snails after treatment with Haplophyllum tuberculatum. *Experimental Parasitology* **132**, 171–179. <https://doi.org/10.1016/j.exppara.2012.06.011>
47. Rossato, M., Castro, I. B., Paganini, C. L., Colares, E. P., Fillmann, G., & Pinho, G. L. L. (2016). Sex steroid imbalances in the muricid Stramonita haemastoma from TBT contaminated sites. *Environmental Science and Pollution Research* **23**, 7861–7868. <https://doi.org/10.1007/s11356-015-5942-0>
48. Sheir, S. K., Mohamad, A. H., Osman, G. Y., & Elhafez, A. E. R. A. (2020). Acute and chronic effects of bisphenol a on hormonal disruption and histological alterations in the freshwater clam, Caelatura nilotica (Cailliaud, 1827). *Egyptian Journal of Aquatic Biology and Fisheries* **24**, 397–406. <https://doi.org/10.21608/EJABF.2020.120508>
49. Smolarz, K., Zabrzańska, S., Konieczna, L., & Hallmann, A. (2018). Changes in steroid profiles of the blue mussel Mytilus trossulus as a function of season, stage of gametogenesis, sex, tissue and mussel bed depth. *General and Comparative Endocrinology* **259**, 231–239. <https://doi.org/10.1016/j.ygcen.2017.12.006>
50. Song, Y., Miao, J., Cai, Y., & Pan, L. (2015). Molecular cloning, characterization, and expression analysis of a gonadotropin-releasing hormone-like cDNA in the clam, Ruditapes philippinarum. *Comparative Biochemistry and Physiology Part B: Biochemistry and Molecular Biology* **189**, 47–54. <https://doi.org/10.1016/j.cbpb.2015.07.005>
51. Takishita, K., Takaki, Y., Chikaraishi, Y., Ikuta, T., Ozawa, G., Yoshida, T., Ohkouchi, N., & Fujikura, K. (2017). Genomic evidence thatmethanotrophic endosymbionts likely providedeep-sea bathymodiolus musselswith a sterol intermediate in cholesterol biosynthesis. *Genome Biology and Evolution* **9**, 1148–1160. <https://doi.org/10.1093/gbe/evx082>
52. Tian, S., Pan, L., & Sun, X. (2013). An investigation of endocrine disrupting effects and toxic mechanisms modulated by benzo[a]pyrene in female scallop Chlamys farreri. *Aquatic Toxicology* **144–145**, 162–171. <https://doi.org/10.1016/j.aquatox.2013.09.031>
53. Tian, S., Pan, L., Tao, Y., & Sun, X. (2015). Environmentally relevant concentrations of benzo[a]pyrene affect steroid levels and affect gonad of male scallop Chlamys farreri. *Ecotoxicology and Environmental Safety* **114**, 150–156. <https://doi.org/10.1016/j.ecoenv.2015.01.019>
54. Wang, S., Ji, C., Li, F., Zhan, J., Sun, T., Tang, J., & Wu, H. (2021). Tetrabromobisphenol A induced reproductive endocrine-disrupting effects in mussel Mytilus galloprovincialis. *Journal of Hazardous Materials* **416**, 126228. <https://doi.org/10.1016/j.jhazmat.2021.126228>
55. Wang, T., Kong, H., Shang, Y., Dupont, S., Peng, J., Wang, X., Deng, Y., Hu, M., & Wang, Y. (2021). Ocean acidification but not hypoxia alters the gonad performance in the thick shell mussel Mytilus coruscus. *Marine Pollution Bulletin* **167**, 112 282. <https://doi.org/10.1016/j.marpolbul.2021.112282>
56. Yang, Y., Pan, L., Zhou, Y., Xu, R., & Li, D. (2020). Benzo[a]pyrene exposure disrupts steroidogenesis and impairs spermatogenesis in diverse reproductive stages of male scallop (Chlamys farreri). *Environmental Research* **191**, 110–125. <https://doi.org/10.1016/j.envres.2020.110125>
57. Zabrzańska, S., Smolarz, K., Hallmann, A., Konieczna, L., Baczek, T., & Wołowicz, M. (2015). Sex-related differences in steroid concentrations in the blue mussel (Mytilus edulis trossulus) from the southern Baltic Sea. Comparative Biochemistry and Physiology Part A : *Molecular and Integrative Physiology* **183**, 14–19. <https://doi.org/10.1016/j.cbpa.2014.12.029>
58. Zapata-Restrepo, L. M., Hauton, C., Williams, I. D., Jensen, A. C., & Hudson, M. D. (2019). Effects of the interaction between temperature and steroid hormones on gametogenesis and sex ratio in the European flat oyster (Ostrea edulis). *Comparative Biochemistry and Physiology Part A : Molecular and Integrative Physiology* **236,** 110523. <https://doi.org/10.1016/j.cbpa.2019.06.023>
59. Zhang, M., Wei, H., Liu, T., Li, W., Li, Y., Wang, S., Xing, Q., Hu, X., Zhang, L., & Bao, Z. (2020). Potential GnRH and steroidogenesis pathways in the scallop Patinopecten yessoensis. *Journal of Steroid Biochemistry and Molecular Biology* **204**, 105756. <https://doi.org/10.1016/j.jsbmb.2020.105756>
60. Zhang, Z., Bai, Q., Xu, X., & Zhang, X. (2021). Effects of the dominance hierarchy on social interactions, cortisol level, HPG-axis activities and reproductive success in the golden cuttlefish Sepia esculenta. *Aquaculture* **533**, 736059. <https://doi.org/10.1016/j.aquaculture.2020.736059>
61. Zheng, B. H., An, L. H., Chang, H., Liu, Y., & Jiang, Z. Q. (2014). Evidence for the presence of sex steroid hormones in Zhikong scallop, Chlamys farreri. *Journal of Steroid Biochemistry and Molecular Biology* **143**, 199–206. <https://doi.org/10.1016/j.jsbmb.2014.03.002>
62. Zhu, X., Guo, C., Lin, C., Wang, D., Wang, C., & Xu, S. (2018). Estradiol-17β and testosterone levels during the annual reproductive cycle of in Mytilus coruscus. *Animal Reproduction Science* **196**, 35–42. <https://doi.org/10.1016/j.anireprosci.2018.06.015>

**3.2 Figure 4**

1. Abd El-Atti, M. S., El-Sayed, A. S. A., & Said, R. M. (2020). Usage of pharmaceutical contraceptive drug for controlling Eobania vermiculata snails by baits technique. *Heliyon* **6**, e05630. <https://doi.org/10.1016/j.heliyon.2020.e05630>
2. Afsar, N., Siddiqui, G., Rasheed, M., Ahmed, V. U., & Khan, A. (2012). GC-MS analysis of fatty acids (FAs) of prosobranch gastropod species thais carinifera from pakistan coast (North Arabian Sea). *Journal of the Chemical Society of Pakistan* **34**, 565–569.
3. Dagorn, F., Couzinet-Mossion, A., Kendel, M., Beninger, P. G., Rabesaotra, V., Barnathan, G., & Wielgosz-Collin, G. (2016). Exploitable lipids and fatty acids in the invasive oyster Crassostrea gigas on the French Atlantic coast. *Marine Drugs* **14**, 1–12. <https://doi.org/10.3390/md14060104>
4. Fiorini, R., Ventrella, V., Trombetti, F., Fabbri, M., Pagliarani, A., & Nesci, S. (2019). Lipid-protein interactions in mitochondrial membranes from bivalve mollusks: Molecular strategies in different species. *Comparative Biochemistry and Physiology Part B: Biochemistry & Molecular Biology* **227**, 12–20. <https://doi.org/10.1016/j.cbpb.2018.08.010>
5. Giner, J. L., Zhao, H., Dixon, M. S., & Wikfors, G. H. (2016). Bioconversion of 13C-labeled microalgal phytosterols to cholesterol by the Northern Bay scallop, Argopecten irradians irradians. *Comparative Biochemistry and Physiology Part B: Biochemistry & Molecular Biology* **192**, 1–8. <https://doi.org/10.1016/j.cbpb.2015.11.003>
6. Hurtado, M. A., Racotta, I. S., Arcos, F., Morales-Bojórquez, E., Moal, J., Soudant, P., & Palacios, E. (2012). Seasonal variations of biochemical, pigment, fatty acid, and sterol compositions in female Crassostrea corteziensis oysters in relation to the reproductive cycle. *Comparative Biochemistry and Physiology Part B: Biochemistry and Molecular Biology* **163**, 172–183. <https://doi.org/10.1016/j.cbpb.2012.05.011>
7. Nuurai, P., Wanichanon, C., & Wanichanon, R. (2020). Effect of gonadotropin releasing hormone on the expression of luteinizing hormone and estrogen in the nerve ganglia and ovary of a tropical abalone, Haliotis asinina Linnaeus. *Acta Histochemica* **122**, 151454. <https://doi.org/10.1016/j.acthis.2019.151454>
8. Zhang, Z., Bai, Q., Xu, X., & Zhang, X. (2021). Effects of the dominance hierarchy on social interactions, cortisol level, HPG-axis activities and reproductive success in the golden cuttlefish Sepia esculenta. *Aquaculture* **533**, 736059. <https://doi.org/10.1016/j.aquaculture.2020.736059>

**3.3 Figure 5**

1. Abd El-Atti, M. S., El-Sayed, A. S. A., & Said, R. M. (2020). Usage of pharmaceutical contraceptive drug for controlling Eobania vermiculata snails by baits technique. *Heliyon* **6**, e05630. <https://doi.org/10.1016/j.heliyon.2020.e05630>
2. Abidli, S., Santos, M. M. H., Lahbib, Y., Castro, L. F. C., Reis-Henriques, M. A., & Trigui El Menif, N. (2012). Tributyltin (TBT) effects on Hexaplex trunculus and Bolinus brandaris (Gastropoda: Muricidae): Imposex induction and sex hormone levels insights. *Ecological Indicators* **13**, 13–21. <https://doi.org/10.1016/j.ecolind.2011.05.001>
3. Abu El Einin, H. M., Ali, R. E., Gad El-Karim, R. M., Youssef, A. A., Abdel-Hamid, H., & Habib, M. R. (2019). Biomphalaria alexandrina: A model organism for assessing the endocrine disrupting effect of 17β-estradiol. *Environmental Science and Pollution Research* **26**, 23328–23336. <https://doi.org/10.1007/s11356-019-05586-0>
4. Akcha, F., Barranger, A., Bachère, E., Berthelin, C. H., Piquemal, D., Alonso, P., Sallan, R. R., Dimastrogiovanni, G., Porte, C., Menard, D., Szczybelski, A., Benabdelmouna, A., Auffret, M., Rouxel, J., & Burgeot, T. (2016). Effects of an environmentally relevant concentration of diuron on oyster genitors during gametogenesis: Responses of early molecular and cellular markers and physiological impacts. *Environmental Science and Pollution Research* **23**, 8008–8020. <https://doi.org/10.1007/s11356-015-5969-2>
5. Avila-Poveda, O. H., Montes-Pérez, R. C., Benitez-Villalobos, F., & Rosas, C. (2013). Development and validation of a solid-phase radioimmunoassay for measuring progesterone and testosterone in octopus gonad extracts. *Malacologia* **56**, 121–134. <https://doi.org/10.4002/040.056.0209>
6. Avila-Poveda, O. H., Montes-Pérez, R. C., Koueta, N., Benítez-Villalobos, F., Ramírez-Pérez, J. S., Jimenez-Gutierrez, L. R., & Rosas, C. (2015). Seasonal changes of progesterone and testosterone concentrations throughout gonad maturation stages of the Mexican octopus, Octopus maya (Octopodidae: Octopus). *Molluscan Research* **35**, 161–172. <https://doi.org/10.1080/13235818.2015.1045055>
7. Binder, A. R. D., Pfaffl, M. W., Hiltwein, F., JuergenGeis, J., & Beggel, S. (2019). Does environmental stress affect cortisol biodistribution in freshwatermussels? *Conservation Physiology* **7**, 1–10. <https://doi.org/10.1093/conphys/coz101>
8. Cappello, T., Fernandes, D., Maisano, M., Casano, A., Bonastre, M., Bebianno, M. J., Mauceri, A., Fasulo, S., & Porte, C. (2017). Sex steroids and metabolic responses in mussels Mytilus galloprovincialis exposed to drospirenone. *Ecotoxicology and Environmental Safety* **143**, 166–172. <https://doi.org/10.1016/j.ecoenv.2017.05.031>
9. Cheour, M. K., Elgharsalli, R., Benmessaoud, R., & Aloui-Bejaoui, N. (2014). Variation of steroid concentrations during the reproductive cycle of the snail Osilinus articulatus in the Kerkennah Islands (Gulf of Gabes, Tunisia). *Cahiers de Biologie Marine* **55**, 191-199 NP – 9.
10. Chong Sánchez, F., Enríquez Díaz, M. & Aldana Aranda, D. (2020). Sex hormones in *Strombus pugilis* (Mollusca: Gastropoda) in different gonadal stages. *Bulletin of Marine Science* 96, 679–694.
11. Dimastrogiovanni, G., Fernandes, D., Bonastre, M., & Porte, C. (2015). Progesterone is actively metabolized to 5α-pregnane-3,20-dione and 3β-hydroxy-5α-pregnan-20-one by the marine mussel Mytilus galloprovincialis. *Aquatic Toxicology* **165**, 93–100. https://doi.org/10.1016/j.aquatox.2015.05.018
12. Dokmak, H. A. A. S., El-Emam, M. A., Mossalem, H. S., El-Tayeb, T. A., & Khalil, M. T. (2021). Impact of carbamide perhydrate on the snail bulinus truncatus, the intermediate host of schistosoma haematobium. *Egyptian Journal of Aquatic Biology and Fisheries* **25**, 85–99. <https://doi.org/10.21608/ejabf.2021.172770>
13. Giusti, A., Ducrot, V., Joaquim-Justo, C., & Lagadic, L. (2013). Testosterone levels and fecundity in the hermaphroditic aquatic snail Lymnaea stagnalis exposed to testosterone and endocrine disruptors. *Environmental Toxicology and Chemistry* **32**, 1740–1745. <https://doi.org/10.1002/etc.2234>
14. Gust, M., Gagné, F., Berlioz-Barbier, A., Besse, J. P., Buronfosse, T., Tournier, M., Tutundjian, R., Garric, J., & Cren-Olivé, C. (2014). Caged mudsnail Potamopyrgus antipodarum (Gray) as an integrated field biomonitoring tool: Exposure assessment and reprotoxic effects of water column contamination. *Water Research* **54**, 222–236. <https://doi.org/10.1016/j.watres.2014.01.057>
15. Habib, M. R., Ghoname, S. I., Ali, R. E., El-Karim, R. M. G., Youssef, A. A., Croll, R. P., & Miller, M. W. (2020). Biochemical and apoptotic changes in the nervous and ovotestis tissues of Biomphalaria alexandrina following infection with Schistosoma mansoni. *Experimental Parasitology* **213**, 107887. <https://doi.org/10.1016/j.exppara.2020.107887>
16. Halem, Z. M., Ross, D. J., & Cox, R. L. (2014). Evidence for intraspecific endocrine disruption of Geukensia demissa (Atlantic ribbed mussel) in an urban watershed. *Comparative Biochemistry and Physiology Part A : Molecular and Integrative Physiology* **175**, 1–6. <https://doi.org/10.1016/j.cbpa.2014.04.016>
17. Huang, W., Xu, F., Li, L., Que, H., & Zhang, G. (2019). The transcription of iodothyronine deiodinase genes is regulated by thyroid hormone receptor in the Pacific oyster Crassostrea gigas. *Journal of Oceanology and Limnology* **37**, 1317–1323. <https://doi.org/10.1007/s00343-019-8207-9>
18. Ibrahim, A. M., & Ghoname, S. I. (2018). Molluscicidal impacts of Anagallis arvensis aqueous extract on biological, hormonal, histological and molecular aspects of Biomphalaria alexandrina snails. *Experimental Parasitology* **192**, 36–41. <https://doi.org/10.1016/j.exppara.2018.07.014>
19. Ibrahim, A. M., & Sayed, D. A. (2019). Toxicological impact of oxyfluorfen 24% herbicide on the reproductive system, antioxidant enzymes, and endocrine disruption of Biomphalaria alexandrina (Ehrenberg, 1831) snails. *Environmental Science and Pollution Research* **26**, 7960–7968. <https://doi.org/10.1007/s11356-019-04251-w>
20. Jiang, S., Miao, J., Wang, X., Liu, P., & Pan, L. (2019). Inhibition of growth in juvenile manila clam Ruditapes philippinarum: Potential adverse outcome pathway of TBBPA. *Chemosphere* **224**, 588–596. <https://doi.org/10.1016/j.chemosphere.2019.02.157>
21. Kandil, M. A., Eweis, E. A., Mobarak Soha, A., & Nada Abbas, M. T. (2020). Effects of chitosan and emamectin benzoate on the reproductive system of Eobania vermiculata (Muller) land snails. *Egyptian Journal of Biological Pest Control* ***30***, 1–8. <https://doi.org/10.1186/s41938-020-00224-1>
22. Knigge, T., Dahboul, F., Alain, D., & Monsinjon, T. (2015). The gametogenic cycle and oestradiol levels in the zebra mussel Dreissena polymorpha: A 1-year study. *Journal of Molluscan Studies* **81**, 58–65. <https://doi.org/10.1093/mollus/eyu056>
23. Lazzara, R., Blázquez, M., Porte, C., & Barata, C. (2012). Low environmental levels of fluoxetine induce spawning and changes in endogenous estradiol levels in the zebra mussel Dreissena polymorpha. *Aquatic Toxicology* **106–107**, 123–130. <https://doi.org/10.1016/j.aquatox.2011.11.003>
24. Lecomte, V., Noury, P., Tutundjian, R., Buronfosse, T., Garric, J., & Gust, M. (2013). Organic solvents impair life-traits and biomarkers in the New Zealand mudsnail Potamopyrgus antipodarum (Gray) at concentrations below OECD recommendations. *Aquatic Toxicology* **140–141**, 196–203. <https://doi.org/10.1016/j.aquatox.2013.06.006>
25. Liu, J., Zhang, Z., Zhang, L., Liu, X., Yang, D., & Ma, X. (2014a). Variations of estradiol-17β and testosterone levels correlated with gametogenesis in the gonad of Zhikong scallop (Chlamys farreri) during annual reproductive cycle. *Canadian Journal of Zoology* **92**, 195–204. <https://doi.org/10.1139/cjz-2013-0202>
26. Liu, P., Miao, J., Song, Y., Pan, L., & Yin, P. (2017). Effects of 2,2’,4,4’-tetrabromodipheny ether (BDE-47) on gonadogenesis of the manila clam Ruditapes philippinarum. *Aquatic Toxicology* **193**, 178–186. <https://doi.org/10.1016/j.aquatox.2017.10.022>
27. Lü, Z. M., Liu, W., Liu, L. Q., Wang, T. M., Shi, H. L., Ping, H. L., Chi, C. F., Yang, J. W., & Wu, C. W. (2016). Cloning, Characterization, and Expression Profile of Estrogen Receptor in Common Chinese Cuttlefish, Sepiella japonica. Journal of Experimental Zoology Part A: *Ecological Genetics and Physiology* **325**, 181–193. <https://doi.org/10.1002/jez.2011>
28. Lustrino, D., Silva, A. C. M., Araujo, I. G., Tunholi, V. M., Tunholi-Alves, V. M., Castro, R. N., Carvalho, D. P., Pinheiro, J., & Marassi, M. P. (2017). Evidence of the presence of thyroid hormones in *Achatina fulica* snails. *Anais da Academia Brasileira de Ciências*, 89(3 Suppl), 2181–2188. <https://doi.org/10.1590/0001-3765201720160698>
29. Martínez-Pita, I., Sánchez-Lazo, C., & Herrera, M. (2016). A non-lethal method for establishing sexual maturation in mussels (Mytilus galloprovincialis (Lamarck, 1819)) during broodstock conditioning in hatcheries. *Aquaculture International* **24**, 1247–1254. <https://doi.org/10.1007/s10499-016-9981-4>
30. Martínez-Pita, I., Sánchez-Lazo, C., Ruíz-Jarabo, I., Herrera, M., & Mancera, J. M. (2012). Biochemical composition, lipid classes, fatty acids and sexual hormones in the mussel Mytilus galloprovincialis from cultivated populations in south Spain. *Aquaculture* **358–359**, 274–283. <https://doi.org/10.1016/j.aquaculture.2012.06.003>
31. Ni, J., Zeng, Z., & Ke, C. (2013). Sex steroid levels and expression patterns of estrogen receptor gene in the oyster Crassostrea angulata during reproductive cycle. Aquaculture 376–379, 105–116. [https://doi.org/10.1016/j.aquaculture.2012.11.023](https://doi.org/10.1016/j.aquaculture.2012.11.023?utm_source=chatgpt.com)
32. Nikonova, L. L., Nekhoroshev, M. V., & Ryabushko, V. I. (2017). Total testosterone and estradiol in the gonads and gametes of the mussel Mytilus galloprovincialis Lam. *Journal of Evolutionary Biochemistry and Physiology* **53**, 519–522. https://doi.org/10.1134/S0022093017060114
33. Nuurai, P., Wanichanon, C., & Wanichanon, R. (2020). Effect of gonadotropin releasing hormone on the expression of luteinizing hormone and estrogen in the nerve ganglia and ovary of a tropical abalone, Haliotis asinina Linnaeus. *Acta Histochemica* **122**, 151454. <https://doi.org/10.1016/j.acthis.2019.151454>
34. Omran, N. E. S. E. S. (2012). Testosterone, gonadotropins and androgen receptor during spermatogenesis of Biomphalaria alexandrina snails (Pulmonata: Basommatophora). *Reproductive Biology* **12**, 301–308. <https://doi.org/10.1016/j.repbio.2012.10.002>
35. Omran, N. E., & Salama, W. M. (2016). The endocrine disruptor effect of the herbicides atrazine and glyphosate on Biomphalaria alexandrina snails. *Toxicology and Industrial Health*, 32, 656–665. <https://doi.org/10.1177/0748233713506959>
36. Rossato, M., Castro, I. B., Paganini, C. L., Colares, E. P., Fillmann, G., & Pinho, G. L. L. (2016). Sex steroid imbalances in the muricid Stramonita haemastoma from TBT contaminated sites. *Environmental Science and Pollution Research* **23**, 7861–7868. <https://doi.org/10.1007/s11356-015-5942-0>
37. Sheir, S. K., Mohamad, A. H., Osman, G. Y., & Elhafez, A. E. R. A. (2020). Acute and chronic effects of bisphenol a on hormonal disruption and histological alterations in the freshwater clam, Caelatura nilotica (Cailliaud, 1827). *Egyptian Journal of Aquatic Biology and Fisheries* **24**, 397–406. <https://doi.org/10.21608/EJABF.2020.120508>
38. Song, Y., Miao, J., Cai, Y., & Pan, L. (2015). Molecular cloning, characterization, and expression analysis of a gonadotropin-releasing hormone-like cDNA in the clam, Ruditapes philippinarum. *Comparative Biochemistry and Physiology Part B: Biochemistry and Molecular Biology* **189**, 47–54. <https://doi.org/10.1016/j.cbpb.2015.07.005>
39. Song, Y., Miao, J., Pan, L., & Wang, X. (2016). Exposure to2,2’,4,4’-tetrabromodiphenyl ether (BDE-47) alters thyroid hormone levels and thyroid hormone-regulated gene transcription in manila clam Ruditapes philippinarum. *Chemosphere*, **152**, 10–16. <https://doi.org/10.1016/j.chemosphere.2016.02.049>
40. Tian, S., Pan, L., & Sun, X. (2013). An investigation of endocrine disrupting effects and toxic mechanisms modulated by benzo[a]pyrene in female scallop Chlamys farreri. *Aquatic Toxicology* **144–145**, 162–171. <https://doi.org/10.1016/j.aquatox.2013.09.031>
41. Tian, S., Pan, L., Tao, Y., & Sun, X. (2015). Environmentally relevant concentrations of benzo[a]pyrene affect steroid levels and affect gonad of male scallop Chlamys farreri. *Ecotoxicology and Environmental Safety* **114**, 150–156. <https://doi.org/10.1016/j.ecoenv.2015.01.019>
42. Yang, Y., Pan, L., Zhou, Y., Xu, R., & Li, D. (2020). Benzo[a]pyrene exposure disrupts steroidogenesis and impairs spermatogenesis in diverse reproductive stages of male scallop (Chlamys farreri). *Environmental Research* **191**, 110–125. <https://doi.org/10.1016/j.envres.2020.110125>
43. Zapata-Restrepo, L. M., Hauton, C., Williams, I. D., Jensen, A. C., & Hudson, M. D. (2019). Effects of the interaction between temperature and steroid hormones on gametogenesis and sex ratio in the European flat oyster (Ostrea edulis). *Comparative Biochemistry and Physiology Part A : Molecular and Integrative Physiology* **236,** 110523. <https://doi.org/10.1016/j.cbpa.2019.06.023>
44. Zhang, M., Wei, H., Liu, T., Li, W., Li, Y., Wang, S., Xing, Q., Hu, X., Zhang, L., & Bao, Z. (2020). Potential GnRH and steroidogenesis pathways in the scallop Patinopecten yessoensis. *Journal of Steroid Biochemistry and Molecular Biology* **204**, 105756. <https://doi.org/10.1016/j.jsbmb.2020.105756>
45. Zhang, Z., Bai, Q., Xu, X., & Zhang, X. (2021). Effects of the dominance hierarchy on social interactions, cortisol level, HPG-axis activities and reproductive success in the golden cuttlefish Sepia esculenta. *Aquaculture* **533**, 736059. <https://doi.org/10.1016/j.aquaculture.2020.736059>
46. Zhu, X., Guo, C., Lin, C., Wang, D., Wang, C., & Xu, S. (2018). Estradiol-17β and testosterone levels during the annual reproductive cycle of in Mytilus coruscus. *Animal Reproduction Science* **196**, 35–42. <https://doi.org/10.1016/j.anireprosci.2018.06.015>

**3.4 Figure 6**

1. Abidli, S., Castro, L. F. C., Lahbib, Y., Reis-Henriques, M. A., Trigui El Menif, N., & Santos, M. M. (2013). Imposex development in Hexaplex trunculus (Gastropoda: Caenogastropoda) involves changes in the transcription levels of the retinoid X receptor (RXR). *Chemosphere* **93**, 1161–1167. <https://doi.org/10.1016/j.chemosphere.2013.06.054>
2. Agnese, M., Rosati, L., Prisco, M., Borzacchiello, L., Abagnale, L., & Andreuccetti, P. (2019). The expression of estrogen receptors during the Mytilus galloprovincialis ovarian cycle*. Journal of Experimental Zoology Part A: Ecological and Integrative Physiology* **331**, 367–373. <https://doi.org/10.1002/jez.2272>
3. André, A., Ruivo, R., Fonseca, E., Froufe, E., Castro, L. F. C., & Santos, M. M. (2019). The retinoic acid receptor (RAR) in molluscs: Function, evolution and endocrine disruption insights. *Aquatic Toxicology* **208**, 80–89. <https://doi.org/10.1016/j.aquatox.2019.01.002>
4. Aquilino, M., Martínez-Guitarte, J. L., García, P., Beltrán, E. M., Fernández, C., & Sánchez-Argüello, P. (2018). Combining the assessment of apical endpoints and gene expression in the freshwater snail Physa acuta after exposure to reclaimed water. *Science of the Total Environment* **642**, 80–189. <https://doi.org/10.1016/j.scitotenv.2018.06.054>
5. Aquilino, M., Sánchez-Argüello, P., Novo, M., & Martínez-Guitarte, J. L. (2019). Effects on tadpole snail gene expression after exposure to vinclozolin. *Ecotoxicology and Environmental Safety* **170**, 568–577. <https://doi.org/10.1016/j.ecoenv.2018.12.015>
6. Balbi, T., Franzellitti, S., Fabbri, R., Montagna, M., Fabbri, E., & Canesi, L. (2016). Impact of bisphenol A (BPA) on early embryo development in the marine mussel Mytilus galloprovincialis: Effects on gene transcription. *Environmental Pollution* **218**, 996–1004. <https://doi.org/10.1016/j.envpol.2016.08.050>
7. Bannister, R., Beresford, N., Granger, D. W., Pounds, N. A., Rand-Weaver, M., White, R., Jobling, S., & Routledge, E. J. (2013). No substantial changes in estrogen receptor and estrogen-related receptor orthologue gene transcription in Marisa cornuarietis exposed to estrogenic chemicals. *Aquatic Toxicology* **140–141**, 19–26. <https://doi.org/10.1016/j.aquatox.2013.05.002>
8. Bouétard, A., Besnard, A. L., Vassaux, D., Lagadic, L., & Coutellec, M. A. (2013). Impact of the redox-cycling herbicide diquat on transcript expression and antioxidant enzymatic activities of the freshwater snail Lymnaea stagnalis. *Aquatic Toxicology* **126**, 256–265. <https://doi.org/10.1016/j.aquatox.2012.11.013>
9. Capitão, A. M. F., Lopes-Marques, M., Páscoa, I., Sainath, S. B., Hiromori, Y., Matsumaru, D., Nakanishi, T., Ruivo, R., Santos, M. M., & Castro, L. F. C. (2021). An ancestral nuclear receptor couple, PPAR-RXR, is exploited by organotins. *Science of the Total Environment* **797**, 149044. <https://doi.org/10.1016/j.scitotenv.2021.149044>
10. Carpenter, S., Rothwell, C. M., Wright, M. L., de Hoog, E., Walker, S., Hudson, E., & Spencer, G. E. (2016). Extending the duration of long-term memories: Interactions between environmental darkness and retinoid signaling. *Neurobiology of Learning and Memory* **136**, 34–46. <https://doi.org/10.1016/j.nlm.2016.09.008>
11. Carter, C. J., Rand, C., Mohammad, I., Lepp, A., Vesprini, N., Wiebe, O., Carlone, R., & Spencer, G. E. (2015). Expression of a retinoic acid receptor (RAR)-like protein in the embryonic and adult nervous system of a protostome species. *Journal of Experimental Zoology Part B: Molecular and Developmental Evolution* **324**, 51–67. <https://doi.org/10.1002/jez.b.22604>
12. De Lisa, E., Paolucci, M., & Di Cosmo, A. (2012). Conservative Nature of Oestradiol Signalling Pathways in the Brain Lobes of Octopus vulgaris Involved in Reproduction, Learning and Motor Coordination. *Journal of Neuroendocrinology* **24**, 275–284. <https://doi.org/10.1111/j.1365-2826.2011.02240.x>
13. Deng, X., Pan, L., Cai, Y., & Jin, Q. (2016). Transcriptomic changes in the ovaries of scallop Chlamys farreri exposed to benzo[a]pyrene. *Genes and Genomics* **38**, 509–518. <https://doi.org/10.1007/s13258-016-0397-3>
14. Gesto, M., Ruivo, R., Páscoa, I., André, A., Castro, L. F. C., & Santos, M. M. (2016). Retinoid level dynamics during gonad recycling in the limpet Patella vulgata. General and Comparative Endocrinology, 225, 142–148. <https://doi.org/10.1016/j.ygcen.2015.10.017>
15. Giraud-Billoud, M., & Castro-Vazquez, A. (2019). Aging and retinoid X receptor agonists on masculinization of female Pomacea canaliculata, with a critical appraisal of imposex evaluation in the Ampullariidae. *Ecotoxicology and Environmental Safety* **169**, 573–582. <https://doi.org/10.1016/j.ecoenv.2018.10.096>
16. Giulianelli, S., Primost, M. A., Lanari, C., & Bigatti, G. (2020). RXR Expression in Marine Gastropods with Different Sensitivity to Imposex Development. *Scientific Reports* **10**, 9507. <https://doi.org/10.1038/s41598-020-66402-1>
17. Gust, M., Gagné, F., Berlioz-Barbier, A., Besse, J. P., Buronfosse, T., Tournier, M., Tutundjian, R., Garric, J., & Cren-Olivé, C. (2014). Caged mudsnail Potamopyrgus antipodarum (Gray) as an integrated field biomonitoring tool: Exposure assessment and reprotoxic effects of water column contamination. *Water Research* **54**, 222–236. <https://doi.org/10.1016/j.watres.2014.01.057>
18. Gutierrez-Mazariegos, J., Nadendla, E. K., Lima, D., Pierzchalski, K., Jones, J. W., Kane, M., Nishikawa, J. I., Hiromori, Y., Nakanishi, T., Santos, M. M., Castro, L. F. C., Bourguet, W., Schubert, M., & Laudet, V. (2014). A mollusk retinoic acid receptor (RAR) ortholog sheds light on the evolution of ligand binding. *Endocrinology* **155**, 4275–4286. <https://doi.org/10.1210/en.2014-1181>
19. Huang, W., Wu, Q., Xu, F., Li, L., Li, J., Que, H., & Zhang, G. (2020). Functional characterization of retinoid X receptor with an emphasis on the mediation of organotin poisoning in the Pacific oyster (Crassostrea gigas). *Gene* **753**, 144780. <https://doi.org/10.1016/j.gene.2020.144780>
20. Huang, W., Xu, F., Li, J., Li, L., Que, H., & Zhang, G. (2015a). Evolution of a novel nuclear receptor subfamily with emphasis on the member from the Pacific oyster Crassostrea gigas. *Gene* **567**, 164–172. <https://doi.org/10.1016/j.gene.2015.04.082>
21. Huang, W., Xu, F., Li, L., Que, H., & Zhang, G. (2019). The transcription of iodothyronine deiodinase genes is regulated by thyroid hormone receptor in the Pacific oyster Crassostrea gigas. *Journal of Oceanology and Limnology* **37**, 1317–1323. <https://doi.org/10.1007/s00343-019-8207-9>
22. Hultin, C. L., Hallgren, P., & Hansson, M. C. (2016). Estrogen receptor genes in gastropods: Phylogenetic divergence and gene expression responses to a synthetic estrogen. *Comparative Biochemistry and Physiology Part C: Toxicology and Pharmacology* **189**, 17–21. <https://doi.org/10.1016/j.cbpc.2016.07.002>
23. Hultin, C. L., Hallgren, P., Persson, A., & Hansson, M. C. (2014). Identification of an estrogen receptor gene in the natural freshwater snail Bithynia tentaculata. *Gene* **540**, 26–31. <https://doi.org/10.1016/j.gene.2014.02.039>
24. Ip, J. C. H., Leung, P. T. Y., Ho, K. K. Y., Qiu, J. W., & Leung, K. M. Y. (2016). De novo transcriptome assembly of the marine gastropod Reishia clavigera for supporting toxic mechanism studies. *Aquatic Toxicology* **178**, 39–48. <https://doi.org/10.1016/j.aquatox.2016.07.006>
25. Jin, K., Jin, Q., Cai, Z., Huang, B., Wei, L., Zhang, M., Guo, W., Liu, Y., & Wang, X. (2021). Molecular Characterization of Retinoic Acid Receptor CgRAR in Pacific Oyster (Crassostrea gigas). *Frontiers in Physiology* **12**, 1–6. <https://doi.org/10.3389/fphys.2021.666842>
26. Johnson, A., de Hoog, E., Tolentino, M., Nasser, T., & Spencer, G. E. (2019). Pharmacological evidence for the role of RAR in axon guidance and embryonic development of a protostome species. *Genesis* **57**, 1–14. <https://doi.org/10.1002/dvg.23301>
27. Juárez, O. E., López-Galindo, L., Pérez-Carrasco, L., Lago-Lestón, A., Rosas, C., Cosmo, A. D., & Galindo-Sánchez, C. E. (2019). Octopus maya white body show sex-specific transcriptomic profiles during the reproductive phase, with high differentiation in signaling pathways. *PLoS ONE* **14**, 1–29. <https://doi.org/10.1371/journal.pone.0216982>
28. Lecomte, V., Noury, P., Tutundjian, R., Buronfosse, T., Garric, J., & Gust, M. (2013). Organic solvents impair life-traits and biomarkers in the New Zealand mudsnail Potamopyrgus antipodarum (Gray) at concentrations below OECD recommendations. *Aquatic Toxicology* **140–141**, 196–203. <https://doi.org/10.1016/j.aquatox.2013.06.006>
29. Lesoway, M. P., & Henry, J. Q. (2021). Retinoids promote penis development in sequentially hermaphroditic snails. *Developmental Biology* **478**, 122–132. <https://doi.org/10.1016/j.ydbio.2021.06.013>
30. Li, H., Liu, J., Huang, X., Wang, D., & Zhang, Z. (2014). Characterization, expression and function analysis of DAX1 gene of scallop (Chlamys farreri jones and preston 1904) during its gametogenesis*. Journal of Ocean University of China* **13**, 696–704. <https://doi.org/10.1007/s11802-014-2299-9>
31. Li, Y. F., Cheng, Y. L., Chen, K., Cheng, Z. Y., Zhu, X., C. R. Cardoso, J., Liang, X., Zhu, Y. T., Power, D. M., & Yang, J. L. (2020). Thyroid hormone receptor: A new player in epinephrine-induced larval metamorphosis of the hard-shelled mussel. *General and Comparative Endocrinology* **287**, 113347. <https://doi.org/10.1016/j.ygcen.2019.113347>
32. Lü, Z. M., Liu, W., Liu, L. Q., Wang, T. M., Shi, H. L., Ping, H. L., Chi, C. F., Yang, J. W., & Wu, C. W. (2016). Cloning, Characterization, and Expression Profile of Estrogen Receptor in Common Chinese Cuttlefish, Sepiella japonica. Journal of Experimental Zoology Part A: *Ecological Genetics and Physiology* **325**, 181–193. <https://doi.org/10.1002/jez.2011>
33. Lü, Z., Zhu, K., Pang, Z., Liu, L., Jiang, L., Liu, B., Shi, H., Ping, H., Chi, C., & Gong, L. (2019). Identification, characterization and mRNA transcript abundance profiles of estrogen related receptor (ERR) in Sepiella japonica imply its possible involvement in female reproduction. *Animal Reproduction Science* **211**, 106231. <https://doi.org/10.1016/j.anireprosci.2019.106231>
34. Lv, J., Feng, L., Bao, Z., Guo, H., Zhang, Y., Jiao, W., Zhang, L., Wang, S., He, Y., & Hu, X. (2013). Molecular Characterization of RXR (Retinoid X Receptor) Gene Isoforms from the Bivalve Species Chlamys farreri. *PLoS ONE* **8**, 1–9. <https://doi.org/10.1371/journal.pone.0074290>
35. Ma, F., Han, X., An, L., Lei, K., Qi, H., & LeBlanc, G. A. (2019). Freshwater snail Parafossarulus striatulus estrogen receptor: Characteristics and expression profiles under lab and field exposure. *Chemosphere* **220**, 611–619. <https://doi.org/10.1016/j.chemosphere.2018.12.176>
36. Martínez-Paz, P., Morales, M., Sánchez-Argüello, P., Morcillo, G., & Martínez-Guitarte, J. L. (2017). Cadmium in vivo exposure alters stress response and endocrine-related genes in the freshwater snail Physa acuta. New biomarker genes in a new model organism. *Environmental Pollution* **220**, 1488–1497. <https://doi.org/10.1016/j.envpol.2016.10.012>
37. Nagasawa, K., Treen, N., Kondo, R., Otoki, Y., Itoh, N., Rotchell, J. M., & Osada, M. (2015). Molecular characterization of an estrogen receptor and estrogen-related receptor and their autoregulatory capabilities in two Mytilus species. *Gene* **564**, 153–159. <https://doi.org/10.1016/j.gene.2015.03.073>
38. Ni, J., Zeng, Z., & Ke, C. (2013). Sex steroid levels and expression patterns of estrogen receptor gene in the oyster Crassostrea angulata during reproductive cycle. Aquaculture 376–379, 105–116. [https://doi.org/10.1016/j.aquaculture.2012.11.023](https://doi.org/10.1016/j.aquaculture.2012.11.023?utm_source=chatgpt.com)
39. Omran, N. E. S. E. S. (2012). Testosterone, gonadotropins and androgen receptor during spermatogenesis of Biomphalaria alexandrina snails (Pulmonata: Basommatophora). *Reproductive Biology* **12**, 301–308. <https://doi.org/10.1016/j.repbio.2012.10.002>
40. Pang, Z., Lü, Z., Wang, M., Gong, L., Liu, B., Jiang, L., & Liu, L. (2019). Characterization, relative abundances of mRNA transcripts, and subcellular localization of two forms of membrane progestin receptors (mPRs) in the common Chinese cuttlefish, Sepiella japonica. *Animal Reproduction Science* **208**, 106107. <https://doi.org/10.1016/j.anireprosci.2019.106107>
41. Pes, K., Friese, A., Cox, C. J., Laizé, V., & Fernández, I. (2021). Biochemical and molecular responses of the Mediterranean mussel (Mytilus galloprovincialis) to short-term exposure to three commonly prescribed drugs. *Marine Environmental Research*, **168**, 105309. <https://doi.org/10.1016/j.marenvres.2021.105309>
42. Raingeard, D., Bilbao, E., Cancio, I., & Cajaraville, M. P. (2013). Retinoid X receptor (RXR), estrogen receptor (ER) and other nuclear receptors in tissues of the mussel Mytilus galloprovincialis: Cloning and transcription pattern. *Comparative Biochemistry and Physiology Part A: Molecular and Integrative Physiology* **165**, 178–190. <https://doi.org/10.1016/j.cbpa.2013.03.001>
43. Rojas-García, A. E., Robledo-Marenco, M. L., & Barrón-Vivanco, B. S. (2014). Exposure to tributyltin chloride induces penis and vas deferens development and increases RXR expression in females of the purple snail ( Plicopurpura pansa ) Abstract Tributyltin ( TBT ) and its derivatives are widely used as antifouling paints for ships. *ISJ – Invertebrate Survival journal* **11**, 204–212.
44. Rosati, L., Agnese, M., Verderame, M., Aniello, F., Venditti, M., Mita, D. G., Andreuccetti, P., & Prisco, M. (2019a). Morphological and molecular responses in ovaries of Mytilus galloprovincialis collected in two different sites of the Naples Bay. *Journal of Experimental Zoology Part A: Ecological and Integrative Physiology* **331**, 52–60. <https://doi.org/10.1002/jez.2231>
45. Stange, D., & Oehlmann, J. (2012a). Identification of oestrogen-responsive transcripts in Potamopyrgus antipodarum. *Journal of Molluscan Studies* **78**, 337–342. <https://doi.org/10.1093/mollus/eys019>
46. Stange, D., Sieratowicz, A., Horres, R., & Oehlmann, J. (2012b). Freshwater mudsnail (Potamopyrgus antipodarum) estrogen receptor: Identification and expression analysis under exposure to (xeno-)hormones. *Ecotoxicology and Environmental Safety*, **75**, 94–101. <https://doi.org/10.1016/j.ecoenv.2011.09.003>
47. Tan, K., Guo, Z., Zhang, H., Ma, H., Li, S., & Zheng, H. (2021). Carotenoids regulation in polymorphic noble scallops Chlamys nobilis under different light cycle. *Aquaculture* **531**, 735937. <https://doi.org/10.1016/j.aquaculture.2020.735937>
48. Tian, S., Pan, L., & Sun, X. (2013). An investigation of endocrine disrupting effects and toxic mechanisms modulated by benzo[a]pyrene in female scallop Chlamys farreri. *Aquatic Toxicology* **144–145**, 162–171. <https://doi.org/10.1016/j.aquatox.2013.09.031>
49. Tong, Y., Zhang, Y., Huang, J., Xiao, S., Zhang, Y., Li, J., Chen, J., & Yu, Z. (2015). Transcriptomics analysis of crassostrea hongkongensis for the discovery of reproduction-related genes. *PLoS ONE* **10**, 1–24. <https://doi.org/10.1371/journal.pone.0134280>
50. Tran, T. K. A., MacFarlane, G. R., Kong, R. Y. C., O’Connor, W. A., & Yu, R. M. K. (2016). Potential mechanisms underlying estrogen-induced expression of the molluscan estrogen receptor (ER) gene. *Aquatic Toxicology* **179**, 82–94. <https://doi.org/10.1016/j.aquatox.2016.08.015>
51. Urushitani, H., Katsu, Y., Kagechika, H., Sousa, A. C. A., Barroso, C. M., Ohta, Y., Shiraishi, H., Iguchi, T., & Horiguchi, T. (2018). Characterization and comparison of transcriptional activities of the retinoid X receptors by various organotin compounds in three prosobranch gastropods; Thais clavigera, Nucella lapillus and Babylonia japonica. *Aquatic Toxicology* **199**, 103–115. <https://doi.org/10.1016/j.aquatox.2018.03.029>
52. Urushitani, H., Katsu, Y., Ohta, Y., Shiraishi, H., Iguchi, T., & Horiguchi, T. (2013). Cloning and characterization of the retinoic acid receptor-like protein in the rock shell, Thais clavigera. *Aquatic Toxicology* **142–143**, 403–413. <https://doi.org/10.1016/j.aquatox.2013.09.008>
53. Vogeler, S., Bean, T. P., Lyons, B. P., & Galloway, T. S. (2016). Dynamics of nuclear receptor gene expression during Pacific oyster development. *BMC Developmental Biology* **16**, 33. <https://doi.org/10.1186/s12861-016-0129-6>
54. Völker, C., Gräf, T., Schneider, I., Oetken, M., & Oehlmann, J. (2014). Combined effects of silver nanoparticles and 17α-ethinylestradiol on the freshwater mudsnail Potamopyrgus antipodarum. *Environmental Science and Pollution Research* **21**, 10661–10670. <https://doi.org/10.1007/s11356-014-3067-5>
55. Yan, L., Su, J., Wang, Z., Zhang, Y., Yan, X., & Yu, R. (2018). Growth performance and biochemical composition of the oysters Crassostrea sikamea, Crassostrea angulata and their hybrids in southern China. *Aquaculture Research* **49**, 1020–1028. <https://doi.org/10.1111/are.13549>
56. Yang, Y., Pan, L., Zhou, Y., Xu, R., & Li, D. (2020). Benzo[a]pyrene exposure disrupts steroidogenesis and impairs spermatogenesis in diverse reproductive stages of male scallop (Chlamys farreri). *Environmental Research* **191**, 110–125. <https://doi.org/10.1016/j.envres.2020.110125>
57. Zeng, M., Chen, D., Li, Q., Chen, H., & Huang, Q. (2020). Estrogen receptor regulates immune defense by suppressing NF-κB signaling in the Crassostrea hongkongensis. *Fish and Shellfish Immunology* **106**, 796–803. <https://doi.org/10.1016/j.fsi.2020.08.038>
58. Zhang, H., Pan, L., & Zhang, L. (2012). Molecular cloning and characterization of estrogen receptor gene in the Scallop Chlamys farreri: Expression profiles in response to endocrine disrupting chemicals. *Comparative Biochemistry and Physiology Part C: Toxicology and Pharmacology* **156**, 51–57. <https://doi.org/10.1016/j.cbpc.2012.03.007>
59. Zhang, Y., Yu, F., Li, J., Tong, Y., Zhang, Y., & Yu, Z. (2014). The first invertebrate RIG-I-like receptor (RLR) homolog gene in the pacific oyster Crassostrea gigas. *Fish and Shellfish Immunology* **40**, 466–471. <https://doi.org/10.1016/j.fsi.2014.07.029>
60. Zhang, Z., Bai, Q., Xu, X., & Zhang, X. (2021). Effects of the dominance hierarchy on social interactions, cortisol level, HPG-axis activities and reproductive success in the golden cuttlefish Sepia esculenta. *Aquaculture* **533**, 736059. <https://doi.org/10.1016/j.aquaculture.2020.736059>
61. Zheng, B. H., An, L. H., Chang, H., Liu, Y., & Jiang, Z. Q. (2014). Evidence for the presence of sex steroid hormones in Zhikong scallop, Chlamys farreri. *Journal of Steroid Biochemistry and Molecular Biology* **143**, 199–206. <https://doi.org/10.1016/j.jsbmb.2014.03.002>

**3.5 Figure 7**

1. Abidli, S., Castro, L. F. C., Lahbib, Y., Reis-Henriques, M. A., Trigui El Menif, N., & Santos, M. M. (2013). Imposex development in Hexaplex trunculus (Gastropoda: Caenogastropoda) involves changes in the transcription levels of the retinoid X receptor (RXR). *Chemosphere* **93**, 1161–1167. <https://doi.org/10.1016/j.chemosphere.2013.06.054>
2. Agnese, M., Rosati, L., Prisco, M., Borzacchiello, L., Abagnale, L., & Andreuccetti, P. (2019). The expression of estrogen receptors during the Mytilus galloprovincialis ovarian cycle*. Journal of Experimental Zoology Part A: Ecological and Integrative Physiology* **331**, 367–373. <https://doi.org/10.1002/jez.2272>
3. Aquilino, M., Martínez-Guitarte, J. L., García, P., Beltrán, E. M., Fernández, C., & Sánchez-Argüello, P. (2018). Combining the assessment of apical endpoints and gene expression in the freshwater snail Physa acuta after exposure to reclaimed water. *Science of the Total Environment* **642**, 80–189. <https://doi.org/10.1016/j.scitotenv.2018.06.054>
4. Aquilino, M., Sánchez-Argüello, P., Novo, M., & Martínez-Guitarte, J. L. (2019). Effects on tadpole snail gene expression after exposure to vinclozolin. *Ecotoxicology and Environmental Safety* **170**, 568–577. <https://doi.org/10.1016/j.ecoenv.2018.12.015>
5. Balbi, T., Franzellitti, S., Fabbri, R., Montagna, M., Fabbri, E., & Canesi, L. (2016). Impact of bisphenol A (BPA) on early embryo development in the marine mussel Mytilus galloprovincialis: Effects on gene transcription. *Environmental Pollution* **218**, 996–1004. <https://doi.org/10.1016/j.envpol.2016.08.050>
6. Bannister, R., Beresford, N., Granger, D. W., Pounds, N. A., Rand-Weaver, M., White, R., Jobling, S., & Routledge, E. J. (2013). No substantial changes in estrogen receptor and estrogen-related receptor orthologue gene transcription in Marisa cornuarietis exposed to estrogenic chemicals. *Aquatic Toxicology* **140–141**, 19–26. <https://doi.org/10.1016/j.aquatox.2013.05.002>
7. Bouétard, A., Besnard, A. L., Vassaux, D., Lagadic, L., & Coutellec, M. A. (2013). Impact of the redox-cycling herbicide diquat on transcript expression and antioxidant enzymatic activities of the freshwater snail Lymnaea stagnalis. *Aquatic Toxicology* **126**, 256–265. <https://doi.org/10.1016/j.aquatox.2012.11.013>
8. Carter, C. J., Rand, C., Mohammad, I., Lepp, A., Vesprini, N., Wiebe, O., Carlone, R., & Spencer, G. E. (2015). Expression of a retinoic acid receptor (RAR)-like protein in the embryonic and adult nervous system of a protostome species. *Journal of Experimental Zoology Part B: Molecular and Developmental Evolution* **324**, 51–67. <https://doi.org/10.1002/jez.b.22604>
9. De Lisa, E., Paolucci, M., & Di Cosmo, A. (2012). Conservative Nature of Oestradiol Signalling Pathways in the Brain Lobes of Octopus vulgaris Involved in Reproduction, Learning and Motor Coordination. *Journal of Neuroendocrinology* **24**, 275–284. <https://doi.org/10.1111/j.1365-2826.2011.02240.x>
10. Deng, X., Pan, L., Cai, Y., & Jin, Q. (2016). Transcriptomic changes in the ovaries of scallop Chlamys farreri exposed to benzo[a]pyrene. *Genes and Genomics* **38**, 509–518. <https://doi.org/10.1007/s13258-016-0397-3>
11. Ding, M., Jiang, S., Miao, J., & Pan, L. (2021). Possible roles of gonadotropin-releasing hormone (GnRH) and melatonin in the control of gonadal development of clam Ruditapes philippinarum. *Comparative Biochemistry and Physiology Part A : Molecular & Integrative Physiology* **262**, 111059. <https://doi.org/10.1016/j.cbpa.2021.111059>
12. Gesto, M., Ruivo, R., Páscoa, I., André, A., Castro, L. F. C., & Santos, M. M. (2016). Retinoid level dynamics during gonad recycling in the limpet Patella vulgata. General and Comparative Endocrinology, 225, 142–148. <https://doi.org/10.1016/j.ygcen.2015.10.017>
13. Gust, M., Gagné, F., Berlioz-Barbier, A., Besse, J. P., Buronfosse, T., Tournier, M., Tutundjian, R., Garric, J., & Cren-Olivé, C. (2014). Caged mudsnail Potamopyrgus antipodarum (Gray) as an integrated field biomonitoring tool: Exposure assessment and reprotoxic effects of water column contamination. *Water Research* **54**, 222–236. <https://doi.org/10.1016/j.watres.2014.01.057>
14. Huang, W., Wu, Q., Xu, F., Li, L., Li, J., Que, H., & Zhang, G. (2020). Functional characterization of retinoid X receptor with an emphasis on the mediation of organotin poisoning in the Pacific oyster (Crassostrea gigas). *Gene* **753**, 144780. <https://doi.org/10.1016/j.gene.2020.144780>
15. Huang, W., Xu, F., Li, J., Li, L., Que, H., & Zhang, G. (2015). Evolution of a novel nuclear receptor subfamily with emphasis on the member from the Pacific oyster Crassostrea gigas. *Gene* **567**, 164–172. <https://doi.org/10.1016/j.gene.2015.04.082>
16. Huang, W., Xu, F., Qu, T., Zhang, R., Li, L., Que, H., & Zhang, G. (2015). Identification of thyroid hormones and functional characterization of thyroid hormone receptor in the pacific oyster Crassostrea gigas provide insight into evolution of the thyroid hormone system. *PLoS ONE* **10**, 1–20. <https://doi.org/10.1371/journal.pone.0144991>
17. Hultin, C. L., Hallgren, P., & Hansson, M. C. (2016). Estrogen receptor genes in gastropods: Phylogenetic divergence and gene expression responses to a synthetic estrogen. *Comparative Biochemistry and Physiology Part C: Toxicology and Pharmacology* **189**, 17–21. <https://doi.org/10.1016/j.cbpc.2016.07.002>
18. Hultin, C. L., Hallgren, P., Persson, A., & Hansson, M. C. (2014). Identification of an estrogen receptor gene in the natural freshwater snail Bithynia tentaculata. *Gene* **540**, 26–31. <https://doi.org/10.1016/j.gene.2014.02.039>
19. Ip, J. C. H., Leung, P. T. Y., Ho, K. K. Y., Qiu, J. W., & Leung, K. M. Y. (2016). De novo transcriptome assembly of the marine gastropod Reishia clavigera for supporting toxic mechanism studies. *Aquatic Toxicology* **178**, 39–48. <https://doi.org/10.1016/j.aquatox.2016.07.006>
20. Jin, K., Jin, Q., Cai, Z., Huang, B., Wei, L., Zhang, M., Guo, W., Liu, Y., & Wang, X. (2021). Molecular Characterization of Retinoic Acid Receptor CgRAR in Pacific Oyster (Crassostrea gigas). *Frontiers in Physiology* **12**, 1–6. <https://doi.org/10.3389/fphys.2021.666842>
21. Jouaux, A., Blin, J. L., Adeline, B., Heude-Berthelin, C., Sourdaine, P., Mathieu, M., & Kellner, K. (2013). Impact of energy storage strategies on gametogenesis and reproductive effort in diploid and triploid Pacific oysters Crassostrea gigas—Involvement of insulin signaling. *Aquaculture* **388–391**, 173–181. <https://doi.org/10.1016/j.aquaculture.2013.01.009>
22. Juárez, O. E., López-Galindo, L., Pérez-Carrasco, L., Lago-Lestón, A., Rosas, C., Cosmo, A. D., & Galindo-Sánchez, C. E. (2019). Octopus maya white body show sex-specific transcriptomic profiles during the reproductive phase, with high differentiation in signaling pathways. *PLoS ONE* **14**, 1–29. <https://doi.org/10.1371/journal.pone.0216982>
23. Lecomte, V., Noury, P., Tutundjian, R., Buronfosse, T., Garric, J., & Gust, M. (2013). Organic solvents impair life-traits and biomarkers in the New Zealand mudsnail Potamopyrgus antipodarum (Gray) at concentrations below OECD recommendations. *Aquatic Toxicology* **140–141**, 196–203. <https://doi.org/10.1016/j.aquatox.2013.06.006>
24. Lesoway, M. P., & Henry, J. Q. (2021). Retinoids promote penis development in sequentially hermaphroditic snails. *Developmental Biology* **478**, 122–132. <https://doi.org/10.1016/j.ydbio.2021.06.013>
25. Li, H., Liu, J., Huang, X., Wang, D., & Zhang, Z. (2014). Characterization, expression and function analysis of DAX1 gene of scallop (Chlamys farreri jones and preston 1904) during its gametogenesis*. Journal of Ocean University of China* **13**, 696–704. <https://doi.org/10.1007/s11802-014-2299-9>
26. Li, Y. F., Cheng, Y. L., Chen, K., Cheng, Z. Y., Zhu, X., C. R. Cardoso, J., Liang, X., Zhu, Y. T., Power, D. M., & Yang, J. L. (2020). Thyroid hormone receptor: A new player in epinephrine-induced larval metamorphosis of the hard-shelled mussel. *General and Comparative Endocrinology* **287**, 113347. <https://doi.org/10.1016/j.ygcen.2019.113347>
27. Liu, H., Zhang, H., & Zheng, H. (2018). Regulatory roles of sterol regulatory element-binding protein (SREBP) on lipid metabolism in the marine invertebrate *Chlamys nobilis*. *Aquaculture*, 493, 251–257. <https://doi.org/10.1016/j.aquaculture.2018.03.023>
28. Lü, Z. M., Liu, W., Liu, L. Q., Wang, T. M., Shi, H. L., Ping, H. L., Chi, C. F., Yang, J. W., & Wu, C. W. (2016). Cloning, Characterization, and Expression Profile of Estrogen Receptor in Common Chinese Cuttlefish, Sepiella japonica. Journal of Experimental Zoology Part A: *Ecological Genetics and Physiology* **325**, 181–193. <https://doi.org/10.1002/jez.2011>
29. Lü, Z., Zhu, K., Pang, Z., Liu, L., Jiang, L., Liu, B., Shi, H., Ping, H., Chi, C., & Gong, L. (2019). Identification, characterization and mRNA transcript abundance profiles of estrogen related receptor (ERR) in Sepiella japonica imply its possible involvement in female reproduction. *Animal Reproduction Science* **211**, 106231. <https://doi.org/10.1016/j.anireprosci.2019.106231>
30. Lv, J., Feng, L., Bao, Z., Guo, H., Zhang, Y., Jiao, W., Zhang, L., Wang, S., He, Y., & Hu, X. (2013). Molecular Characterization of RXR (Retinoid X Receptor) Gene Isoforms from the Bivalve Species Chlamys farreri. *PLoS ONE* **8**, 1–9. <https://doi.org/10.1371/journal.pone.0074290>
31. Ma, F., Han, X., An, L., Lei, K., Qi, H., & LeBlanc, G. A. (2019). Freshwater snail Parafossarulus striatulus estrogen receptor: Characteristics and expression profiles under lab and field exposure. *Chemosphere* **220**, 611–619. <https://doi.org/10.1016/j.chemosphere.2018.12.176>
32. Martínez-Paz, P., Morales, M., Sánchez-Argüello, P., Morcillo, G., & Martínez-Guitarte, J. L. (2017). Cadmium in vivo exposure alters stress response and endocrine-related genes in the freshwater snail Physa acuta. New biomarker genes in a new model organism. *Environmental Pollution* **220**, 1488–1497. <https://doi.org/10.1016/j.envpol.2016.10.012>
33. Nagasawa, K., Treen, N., Kondo, R., Otoki, Y., Itoh, N., Rotchell, J. M., & Osada, M. (2015). Molecular characterization of an estrogen receptor and estrogen-related receptor and their autoregulatory capabilities in two Mytilus species. *Gene* **564**, 153–159. <https://doi.org/10.1016/j.gene.2015.03.073>
34. Ni, J., Zeng, Z., & Ke, C. (2013). Sex steroid levels and expression patterns of estrogen receptor gene in the oyster Crassostrea angulata during reproductive cycle. Aquaculture 376–379, 105–116. [https://doi.org/10.1016/j.aquaculture.2012.11.023](https://doi.org/10.1016/j.aquaculture.2012.11.023?utm_source=chatgpt.com)
35. Pang, Z., Lü, Z., Wang, M., Gong, L., Liu, B., Jiang, L., & Liu, L. (2019). Characterization, relative abundances of mRNA transcripts, and subcellular localization of two forms of membrane progestin receptors (mPRs) in the common Chinese cuttlefish, Sepiella japonica. *Animal Reproduction Science* **208**, 106107. <https://doi.org/10.1016/j.anireprosci.2019.106107>
36. Pes, K., Friese, A., Cox, C. J., Laizé, V., & Fernández, I. (2021). Biochemical and molecular responses of the Mediterranean mussel (Mytilus galloprovincialis) to short-term exposure to three commonly prescribed drugs. *Marine Environmental Research*, **168**, 105309. <https://doi.org/10.1016/j.marenvres.2021.105309>
37. Raingeard, D., Bilbao, E., Cancio, I., & Cajaraville, M. P. (2013). Retinoid X receptor (RXR), estrogen receptor (ER) and other nuclear receptors in tissues of the mussel Mytilus galloprovincialis: Cloning and transcription pattern. *Comparative Biochemistry and Physiology Part A: Molecular and Integrative Physiology* **165**, 178–190. <https://doi.org/10.1016/j.cbpa.2013.03.001>
38. Rojas-garcía, A. E., Robledo-marenco, M. L., & Barrón-vivanco, B. S. (2014). Exposure to tributyltin chloride induces penis and vas deferens development and increases RXR expression in females of the purple snail ( Plicopurpura pansa ) Abstract Tributyltin ( TBT ) and its derivatives are widely used as antifouling paints for ships. *ISJ – Invertebrate Survival journal* **11**, 204–212.
39. Rosati, L., Agnese, M., Verderame, M., Aniello, F., Venditti, M., Mita, D. G., Andreuccetti, P., & Prisco, M. (2019a). Morphological and molecular responses in ovaries of Mytilus galloprovincialis collected in two different sites of the Naples Bay. *Journal of Experimental Zoology Part A: Ecological and Integrative Physiology* **331**, 52–60. <https://doi.org/10.1002/jez.2231>
40. Shi, Y., Guan, Y., & He, M. (2013). Molecular identification of insulin-related peptide receptor and its potential role in regulating development in Pinctada fucata. *Aquaculture* **408–409**, 118–127. <https://doi.org/10.1016/j.aquaculture.2013.05.038>
41. Stange, D., & Oehlmann, J. (2012a). Identification of oestrogen-responsive transcripts in Potamopyrgus antipodarum. *Journal of Molluscan Studies* **78**, 337–342. <https://doi.org/10.1093/mollus/eys019>
42. Stange, D., Sieratowicz, A., Horres, R., & Oehlmann, J. (2012b). Freshwater mudsnail (Potamopyrgus antipodarum) estrogen receptor: Identification and expression analysis under exposure to (xeno-)hormones. *Ecotoxicology and Environmental Safety*, **75**, 94–101. <https://doi.org/10.1016/j.ecoenv.2011.09.003>
43. Tan, K., Guo, Z., Zhang, H., Ma, H., Li, S., & Zheng, H. (2021). Carotenoids regulation in polymorphic noble scallops Chlamys nobilis under different light cycle. *Aquaculture* **531**, 735937. <https://doi.org/10.1016/j.aquaculture.2020.735937>
44. Tian, S., Pan, L., & Sun, X. (2013). An investigation of endocrine disrupting effects and toxic mechanisms modulated by benzo[a]pyrene in female scallop Chlamys farreri. *Aquatic Toxicology* **144–145**, 162–171. <https://doi.org/10.1016/j.aquatox.2013.09.031>
45. Tong, Y., Zhang, Y., Huang, J., Xiao, S., Zhang, Y., Li, J., Chen, J., & Yu, Z. (2015). Transcriptomics analysis of crassostrea hongkongensis for the discovery of reproduction-related genes. *PLoS ONE* **10**, 1–24. <https://doi.org/10.1371/journal.pone.0134280>
46. Tran, T. K. A., MacFarlane, G. R., Kong, R. Y. C., O’Connor, W. A., & Yu, R. M. K. (2016). Potential mechanisms underlying estrogen-induced expression of the molluscan estrogen receptor (ER) gene. *Aquatic Toxicology* **179**, 82–94. <https://doi.org/10.1016/j.aquatox.2016.08.015>
47. Vogeler, S., Bean, T. P., Lyons, B. P., & Galloway, T. S. (2016). Dynamics of nuclear receptor gene expression during Pacific oyster development. *BMC Developmental Biology* **16**, 33. <https://doi.org/10.1186/s12861-016-0129-6>
48. Völker, C., Gräf, T., Schneider, I., Oetken, M., & Oehlmann, J. (2014). Combined effects of silver nanoparticles and 17α-ethinylestradiol on the freshwater mudsnail Potamopyrgus antipodarum. *Environmental Science and Pollution Research* **21**, 10661–10670. <https://doi.org/10.1007/s11356-014-3067-5>
49. Wang, F., Cai, W., Shi, W., Wu, H., Shen, Q., He, Y., Cui, S., & An, L. (2021). Single molecule real-time sequencing revealing novel insights on the response to estrogen and androgen exposure in freshwater snails. *Aquatic Toxicology* **239**, 105953. <https://doi.org/10.1016/j.aquatox.2021.105953>
50. Wang, Q., & He, M. (2014). Molecular characterization and analysis of a putative 5-HT receptor involved in reproduction process of the pearl oyster Pinctada fucata. *General and Comparative Endocrinology* **204**, 71–79. <https://doi.org/10.1016/j.ygcen.2014.05.010>
51. White, S. H., Carter, C. J., & Magoski, N. S. (2014). A potentially novel nicotinic receptor in Aplysia neuroendocrine cells. *Journal of Neurophysiology* **112**, 446–462. <https://doi.org/10.1152/jn.00796.2013>
52. Xu, R., Pan, L., Yang, Y., & Zhou, Y. (2020). Characterizing transcriptome in female scallop Chlamys farreri provides new insights into the molecular mechanisms of reproductive regulation during ovarian development and spawn. *Gene* **758**, 144967. <https://doi.org/10.1016/j.gene.2020.144967>
53. Yan, L., Su, J., Wang, Z., Zhang, Y., Yan, X., & Yu, R. (2018). Growth performance and biochemical composition of the oysters Crassostrea sikamea, Crassostrea angulata and their hybrids in southern China. *Aquaculture Research* **49**, 1020–1028. <https://doi.org/10.1111/are.13549>
54. Yang, Y., Pan, L., Zhou, Y., Xu, R., & Li, D. (2020). Benzo[a]pyrene exposure disrupts steroidogenesis and impairs spermatogenesis in diverse reproductive stages of male scallop (Chlamys farreri). *Environmental Research* **191**, 110–125. <https://doi.org/10.1016/j.envres.2020.110125>
55. Zeng, M., Chen, D., Li, Q., Chen, H., & Huang, Q. (2020). Estrogen receptor regulates immune defense by suppressing NF-κB signaling in the Crassostrea hongkongensis. *Fish and Shellfish Immunology* **106**, 796–803. <https://doi.org/10.1016/j.fsi.2020.08.038>
56. Zhang, H., Pan, L., & Zhang, L. (2012). Molecular cloning and characterization of estrogen receptor gene in the Scallop Chlamys farreri: Expression profiles in response to endocrine disrupting chemicals. *Comparative Biochemistry and Physiology Part C: Toxicology and Pharmacology* **156**, 51–57. <https://doi.org/10.1016/j.cbpc.2012.03.007>
57. Zhang, M., Wei, H., Liu, T., Li, W., Li, Y., Wang, S., Xing, Q., Hu, X., Zhang, L., & Bao, Z. (2020). Potential GnRH and steroidogenesis pathways in the scallop Patinopecten yessoensis. *Journal of Steroid Biochemistry and Molecular Biology* **204**, 105756. <https://doi.org/10.1016/j.jsbmb.2020.105756>
58. Zhang, Y., Yu, F., Li, J., Tong, Y., Zhang, Y., & Yu, Z. (2014). The first invertebrate RIG-I-like receptor (RLR) homolog gene in the pacific oyster Crassostrea gigas. *Fish and Shellfish Immunology* **40**, 466–471. <https://doi.org/10.1016/j.fsi.2014.07.029>
59. Zhang, Z., Bai, Q., Xu, X., & Zhang, X. (2021). Effects of the dominance hierarchy on social interactions, cortisol level, HPG-axis activities and reproductive success in the golden cuttlefish Sepia esculenta. *Aquaculture* **533**, 736059. <https://doi.org/10.1016/j.aquaculture.2020.736059>
60. Zheng, B. H., An, L. H., Chang, H., Liu, Y., & Jiang, Z. Q. (2014). Evidence for the presence of sex steroid hormones in Zhikong scallop, Chlamys farreri. *Journal of Steroid Biochemistry and Molecular Biology* **143**, 199–206. <https://doi.org/10.1016/j.jsbmb.2014.03.002>

**3.6 Figure 8**

1. Baynes, A., Montagut Pino, G., Duong, G. H., Lockyer, A. E., McDougall, C., Jobling, S., & Routledge, E. J. (2019). Early embryonic exposure of freshwater gastropods to pharmaceutical 5-alpha-reductase inhibitors results in a surprising open-coiled “banana-shaped” shell. *Scientific Reports* **9**, 1–12. https://doi.org/10.1038/s41598-019-52850-x
2. Coelho, I., Lima, D., André, A., Melo, C., Ruivo, R., Reis-Henriques, M. A., Santos, M. M. H., & Castro, L. F. C. (2012). Molecular characterization of Adh3 from the mollusc Nucella lapillus: Tissue gene expression after tributyltin and retinol exposure. *Journal of Molluscan Studies* **78**, 343–348. https://doi.org/10.1093/mollus/eys018
3. Deng, X., Pan, L., Cai, Y., & Jin, Q. (2016). Transcriptomic changes in the ovaries of scallop Chlamys farreri exposed to benzo[a]pyrene. *Genes and Genomics* **38**, 509–518. <https://doi.org/10.1007/s13258-016-0397-3>
4. Ding, M., Jiang, S., Miao, J., & Pan, L. (2021). Possible roles of gonadotropin-releasing hormone (GnRH) and melatonin in the control of gonadal development of clam Ruditapes philippinarum. *Comparative Biochemistry and Physiology Part A : Molecular & Integrative Physiology* **262**, 111059. <https://doi.org/10.1016/j.cbpa.2021.111059>
5. Huang, W., Xu, F., Qu, T., Li, L., Que, H., & Zhang, G. (2015). Iodothyronine deiodinase gene analysis of the Pacific oyster Crassostrea gigas reveals possible conservation of thyroid hormone feedback regulation mechanism in mollusks. *Chinese Journal of Oceanology and Limnology* **33**, 997–1006. <https://doi.org/10.1007/s00343-015-4300-x>
6. Ip, J. C. H., Leung, P. T. Y., Ho, K. K. Y., Qiu, J. W., & Leung, K. M. Y. (2016). De novo transcriptome assembly of the marine gastropod Reishia clavigera for supporting toxic mechanism studies. *Aquatic Toxicology* **178**, 39–48. <https://doi.org/10.1016/j.aquatox.2016.07.006>
7. Jiang, S., Miao, J., Wang, X., Liu, P., & Pan, L. (2019). Inhibition of growth in juvenile manila clam Ruditapes philippinarum: Potential adverse outcome pathway of TBBPA. *Chemosphere* **224**, 588–596. <https://doi.org/10.1016/j.chemosphere.2019.02.157>
8. Liu, P., Miao, J., Song, Y., Pan, L., & Yin, P. (2017). Effects of 2,2’,4,4’-tetrabromodipheny ether (BDE-47) on gonadogenesis of the manila clam Ruditapes philippinarum. *Aquatic Toxicology* **193**, 178–186. <https://doi.org/10.1016/j.aquatox.2017.10.022>
9. Prisco, M., Agnese, M., De Marino, A., Andreuccetti, P., & Rosati, L. (2017). Spermatogenic Cycle and Steroidogenic Control of Spermatogenesis in Mytilus galloprovincialis Collected in the Bay of Naples. *Anatomical Record* **300**, 1881–1894. <https://doi.org/10.1002/ar.23626>
10. Rosati, L., Agnese, M., Abagnale, L., Aniello, F., Andreuccetti, P., & Prisco, M. (2019b). The Mussel Mytilus galloprovincialis in the Bay of Naples: New Insights on Oogenic Cycle and Its Hormonal Control. *Anatomical Record* **302**, 1039–1049. <https://doi.org/10.1002/ar.24075>
11. Rothwell, C. M., Simmons, J., Peters, G., & Spencer, G. E. (2014). Novel interactive effects of darkness and retinoid signaling in the ability to form long-term memory following aversive operant conditioning. *Neurobiology of Learning and Memory* **114**, 251–263. <https://doi.org/10.1016/j.nlm.2014.07.007>
12. Song, Y., Miao, J., Pan, L., & Wang, X. (2016). Exposure to2,2’,4,4’-tetrabromodiphenyl ether (BDE-47) alters thyroid hormone levels and thyroid hormone-regulated gene transcription in manila clam Ruditapes philippinarum. *Chemosphere*, **152**, 10–16. <https://doi.org/10.1016/j.chemosphere.2016.02.049>
13. Thitiphuree, T., Nagasawa, K., & Osada, M. (2019). Molecular identification of steroidogenesis-related genes in scallops and their potential roles in gametogenesis. *Journal of Steroid Biochemistry and Molecular Biology* **186**, 22–33. <https://doi.org/10.1016/j.jsbmb.2018.09.004>
14. Tian, S., Pan, L., & Sun, X. (2013). An investigation of endocrine disrupting effects and toxic mechanisms modulated by benzo[a]pyrene in female scallop Chlamys farreri. *Aquatic Toxicology* **144–145**, 162–171. <https://doi.org/10.1016/j.aquatox.2013.09.031>
15. Tong, Y., Zhang, Y., Huang, J., Xiao, S., Zhang, Y., Li, J., Chen, J., & Yu, Z. (2015). Transcriptomics analysis of crassostrea hongkongensis for the discovery of reproduction-related genes. *PLoS ONE* **10**, 1–24. <https://doi.org/10.1371/journal.pone.0134280>
16. Vöcking, O., Leclère, L., & Hausen, H. (2021). The rhodopsin-retinochrome system for retinal re-isomerization predates the origin of cephalopod eyes. *BMC Ecology and Evolution* **21**, 1–14. <https://doi.org/10.1186/s12862-021-01939-x>
17. Wang, S., Ji, C., Li, F., Zhan, J., Sun, T., Tang, J., & Wu, H. (2021). Tetrabromobisphenol A induced reproductive endocrine-disrupting effects in mussel Mytilus galloprovincialis. *Journal of Hazardous Materials* **416**, 126228. <https://doi.org/10.1016/j.jhazmat.2021.126228>
18. Xu, R., Pan, L., Yang, Y., & Zhou, Y. (2020). Characterizing transcriptome in female scallop Chlamys farreri provides new insights into the molecular mechanisms of reproductive regulation during ovarian development and spawn. *Gene* **758**, 144967. <https://doi.org/10.1016/j.gene.2020.144967>
19. Yang, Y., Pan, L., Zhou, Y., Xu, R., & Li, D. (2020). Benzo[a]pyrene exposure disrupts steroidogenesis and impairs spermatogenesis in diverse reproductive stages of male scallop (Chlamys farreri). *Environmental Research* **191**, 110–125. <https://doi.org/10.1016/j.envres.2020.110125>
20. Zhang, M., Wei, H., Liu, T., Li, W., Li, Y., Wang, S., Xing, Q., Hu, X., Zhang, L., & Bao, Z. (2020). Potential GnRH and steroidogenesis pathways in the scallop Patinopecten yessoensis. *Journal of Steroid Biochemistry and Molecular Biology* **204**, 105756. <https://doi.org/10.1016/j.jsbmb.2020.105756>

**3.7 Figure 9**

1. Aquilino, M., Sánchez-Argüello, P., Novo, M., & Martínez-Guitarte, J. L. (2019). Effects on tadpole snail gene expression after exposure to vinclozolin. *Ecotoxicology and Environmental Safety* **170**, 568–577. <https://doi.org/10.1016/j.ecoenv.2018.12.015>
2. Coelho, I., Lima, D., André, A., Melo, C., Ruivo, R., Reis-Henriques, M. A., Santos, M. M. H., & Castro, L. F. C. (2012). Molecular characterization of Adh3 from the mollusc Nucella lapillus: Tissue gene expression after tributyltin and retinol exposure. *Journal of Molluscan Studies* **78**, 343–348. <https://doi.org/10.1093/mollus/eys018>
3. Cubero-Leon, E., Puinean, A. M., Labadie, P., Ciocan, C., Itoh, N., Kishida, M., Osada, M., Minier, C., Hill, E. M., & Rotchell, J. M. (2012). Two CYP3A-like genes in the marine mussel Mytilus edulis: mRNA expression modulation following short-term exposure to endocrine disruptors. Marine Environmental Research 74, 32–39. [https://doi.org/10.1016/j.marenvres.2011.11.012](https://doi.org/10.1016/j.marenvres.2011.11.012?utm_source=chatgpt.com)
4. De Lisa, E., Paolucci, M., & Di Cosmo, A. (2012). Conservative Nature of Oestradiol Signalling Pathways in the Brain Lobes of Octopus vulgaris Involved in Reproduction, Learning and Motor Coordination. *Journal of Neuroendocrinology* **24**, 275–284. <https://doi.org/10.1111/j.1365-2826.2011.02240.x>
5. Deng, X., Pan, L., Cai, Y., & Jin, Q. (2016). Transcriptomic changes in the ovaries of scallop Chlamys farreri exposed to benzo[a]pyrene. *Genes and Genomics* **38**, 509–518. <https://doi.org/10.1007/s13258-016-0397-3>
6. Ding, M., Jiang, S., Miao, J., & Pan, L. (2021). Possible roles of gonadotropin-releasing hormone (GnRH) and melatonin in the control of gonadal development of clam Ruditapes philippinarum. *Comparative Biochemistry and Physiology Part A : Molecular & Integrative Physiology* **262**, 111059. <https://doi.org/10.1016/j.cbpa.2021.111059>
7. Huang, W., Xu, F., Qu, T., Li, L., Que, H., & Zhang, G. (2015). Iodothyronine deiodinase gene analysis of the Pacific oyster Crassostrea gigas reveals possible conservation of thyroid hormone feedback regulation mechanism in mollusks. *Chinese Journal of Oceanology and Limnology* **33**, 997–1006. <https://doi.org/10.1007/s00343-015-4300-x>
8. Ip, J. C. H., Leung, P. T. Y., Ho, K. K. Y., Qiu, J. W., & Leung, K. M. Y. (2016). De novo transcriptome assembly of the marine gastropod Reishia clavigera for supporting toxic mechanism studies. *Aquatic Toxicology* **178**, 39–48. <https://doi.org/10.1016/j.aquatox.2016.07.006>
9. Jiang, S., Miao, J., Wang, X., Liu, P., & Pan, L. (2019). Inhibition of growth in juvenile manila clam Ruditapes philippinarum: Potential adverse outcome pathway of TBBPA. *Chemosphere* **224**, 588–596. <https://doi.org/10.1016/j.chemosphere.2019.02.157>
10. Juárez, O. E., López-Galindo, L., Pérez-Carrasco, L., Lago-Lestón, A., Rosas, C., Cosmo, A. D., & Galindo-Sánchez, C. E. (2019). Octopus maya white body show sex-specific transcriptomic profiles during the reproductive phase, with high differentiation in signaling pathways. *PLoS ONE* **14**, 1–29. <https://doi.org/10.1371/journal.pone.0216982>
11. Lesoway, M. P., & Henry, J. Q. (2021). Retinoids promote penis development in sequentially hermaphroditic snails. Developmental Biology 478, 122–132. https://doi.org/10.1016/j.ydbio.2021.06.013
12. Lima, D., MacHado, A., Reis-Henriques, M. A., Rocha, E., Santos, M. M., & Castro, L. F. C. (2013). Cloning and expression analysis of the 17β hydroxysteroid dehydrogenase type 12 (HSD17B12) in the neogastropod Nucella lapillus. *Journal of Steroid Biochemistry and Molecular Biology*, **134**, 8–14. <https://doi.org/10.1016/j.jsbmb.2012.10.005>
13. Lin, C., Guo, C., Zhu, X., Wang, D., Xu, J., & Xu, S. (2019). Ovarian transcriptome analysis of Mactra chinensis provides insights into genes expressed during the intermediate and ripening stages. *Animal Reproduction Science* **208**, 106078. <https://doi.org/10.1016/j.anireprosci.2019.05.007>
14. Liu, J., Zhang, Z., Ma, X., Liang, S., & Yang, D. (2014b). Characteristics of 17β-hydroxysteroid dehydrogenase 8 and its potential role in gonad of Zhikong scallop Chlamys farreri. *Journal of Steroid Biochemistry and Molecular Biology* **141**, 77–86. <https://doi.org/10.1016/j.jsbmb.2014.01.008>
15. Liu, P., Miao, J., Song, Y., Pan, L., & Yin, P. (2017). Effects of 2,2’,4,4’-tetrabromodipheny ether (BDE-47) on gonadogenesis of the manila clam Ruditapes philippinarum. *Aquatic Toxicology* **193**, 178–186. <https://doi.org/10.1016/j.aquatox.2017.10.022>
16. Meng, X., Li, F., Wang, X., Liu, J., Ji, C., & Wu, H. (2019). Combinatorial immune and stress response, cytoskeleton and signal transduction effects of graphene and triphenyl phosphate (TPP) in mussel Mytilus galloprovincialis. *Journal of Hazardous Materials* **378**, 120778. https://doi.org/10.1016/j.jhazmat.2019.120778
17. Rothwell, C. M., Simmons, J., Peters, G., & Spencer, G. E. (2014). Novel interactive effects of darkness and retinoid signaling in the ability to form long-term memory following aversive operant conditioning. *Neurobiology of Learning and Memory* **114**, 251–263. <https://doi.org/10.1016/j.nlm.2014.07.007>
18. Song, Y., Miao, J., Cai, Y., & Pan, L. (2015). Molecular cloning, characterization, and expression analysis of a gonadotropin-releasing hormone-like cDNA in the clam, Ruditapes philippinarum. *Comparative Biochemistry and Physiology Part B: Biochemistry and Molecular Biology* **189**, 47–54. <https://doi.org/10.1016/j.cbpb.2015.07.005>
19. Song, Y., Miao, J., Pan, L., & Wang, X. (2016). Exposure to2,2’,4,4’-tetrabromodiphenyl ether (BDE-47) alters thyroid hormone levels and thyroid hormone-regulated gene transcription in manila clam Ruditapes philippinarum. *Chemosphere*, **152**, 10–16. <https://doi.org/10.1016/j.chemosphere.2016.02.049>
20. Strehse, J. S., Brenner, M., Kisiela, M., & Maser, E. (2020). The explosive trinitrotoluene (TNT) induces gene expression of carbonyl reductase in the blue mussel (Mytilus spp.): A new promising biomarker for sea dumped war relicts? *Archives of Toxicology* **94**, 4043–4054. <https://doi.org/10.1007/s00204-020-02931-y>
21. Takishita, K., Takaki, Y., Chikaraishi, Y., Ikuta, T., Ozawa, G., Yoshida, T., Ohkouchi, N., & Fujikura, K. (2017). Genomic evidence thatmethanotrophic endosymbionts likely providedeep-sea bathymodiolus musselswith a sterol intermediate in cholesterol biosynthesis. *Genome Biology and Evolution* **9**, 1148–1160. <https://doi.org/10.1093/gbe/evx082>
22. Thitiphuree, T., Nagasawa, K., & Osada, M. (2019). Molecular identification of steroidogenesis-related genes in scallops and their potential roles in gametogenesis. *Journal of Steroid Biochemistry and Molecular Biology* **186**, 22–33. <https://doi.org/10.1016/j.jsbmb.2018.09.004>
23. Tian, S., Pan, L., & Sun, X. (2013). An investigation of endocrine disrupting effects and toxic mechanisms modulated by benzo[a]pyrene in female scallop Chlamys farreri. *Aquatic Toxicology* **144–145**, 162–171. <https://doi.org/10.1016/j.aquatox.2013.09.031>
24. Tian, S., Pan, L., & Zhang, H. (2014). Identification of a CYP3A-like gene and CYPs mRNA expression modulation following exposure to benzo[a]pyrene in the bivalve mollusk Chlamys farreri. *Marine Environmental Research* **94**, 7–15. <https://doi.org/10.1016/j.marenvres.2013.11.001>
25. Tong, Y., Zhang, Y., Huang, J., Xiao, S., Zhang, Y., Li, J., Chen, J., & Yu, Z. (2015). Transcriptomics analysis of crassostrea hongkongensis for the discovery of reproduction-related genes. *PLoS ONE* **10**, 1–24. <https://doi.org/10.1371/journal.pone.0134280>
26. Vöcking, O., Leclère, L., & Hausen, H. (2021). The rhodopsin-retinochrome system for retinal re-isomerization predates the origin of cephalopod eyes. *BMC Ecology and Evolution* **21**, 1–14. <https://doi.org/10.1186/s12862-021-01939-x>
27. Wang, T., Kong, H., Shang, Y., Dupont, S., Peng, J., Wang, X., Deng, Y., Hu, M., & Wang, Y. (2021). Ocean acidification but not hypoxia alters the gonad performance in the thick shell mussel Mytilus coruscus. *Marine Pollution Bulletin* **167**, 112 282. <https://doi.org/10.1016/j.marpolbul.2021.112282>
28. Xu, R., Pan, L., Yang, Y., & Zhou, Y. (2020). Characterizing transcriptome in female scallop Chlamys farreri provides new insights into the molecular mechanisms of reproductive regulation during ovarian development and spawn. *Gene* **758**, 144967. <https://doi.org/10.1016/j.gene.2020.144967>
29. Yang, Y., Pan, L., Zhou, Y., Xu, R., & Li, D. (2020). Benzo[a]pyrene exposure disrupts steroidogenesis and impairs spermatogenesis in diverse reproductive stages of male scallop (Chlamys farreri). *Environmental Research* **191**, 110–125. <https://doi.org/10.1016/j.envres.2020.110125>
30. Zhai, H. N., Zhou, J., & Cai, Z. H. (2012). Cloning, characterization, and expression analysis of a putative 17 beta-hydroxysteroid dehydrogenase 11 in the abalone, Haliotis diversicolor supertexta. *Journal of Steroid Biochemistry and Molecular Biology* **130**, 57–63. <https://doi.org/10.1016/j.jsbmb.2011.12.013>
31. Zhang, M., Wei, H., Liu, T., Li, W., Li, Y., Wang, S., Xing, Q., Hu, X., Zhang, L., & Bao, Z. (2020). Potential GnRH and steroidogenesis pathways in the scallop Patinopecten yessoensis. *Journal of Steroid Biochemistry and Molecular Biology* **204**, 105756. <https://doi.org/10.1016/j.jsbmb.2020.105756>
32. Zhang, Y., Wang, Q., Ji, Y., Zhang, Q., Wu, H., Xie, J., & Zhao, J. (2014). Identification and mRNA expression of two 17β-hydroxysteroid dehydrogenase genes in the marine mussel Mytilus galloprovincialis following exposure to endocrine disrupting chemicals. *Environmental Toxicology and Pharmacology* **37**, 1243–1255. <https://doi.org/10.1016/j.etap.2014.04.027>
33. Zhang, Z., Bai, Q., Xu, X., & Zhang, X. (2021). Effects of the dominance hierarchy on social interactions, cortisol level, HPG-axis activities and reproductive success in the golden cuttlefish Sepia esculenta. *Aquaculture* **533**, 736059. <https://doi.org/10.1016/j.aquaculture.2020.736059>

**3.8 Figure 10**

1. Afsar, N., Siddiqui, G., Rasheed, M., Ahmed, V. U., & Khan, A. (2012). GC-MS analysis of fatty acids (FAs) of prosobranch gastropod species thais carinifera from pakistan coast (North Arabian Sea). Journal of the Chemical Society of Pakistan 34, 565–569
2. Dagorn, F., Couzinet-Mossion, A., Kendel, M., Beninger, P. G., Rabesaotra, V., Barnathan, G., & Wielgosz-Collin, G. (2016). Exploitable lipids and fatty acids in the invasive oyster Crassostrea gigas on the French Atlantic coast. Marine Drugs 14(6), 104. [https://doi.org/10.3390/md14060104](https://doi.org/10.3390/md14060104?utm_source=chatgpt.com)
3. Fiorini, R., Ventrella, V., Trombetti, F., Fabbri, M., Pagliarani, A., & Nesci, S. (2019). Lipid-protein interactions in mitochondrial membranes from bivalve mollusks: Molecular strategies in different species. Comparative Biochemistry and Physiology Part B: Biochemistry & Molecular Biology 227, 12–20. https://doi.org/10.1016/j.cbpb.2018.08.010
4. Hurtado, M. A., Racotta, I. S., Arcos, F., Morales-Bojórquez, E., Moal, J., Soudant, P., & Palacios, E. (2012). Seasonal variations of biochemical, pigment, fatty acid, and sterol compositions in female Crassostrea corteziensis oysters in relation to the reproductive cycle. Comparative Biochemistry and Physiology Part B: Biochemistry and Molecular Biology 163, 172–183.
5. Kawashima, H., Ohnishi, M., & Ogawa, S. (2013). Distribution of unusual cholesterol precursors, 4-Methyl- and 4, 4-dimethylsterols with Δ8 unsaturation, in gonads of marine archaeogastropods. Journal of Oleo Science 62, 465–470. https://doi.org/10.5650/jos.62.465https://doi.org/10.1016/j.cbpb.2012.05.011
6. Takishita, K., Takaki, Y., Chikaraishi, Y., Ikuta, T., Ozawa, G., Yoshida, T., Ohkouchi, N., & Fujikura, K. (2017). Genomic evidence thatmethanotrophic endosymbionts likely providedeep-sea bathymodiolus musselswith a sterol intermediate in cholesterol biosynthesis. Genome Biology and Evolution 9, 1148–1160. https://doi.org/10.1093/gbe/evx082

**3.9 Figure 11**

1. Abd El-Atti, M. S., El-Sayed, A. S. A., & Said, R. M. (2020). Usage of pharmaceutical contraceptive drug for controlling Eobania vermiculata snails by baits technique. Heliyon 6, e05630. https://doi.org/10.1016/j.heliyon.2020.e05630
2. Abdel-Hamid, H., & Mekawey, A. A. I. (2014). Biological and hematological responses of Biomphalaria alexandrina to mycobiosynthsis silver nanoparticles. Journal of the Egyptian Society of Parasitology 44, 627–637. https://doi.org/10.12816/0007866
3. Abidli, S., Santos, M. M. H., Lahbib, Y., Castro, L. F. C., Reis-Henriques, M. A., & Trigui El Menif, N. (2012). Tributyltin (TBT) effects on Hexaplex trunculus and Bolinus brandaris (Gastropoda: Muricidae): Imposex induction and sex hormone levels insights. Ecological Indicators 13, 13–21. https://doi.org/10.1016/j.ecolind.2011.05.001
4. Abu El Einin, H. M., Ali, R. E., Gad El-Karim, R. M., Youssef, A. A., Abdel-Hamid, H., & Habib, M. R. (2019). Biomphalaria alexandrina: A model organism for assessing the endocrine disrupting effect of 17β-estradiol. Environmental Science and Pollution Research 26, 23328–23336. <https://doi.org/10.1007/s11356-019-05586-0>
5. Afsar, N., Siddiqui, G., Rasheed, M., Ahmed, V. U., & Khan, A. (2012). GC-MS analysis of fatty acids (FAs) of prosobranch gastropod species *Thais carinifera* from Pakistan Coast (North Arabian Sea). *Journal of the Chemical Society of Pakistan*, 34(3), 565.
6. Akcha, F., Barranger, A., Bachère, E., Berthelin, C. H., Piquemal, D., Alonso, P., Sallan, R. R., Dimastrogiovanni, G., Porte, C., Menard, D., Szczybelski, A., Benabdelmouna, A., Auffret, M., Rouxel, J., & Burgeot, T. (2016). Effects of an environmentally relevant concentration of diuron on oyster genitors during gametogenesis: Responses of early molecular and cellular markers and physiological impacts. Environmental Science and Pollution Research 23, 8008–8020. https://doi.org/10.1007/s11356-015-5969-2
7. Avila-Poveda, O. H., Montes-Pérez, R. C., Benitez-Villalobos, F., & Rosas, C. (2013). Development and validation of a solid-phase radioimmunoassay for measuring progesterone and testosterone in octopus gonad extracts. Malacologia 56, 121–134. https://doi.org/10.4002/040.056.0209
8. Avila-Poveda, O. H., Montes-Pérez, R. C., Koueta, N., Benítez-Villalobos, F., Ramírez-Pérez, J. S., Jimenez-Gutierrez, L. R., & Rosas, C. (2015). Seasonal changes of progesterone and testosterone concentrations throughout gonad maturation stages of the Mexican octopus, Octopus maya (Octopodidae: Octopus). Molluscan Research 35, 161–172. https://doi.org/10.1080/13235818.2015.1045055
9. Baynes, A., Montagut Pino, G., Duong, G. H., Lockyer, A. E., McDougall, C., Jobling, S., & Routledge, E. J. (2019). Early embryonic exposure of freshwater gastropods to pharmaceutical 5-alpha-reductase inhibitors results in a surprising open-coiled “banana-shaped” shell. Scientific Reports 9, 1–12. https://doi.org/10.1038/s41598-019-52850-x
10. Binder, A. R. D., Pfaffl, M. W., Hiltwein, F., JuergenGeis, J., & Beggel, S. (2019). Does environmental stress affect cortisol biodistribution in freshwatermussels? Conservation Physiology 7, 1–10. https://doi.org/10.1093/conphys/coz101
11. Cappello, T., Fernandes, D., Maisano, M., Casano, A., Bonastre, M., Bebianno, M. J., Mauceri, A., Fasulo, S., & Porte, C. (2017). Sex steroids and metabolic responses in mussels Mytilus galloprovincialis exposed to drospirenone. Ecotoxicology and Environmental Safety 143, 166–172. https://doi.org/10.1016/j.ecoenv.2017.05.031
12. Cheour, M. K., Elgharsalli, R., Benmessaoud, R., & Aloui-Bejaoui, N. (2014). Variation of steroid concentrations during the reproductive cycle of the snail Osilinus articulatus in the Kerkennah Islands (Gulf of Gabes, Tunisia). Cahiers de Biologie Marine 55, 191-199 NP – 9.
13. Chong Sánchez, F., Enríquez Díaz, M. & Aldana Aranda, D. (2020). Sex hormones in *Strombus pugilis* (Mollusca: Gastropoda) in different gonadal stages. *Bulletin of Marine Science* 96, 679–694.
14. Chong Sánchez, F., Enriquez Díaz, M., Murillo Rodríguez, E., & Aldana Aranda, D. (2019). First use of a non-invasive technique for determination of sex hormones in the queen conch Lobatus gigas, Mollusca Gastropoda. Aquaculture International 27, 437–448. <https://doi.org/10.1007/s10499-018-0336-1>
15. Dagorn, F., Couzinet-Mossion, A., Kendel, M., Beninger, P. G., Rabesaotra, V., Barnathan, G., & Wielgosz-Collin, G. (2016). Exploitable lipids and fatty acids in the invasive oyster Crassostrea gigas on the French Atlantic coast. Marine Drugs 14, 1–12. https://doi.org/10.3390/md14060104
16. Deng, X., Pan, L., Cai, Y., & Jin, Q. (2016). Transcriptomic changes in the ovaries of scallop Chlamys farreri exposed to benzo[a]pyrene. Genes and Genomics 38, 509–518. https://doi.org/10.1007/s13258-016-0397-3
17. Dimastrogiovanni, G., Fernandes, D., Bonastre, M., & Porte, C. (2015). Progesterone is actively metabolized to 5α-pregnane-3,20-dione and 3β-hydroxy-5α-pregnan-20-one by the marine mussel Mytilus galloprovincialis. Aquatic Toxicology 165, 93–100. https://doi.org/10.1016/j.aquatox.2015.05.018
18. Ding, M., Jiang, S., Miao, J., & Pan, L. (2021). Possible roles of gonadotropin-releasing hormone (GnRH) and melatonin in the control of gonadal development of clam Ruditapes philippinarum. Comparative Biochemistry and Physiology Part A : Molecular & Integrative Physiology 262, 111059. <https://doi.org/10.1016/j.cbpa.2021.111059>
19. Dokmak, H. A. A. S., El-Emam, M. A., Mossalem, H. S., El-Tayeb, T. A., & Khalil, M. T. (2021). Impact of carbamide perhydrate on the snail bulinus truncatus, the intermediate host of schistosoma haematobium. Egyptian Journal of Aquatic Biology and Fisheries 25, 85–99. <https://doi.org/10.21608/ejabf.2021.172770>
20. Fiorini, R., Ventrella, V., Trombetti, F., Fabbri, M., Pagliarani, A., & Nesci, S. (2019). Lipid–protein interactions in mitochondrial membranes from bivalve mollusks: Molecular strategies in different species. *Comparative Biochemistry and Physiology Part B: Biochemistry and Molecular Biology*, 227, 12–20. <https://doi.org/10.1016/j.cbpb.2018.08.010>
21. Giusti, A., Ducrot, V., Joaquim -Justo, C., & Lagadic, L. (2013). Testosterone levels and fecundity in the hermaphroditic aquatic snail Lymnaea stagnalis exposed to testosterone and endocrine disruptors. Environmental Toxicology and Chemistry 32, 1740–1745. https://doi.org/10.1002/etc.2234
22. Goto, Y., Kajiwara, M., Yanagisawa, Y., Hirose, H., Yoshimi, T., Umemura, M., Nakano, H., Takahashi, S., Shida, Y., Iguchi, T., Takahashi, Y., & Miura, T. (2012). Detection of vertebrate-type steroid hormones and their converting activities in the neogastropod Thais clavigera (Küster, 1858). Journal of Molluscan Studies 78, 197–204. https://doi.org/10.1093/mollus/eys001
23. Gust, M., Gagné, F., Berlioz-Barbier, A., Besse, J. P., Buronfosse, T., Tournier, M., Tutundjian, R., Garric, J., & Cren-Olivé, C. (2014). Caged mudsnail Potamopyrgus antipodarum (Gray) as an integrated field biomonitoring tool: Exposure assessment and reprotoxic effects of water column contamination. Water Research 54, 222–236. https://doi.org/10.1016/j.watres.2014.01.057
24. Habib, M. R., Ghoname, S. I., Ali, R. E., El-Karim, R. M. G., Youssef, A. A., Croll, R. P., & Miller, M. W. (2020). Biochemical and apoptotic changes in the nervous and ovotestis tissues of Biomphalaria alexandrina following infection with Schistosoma mansoni. Experimental Parasitology 213, 107887. https://doi.org/10.1016/j.exppara.2020.107887
25. Halem, Z. M., Ross, D. J., & Cox, R. L. (2014). Evidence for intraspecific endocrine disruption of Geukensia demissa (Atlantic ribbed mussel) in an urban watershed. Comparative Biochemistry and Physiology Part A : Molecular and Integrative Physiology 175, 1–6. https://doi.org/10.1016/j.cbpa.2014.04.016
26. Hallmann, A., Konieczna, L., Swiezak, J., Milczarek, R., & Smolarz, K. (2019). Aromatisation of steroids in the bivalve *Mytilus trossulus*. *PeerJ*, 7, e6953. <https://doi.org/10.7717/peerj.6953>
27. Hallmann, A., Smolarz, K., Konieczna, L., Zabrzańska, S., Belka, M., & Baczek, T. (2016). LC-MS measurment of free steroids in mussels (Mytilus trossulus) from the southern Baltic Sea. Journal of Pharmaceutical and Biomedical Analysis 117, 311–315. <https://doi.org/10.1016/j.jpba.2015.09.013>
28. Hurtado, M. A., Racotta, I. S., Arcos, F., Morales-Bojórquez, E., Moal, J., Soudant, P., & Palacios, E. (2012). Seasonal variations of biochemical, pigment, fatty acid, and sterol compositions in female Crassostrea corteziensis oysters in relation to the reproductive cycle. *Comparative Biochemistry and Physiology Part B: Biochemistry and Molecular Biology* **163**, 172–183. <https://doi.org/10.1016/j.cbpb.2012.05.011>
29. Ibrahim, A. M., & Ghoname, S. I. (2018). Molluscicidal impacts of Anagallis arvensis aqueous extract on biological, hormonal, histological and molecular aspects of Biomphalaria alexandrina snails. Experimental Parasitology 192, 36–41. https://doi.org/10.1016/j.exppara.2018.07.014
30. Ibrahim, A. M., & Sayed, D. A. (2019). Toxicological impact of oxyfluorfen 24% herbicide on the reproductive system, antioxidant enzymes, and endocrine disruption of Biomphalaria alexandrina (Ehrenberg, 1831) snails. Environmental Science and Pollution Research 26, 7960–7968. https://doi.org/10.1007/s11356-019-04251-w
31. Ip, J. C., Leung, P. T., Ho, K. K., Qiu, J. W., & Leung, K. M. (2016). *De novo* transcriptome assembly of the marine gastropod *Reishia clavigera* for supporting toxic mechanism studies. *Aquatic Toxicology*, 178, 39–48. <https://doi.org/10.1016/j.aquatox.2016.07.006>
32. Kandil, M. A., Eweis, E. A., Mobarak Soha, A., & Nada Abbas, M. T. (2020). Effects of chitosan and emamectin benzoate on the reproductive system of Eobania vermiculata (Muller) land snails. Egyptian Journal of Biological Pest Control 30, 1–8. <https://doi.org/10.1186/s41938-020-00224-1>
33. Knigge, T., Dahboul, F., Alain, D., & Monsinjon, T. (2015). The gametogenic cycle and oestradiol levels in the zebra mussel Dreissena polymorpha: A 1-year study. Journal of Molluscan Studies 81, 58–65. https://doi.org/10.1093/mollus/eyu056
34. Lazzara, R., Blázquez, M., Porte, C., & Barata, C. (2012). Low environmental levels of fluoxetine induce spawning and changes in endogenous estradiol levels in the zebra mussel Dreissena polymorpha. Aquatic Toxicology 106–107, 123–130. https://doi.org/10.1016/j.aquatox.2011.11.003
35. Lecomte, V., Noury, P., Tutundjian, R., Buronfosse, T., Garric, J., & Gust, M. (2013). Organic solvents impair life-traits and biomarkers in the New Zealand mudsnail Potamopyrgus antipodarum (Gray) at concentrations below OECD recommendations. Aquatic Toxicology 140–141, 196–203. https://doi.org/10.1016/j.aquatox.2013.06.006
36. Lin, C., Guo, C., Zhu, X., Wang, D., Xu, J., & Xu, S. (2019). Ovarian transcriptome analysis of Mactra chinensis provides insights into genes expressed during the intermediate and ripening stages. Animal Reproduction Science 208, 106078. https://doi.org/10.1016/j.anireprosci.2019.05.007
37. Liu, J., Zhang, Z., Zhang, L., Liu, X., Yang, D., & Ma, X. (2014a). Variations of estradiol-17β and testosterone levels correlated with gametogenesis in the gonad of Zhikong scallop (Chlamys farreri) during annual reproductive cycle. Canadian Journal of Zoology 92, 195–204. <https://doi.org/10.1139/cjz-2013-0202>
38. Liu, P., Miao, J., Song, Y., Pan, L., & Yin, P. (2017). Effects of 2,2’,4,4’-tetrabromodipheny ether (BDE-47) on gonadogenesis of the manila clam Ruditapes philippinarum. Aquatic Toxicology 193, 178–186. <https://doi.org/10.1016/j.aquatox.2017.10.022>
39. Liu, P., Miao, J., Song, Y., Pan, L., & Yin, P. (2017). Effects of 2,2’,4,4’-tetrabromodipheny ether (BDE-47) on gonadogenesis of the manila clam Ruditapes philippinarum. Aquatic Toxicology 193, 178–186. https://doi.org/10.1016/j.aquatox.2017.10.022
40. Lü, Z. M., Liu, W., Liu, L. Q., Wang, T. M., Shi, H. L., Ping, H. L., Chi, C. F., Yang, J. W., & Wu, C. W. (2016). Cloning, Characterization, and Expression Profile of Estrogen Receptor in Common Chinese Cuttlefish, Sepiella japonica. Journal of Experimental Zoology Part A: Ecological Genetics and Physiology 325, 181–193. https://doi.org/10.1002/jez.2011
41. Martínez-Pita, I., Sánchez-Lazo, C., & Herrera, M. (2016). A non-lethal method for establishing sexual maturation in mussels (Mytilus galloprovincialis (Lamarck, 1819)) during broodstock conditioning in hatcheries. Aquaculture International 24, 1247–1254. https://doi.org/10.1007/s10499-016-9981-4
42. Martínez-Pita, I., Sánchez-Lazo, C., Ruíz-Jarabo, I., Herrera, M., & Mancera, J. M. (2012). Biochemical composition, lipid classes, fatty acids and sexual hormones in the mussel Mytilus galloprovincialis from cultivated populations in south Spain. Aquaculture 358–359, 274–283. https://doi.org/10.1016/j.aquaculture.2012.06.003
43. Meng, X., Li, F., Wang, X., Liu, J., Ji, C., & Wu, H. (2019). Combinatorial immune and stress response, cytoskeleton and signal transduction effects of graphene and triphenyl phosphate (TPP) in the mussel *Mytilus galloprovincialis*. *Journal of Hazardous Materials*, 378, 120778. <https://doi.org/10.1016/j.jhazmat.2019.120778>
44. Mezghani-Chaari, S., Machreki-Ajimi, M., Hamza-Chaffai, A., & Minier, C. (2017). High estradiol exposure disrupts the reproductive cycle of the clam Ruditapes decussatus in a sex-specific way. Environmental Science and Pollution Research 24, 26670–26680. https://doi.org/10.1007/s11356-017-0146-4
45. Nikonova, L. L., Nekhoroshev, M. V., & Ryabushko, V. I. (2017). Total testosterone and estradiol in the gonads and gametes of the mussel Mytilus galloprovincialis Lam. Journal of Evolutionary Biochemistry and Physiology 53, 519–522. https://doi.org/10.1134/S0022093017060114
46. Nuurai, P., Wanichanon, C., & Wanichanon, R. (2020). Effect of gonadotropin releasing hormone on the expression of luteinizing hormone and estrogen in the nerve ganglia and ovary of a tropical abalone, Haliotis asinina Linnaeus. Acta Histochemica 122, 151454. https://doi.org/10.1016/j.acthis.2019.151454
47. Ocharoen, Y., Boonphakdee, C., Boonphakdee, T., Shinn, A. P., & Moonmangmee, S. (2018). High levels of the endocrine disruptors bisphenol-A and 17β-estradiol detected in populations of green mussel, Perna viridis, cultured in the Gulf of Thailand. Aquaculture, 497, 348–356. https://doi.org/10.1016/j.aquaculture.2018.07.057
48. Omran, N. E. S. E. S. (2012). Testosterone, gonadotropins and androgen receptor during spermatogenesis of Biomphalaria alexandrina snails (Pulmonata: Basommatophora). Reproductive Biology 12, 301–308. https://doi.org/10.1016/j.repbio.2012.10.002
49. Omran, N. E., & Salama, W. M. (2016). The endocrine disruptor effect of the herbicides atrazine and glyphosate on Biomphalaria alexandrina snails. Toxicology and Industrial Health, 32, 656–665. https://doi.org/10.1177/0748233713506959
50. Prisco, M., Agnese, M., De Marino, A., Andreuccetti, P., & Rosati, L. (2017). Spermatogenic Cycle and Steroidogenic Control of Spermatogenesis in Mytilus galloprovincialis Collected in the Bay of Naples. Anatomical Record 300, 1881–1894. https://doi.org/10.1002/ar.23626
51. Rizk, M. Z., Metwally, N. S., Hamed, M. A., & Mohamed, A. M. (2012). Correlation between steroid sex hormones, egg laying capacity and cercarial shedding in Biomphalaria alexandrina snails after treatment with Haplophyllum tuberculatum. Experimental Parasitology 132, 171–179. https://doi.org/10.1016/j.exppara.2012.06.011
52. Rosati, L., Agnese, M., Abagnale, L., Aniello, F., Andreuccetti, P., & Prisco, M. (2019b). The Mussel Mytilus galloprovincialis in the Bay of Naples: New Insights on Oogenic Cycle and Its Hormonal Control. Anatomical Record 302, 1039–1049. https://doi.org/10.1002/ar.24075
53. Rossato, M., Castro, I. B., Paganini, C. L., Colares, E. P., Fillmann, G., & Pinho, G. L. L. (2016). Sex steroid imbalances in the muricid Stramonita haemastoma from TBT contaminated sites. Environmental Science and Pollution Research 23, 7861–7868. <https://doi.org/10.1007/s11356-015-5942-0>
54. Sánchez, F. C., Díaz, M. E., & Aranda, D. A. (2020). Sex hormones in Strombus pugilis (Mollusca: Gastropoda) in different gonadal stages. Bulletin of Marine Science 96, 679–694. <https://doi.org/10.5343/bms.2019.0094>
55. Sheir, S. K., Mohamad, A. H., Osman, G. Y., & Elhafez, A. E. R. A. (2020). Acute and chronic effects of bisphenol a on hormonal disruption and histological alterations in the freshwater clam, Caelatura nilotica (Cailliaud, 1827). Egyptian Journal of Aquatic Biology and Fisheries 24, 397–406. https://doi.org/10.21608/EJABF.2020.120508
56. Smolarz, K., Zabrzańska, S., Konieczna, L., & Hallmann, A. (2018). Changes in steroid profiles of the blue mussel Mytilus trossulus as a function of season, stage of gametogenesis, sex, tissue and mussel bed depth. General and Comparative Endocrinology 259, 231–239. https://doi.org/10.1016/j.ygcen.2017.12.006
57. Song, Y., Miao, J., Cai, Y., & Pan, L. (2015). Molecular cloning, characterization, and expression analysis of a gonadotropin-releasing hormone-like cDNA in the clam, Ruditapes philippinarum. Comparative Biochemistry and Physiology Part B: Biochemistry and Molecular Biology 189, 47–54. https://doi.org/10.1016/j.cbpb.2015.07.005
58. Takishita, K., Takaki, Y., Chikaraishi, Y., Ikuta, T., Ozawa, G., Yoshida, T., Ohkouchi, N., & Fujikura, K. (2017). Genomic evidence thatmethanotrophic endosymbionts likely providedeep-sea bathymodiolus musselswith a sterol intermediate in cholesterol biosynthesis. Genome Biology and Evolution 9, 1148–1160. https://doi.org/10.1093/gbe/evx082
59. Thitiphuree, T., Nagasawa, K., & Osada, M. (2019). Molecular identification of steroidogenesis-related genes in scallops and their potential roles in gametogenesis. Journal of Steroid Biochemistry and Molecular Biology 186, 22–33. https://doi.org/10.1016/j.jsbmb.2018.09.004
60. Tian, S., Pan, L., & Sun, X. (2013). An investigation of endocrine disrupting effects and toxic mechanisms modulated by benzo[a]pyrene in female scallop Chlamys farreri. Aquatic Toxicology 144–145, 162–171. <https://doi.org/10.1016/j.aquatox.2013.09.031>
61. Tian, S., Pan, L., & Sun, X. (2013). An investigation of endocrine disrupting effects and toxic mechanisms modulated by benzo[a]pyrene in female scallop Chlamys farreri. Aquatic Toxicology 144–145, 162–171. https://doi.org/10.1016/j.aquatox.2013.09.031
62. Tian, S., Pan, L., Tao, Y., & Sun, X. (2015). Environmentally relevant concentrations of benzo[a]pyrene affect steroid levels and affect gonad of male scallop Chlamys farreri. Ecotoxicology and Environmental Safety 114, 150–156. https://doi.org/10.1016/j.ecoenv.2015.01.019
63. Tong, Y., Zhang, Y., Huang, J., Xiao, S., Zhang, Y., Li, J., Chen, J., & Yu, Z. (2015). Transcriptomics analysis of *Crassostrea hongkongensis* for the discovery of reproduction-related genes. *PLOS ONE*, 10(8), e0134280. <https://doi.org/10.1371/journal.pone.0134280>
64. Wang, S., Ji, C., Li, F., Zhan, J., Sun, T., Tang, J., & Wu, H. (2021). Tetrabromobisphenol A induced reproductive endocrine-disrupting effects in mussel Mytilus galloprovincialis. Journal of Hazardous Materials 416, 126228. https://doi.org/10.1016/j.jhazmat.2021.126228
65. Wang, T., Kong, H., Shang, Y., Dupont, S., Peng, J., Wang, X., Deng, Y., Hu, M., & Wang, Y. (2021). Ocean acidification but not hypoxia alters the gonad performance in the thick shell mussel Mytilus coruscus. Marine Pollution Bulletin 167, 112 282. https://doi.org/10.1016/j.marpolbul.2021.112282
66. Xu, R., Pan, L., Yang, Y., & Zhou, Y. (2020). Characterizing transcriptome in female scallop Chlamys farreri provides new insights into the molecular mechanisms of reproductive regulation during ovarian development and spawn. Gene 758, 144967. https://doi.org/10.1016/j.gene.2020.144967
67. Yang, Y., Pan, L., Zhou, Y., Xu, R., & Li, D. (2020). Benzo[a]pyrene exposure disrupts steroidogenesis and impairs spermatogenesis in diverse reproductive stages of male scallop (Chlamys farreri). Environmental Research 191, 110–125. https://doi.org/10.1016/j.envres.2020.110125
68. Yang, Y., Pan, L., Zhou, Y., Xu, R., & Li, D. (2020). Benzo[a]pyrene exposure disrupts steroidogenesis and impairs spermatogenesis in diverse reproductive stages of male scallop (Chlamys farreri). Environmental Research 191, 110–125. https://doi.org/10.1016/j.envres.2020.110125
69. Zabrzańska, S., Smolarz, K., Hallmann, A., Konieczna, L., Baczek, T., & Wołowicz, M. (2015). Sex-related differences in steroid concentrations in the blue mussel (Mytilus edulis trossulus) from the southern Baltic Sea. Comparative Biochemistry and Physiology Part A : Molecular and Integrative Physiology 183, 14–19. https://doi.org/10.1016/j.cbpa.2014.12.029
70. Zapata-Restrepo, L. M., Hauton, C., Williams, I. D., Jensen, A. C., & Hudson, M. D. (2019). Effects of the interaction between temperature and steroid hormones on gametogenesis and sex ratio in the European flat oyster (Ostrea edulis). Comparative Biochemistry and Physiology Part A : Molecular and Integrative Physiology 236, 110523. https://doi.org/10.1016/j.cbpa.2019.06.023
71. Zhang, M., Wei, H., Liu, T., Li, W., Li, Y., Wang, S., Xing, Q., Hu, X., Zhang, L., & Bao, Z. (2020). Potential GnRH and steroidogenesis pathways in the scallop Patinopecten yessoensis. Journal of Steroid Biochemistry and Molecular Biology 204, 105756. https://doi.org/10.1016/j.jsbmb.2020.105756
72. Zhang, M., Wei, H., Liu, T., Li, W., Li, Y., Wang, S., Xing, Q., Hu, X., Zhang, L., & Bao, Z. (2020). Potential GnRH and steroidogenesis pathways in the scallop Patinopecten yessoensis. Journal of Steroid Biochemistry and Molecular Biology 204, 105756. https://doi.org/10.1016/j.jsbmb.2020.105756
73. Zhang, Z., Bai, Q., Xu, X., & Zhang, X. (2021). Effects of the dominance hierarchy on social interactions, cortisol level, HPG-axis activities and reproductive success in the golden cuttlefish Sepia esculenta. Aquaculture 533, 736059. https://doi.org/10.1016/j.aquaculture.2020.736059
74. Zheng, B. H., An, L. H., Chang, H., Liu, Y., & Jiang, Z. Q. (2014). Evidence for the presence of sex steroid hormones in Zhikong scallop, Chlamys farreri. Journal of Steroid Biochemistry and Molecular Biology 143, 199–206. https://doi.org/10.1016/j.jsbmb.2014.03.002
75. Zhu, X., Guo, C., Lin, C., Wang, D., Wang, C., & Xu, S. (2018). Estradiol-17β and testosterone levels during the annual reproductive cycle of in Mytilus coruscus. Animal Reproduction Science 196, 35–42. <https://doi.org/10.1016/j.anireprosci.2018.06.015>

**3.10 Figure 13**

1. Abidli, S., Castro, L. F. C., Lahbib, Y., Reis-Henriques, M. A., Trigui El Menif, N., & Santos, M. M. (2013). Imposex development in Hexaplex trunculus (Gastropoda: Caenogastropoda) involves changes in the transcription levels of the retinoid X receptor (RXR). Chemosphere 93, 1161–1167. https://doi.org/10.1016/j.chemosphere.2013.06.054
2. André, A., Ruivo, R., Fonseca, E., Froufe, E., Castro, L. F. C., & Santos, M. M. (2019). The retinoic acid receptor (RAR) in molluscs: Function, evolution and endocrine disruption insights. Aquatic Toxicology 208, 80–89. https://doi.org/10.1016/j.aquatox.2019.01.002
3. Aquilino, M., Martínez-Guitarte, J. L., García, P., Beltrán, E. M., Fernández, C., & Sánchez-Argüello, P. (2018). Combining the assessment of apical endpoints and gene expression in the freshwater snail Physa acuta after exposure to reclaimed water. Science of the Total Environment 642, 80–189. https://doi.org/10.1016/j.scitotenv.2018.06.054
4. Bouétard, A., Besnard, A. L., Vassaux, D., Lagadic, L., & Coutellec, M. A. (2013). Impact of the redox-cycling herbicide diquat on transcript expression and antioxidant enzymatic activities of the freshwater snail Lymnaea stagnalis. Aquatic Toxicology 126, 256–265. https://doi.org/10.1016/j.aquatox.2012.11.013
5. Carpenter, S., Rothwell, C. M., Wright, M. L., de Hoog, E., Walker, S., Hudson, E., & Spencer, G. E. (2016). Extending the duration of long-term memories: Interactions between environmental darkness and retinoid signaling. Neurobiology of Learning and Memory 136, 34–46. https://doi.org/10.1016/j.nlm.2016.09.008
6. Carter, C. J., Rand, C., Mohammad, I., Lepp, A., Vesprini, N., Wiebe, O., Carlone, R., & Spencer, G. E. (2015). Expression of a retinoic acid receptor (RAR)-like protein in the embryonic and adult nervous system of a protostome species. Journal of Experimental Zoology Part B: Molecular and Developmental Evolution 324, 51–67. https://doi.org/10.1002/jez.b.22604
7. Coelho, I., Lima, D., André, A., Melo, C., Ruivo, R., Reis-Henriques, M. A., Santos, M. M. H., & Castro, L. F. C. (2012). Molecular characterization of Adh3 from the mollusc Nucella lapillus: Tissue gene expression after tributyltin and retinol exposure. Journal of Molluscan Studies 78, 343–348. https://doi.org/10.1093/mollus/eys018
8. Gesto, M., Castro, L. F. C., & Santos, M. M. (2013). Differences in retinoid levels and metabolism among gastropod lineages: Imposex-susceptible gastropods lack the ability to store retinoids in the form of retinyl esters. Aquatic Toxicology 142–143, 96–103. https://doi.org/10.1016/j.aquatox.2013.08.001
9. Gesto, M., Castro, L. F. C., Reis-Henriques, M. A., & Santos, M. M. (2012). Retinol metabolism in the mollusk osilinus lineatus indicates an ancient origin for retinyl ester storage capacity. PLoS ONE 7, e35138 https://doi.org/10.1371/journal.pone.0035138
10. Gesto, M., Ruivo, R., Páscoa, I., André, A., Castro, L. F. C., & Santos, M. M. (2016). Retinoid level dynamics during gonad recycling in the limpet Patella vulgata. General and Comparative Endocrinology, 225, 142–148. https://doi.org/10.1016/j.ygcen.2015.10.017
11. Giraud-Billoud, M., & Castro-Vazquez, A. (2019). Aging and retinoid X receptor agonists on masculinization of female Pomacea canaliculata, with a critical appraisal of imposex evaluation in the Ampullariidae. Ecotoxicology and Environmental Safety 169, 573–582. https://doi.org/10.1016/j.ecoenv.2018.10.096
12. Giulianelli, S., Primost, M. A., Lanari, C., & Bigatti, G. (2020). RXR Expression in Marine Gastropods with Different Sensitivity to Imposex Development. Scientific Reports 10, 9507. https://doi.org/10.1038/s41598-020-66402-1
13. Gutierrez-Mazariegos, J., Nadendla, E. K., Lima, D., Pierzchalski, K., Jones, J. W., Kane, M., Nishikawa, J. I., Hiromori, Y., Nakanishi, T., Santos, M. M., Castro, L. F. C., Bourguet, W., Schubert, M., & Laudet, V. (2014). A mollusk retinoic acid receptor (RAR) ortholog sheds light on the evolution of ligand binding. Endocrinology 155, 4275–4286. https://doi.org/10.1210/en.2014-1181
14. Huang, W., Wu, Q., Xu, F., Li, L., Li, J., Que, H., & Zhang, G. (2020). Functional characterization of retinoid X receptor with an emphasis on the mediation of organotin poisoning in the Pacific oyster (Crassostrea gigas). Gene 753, 144780. https://doi.org/10.1016/j.gene.2020.144780
15. Ip, J. C. H., Leung, P. T. Y., Ho, K. K. Y., Qiu, J. W., & Leung, K. M. Y. (2016). De novo transcriptome assembly of the marine gastropod Reishia clavigera for supporting toxic mechanism studies. Aquatic Toxicology 178, 39–48. https://doi.org/10.1016/j.aquatox.2016.07.006
16. Jin, K., Jin, Q., Cai, Z., Huang, B., Wei, L., Zhang, M., Guo, W., Liu, Y., & Wang, X. (2021). Molecular Characterization of Retinoic Acid Receptor CgRAR in Pacific Oyster (Crassostrea gigas). Frontiers in Physiology 12, 1–6. https://doi.org/10.3389/fphys.2021.666842
17. Johnson, A., de Hoog, E., Tolentino, M., Nasser, T., & Spencer, G. E. (2019). Pharmacological evidence for the role of RAR in axon guidance and embryonic development of a protostome species. Genesis 57, 1–14. https://doi.org/10.1002/dvg.23301
18. Lesoway, M. P., & Henry, J. Q. (2021). Retinoids promote penis development in sequentially hermaphroditic snails. Developmental Biology 478, 122–132. https://doi.org/10.1016/j.ydbio.2021.06.013
19. Lv, J., Feng, L., Bao, Z., Guo, H., Zhang, Y., Jiao, W., Zhang, L., Wang, S., He, Y., & Hu, X. (2013). Molecular Characterization of RXR (Retinoid X Receptor) Gene Isoforms from the Bivalve Species Chlamys farreri. PLoS ONE 8, 1–9. https://doi.org/10.1371/journal.pone.0074290
20. Martínez-Paz, P., Morales, M., Sánchez-Argüello, P., Morcillo, G., & Martínez-Guitarte, J. L. (2017). Cadmium in vivo exposure alters stress response and endocrine-related genes in the freshwater snail Physa acuta. New biomarker genes in a new model organism. Environmental Pollution 220, 1488–1497. https://doi.org/10.1016/j.envpol.2016.10.012
21. Raingeard, D., Bilbao, E., Cancio, I., & Cajaraville, M. P. (2013). Retinoid X receptor (RXR), estrogen receptor (ER) and other nuclear receptors in tissues of the mussel Mytilus galloprovincialis: Cloning and transcription pattern. Comparative Biochemistry and Physiology Part A: Molecular and Integrative Physiology 165, 178–190. https://doi.org/10.1016/j.cbpa.2013.03.001
22. Rojas-garcía, A. E., Robledo-marenco, M. L., & Barrón-vivanco, B. S. (2014). Exposure to tributyltin chloride induces penis and vas deferens development and increases RXR expression in females of the purple snail ( Plicopurpura pansa ) Abstract Tributyltin ( TBT ) and its derivatives are widely used as antifouling paints for ships. ISJ – Invertebrate Survival journal 11, 204–212.
23. Rothwell, C. M., Simmons, J., Peters, G., & Spencer, G. E. (2014). Novel interactive effects of darkness and retinoid signaling in the ability to form long-term memory following aversive operant conditioning. Neurobiology of Learning and Memory 114, 251–263. https://doi.org/10.1016/j.nlm.2014.07.007
24. Tan, K., Guo, Z., Zhang, H., Ma, H., Li, S., & Zheng, H. (2021). Carotenoids regulation in polymorphic noble scallops Chlamys nobilis under different light cycle. Aquaculture 531, 735937. https://doi.org/10.1016/j.aquaculture.2020.735937
25. Urushitani, H., Katsu, Y., Kagechika, H., Sousa, A. C. A., Barroso, C. M., Ohta, Y., Shiraishi, H., Iguchi, T., & Horiguchi, T. (2018). Characterization and comparison of transcriptional activities of the retinoid X receptors by various organotin compounds in three prosobranch gastropods; Thais clavigera, Nucella lapillus and Babylonia japonica. Aquatic Toxicology 199, 103–115. https://doi.org/10.1016/j.aquatox.2018.03.029
26. Urushitani, H., Katsu, Y., Ohta, Y., Shiraishi, H., Iguchi, T., & Horiguchi, T. (2013). Cloning and characterization of the retinoic acid receptor-like protein in the rock shell, Thais clavigera. Aquatic Toxicology 142–143, 403–413. https://doi.org/10.1016/j.aquatox.2013.09.008
27. Vogeler, S., Bean, T. P., Lyons, B. P., & Galloway, T. S. (2016). Dynamics of nuclear receptor gene expression during Pacific oyster development. BMC Developmental Biology 16, 33. <https://doi.org/10.1186/s12861-016-0129-6>
28. Vöcking, O., Leclère, L., & Hausen, H. (2021). The rhodopsin–retinochrome system for retinal re-isomerization predates the origin of cephalopod eyes. *BMC Ecology and Evolution*, 21, 215. https://doi.org/10.1186/s12862-021-01939-x

**3.11 Figure 15**

1. Agnese, M., Rosati, L., Prisco, M., Borzacchiello, L., Abagnale, L., & Andreuccetti, P. (2019). The expression of estrogen receptors during the Mytilus galloprovincialis ovarian cycle. Journal of Experimental Zoology Part A: Ecological and Integrative Physiology 331, 367–373. https://doi.org/10.1002/jez.2272
2. Balbi, T., Franzellitti, S., Fabbri, R., Montagna, M., Fabbri, E., & Canesi, L. (2016). Impact of bisphenol A (BPA) on early embryo development in the marine mussel Mytilus galloprovincialis: Effects on gene transcription. Environmental Pollution 218, 996–1004. https://doi.org/10.1016/j.envpol.2016.08.050
3. Deng, X., Pan, L., Cai, Y., & Jin, Q. (2016). Transcriptomic changes in the ovaries of scallop Chlamys farreri exposed to benzo[a]pyrene. Genes and Genomics 38, 509–518. https://doi.org/10.1007/s13258-016-0397-3
4. Jianbin Ni, Zhen Zeng, C. K. (2013). Sex steroid levels and expression patterns of estrogen receptor gene in the oyster Crassostrea angulata during reproductive cycle, Aquaculture 376-379, 105–116. https://doi.org/10.1016/j.aquaculture.2012.11.023
5. Nagasawa, K., Treen, N., Kondo, R., Otoki, Y., Itoh, N., Rotchell, J. M., & Osada, M. (2015). Molecular characterization of an estrogen receptor and estrogen-related receptor and their autoregulatory capabilities in two Mytilus species. Gene 564, 153–159. https://doi.org/10.1016/j.gene.2015.03.073
6. Raingeard, D., Bilbao, E., Cancio, I., & Cajaraville, M. P. (2013). Retinoid X receptor (RXR), estrogen receptor (ER) and other nuclear receptors in tissues of the mussel Mytilus galloprovincialis: Cloning and transcription pattern. Comparative Biochemistry and Physiology Part A: Molecular and Integrative Physiology 165, 178–190. https://doi.org/10.1016/j.cbpa.2013.03.001
7. Rosati, L., Agnese, M., Verderame, M., Aniello, F., Venditti, M., Mita, D. G., Andreuccetti, P., & Prisco, M. (2019a). Morphological and molecular responses in ovaries of Mytilus galloprovincialis collected in two different sites of the Naples Bay. Journal of Experimental Zoology Part A: Ecological and Integrative Physiology 331, 52–60. https://doi.org/10.1002/jez.2231
8. Tian, S., Pan, L., & Sun, X. (2013). An investigation of endocrine disrupting effects and toxic mechanisms modulated by benzo[a]pyrene in female scallop Chlamys farreri. Aquatic Toxicology 144–145, 162–171. https://doi.org/10.1016/j.aquatox.2013.09.031
9. Tong, Y., Zhang, Y., Huang, J., Xiao, S., Zhang, Y., Li, J., Chen, J., & Yu, Z. (2015). Transcriptomics analysis of crassostrea hongkongensis for the discovery of reproduction-related genes. PLoS ONE 10, 1–24. https://doi.org/10.1371/journal.pone.0134280
10. Tran, T. K. A., MacFarlane, G. R., Kong, R. Y. C., O’Connor, W. A., & Yu, R. M. K. (2016). Potential mechanisms underlying estrogen-induced expression of the molluscan estrogen receptor (ER) gene. Aquatic Toxicology 179, 82–94. https://doi.org/10.1016/j.aquatox.2016.08.015
11. Vogeler, S., Bean, T. P., Lyons, B. P., & Galloway, T. S. (2016). Dynamics of nuclear receptor gene expression during Pacific oyster development. BMC Developmental Biology 16, 33. https://doi.org/10.1186/s12861-016-0129-6
12. Zeng, M., Chen, D., Li, Q., Chen, H., & Huang, Q. (2020). Estrogen receptor regulates immune defense by suppressing NF-κB signaling in the Crassostrea hongkongensis. Fish and Shellfish Immunology 106, 796–803. https://doi.org/10.1016/j.fsi.2020.08.038
13. Zhang, H., Pan, L., & Zhang, L. (2012). Molecular cloning and characterization of estrogen receptor gene in the Scallop Chlamys farreri: Expression profiles in response to endocrine disrupting chemicals. Comparative Biochemistry and Physiology Part C: Toxicology and Pharmacology 156, 51–57. https://doi.org/10.1016/j.cbpc.2012.03.007
14. Zheng, B. H., An, L. H., Chang, H., Liu, Y., & Jiang, Z. Q. (2014). Evidence for the presence of sex steroid hormones in Zhikong scallop, Chlamys farreri. Journal of Steroid Biochemistry and Molecular Biology 143, 199–206. https://doi.org/10.1016/j.jsbmb.2014.03.002

# 4. References included in data inventories

**4.1. Mollusca AND Hormones**

1. Abd El-Atti, M. S., El-Sayed, A. S. A., & Said, R. M. (2020). Usage of pharmaceutical contraceptive drug for controlling Eobania vermiculata snails by baits technique. *Heliyon* **6**, e05630. <https://doi.org/10.1016/j.heliyon.2020.e05630>
2. Abdel-Hamid, H., & Mekawey, A. A. I. (2014). Biological and hematological responses of Biomphalaria alexandrina to mycobiosynthsis silver nanoparticles. *Journal of the Egyptian Society of Parasitology* **44**, 627–637. <https://doi.org/10.12816/0007866>
3. Abidli, S., Santos, M. M. H., Lahbib, Y., Castro, L. F. C., Reis-Henriques, M. A., & Trigui El Menif, N. (2012). Tributyltin (TBT) effects on Hexaplex trunculus and Bolinus brandaris (Gastropoda: Muricidae): Imposex induction and sex hormone levels insights. *Ecological Indicators* **13**, 13–21. <https://doi.org/10.1016/j.ecolind.2011.05.001>
4. Abu El Einin, H. M., Ali, R. E., Gad El-Karim, R. M., Youssef, A. A., Abdel-Hamid, H., & Habib, M. R. (2019). Biomphalaria alexandrina: A model organism for assessing the endocrine disrupting effect of 17β-estradiol. *Environmental Science and Pollution Research* **26**, 23328–23336. <https://doi.org/10.1007/s11356-019-05586-0>
5. Afsar, N., Siddiqui, G., Rasheed, M., Ahmed, V. U., & Khan, A. (2012). GC-MS analysis of fatty acids (FAs) of prosobranch gastropod species thais carinifera from pakistan coast (North Arabian Sea). *Journal of the Chemical Society of Pakistan* **34**, 565–569.
6. Akcha, F., Barranger, A., Bachère, E., Berthelin, C. H., Piquemal, D., Alonso, P., Sallan, R. R., Dimastrogiovanni, G., Porte, C., Menard, D., Szczybelski, A., Benabdelmouna, A., Auffret, M., Rouxel, J., & Burgeot, T. (2016). Effects of an environmentally relevant concentration of diuron on oyster genitors during gametogenesis: Responses of early molecular and cellular markers and physiological impacts. *Environmental Science and Pollution Research* **23**, 8008–8020. <https://doi.org/10.1007/s11356-015-5969-2>
7. Avila-Poveda, O. H., Montes-Pérez, R. C., Benitez-Villalobos, F., & Rosas, C. (2013). Development and validation of a solid-phase radioimmunoassay for measuring progesterone and testosterone in octopus gonad extracts. *Malacologia* **56**, 121–134. <https://doi.org/10.4002/040.056.0209>
8. Avila-Poveda, O. H., Montes-Pérez, R. C., Koueta, N., Benítez-Villalobos, F., Ramírez-Pérez, J. S., Jimenez-Gutierrez, L. R., & Rosas, C. (2015). Seasonal changes of progesterone and testosterone concentrations throughout gonad maturation stages of the Mexican octopus, Octopus maya (Octopodidae: Octopus). *Molluscan Research* **35**, 161–172. <https://doi.org/10.1080/13235818.2015.1045055>
9. Binder, A. R. D., Pfaffl, M. W., Hiltwein, F., JuergenGeis, J., & Beggel, S. (2019). Does environmental stress affect cortisol biodistribution in freshwatermussels? *Conservation Physiology* **7**, 1–10. <https://doi.org/10.1093/conphys/coz101>
10. Bogdanov, A., Hertzer, C., Kehraus, S., Nietzer, S., Rohde, S., Schupp, P. J., Wägele, H., & Gabriele M. König. (2017). Secondary metabolome and its defensive role in the aeolidoidean Phyllodesmium longicirrum, (Gastropoda, Heterobranchia, Nudibranchia). *Beilstein Journal of Organic Chemistry* **13**, 502–519. <https://doi.org/10.3762/bjoc.13.50>
11. Cappello, T., Fernandes, D., Maisano, M., Casano, A., Bonastre, M., Bebianno, M. J., Mauceri, A., Fasulo, S., & Porte, C. (2017). Sex steroids and metabolic responses in mussels Mytilus galloprovincialis exposed to drospirenone. *Ecotoxicology and Environmental Safety* **143**, 166–172. <https://doi.org/10.1016/j.ecoenv.2017.05.031>
12. Cheour, M. K., Elgharsalli, R., Benmessaoud, R., & Aloui-Bejaoui, N. (2014). Variation of steroid concentrations during the reproductive cycle of the snail Osilinus articulatus in the Kerkennah Islands (Gulf of Gabes, Tunisia). *Cahiers de Biologie Marine* **55**, 191-199 NP – 9.
13. Chong Sánchez, F., Enríquez Díaz, M. & Aldana Aranda, D. (2020). Sex hormones in *Strombus pugilis* (Mollusca: Gastropoda) in different gonadal stages. *Bulletin of Marine Science* 96, 679–694.
14. Chong Sánchez, F., Enriquez Díaz, M., Murillo Rodríguez, E., & Aldana Aranda, D. (2019). First use of a non-invasive technique for determination of sex hormones in the queen conch Lobatus gigas, Mollusca Gastropoda. *Aquaculture International* **27**, 437–448. <https://doi.org/10.1007/s10499-018-0336-1>
15. Cubero-Leon, E., Puinean, A. M., Labadie, P., Ciocan, C., Itoh, N., Kishida, M., Osada, M., Minier, C., Hill, E. M., & Rotchell, J. M. (2012). Two CYP3A-like genes in the marine mussel Mytilus edulis: mRNA expression modulation following short-term exposure to endocrine disruptors. Marine Environmental Research, 74, 32–39. https://doi.org/10.1016/j.marenvres.2011.11.012
16. Dagorn, F., Couzinet-Mossion, A., Kendel, M., Beninger, P. G., Rabesaotra, V., Barnathan, G., & Wielgosz-Collin, G. (2016). Exploitable lipids and fatty acids in the invasive oyster Crassostrea gigas on the French Atlantic coast. *Marine Drugs* **14**, 1–12. <https://doi.org/10.3390/md14060104>
17. Dimastrogiovanni, G., Fernandes, D., Bonastre, M., & Porte, C. (2015). Progesterone is actively metabolized to 5α-pregnane-3,20-dione and 3β-hydroxy-5α-pregnan-20-one by the marine mussel Mytilus galloprovincialis. *Aquatic Toxicology* **165**, 93–100. https://doi.org/10.1016/j.aquatox.2015.05.018
18. Dokmak, H. A. A. S., El-Emam, M. A., Mossalem, H. S., El-Tayeb, T. A., & Khalil, M. T. (2021). Impact of carbamide perhydrate on the snail bulinus truncatus, the intermediate host of schistosoma haematobium. *Egyptian Journal of Aquatic Biology and Fisheries* **25**, 85–99. <https://doi.org/10.21608/ejabf.2021.172770>
19. Fiorini, R., Ventrella, V., Trombetti, F., Fabbri, M., Pagliarani, A., & Nesci, S. (2019). Lipid-protein interactions in mitochondrial membranes from bivalve mollusks: Molecular strategies in different species. *Comparative Biochemistry and Physiology Part B: Biochemistry & Molecular Biology* **227**, 12–20. <https://doi.org/10.1016/j.cbpb.2018.08.010>
20. Gesto, M., Castro, L. F. C., & Santos, M. M. (2013). Differences in retinoid levels and metabolism among gastropod lineages: Imposex-susceptible gastropods lack the ability to store retinoids in the form of retinyl esters. *Aquatic Toxicology* **142–143**, 96–103. <https://doi.org/10.1016/j.aquatox.2013.08.001>
21. Gesto, M., Castro, L. F. C., Reis-Henriques, M. A., & Santos, M. M. (2012). Retinol metabolism in the mollusk osilinus lineatus indicates an ancient origin for retinyl ester storage capacity. *PLoS ONE* **7**, e35138 <https://doi.org/10.1371/journal.pone.0035138>
22. Gesto, M., Ruivo, R., Páscoa, I., André, A., Castro, L. F. C., & Santos, M. M. (2016). Retinoid level dynamics during gonad recycling in the limpet Patella vulgata. General and Comparative Endocrinology, 225, 142–148. <https://doi.org/10.1016/j.ygcen.2015.10.017>
23. Giner, J. L., Zhao, H., Dixon, M. S., & Wikfors, G. H. (2016). Bioconversion of 13C-labeled microalgal phytosterols to cholesterol by the Northern Bay scallop, Argopecten irradians irradians. *Comparative Biochemistry and Physiology Part B: Biochemistry & Molecular Biology* **192**, 1–8. <https://doi.org/10.1016/j.cbpb.2015.11.003>
24. Giusti, A., Ducrot, V., Joaquim-Justo, C., & Lagadic, L. (2013). Testosterone levels and fecundity in the hermaphroditic aquatic snail Lymnaea stagnalis exposed to testosterone and endocrine disruptors. *Environmental Toxicology and Chemistry* **32**, 1740–1745. <https://doi.org/10.1002/etc.2234>
25. Goto, Y., Kajiwara, M., Yanagisawa, Y., Hirose, H., Yoshimi, T., Umemura, M., Nakano, H., Takahashi, S., Shida, Y., Iguchi, T., Takahashi, Y., & Miura, T. (2012). Detection of vertebrate-type steroid hormones and their converting activities in the neogastropod Thais clavigera (Küster, 1858). *Journal of Molluscan Studies* **78**, 197–204. <https://doi.org/10.1093/mollus/eys001>
26. Gust, M., Gagné, F., Berlioz-Barbier, A., Besse, J. P., Buronfosse, T., Tournier, M., Tutundjian, R., Garric, J., & Cren-Olivé, C. (2014). Caged mudsnail Potamopyrgus antipodarum (Gray) as an integrated field biomonitoring tool: Exposure assessment and reprotoxic effects of water column contamination. *Water Research* **54**, 222–236. <https://doi.org/10.1016/j.watres.2014.01.057>
27. Gutierrez-Mazariegos, J., Nadendla, E. K., Lima, D., Pierzchalski, K., Jones, J. W., Kane, M., Nishikawa, J. I., Hiromori, Y., Nakanishi, T., Santos, M. M., Castro, L. F. C., Bourguet, W., Schubert, M., & Laudet, V. (2014). A mollusk retinoic acid receptor (RAR) ortholog sheds light on the evolution of ligand binding. *Endocrinology* **155**, 4275–4286. <https://doi.org/10.1210/en.2014-1181>
28. Habib, M. R., Ghoname, S. I., Ali, R. E., El-Karim, R. M. G., Youssef, A. A., Croll, R. P., & Miller, M. W. (2020). Biochemical and apoptotic changes in the nervous and ovotestis tissues of Biomphalaria alexandrina following infection with Schistosoma mansoni. *Experimental Parasitology* **213**, 107887. <https://doi.org/10.1016/j.exppara.2020.107887>
29. Halem, Z. M., Ross, D. J., & Cox, R. L. (2014). Evidence for intraspecific endocrine disruption of Geukensia demissa (Atlantic ribbed mussel) in an urban watershed. *Comparative Biochemistry and Physiology Part A : Molecular and Integrative Physiology* **175**, 1–6. <https://doi.org/10.1016/j.cbpa.2014.04.016>
30. Hallmann, A., Smolarz, K., Konieczna, L., Zabrzańska, S., Belka, M., & Baczek, T. (2016). LC-MS measurment of free steroids in mussels (Mytilus trossulus) from the southern Baltic Sea. *Journal of Pharmaceutical and Biomedical Analysis* **117**, 311–315. <https://doi.org/10.1016/j.jpba.2015.09.013>
31. Hallmann, A., Konieczna, L., Swiezak, J., Milczarek, R., & Smolarz, K. (2019). Aromatisation of steroids in the bivalve *Mytilus trossulus*. *PeerJ*, 7, e6953. <https://doi.org/10.7717/peerj.6953>
32. Huang, W., Xu, F., Li, L., Que, H., & Zhang, G. (2019). The transcription of iodothyronine deiodinase genes is regulated by thyroid hormone receptor in the Pacific oyster Crassostrea gigas. *Journal of Oceanology and Limnology* **37**, 1317–1323. <https://doi.org/10.1007/s00343-019-8207-9>
33. Huang, W., Xu, F., Qu, T., Zhang, R., Li, L., Que, H., & Zhang, G. (2015). Identification of thyroid hormones and functional characterization of thyroid hormone receptor in the pacific oyster Crassostrea gigas provide insight into evolution of the thyroid hormone system. *PLoS ONE* **10**, 1–20. <https://doi.org/10.1371/journal.pone.0144991>
34. Hurtado, M. A., Racotta, I. S., Arcos, F., Morales-Bojórquez, E., Moal, J., Soudant, P., & Palacios, E. (2012). Seasonal variations of biochemical, pigment, fatty acid, and sterol compositions in female Crassostrea corteziensis oysters in relation to the reproductive cycle. *Comparative Biochemistry and Physiology Part B: Biochemistry and Molecular Biology* **163**, 172–183. <https://doi.org/10.1016/j.cbpb.2012.05.011>
35. Ibrahim, A. M., & Ghoname, S. I. (2018). Molluscicidal impacts of Anagallis arvensis aqueous extract on biological, hormonal, histological and molecular aspects of Biomphalaria alexandrina snails. *Experimental Parasitology* **192**, 36–41. <https://doi.org/10.1016/j.exppara.2018.07.014>
36. Ibrahim, A. M., & Sayed, D. A. (2019). Toxicological impact of oxyfluorfen 24% herbicide on the reproductive system, antioxidant enzymes, and endocrine disruption of Biomphalaria alexandrina (Ehrenberg, 1831) snails. *Environmental Science and Pollution Research* **26**, 7960–7968. <https://doi.org/10.1007/s11356-019-04251-w>
37. Jiang, S., Miao, J., Wang, X., Liu, P., & Pan, L. (2019). Inhibition of growth in juvenile manila clam Ruditapes philippinarum: Potential adverse outcome pathway of TBBPA. *Chemosphere* **224**, 588–596. <https://doi.org/10.1016/j.chemosphere.2019.02.157>
38. Kandil, M. A., Eweis, E. A., Mobarak Soha, A., & Nada Abbas, M. T. (2020). Effects of chitosan and emamectin benzoate on the reproductive system of Eobania vermiculata (Muller) land snails. *Egyptian Journal of Biological Pest Control* ***30***, 1–8. <https://doi.org/10.1186/s41938-020-00224-1>
39. Kawashima, H., Ohnishi, M., & Ogawa, S. (2013). Distribution of unusual cholesterol precursors, 4-Methyl- and 4, 4-dimethylsterols with Δ8 unsaturation, in gonads of marine archaeogastropods. *Journal of Oleo Science* **62**, 465–470. <https://doi.org/10.5650/jos.62.465>
40. Knigge, T., Dahboul, F., Alain, D., & Monsinjon, T. (2015). The gametogenic cycle and oestradiol levels in the zebra mussel Dreissena polymorpha: A 1-year study. *Journal of Molluscan Studies* **81**, 58–65. <https://doi.org/10.1093/mollus/eyu056>
41. Lazzara, R., Blázquez, M., Porte, C., & Barata, C. (2012). Low environmental levels of fluoxetine induce spawning and changes in endogenous estradiol levels in the zebra mussel Dreissena polymorpha. *Aquatic Toxicology* **106–107**, 123–130. <https://doi.org/10.1016/j.aquatox.2011.11.003>
42. Lecomte, V., Noury, P., Tutundjian, R., Buronfosse, T., Garric, J., & Gust, M. (2013). Organic solvents impair life-traits and biomarkers in the New Zealand mudsnail Potamopyrgus antipodarum (Gray) at concentrations below OECD recommendations. *Aquatic Toxicology* **140–141**, 196–203. <https://doi.org/10.1016/j.aquatox.2013.06.006>
43. Liu, J., Zhang, Z., Zhang, L., Liu, X., Yang, D., & Ma, X. (2014a). Variations of estradiol-17β and testosterone levels correlated with gametogenesis in the gonad of Zhikong scallop (Chlamys farreri) during annual reproductive cycle. *Canadian Journal of Zoology* **92**, 195–204. <https://doi.org/10.1139/cjz-2013-0202>
44. Liu, P., Miao, J., Song, Y., Pan, L., & Yin, P. (2017). Effects of 2,2’,4,4’-tetrabromodipheny ether (BDE-47) on gonadogenesis of the manila clam Ruditapes philippinarum. *Aquatic Toxicology* **193**, 178–186. <https://doi.org/10.1016/j.aquatox.2017.10.022>
45. Lü, Z. M., Liu, W., Liu, L. Q., Wang, T. M., Shi, H. L., Ping, H. L., Chi, C. F., Yang, J. W., & Wu, C. W. (2016). Cloning, Characterization, and Expression Profile of Estrogen Receptor in Common Chinese Cuttlefish, Sepiella japonica. Journal of Experimental Zoology Part A: *Ecological Genetics and Physiology* **325**, 181–193. <https://doi.org/10.1002/jez.2011>
46. Lustrino, D., Silva, A. C. M., Araujo, I. G., Tunholi, V. M., Tunholi-Alves, V. M., Castro, R. N., Carvalho, D. P., Pinheiro, J., & Marassi, M. P. (2017). Evidence of the presence of thyroid hormones in achatina fulica snails. *Anais Da Academia Brasileira de Ciencias*, **89**, 2181–2188. <https://doi.org/10.1590/0001-3765201720160698>
47. Martínez-Pita, I., Sánchez-Lazo, C., & Herrera, M. (2016). A non-lethal method for establishing sexual maturation in mussels (Mytilus galloprovincialis (Lamarck, 1819)) during broodstock conditioning in hatcheries. *Aquaculture International* **24**, 1247–1254. <https://doi.org/10.1007/s10499-016-9981-4>
48. Martínez-Pita, I., Sánchez-Lazo, C., Ruíz-Jarabo, I., Herrera, M., & Mancera, J. M. (2012). Biochemical composition, lipid classes, fatty acids and sexual hormones in the mussel Mytilus galloprovincialis from cultivated populations in south Spain. *Aquaculture* **358–359**, 274–283. <https://doi.org/10.1016/j.aquaculture.2012.06.003>
49. Mezghani-Chaari, S., Machreki-Ajimi, M., Hamza-Chaffai, A., & Minier, C. (2017). High estradiol exposure disrupts the reproductive cycle of the clam Ruditapes decussatus in a sex-specific way. *Environmental Science and Pollution Research* **24**, 26670–26680. <https://doi.org/10.1007/s11356-017-0146-4>
50. Ni, J., Zeng, Z., & Ke, C. (2013). Sex steroid levels and expression patterns of estrogen receptor gene in the oyster *Crassostrea angulata* during the reproductive cycle. *Aquaculture*, 376–379, 105–116. <https://doi.org/10.1016/j.aquaculture.2012.11.023>
51. Nikonova, L. L., Nekhoroshev, M. V., & Ryabushko, V. I. (2017). Total testosterone and estradiol in the gonads and gametes of the mussel Mytilus galloprovincialis Lam. *Journal of Evolutionary Biochemistry and Physiology* **53**, 519–522. https://doi.org/10.1134/S0022093017060114
52. Nuurai, P., Wanichanon, C., & Wanichanon, R. (2020). Effect of gonadotropin releasing hormone on the expression of luteinizing hormone and estrogen in the nerve ganglia and ovary of a tropical abalone, Haliotis asinina Linnaeus. *Acta Histochemica* **122**, 151454. <https://doi.org/10.1016/j.acthis.2019.151454>
53. Ocharoen, Y., Boonphakdee, C., Boonphakdee, T., Shinn, A. P., & Moonmangmee, S. (2018). High levels of the endocrine disruptors bisphenol-A and 17β-estradiol detected in populations of green mussel, Perna viridis, cultured in the Gulf of Thailand. *Aquaculture*, **497**, 348–356. <https://doi.org/10.1016/j.aquaculture.2018.07.057>
54. Omran, N. E. S. E. S. (2012). Testosterone, gonadotropins and androgen receptor during spermatogenesis of Biomphalaria alexandrina snails (Pulmonata: Basommatophora). *Reproductive Biology* **12**, 301–308. <https://doi.org/10.1016/j.repbio.2012.10.002>
55. Omran, N. E., & Salama, W. M. (2016). The endocrine disruptor effect of the herbicides atrazine and glyphosate on Biomphalaria alexandrina snails. *Toxicology and Industrial Health*, 32, 656–665. <https://doi.org/10.1177/0748233713506959>
56. Pryce, K., Samuel, D., Lagares, E., Myrthil, M., Bess, F., Harris, A., Welsh, C., Carroll, M. A., & Catapane, E. J. (2015). Presence of Octopamine and an Octopamine Receptor in Crassostrea virginica. *In Vivo* **37**, 16–24.
57. Rizk, M. Z., Metwally, N. S., Hamed, M. A., & Mohamed, A. M. (2012). Correlation between steroid sex hormones, egg laying capacity and cercarial shedding in Biomphalaria alexandrina snails after treatment with Haplophyllum tuberculatum. *Experimental Parasitology* **132**, 171–179. <https://doi.org/10.1016/j.exppara.2012.06.011>
58. Rossato, M., Castro, I. B., Paganini, C. L., Colares, E. P., Fillmann, G., & Pinho, G. L. L. (2016). Sex steroid imbalances in the muricid Stramonita haemastoma from TBT contaminated sites. *Environmental Science and Pollution Research* **23**, 7861–7868. <https://doi.org/10.1007/s11356-015-5942-0>
59. Sheir, S. K., Mohamad, A. H., Osman, G. Y., & Elhafez, A. E. R. A. (2020). Acute and chronic effects of bisphenol a on hormonal disruption and histological alterations in the freshwater clam, Caelatura nilotica (Cailliaud, 1827). *Egyptian Journal of Aquatic Biology and Fisheries* **24**, 397–406. <https://doi.org/10.21608/EJABF.2020.120508>
60. Smolarz, K., Zabrzańska, S., Konieczna, L., & Hallmann, A. (2018). Changes in steroid profiles of the blue mussel Mytilus trossulus as a function of season, stage of gametogenesis, sex, tissue and mussel bed depth. *General and Comparative Endocrinology* **259**, 231–239. <https://doi.org/10.1016/j.ygcen.2017.12.006>
61. Song, Y., Miao, J., Cai, Y., & Pan, L. (2015). Molecular cloning, characterization, and expression analysis of a gonadotropin-releasing hormone-like cDNA in the clam, Ruditapes philippinarum. *Comparative Biochemistry and Physiology Part B: Biochemistry and Molecular Biology* **189**, 47–54. <https://doi.org/10.1016/j.cbpb.2015.07.005>
62. Song, Y., Miao, J., Pan, L., & Wang, X. (2016). Exposure to2,2’,4,4’-tetrabromodiphenyl ether (BDE-47) alters thyroid hormone levels and thyroid hormone-regulated gene transcription in manila clam Ruditapes philippinarum. *Chemosphere*, **152**, 10–16. <https://doi.org/10.1016/j.chemosphere.2016.02.049>
63. Takishita, K., Takaki, Y., Chikaraishi, Y., Ikuta, T., Ozawa, G., Yoshida, T., Ohkouchi, N., & Fujikura, K. (2017). Genomic evidence thatmethanotrophic endosymbionts likely providedeep-sea bathymodiolus musselswith a sterol intermediate in cholesterol biosynthesis. *Genome Biology and Evolution* **9**, 1148–1160. <https://doi.org/10.1093/gbe/evx082>
64. Tian, S., Pan, L., & Sun, X. (2013). An investigation of endocrine disrupting effects and toxic mechanisms modulated by benzo[a]pyrene in female scallop Chlamys farreri. *Aquatic Toxicology* **144–145**, 162–171. <https://doi.org/10.1016/j.aquatox.2013.09.031>
65. Tian, S., Pan, L., Tao, Y., & Sun, X. (2015). Environmentally relevant concentrations of benzo[a]pyrene affect steroid levels and affect gonad of male scallop Chlamys farreri. *Ecotoxicology and Environmental Safety* **114**, 150–156. <https://doi.org/10.1016/j.ecoenv.2015.01.019>
66. Wang, S., Ji, C., Li, F., Zhan, J., Sun, T., Tang, J., & Wu, H. (2021). Tetrabromobisphenol A induced reproductive endocrine-disrupting effects in mussel Mytilus galloprovincialis. *Journal of Hazardous Materials* **416**, 126228. <https://doi.org/10.1016/j.jhazmat.2021.126228>
67. Wang, T., Kong, H., Shang, Y., Dupont, S., Peng, J., Wang, X., Deng, Y., Hu, M., & Wang, Y. (2021). Ocean acidification but not hypoxia alters the gonad performance in the thick shell mussel Mytilus coruscus. *Marine Pollution Bulletin* **167**, 112 282. <https://doi.org/10.1016/j.marpolbul.2021.112282>
68. Yang, Y., Pan, L., Zhou, Y., Xu, R., & Li, D. (2020). Benzo[a]pyrene exposure disrupts steroidogenesis and impairs spermatogenesis in diverse reproductive stages of male scallop (Chlamys farreri). *Environmental Research* **191**, 110–125. <https://doi.org/10.1016/j.envres.2020.110125>
69. Zabrzańska, S., Smolarz, K., Hallmann, A., Konieczna, L., Baczek, T., & Wołowicz, M. (2015). Sex-related differences in steroid concentrations in the blue mussel (Mytilus edulis trossulus) from the southern Baltic Sea. Comparative Biochemistry and Physiology Part A : *Molecular and Integrative Physiology* **183**, 14–19. <https://doi.org/10.1016/j.cbpa.2014.12.029>
70. Zapata-Restrepo, L. M., Hauton, C., Williams, I. D., Jensen, A. C., & Hudson, M. D. (2019). Effects of the interaction between temperature and steroid hormones on gametogenesis and sex ratio in the European flat oyster (Ostrea edulis). *Comparative Biochemistry and Physiology Part A : Molecular and Integrative Physiology* **236,** 110523. <https://doi.org/10.1016/j.cbpa.2019.06.023>
71. Zhang, M., Wei, H., Liu, T., Li, W., Li, Y., Wang, S., Xing, Q., Hu, X., Zhang, L., & Bao, Z. (2020). Potential GnRH and steroidogenesis pathways in the scallop Patinopecten yessoensis. *Journal of Steroid Biochemistry and Molecular Biology* **204**, 105756. <https://doi.org/10.1016/j.jsbmb.2020.105756>
72. Zhang, Z., Bai, Q., Xu, X., & Zhang, X. (2021). Effects of the dominance hierarchy on social interactions, cortisol level, HPG-axis activities and reproductive success in the golden cuttlefish Sepia esculenta. *Aquaculture* **533**, 736059. <https://doi.org/10.1016/j.aquaculture.2020.736059>
73. Zheng, B. H., An, L. H., Chang, H., Liu, Y., & Jiang, Z. Q. (2014). Evidence for the presence of sex steroid hormones in Zhikong scallop, Chlamys farreri. *Journal of Steroid Biochemistry and Molecular Biology* **143**, 199–206. <https://doi.org/10.1016/j.jsbmb.2014.03.002>
74. Zhu, X., Guo, C., Lin, C., Wang, D., Wang, C., & Xu, S. (2018). Estradiol-17β and testosterone levels during the annual reproductive cycle of in Mytilus coruscus. Animal Reproduction Science 196, 35–42. https://doi.org/10.1016/j.anireprosci.2018.06.01

**4.2. Mollusca AND Receptors**

1. Abidli, S., Castro, L. F. C., Lahbib, Y., Reis-Henriques, M. A., Trigui El Menif, N., & Santos, M. M. (2013). Imposex development in Hexaplex trunculus (Gastropoda: Caenogastropoda) involves changes in the transcription levels of the retinoid X receptor (RXR). *Chemosphere* **93**, 1161–1167. <https://doi.org/10.1016/j.chemosphere.2013.06.054>
2. Agnese, M., Rosati, L., Prisco, M., Borzacchiello, L., Abagnale, L., & Andreuccetti, P. (2019). The expression of estrogen receptors during the Mytilus galloprovincialis ovarian cycle*. Journal of Experimental Zoology Part A: Ecological and Integrative Physiology* **331**, 367–373. <https://doi.org/10.1002/jez.2272>
3. André, A., Ruivo, R., Fonseca, E., Froufe, E., Castro, L. F. C., & Santos, M. M. (2019). The retinoic acid receptor (RAR) in molluscs: Function, evolution and endocrine disruption insights. *Aquatic Toxicology* **208**, 80–89. <https://doi.org/10.1016/j.aquatox.2019.01.002>
4. Aquilino, M., Martínez-Guitarte, J. L., García, P., Beltrán, E. M., Fernández, C., & Sánchez-Argüello, P. (2018). Combining the assessment of apical endpoints and gene expression in the freshwater snail Physa acuta after exposure to reclaimed water. *Science of the Total Environment* **642**, 80–189. <https://doi.org/10.1016/j.scitotenv.2018.06.054>
5. Aquilino, M., Sánchez-Argüello, P., Novo, M., & Martínez-Guitarte, J. L. (2019). Effects on tadpole snail gene expression after exposure to vinclozolin. *Ecotoxicology and Environmental Safety* **170**, 568–577. <https://doi.org/10.1016/j.ecoenv.2018.12.015>
6. Balbi, T., Franzellitti, S., Fabbri, R., Montagna, M., Fabbri, E., & Canesi, L. (2016). Impact of bisphenol A (BPA) on early embryo development in the marine mussel Mytilus galloprovincialis: Effects on gene transcription. *Environmental Pollution* **218**, 996–1004. <https://doi.org/10.1016/j.envpol.2016.08.050>
7. Bannister, R., Beresford, N., Granger, D. W., Pounds, N. A., Rand-Weaver, M., White, R., Jobling, S., & Routledge, E. J. (2013). No substantial changes in estrogen receptor and estrogen-related receptor orthologue gene transcription in Marisa cornuarietis exposed to estrogenic chemicals. *Aquatic Toxicology* **140–141**, 19–26. <https://doi.org/10.1016/j.aquatox.2013.05.002>
8. Bouétard, A., Besnard, A. L., Vassaux, D., Lagadic, L., & Coutellec, M. A. (2013). Impact of the redox-cycling herbicide diquat on transcript expression and antioxidant enzymatic activities of the freshwater snail Lymnaea stagnalis. *Aquatic Toxicology* **126**, 256–265. <https://doi.org/10.1016/j.aquatox.2012.11.013>
9. Capitão, A. M. F., Lopes-Marques, M., Páscoa, I., Sainath, S. B., Hiromori, Y., Matsumaru, D., Nakanishi, T., Ruivo, R., Santos, M. M., & Castro, L. F. C. (2021). An ancestral nuclear receptor couple, PPAR-RXR, is exploited by organotins. *Science of the Total Environment* **797**, 149044. <https://doi.org/10.1016/j.scitotenv.2021.149044>
10. Carpenter, S., Rothwell, C. M., Wright, M. L., de Hoog, E., Walker, S., Hudson, E., & Spencer, G. E. (2016). Extending the duration of long-term memories: Interactions between environmental darkness and retinoid signaling. *Neurobiology of Learning and Memory* **136**, 34–46. <https://doi.org/10.1016/j.nlm.2016.09.008>
11. Carter, C. J., Rand, C., Mohammad, I., Lepp, A., Vesprini, N., Wiebe, O., Carlone, R., & Spencer, G. E. (2015). Expression of a retinoic acid receptor (RAR)-like protein in the embryonic and adult nervous system of a protostome species. *Journal of Experimental Zoology Part B: Molecular and Developmental Evolution* **324**, 51–67. <https://doi.org/10.1002/jez.b.22604>
12. De Lisa, E., Paolucci, M., & Di Cosmo, A. (2012). Conservative Nature of Oestradiol Signalling Pathways in the Brain Lobes of Octopus vulgaris Involved in Reproduction, Learning and Motor Coordination. *Journal of Neuroendocrinology* **24**, 275–284. <https://doi.org/10.1111/j.1365-2826.2011.02240.x>
13. Deng, X., Pan, L., Cai, Y., & Jin, Q. (2016). Transcriptomic changes in the ovaries of scallop Chlamys farreri exposed to benzo[a]pyrene. *Genes and Genomics* **38**, 509–518. <https://doi.org/10.1007/s13258-016-0397-3>
14. Ding, M., Jiang, S., Miao, J., & Pan, L. (2021). Possible roles of gonadotropin-releasing hormone (GnRH) and melatonin in the control of gonadal development of clam Ruditapes philippinarum. *Comparative Biochemistry and Physiology Part A : Molecular & Integrative Physiology* **262**, 111059. <https://doi.org/10.1016/j.cbpa.2021.111059>
15. Gesto, M., Ruivo, R., Páscoa, I., André, A., Castro, L. F. C., & Santos, M. M. (2016). Retinoid level dynamics during gonad recycling in the limpet Patella vulgata. General and Comparative Endocrinology, 225, 142–148. <https://doi.org/10.1016/j.ygcen.2015.10.017>
16. Giraud-Billoud, M., & Castro-Vazquez, A. (2019). Aging and retinoid X receptor agonists on masculinization of female Pomacea canaliculata, with a critical appraisal of imposex evaluation in the Ampullariidae. *Ecotoxicology and Environmental Safety* **169**, 573–582. <https://doi.org/10.1016/j.ecoenv.2018.10.096>
17. Giulianelli, S., Primost, M. A., Lanari, C., & Bigatti, G. (2020). RXR Expression in Marine Gastropods with Different Sensitivity to Imposex Development. *Scientific Reports* **10**, 9507. <https://doi.org/10.1038/s41598-020-66402-1>
18. Gust, M., Gagné, F., Berlioz-Barbier, A., Besse, J. P., Buronfosse, T., Tournier, M., Tutundjian, R., Garric, J., & Cren-Olivé, C. (2014). Caged mudsnail Potamopyrgus antipodarum (Gray) as an integrated field biomonitoring tool: Exposure assessment and reprotoxic effects of water column contamination. *Water Research* **54**, 222–236. <https://doi.org/10.1016/j.watres.2014.01.057>
19. Gutierrez-Mazariegos, J., Nadendla, E. K., Lima, D., Pierzchalski, K., Jones, J. W., Kane, M., Nishikawa, J. I., Hiromori, Y., Nakanishi, T., Santos, M. M., Castro, L. F. C., Bourguet, W., Schubert, M., & Laudet, V. (2014). A mollusk retinoic acid receptor (RAR) ortholog sheds light on the evolution of ligand binding. *Endocrinology* **155**, 4275–4286. <https://doi.org/10.1210/en.2014-1181>
20. Huang, W., Wu, Q., Xu, F., Li, L., Li, J., Que, H., & Zhang, G. (2020). Functional characterization of retinoid X receptor with an emphasis on the mediation of organotin poisoning in the Pacific oyster (Crassostrea gigas). *Gene* **753**, 144780. <https://doi.org/10.1016/j.gene.2020.144780>
21. Huang, W., Xu, F., Li, J., Li, L., Que, H., & Zhang, G. (2015). Evolution of a novel nuclear receptor subfamily with emphasis on the member from the Pacific oyster Crassostrea gigas. *Gene* **567**, 164–172. <https://doi.org/10.1016/j.gene.2015.04.082>
22. Huang, W., Xu, F., Li, L., Que, H., & Zhang, G. (2019). The transcription of iodothyronine deiodinase genes is regulated by thyroid hormone receptor in the Pacific oyster Crassostrea gigas. *Journal of Oceanology and Limnology* **37**, 1317–1323. <https://doi.org/10.1007/s00343-019-8207-9>
23. Huang, W., Xu, F., Qu, T., Zhang, R., Li, L., Que, H., & Zhang, G. (2015). Identification of thyroid hormones and functional characterization of thyroid hormone receptor in the pacific oyster Crassostrea gigas provide insight into evolution of the thyroid hormone system. *PLoS ONE* **10**, 1–20. <https://doi.org/10.1371/journal.pone.0144991>
24. Hultin, C. L., Hallgren, P., & Hansson, M. C. (2016). Estrogen receptor genes in gastropods: Phylogenetic divergence and gene expression responses to a synthetic estrogen. *Comparative Biochemistry and Physiology Part C: Toxicology and Pharmacology* **189**, 17–21. <https://doi.org/10.1016/j.cbpc.2016.07.002>
25. Hultin, C. L., Hallgren, P., Persson, A., & Hansson, M. C. (2014). Identification of an estrogen receptor gene in the natural freshwater snail Bithynia tentaculata. *Gene* **540**, 26–31. <https://doi.org/10.1016/j.gene.2014.02.039>
26. Ip, J. C. H., Leung, P. T. Y., Ho, K. K. Y., Qiu, J. W., & Leung, K. M. Y. (2016). De novo transcriptome assembly of the marine gastropod Reishia clavigera for supporting toxic mechanism studies. *Aquatic Toxicology* **178**, 39–48. <https://doi.org/10.1016/j.aquatox.2016.07.006>
27. Jin, K., Jin, Q., Cai, Z., Huang, B., Wei, L., Zhang, M., Guo, W., Liu, Y., & Wang, X. (2021). Molecular Characterization of Retinoic Acid Receptor CgRAR in Pacific Oyster (Crassostrea gigas). *Frontiers in Physiology* **12**, 1–6. <https://doi.org/10.3389/fphys.2021.666842>
28. Johnson, A., de Hoog, E., Tolentino, M., Nasser, T., & Spencer, G. E. (2019). Pharmacological evidence for the role of RAR in axon guidance and embryonic development of a protostome species. *Genesis* **57**, 1–14. <https://doi.org/10.1002/dvg.23301>
29. Jouaux, A., Blin, J. L., Adeline, B., Heude-Berthelin, C., Sourdaine, P., Mathieu, M., & Kellner, K. (2013). Impact of energy storage strategies on gametogenesis and reproductive effort in diploid and triploid Pacific oysters Crassostrea gigas—Involvement of insulin signaling. *Aquaculture* **388–391**, 173–181. <https://doi.org/10.1016/j.aquaculture.2013.01.009>
30. Juárez, O. E., López-Galindo, L., Pérez-Carrasco, L., Lago-Lestón, A., Rosas, C., Cosmo, A. D., & Galindo-Sánchez, C. E. (2019). Octopus maya white body show sex-specific transcriptomic profiles during the reproductive phase, with high differentiation in signaling pathways. *PLoS ONE* **14**, 1–29. <https://doi.org/10.1371/journal.pone.0216982>
31. Lecomte, V., Noury, P., Tutundjian, R., Buronfosse, T., Garric, J., & Gust, M. (2013). Organic solvents impair life-traits and biomarkers in the New Zealand mudsnail Potamopyrgus antipodarum (Gray) at concentrations below OECD recommendations. *Aquatic Toxicology* **140–141**, 196–203. <https://doi.org/10.1016/j.aquatox.2013.06.006>
32. Lesoway, M. P., & Henry, J. Q. (2021). Retinoids promote penis development in sequentially hermaphroditic snails. *Developmental Biology* **478**, 122–132. <https://doi.org/10.1016/j.ydbio.2021.06.013>
33. Li, H., Liu, J., Huang, X., Wang, D., & Zhang, Z. (2014). Characterization, expression and function analysis of DAX1 gene of scallop (Chlamys farreri jones and preston 1904) during its gametogenesis*. Journal of Ocean University of China* **13**, 696–704. <https://doi.org/10.1007/s11802-014-2299-9>
34. Li, Y. F., Cheng, Y. L., Chen, K., Cheng, Z. Y., Zhu, X., C. R. Cardoso, J., Liang, X., Zhu, Y. T., Power, D. M., & Yang, J. L. (2020). Thyroid hormone receptor: A new player in epinephrine-induced larval metamorphosis of the hard-shelled mussel. *General and Comparative Endocrinology* **287**, 113347. <https://doi.org/10.1016/j.ygcen.2019.113347>
35. Liu, H., Zhang, H., & Zheng, H. (2018). Regulatory roles of sterol regulatory element-binding protein (SREBP) on lipid metabolism in the marine invertebrate Chlamys nobilis. Aquaculture, 493, 251–257. <https://doi.org/10.1016/j.aquaculture.2018.03.023>
36. Lü, Z. M., Liu, W., Liu, L. Q., Wang, T. M., Shi, H. L., Ping, H. L., Chi, C. F., Yang, J. W., & Wu, C. W. (2016). Cloning, Characterization, and Expression Profile of Estrogen Receptor in Common Chinese Cuttlefish, Sepiella japonica. Journal of Experimental Zoology Part A: *Ecological Genetics and Physiology* **325**, 181–193. <https://doi.org/10.1002/jez.2011>
37. Lü, Z., Zhu, K., Pang, Z., Liu, L., Jiang, L., Liu, B., Shi, H., Ping, H., Chi, C., & Gong, L. (2019). Identification, characterization and mRNA transcript abundance profiles of estrogen related receptor (ERR) in Sepiella japonica imply its possible involvement in female reproduction. *Animal Reproduction Science* **211**, 106231. <https://doi.org/10.1016/j.anireprosci.2019.106231>
38. Lv, J., Feng, L., Bao, Z., Guo, H., Zhang, Y., Jiao, W., Zhang, L., Wang, S., He, Y., & Hu, X. (2013). Molecular Characterization of RXR (Retinoid X Receptor) Gene Isoforms from the Bivalve Species Chlamys farreri. *PLoS ONE* **8**, 1–9. <https://doi.org/10.1371/journal.pone.0074290>
39. Ma, F., Han, X., An, L., Lei, K., Qi, H., & LeBlanc, G. A. (2019). Freshwater snail Parafossarulus striatulus estrogen receptor: Characteristics and expression profiles under lab and field exposure. *Chemosphere* **220**, 611–619. <https://doi.org/10.1016/j.chemosphere.2018.12.176>
40. Martínez-Paz, P., Morales, M., Sánchez-Argüello, P., Morcillo, G., & Martínez-Guitarte, J. L. (2017). Cadmium in vivo exposure alters stress response and endocrine-related genes in the freshwater snail Physa acuta. New biomarker genes in a new model organism. *Environmental Pollution* **220**, 1488–1497. <https://doi.org/10.1016/j.envpol.2016.10.012>
41. Nagasawa, K., Treen, N., Kondo, R., Otoki, Y., Itoh, N., Rotchell, J. M., & Osada, M. (2015). Molecular characterization of an estrogen receptor and estrogen-related receptor and their autoregulatory capabilities in two Mytilus species. *Gene* **564**, 153–159. <https://doi.org/10.1016/j.gene.2015.03.073>
42. Ni, J., Zeng, Z., & Ke, C. (2013). Sex steroid levels and expression patterns of estrogen receptor gene in the oyster Crassostrea angulata during the reproductive cycle. Aquaculture, 376–379, 105–116. https://doi.org/10.1016/j.aquaculture.2012.11.023
43. Omran, N. E. S. E. S. (2012). Testosterone, gonadotropins and androgen receptor during spermatogenesis of Biomphalaria alexandrina snails (Pulmonata: Basommatophora). *Reproductive Biology* **12**, 301–308. <https://doi.org/10.1016/j.repbio.2012.10.002>
44. Pang, Z., Lü, Z., Wang, M., Gong, L., Liu, B., Jiang, L., & Liu, L. (2019). Characterization, relative abundances of mRNA transcripts, and subcellular localization of two forms of membrane progestin receptors (mPRs) in the common Chinese cuttlefish, Sepiella japonica. *Animal Reproduction Science* **208**, 106107. <https://doi.org/10.1016/j.anireprosci.2019.106107>
45. Pes, K., Friese, A., Cox, C. J., Laizé, V., & Fernández, I. (2021). Biochemical and molecular responses of the Mediterranean mussel (Mytilus galloprovincialis) to short-term exposure to three commonly prescribed drugs. *Marine Environmental Research*, **168**, 105309. <https://doi.org/10.1016/j.marenvres.2021.105309>
46. Pryce, K., Samuel, D., Lagares, E., Myrthil, M., Bess, F., Harris, A., Welsh, C., Carroll, M. A., & Catapane, E. J. (2015). Presence of Octopamine and an Octopamine Receptor in Crassostrea virginica. *In Vivo* **37**, 16–24.
47. Raingeard, D., Bilbao, E., Cancio, I., & Cajaraville, M. P. (2013). Retinoid X receptor (RXR), estrogen receptor (ER) and other nuclear receptors in tissues of the mussel Mytilus galloprovincialis: Cloning and transcription pattern. *Comparative Biochemistry and Physiology Part A: Molecular and Integrative Physiology* **165**, 178–190. <https://doi.org/10.1016/j.cbpa.2013.03.001>
48. Rojas-garcía, A. E., Robledo-marenco, M. L., & Barrón-vivanco, B. S. (2014). Exposure to tributyltin chloride induces penis and vas deferens development and increases RXR expression in females of the purple snail ( Plicopurpura pansa ) Abstract Tributyltin ( TBT ) and its derivatives are widely used as antifouling paints for ships. *ISJ – Invertebrate Survival journal* **11**, 204–212.
49. Rosati, L., Agnese, M., Verderame, M., Aniello, F., Venditti, M., Mita, D. G., Andreuccetti, P., & Prisco, M. (2019a). Morphological and molecular responses in ovaries of Mytilus galloprovincialis collected in two different sites of the Naples Bay. *Journal of Experimental Zoology Part A: Ecological and Integrative Physiology* **331**, 52–60. <https://doi.org/10.1002/jez.2231>
50. Shi, Y., Guan, Y., & He, M. (2013). Molecular identification of insulin-related peptide receptor and its potential role in regulating development in Pinctada fucata. *Aquaculture* **408–409**, 118–127. <https://doi.org/10.1016/j.aquaculture.2013.05.038>
51. Stange, D., & Oehlmann, J. (2012a). Identification of oestrogen-responsive transcripts in Potamopyrgus antipodarum. *Journal of Molluscan Studies* **78**, 337–342. <https://doi.org/10.1093/mollus/eys019>
52. Stange, D., Sieratowicz, A., Horres, R., & Oehlmann, J. (2012b). Freshwater mudsnail (Potamopyrgus antipodarum) estrogen receptor: Identification and expression analysis under exposure to (xeno-)hormones. *Ecotoxicology and Environmental Safety*, **75**, 94–101. <https://doi.org/10.1016/j.ecoenv.2011.09.003>
53. Tan, K., Guo, Z., Zhang, H., Ma, H., Li, S., & Zheng, H. (2021). Carotenoids regulation in polymorphic noble scallops Chlamys nobilis under different light cycle. *Aquaculture* **531**, 735937. <https://doi.org/10.1016/j.aquaculture.2020.735937>
54. Tian, S., Pan, L., & Sun, X. (2013). An investigation of endocrine disrupting effects and toxic mechanisms modulated by benzo[a]pyrene in female scallop Chlamys farreri. *Aquatic Toxicology* **144–145**, 162–171. <https://doi.org/10.1016/j.aquatox.2013.09.031>
55. Tong, Y., Zhang, Y., Huang, J., Xiao, S., Zhang, Y., Li, J., Chen, J., & Yu, Z. (2015). Transcriptomics analysis of crassostrea hongkongensis for the discovery of reproduction-related genes. *PLoS ONE* **10**, 1–24. <https://doi.org/10.1371/journal.pone.0134280>
56. Tran, T. K. A., MacFarlane, G. R., Kong, R. Y. C., O’Connor, W. A., & Yu, R. M. K. (2016). Potential mechanisms underlying estrogen-induced expression of the molluscan estrogen receptor (ER) gene. *Aquatic Toxicology* **179**, 82–94. <https://doi.org/10.1016/j.aquatox.2016.08.015>
57. Urushitani, H., Katsu, Y., Kagechika, H., Sousa, A. C. A., Barroso, C. M., Ohta, Y., Shiraishi, H., Iguchi, T., & Horiguchi, T. (2018). Characterization and comparison of transcriptional activities of the retinoid X receptors by various organotin compounds in three prosobranch gastropods; Thais clavigera, Nucella lapillus and Babylonia japonica. *Aquatic Toxicology* **199**, 103–115. <https://doi.org/10.1016/j.aquatox.2018.03.029>
58. Urushitani, H., Katsu, Y., Ohta, Y., Shiraishi, H., Iguchi, T., & Horiguchi, T. (2013). Cloning and characterization of the retinoic acid receptor-like protein in the rock shell, Thais clavigera. *Aquatic Toxicology* **142–143**, 403–413. <https://doi.org/10.1016/j.aquatox.2013.09.008>
59. Vogeler, S., Bean, T. P., Lyons, B. P., & Galloway, T. S. (2016). Dynamics of nuclear receptor gene expression during Pacific oyster development. *BMC Developmental Biology* **16**, 33. <https://doi.org/10.1186/s12861-016-0129-6>
60. Völker, C., Gräf, T., Schneider, I., Oetken, M., & Oehlmann, J. (2014). Combined effects of silver nanoparticles and 17α-ethinylestradiol on the freshwater mudsnail Potamopyrgus antipodarum. *Environmental Science and Pollution Research* **21**, 10661–10670. <https://doi.org/10.1007/s11356-014-3067-5>
61. Wang, F., Cai, W., Shi, W., Wu, H., Shen, Q., He, Y., Cui, S., & An, L. (2021). Single molecule real-time sequencing revealing novel insights on the response to estrogen and androgen exposure in freshwater snails. *Aquatic Toxicology* **239**, 105953. <https://doi.org/10.1016/j.aquatox.2021.105953>
62. Wang, Q., & He, M. (2014). Molecular characterization and analysis of a putative 5-HT receptor involved in reproduction process of the pearl oyster Pinctada fucata. *General and Comparative Endocrinology* **204**, 71–79. <https://doi.org/10.1016/j.ygcen.2014.05.010>
63. White, S. H., Carter, C. J., & Magoski, N. S. (2014). A potentially novel nicotinic receptor in Aplysia neuroendocrine cells. *Journal of Neurophysiology* **112**, 446–462. <https://doi.org/10.1152/jn.00796.2013>
64. Xu, R., Pan, L., Yang, Y., & Zhou, Y. (2020). Characterizing transcriptome in female scallop Chlamys farreri provides new insights into the molecular mechanisms of reproductive regulation during ovarian development and spawn. *Gene* **758**, 144967. <https://doi.org/10.1016/j.gene.2020.144967>
65. Yan, L., Su, J., Wang, Z., Zhang, Y., Yan, X., & Yu, R. (2018). Growth performance and biochemical composition of the oysters Crassostrea sikamea, Crassostrea angulata and their hybrids in southern China. *Aquaculture Research* **49**, 1020–1028. <https://doi.org/10.1111/are.13549>
66. Yang, Y., Pan, L., Zhou, Y., Xu, R., & Li, D. (2020). Benzo[a]pyrene exposure disrupts steroidogenesis and impairs spermatogenesis in diverse reproductive stages of male scallop (Chlamys farreri). *Environmental Research* **191**, 110–125. <https://doi.org/10.1016/j.envres.2020.110125>
67. Zeng, M., Chen, D., Li, Q., Chen, H., & Huang, Q. (2020). Estrogen receptor regulates immune defense by suppressing NF-κB signaling in the Crassostrea hongkongensis. *Fish and Shellfish Immunology* **106**, 796–803. <https://doi.org/10.1016/j.fsi.2020.08.038>
68. Zhang, H., Pan, L., & Zhang, L. (2012). Molecular cloning and characterization of estrogen receptor gene in the Scallop Chlamys farreri: Expression profiles in response to endocrine disrupting chemicals. *Comparative Biochemistry and Physiology Part C: Toxicology and Pharmacology* **156**, 51–57. <https://doi.org/10.1016/j.cbpc.2012.03.007>
69. Zhang, M., Wei, H., Liu, T., Li, W., Li, Y., Wang, S., Xing, Q., Hu, X., Zhang, L., & Bao, Z. (2020). Potential GnRH and steroidogenesis pathways in the scallop Patinopecten yessoensis. *Journal of Steroid Biochemistry and Molecular Biology* **204**, 105756. <https://doi.org/10.1016/j.jsbmb.2020.105756>
70. Zhang, Y., Yu, F., Li, J., Tong, Y., Zhang, Y., & Yu, Z. (2014). The first invertebrate RIG-I-like receptor (RLR) homolog gene in the pacific oyster Crassostrea gigas. *Fish and Shellfish Immunology* **40**, 466–471. <https://doi.org/10.1016/j.fsi.2014.07.029>
71. Zhang, Z., Bai, Q., Xu, X., & Zhang, X. (2021). Effects of the dominance hierarchy on social interactions, cortisol level, HPG-axis activities and reproductive success in the golden cuttlefish Sepia esculenta. *Aquaculture* **533**, 736059. <https://doi.org/10.1016/j.aquaculture.2020.736059>
72. Zheng, B. H., An, L. H., Chang, H., Liu, Y., & Jiang, Z. Q. (2014). Evidence for the presence of sex steroid hormones in Zhikong scallop, Chlamys farreri. *Journal of Steroid Biochemistry and Molecular Biology* **143**, 199–206. <https://doi.org/10.1016/j.jsbmb.2014.03.002>

**4.3. Mollusca AND Enzymes**

1. Aquilino, M., Sánchez-Argüello, P., Novo, M., & Martínez-Guitarte, J. L. (2019). Effects on tadpole snail gene expression after exposure to vinclozolin. *Ecotoxicology and Environmental Safety* **170**, 568–577. <https://doi.org/10.1016/j.ecoenv.2018.12.015>
2. Baynes, A., Montagut Pino, G., Duong, G. H., Lockyer, A. E., McDougall, C., Jobling, S., & Routledge, E. J. (2019). Early embryonic exposure of freshwater gastropods to pharmaceutical 5-alpha-reductase inhibitors results in a surprising open-coiled “banana-shaped” shell. *Scientific Reports* **9**, 1–12. https://doi.org/10.1038/s41598-019-52850-x
3. Coelho, I., Lima, D., André, A., Melo, C., Ruivo, R., Reis-Henriques, M. A., Santos, M. M. H., & Castro, L. F. C. (2012). Molecular characterization of Adh3 from the mollusc Nucella lapillus: Tissue gene expression after tributyltin and retinol exposure. *Journal of Molluscan Studies* **78**, 343–348. <https://doi.org/10.1093/mollus/eys018>
4. Cubero-Leon, E., Puinean, A. M., Labadie, P., Ciocan, C., Itoh, N., Kishida, M., Osada, M., Minier, C., Hill, E. M., & Rotchell, J. M. (2012). Two CYP3A-like genes in the marine mussel Mytilus edulis: mRNA expression modulation following short-term exposure to endocrine disruptors. Marine Environmental Research, 74, 32–39. <https://doi.org/10.1016/j.marenvres.2011.11.012>
5. De Lisa, E., Paolucci, M., & Di Cosmo, A. (2012). Conservative Nature of Oestradiol Signalling Pathways in the Brain Lobes of Octopus vulgaris Involved in Reproduction, Learning and Motor Coordination. *Journal of Neuroendocrinology* **24**, 275–284. <https://doi.org/10.1111/j.1365-2826.2011.02240.x>
6. Deng, X., Pan, L., Cai, Y., & Jin, Q. (2016). Transcriptomic changes in the ovaries of scallop Chlamys farreri exposed to benzo[a]pyrene. *Genes and Genomics* **38**, 509–518. <https://doi.org/10.1007/s13258-016-0397-3>
7. Ding, M., Jiang, S., Miao, J., & Pan, L. (2021). Possible roles of gonadotropin-releasing hormone (GnRH) and melatonin in the control of gonadal development of clam Ruditapes philippinarum. *Comparative Biochemistry and Physiology Part A : Molecular & Integrative Physiology* **262**, 111059. <https://doi.org/10.1016/j.cbpa.2021.111059>
8. Huang, W., Xu, F., Qu, T., Li, L., Que, H., & Zhang, G. (2015). Iodothyronine deiodinase gene analysis of the Pacific oyster Crassostrea gigas reveals possible conservation of thyroid hormone feedback regulation mechanism in mollusks. *Chinese Journal of Oceanology and Limnology* **33**, 997–1006. <https://doi.org/10.1007/s00343-015-4300-x>
9. Ip, J. C. H., Leung, P. T. Y., Ho, K. K. Y., Qiu, J. W., & Leung, K. M. Y. (2016). De novo transcriptome assembly of the marine gastropod Reishia clavigera for supporting toxic mechanism studies. *Aquatic Toxicology* **178**, 39–48. <https://doi.org/10.1016/j.aquatox.2016.07.006>
10. Jiang, S., Miao, J., Wang, X., Liu, P., & Pan, L. (2019). Inhibition of growth in juvenile manila clam Ruditapes philippinarum: Potential adverse outcome pathway of TBBPA. *Chemosphere* **224**, 588–596. <https://doi.org/10.1016/j.chemosphere.2019.02.157>
11. Juárez, O. E., López-Galindo, L., Pérez-Carrasco, L., Lago-Lestón, A., Rosas, C., Cosmo, A. D., & Galindo-Sánchez, C. E. (2019). Octopus maya white body show sex-specific transcriptomic profiles during the reproductive phase, with high differentiation in signaling pathways. *PLoS ONE* **14**, 1–29. <https://doi.org/10.1371/journal.pone.0216982>
12. Lesoway, M. P., & Henry, J. Q. (2021). Retinoids promote penis development in sequentially hermaphroditic snails. Developmental Biology 478, 122–132. <https://doi.org/10.1016/j.ydbio.2021.06.013>
13. Lima, D., MacHado, A., Reis-Henriques, M. A., Rocha, E., Santos, M. M., & Castro, L. F. C. (2013). Cloning and expression analysis of the 17β hydroxysteroid dehydrogenase type 12 (HSD17B12) in the neogastropod Nucella lapillus. *Journal of Steroid Biochemistry and Molecular Biology*, **134**, 8–14. <https://doi.org/10.1016/j.jsbmb.2012.10.005>
14. Lin, C., Guo, C., Zhu, X., Wang, D., Xu, J., & Xu, S. (2019). Ovarian transcriptome analysis of Mactra chinensis provides insights into genes expressed during the intermediate and ripening stages. *Animal Reproduction Science* **208**, 106078. <https://doi.org/10.1016/j.anireprosci.2019.05.007>
15. Liu, J., Zhang, Z., Ma, X., Liang, S., & Yang, D. (2014b). Characteristics of 17β-hydroxysteroid dehydrogenase 8 and its potential role in gonad of Zhikong scallop Chlamys farreri. *Journal of Steroid Biochemistry and Molecular Biology* **141**, 77–86. <https://doi.org/10.1016/j.jsbmb.2014.01.008>
16. Liu, P., Miao, J., Song, Y., Pan, L., & Yin, P. (2017). Effects of 2,2’,4,4’-tetrabromodipheny ether (BDE-47) on gonadogenesis of the manila clam Ruditapes philippinarum. *Aquatic Toxicology* **193**, 178–186. <https://doi.org/10.1016/j.aquatox.2017.10.022>
17. Meng, X., Li, F., Wang, X., Liu, J., Ji, C., & Wu, H. (2019). Combinatorial immune and stress response, cytoskeleton and signal transduction effects of graphene and triphenyl phosphate (TPP) in mussel Mytilus galloprovincialis. *Journal of Hazardous Materials* **378**, 120778. <https://doi.org/10.1016/j.jhazmat.2019.120778>
18. Omran, N. E., & Salama, W. M. (2016). The endocrine disruptor effect of the herbicides atrazine and glyphosate on Biomphalaria alexandrina snails. Toxicology and Industrial Health, 32(4), 656–665. <https://doi.org/10.1177/0748233713506959>
19. Prisco, M., Agnese, M., De Marino, A., Andreuccetti, P., & Rosati, L. (2017). Spermatogenic Cycle and Steroidogenic Control of Spermatogenesis in Mytilus galloprovincialis Collected in the Bay of Naples. *Anatomical Record* **300**, 1881–1894. <https://doi.org/10.1002/ar.23626>
20. Rosati, L., Agnese, M., Abagnale, L., Aniello, F., Andreuccetti, P., & Prisco, M. (2019b). The Mussel Mytilus galloprovincialis in the Bay of Naples: New Insights on Oogenic Cycle and Its Hormonal Control. *Anatomical Record* **302**, 1039–1049. <https://doi.org/10.1002/ar.24075>
21. Rothwell, C. M., Simmons, J., Peters, G., & Spencer, G. E. (2014). Novel interactive effects of darkness and retinoid signaling in the ability to form long-term memory following aversive operant conditioning. *Neurobiology of Learning and Memory* **114**, 251–263. <https://doi.org/10.1016/j.nlm.2014.07.007>
22. Song, Y., Miao, J., Cai, Y., & Pan, L. (2015). Molecular cloning, characterization, and expression analysis of a gonadotropin-releasing hormone-like cDNA in the clam, Ruditapes philippinarum. *Comparative Biochemistry and Physiology Part B: Biochemistry and Molecular Biology* **189**, 47–54. <https://doi.org/10.1016/j.cbpb.2015.07.005>
23. Song, Y., Miao, J., Pan, L., & Wang, X. (2016). Exposure to2,2’,4,4’-tetrabromodiphenyl ether (BDE-47) alters thyroid hormone levels and thyroid hormone-regulated gene transcription in manila clam Ruditapes philippinarum. *Chemosphere*, **152**, 10–16. <https://doi.org/10.1016/j.chemosphere.2016.02.049>
24. Strehse, J. S., Brenner, M., Kisiela, M., & Maser, E. (2020). The explosive trinitrotoluene (TNT) induces gene expression of carbonyl reductase in the blue mussel (Mytilus spp.): A new promising biomarker for sea dumped war relicts? *Archives of Toxicology* **94**, 4043–4054. <https://doi.org/10.1007/s00204-020-02931-y>
25. Takishita, K., Takaki, Y., Chikaraishi, Y., Ikuta, T., Ozawa, G., Yoshida, T., Ohkouchi, N., & Fujikura, K. (2017). Genomic evidence thatmethanotrophic endosymbionts likely providedeep-sea bathymodiolus musselswith a sterol intermediate in cholesterol biosynthesis. *Genome Biology and Evolution* **9**, 1148–1160. <https://doi.org/10.1093/gbe/evx082>
26. Thitiphuree, T., Nagasawa, K., & Osada, M. (2019). Molecular identification of steroidogenesis-related genes in scallops and their potential roles in gametogenesis. *Journal of Steroid Biochemistry and Molecular Biology* **186**, 22–33. <https://doi.org/10.1016/j.jsbmb.2018.09.004>
27. Tian, S., Pan, L., & Sun, X. (2013). An investigation of endocrine disrupting effects and toxic mechanisms modulated by benzo[a]pyrene in female scallop Chlamys farreri. *Aquatic Toxicology* **144–145**, 162–171. <https://doi.org/10.1016/j.aquatox.2013.09.031>
28. Tian, S., Pan, L., & Zhang, H. (2014). Identification of a CYP3A-like gene and CYPs mRNA expression modulation following exposure to benzo[a]pyrene in the bivalve mollusk Chlamys farreri. *Marine Environmental Research* **94**, 7–15. <https://doi.org/10.1016/j.marenvres.2013.11.001>
29. Tong, Y., Zhang, Y., Huang, J., Xiao, S., Zhang, Y., Li, J., Chen, J., & Yu, Z. (2015). Transcriptomics analysis of crassostrea hongkongensis for the discovery of reproduction-related genes. *PLoS ONE* **10**, 1–24. <https://doi.org/10.1371/journal.pone.0134280>
30. Vöcking, O., Leclère, L., & Hausen, H. (2021). The rhodopsin-retinochrome system for retinal re-isomerization predates the origin of cephalopod eyes. *BMC Ecology and Evolution* **21**, 1–14. <https://doi.org/10.1186/s12862-021-01939-x>
31. Wang, S., Ji, C., Li, F., Zhan, J., Sun, T., Tang, J., & Wu, H. (2021). Tetrabromobisphenol A induced reproductive endocrine-disrupting effects in mussel Mytilus galloprovincialis. *Journal of Hazardous Materials* **416**, 126228. <https://doi.org/10.1016/j.jhazmat.2021.126228>
32. Wang, T., Kong, H., Shang, Y., Dupont, S., Peng, J., Wang, X., Deng, Y., Hu, M., & Wang, Y. (2021). Ocean acidification but not hypoxia alters the gonad performance in the thick shell mussel Mytilus coruscus. *Marine Pollution Bulletin* **167**, 112 282. <https://doi.org/10.1016/j.marpolbul.2021.112282>
33. Xu, R., Pan, L., Yang, Y., & Zhou, Y. (2020). Characterizing transcriptome in female scallop Chlamys farreri provides new insights into the molecular mechanisms of reproductive regulation during ovarian development and spawn. *Gene* **758**, 144967. <https://doi.org/10.1016/j.gene.2020.144967>
34. Yang, Y., Pan, L., Zhou, Y., Xu, R., & Li, D. (2020). Benzo[a]pyrene exposure disrupts steroidogenesis and impairs spermatogenesis in diverse reproductive stages of male scallop (Chlamys farreri). *Environmental Research* **191**, 110–125. <https://doi.org/10.1016/j.envres.2020.110125>
35. Zhai, H. N., Zhou, J., & Cai, Z. H. (2012). Cloning, characterization, and expression analysis of a putative 17 beta-hydroxysteroid dehydrogenase 11 in the abalone, Haliotis diversicolor supertexta. *Journal of Steroid Biochemistry and Molecular Biology* **130**, 57–63. <https://doi.org/10.1016/j.jsbmb.2011.12.013>
36. Zhang, M., Wei, H., Liu, T., Li, W., Li, Y., Wang, S., Xing, Q., Hu, X., Zhang, L., & Bao, Z. (2020). Potential GnRH and steroidogenesis pathways in the scallop Patinopecten yessoensis. *Journal of Steroid Biochemistry and Molecular Biology* **204**, 105756. <https://doi.org/10.1016/j.jsbmb.2020.105756>
37. Zhang, Y., Wang, Q., Ji, Y., Zhang, Q., Wu, H., Xie, J., & Zhao, J. (2014). Identification and mRNA expression of two 17β-hydroxysteroid dehydrogenase genes in the marine mussel Mytilus galloprovincialis following exposure to endocrine disrupting chemicals. *Environmental Toxicology and Pharmacology* **37**, 1243–1255. <https://doi.org/10.1016/j.etap.2014.04.027>
38. Zhang, Z., Bai, Q., Xu, X., & Zhang, X. (2021). Effects of the dominance hierarchy on social interactions, cortisol level, HPG-axis activities and reproductive success in the golden cuttlefish Sepia esculenta. *Aquaculture* **533**, 736059. <https://doi.org/10.1016/j.aquaculture.2020.736059>
